# Synthesis of Chiral Iodoaniline-Lactate Based Catalysts for the α-Functionalization of Ketones

**DOI:** 10.1021/acsorginorgau.3c00012

**Published:** 2023-05-09

**Authors:** Rawiyah Alkahtani, Thomas Wirth

**Affiliations:** † School of Chemistry, Cardiff University, Main Building, Park Place, CF10 3AT, Cardiff, United Kingdom; ‡ Chemistry Department, College of Science, Princess Nourah bint Abdulrahman University, 11671, Riyadh, Saudi Arabia

**Keywords:** Hypervalent iodine, Organocatalysis, Oxidation, Oxysulfonylation, Stereoselective synthesis

## Abstract

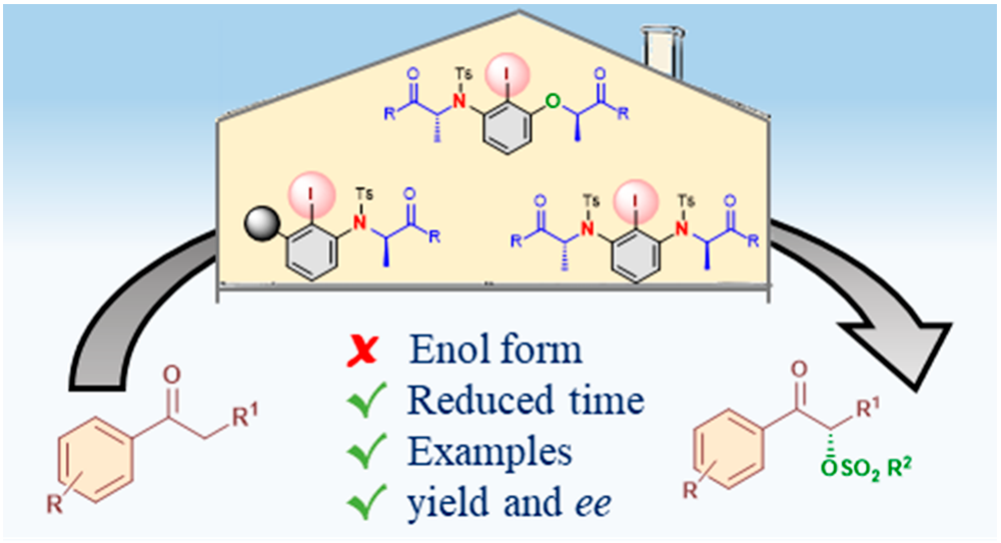

A family of chiral iodoaniline-lactate based catalysts with *C*_1_ and *C*_2_ symmetry were efficiently synthesized. Comparisons between the reactivity and selectivity between the new and previously reported catalysts are made. The new catalysts promoted the α-oxysulfonylation of ketones in shorter reaction times and with higher yields of up to 99%. A scope for the oxysulfonylation reaction is presented, forming a variety of reported and novel products with enantioselectivities of up to 83%.

Chiral iodoarene catalysts have become an environmentally and chemically green alternative to transition-metal-based catalysts due to their facile availability, low toxicity, versatile reactivity, high stability toward moisture and atmospheric oxygen, ease of recovery, and ease of handling.^1^ Over the past decades, enantioselective reactions catalyzed by chiral hypervalent iodine reagents or chiral iodoarene precatalysts have attracted significant attention. A variety of chiral iodoarene backbones have been reported,^2,3^ for instance, *C*_1_- or *C*_2_-symmetric compounds with central,^4^ axial biaryl,^5^ spirobiindane,^6^ or planar^7^ chirality. To date, lactate-based chiral hypervalent iodine reagents of type **6** are considered one of the most reported and successful catalysts (Scheme 1). Such catalysts are easily synthesized through coupling reactions between lactic acid derivatives **1** to iodophenol **2** or iodoresorcinol **3**.^8,9^ They can be subsequently oxidized to the corresponding hypervalent iodine(III) reagents by use of an oxidant such as 3-chloroperoxybenzoic acid (*m*CPBA),^10^ sodium perborate,^11^ or Selectfluor as reported independently by Fujita^11^ and Ishihara.^4,12^ Many modifications of this skeleton have been investigated by different groups where new derivatives were designed to obtain optimal results for specific applications.^9^

**Scheme 1:**
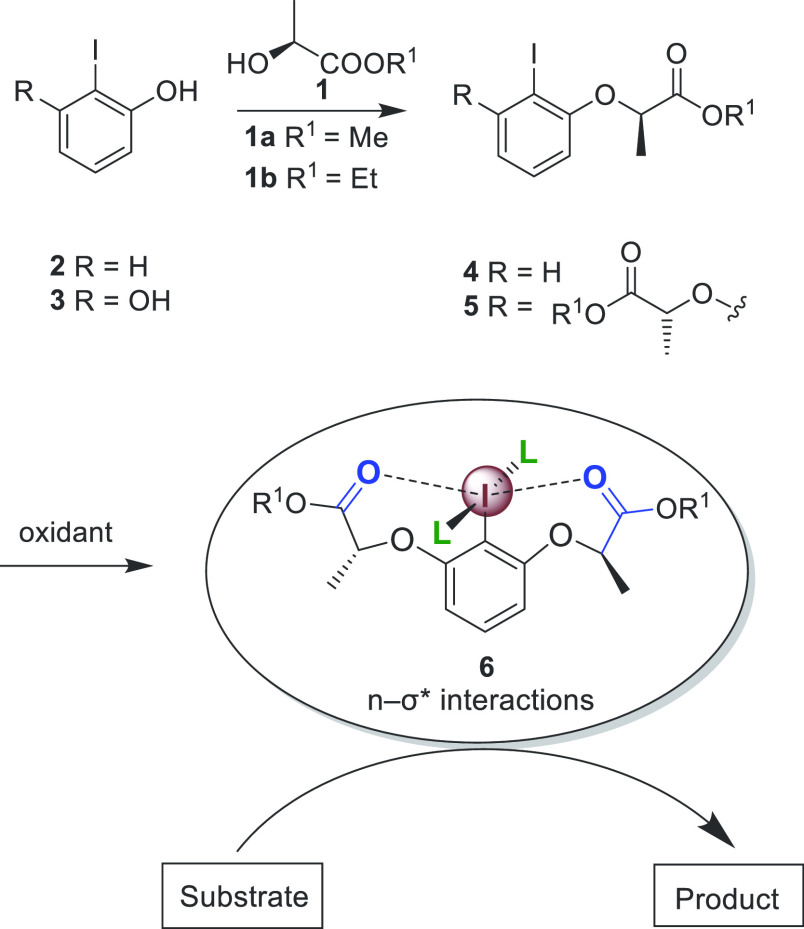
General Method for the Synthesis of Lactate-Based Chiral Hypervalent Iodine Reagents

Typically, high reactivities and selectivities for hypervalent iodine reagents of type **6** have been reported,^10,11,13−16^ and these reagents have been utilized within numerous synthetically useful oxidative transformations. The intramolecular n−σ* interaction between the carbonyl groups and the iodine(III) center demonstrates helical chirality around the iodine atom in compound **6**, which strongly influences the enantioinduction in stereoselective transformations (Scheme 1). We have reported the stereoselective dioxytosylation of styrene derivatives with chiral iodine(III) reagents for the first time;^17^ Fujita and co-workers used this reaction to investigate the performance of *C*_1_- and *C*_2_-lactate-based aryl-λ^3^-iodanes **7**.^18^ The desired 1,2-dioxytosylated product was generated with high yield and an enantioinduction of 90% ee, superior to our initial results (65% ee) (Scheme 2a). Later, we reported the first catalytic version of a stereoselective oxytosylation.^19^ Coeffard et al. reported a new methodology for the α-oxytosylation of α-substituted β-ketoesters with high yields and promising enantioselectivities using *C*_2_-symmetric iodoarenes **8** as chiral catalysts (Scheme 2b).^20^ Additionally, Legault et al. reported a modified version that enables the introduction of a tosylate nucleophile to the α-position of carbonyls with high yields and high enantiomeric excess using enol esters and chiral iodine catalyst **9** (Scheme 2c).^10^ This was an alternative strategy to the direct α-oxytosylation of ketones which resulted in much lower selectivities with the same catalysts.

**Scheme 2:**
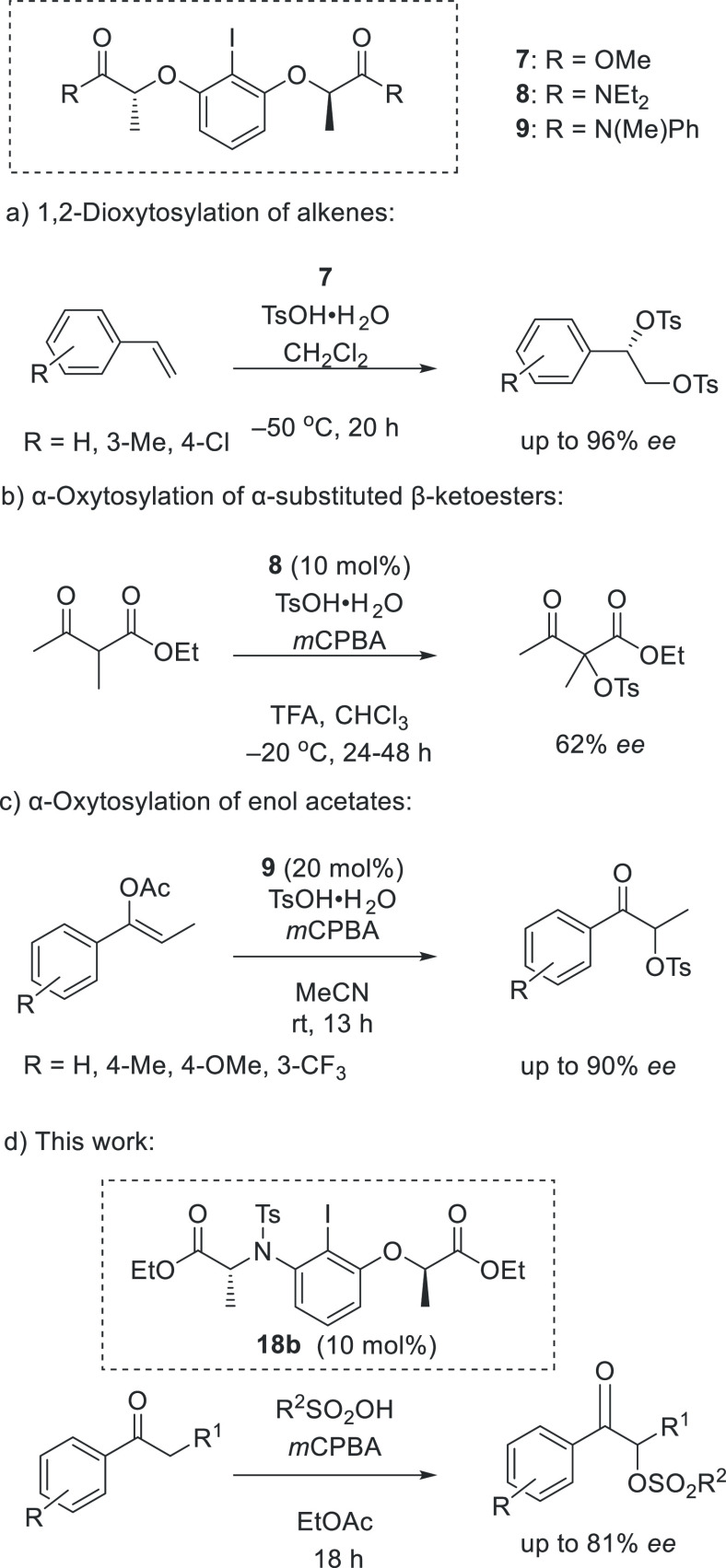
Previous Studies of Oxytosylations with *C*_1_- and *C*_2_-Symmetric Lactate-Based Chiral Iodine Catalysts

Herein, we report the synthesis of novel chiral iodoarene lactate-based catalysts where the oxygen atom in the previous versions of these catalysts has been replaced with a protected nitrogen atom (Scheme 2d). The reactivities and selectivities in the α-oxytosylation of ketones were compared between the different structures. The addition of general oxysulfonyl nucleophiles to the α-position of carbonyl compounds under catalytic reaction conditions was developed without the requisition of enol ethers as substrates.

The synthesis of this new family of chiral iodoarene catalysts commenced with the nitrogen protection of iodoaniline derivatives as secondary sulfonamides. Sulfonyl protecting groups are effective in protecting amines as their nucleophilicity and basicity is being reduced by this protection. (*S*)-Methyl lactate or (*S*)-ethyl lactate were then connected to the secondary sulfonamides through a Mitsunobu reaction.

Iodoaniline was protected effectively with high yields for the synthesis of compounds **12a**–**e** by using different sulfonyl chloride derivatives in the presence of pyridine in dichloromethane. Compound **12f** was prepared in 25% yield by using trifluoromethanesulfonic acid anhydride in the presence of triethylamine as a base. The protected amine derivatives **12** were then reacted successfully with (*S*)-methyl- or (*S*)-ethyl lactate under Mitsunobu reaction conditions (PPh_3_, DIAD), generating chiral iodoaniline catalysts **13a**–**g** in good yields (Scheme 3).

**Scheme 3:**
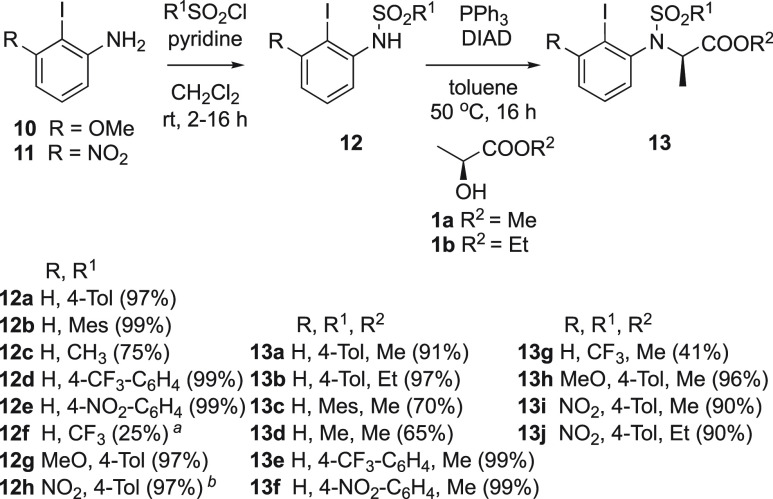
Synthesis of Novel Chiral Iodoaniline Catalysts Reaction conditions: (CF_3_SO_2_)_2_O, Et_3_N, CH_2_Cl_2_. Reaction conditions: 4-NO_2_–C_6_H_4_SO_2_Cl, pyridine, 4 h.

For the synthesis of the methoxy-substituted iodoarene **13h**, the iodine atom was introduced in the 2-position of 3-methoxyaniline following a reported procedure.^21^ The key intermediate **10** was protected with a tosyl group^22^ and then subjected to a Mitsunobu reaction, generating the desired chiral iodoarene **13h** in 96% yield as shown in Scheme 3.

For the synthesis of iodoarenes bearing an electron-withdrawing nitro group **13i** and **13j**, 2-iodo-3-nitroaniline **11** was prepared according to a literature procedure from 2,6-dinitroaniline through diazotation, introduction of iodine and reduction of one nitro group.^23^ In the reduction step, many attempts were performed to increase the yield of product **11**. Unfortunately, most attempts caused loss of the iodine atom and did not increase the yield. The protected amine **12h** was formed in high yield when the sulfonylation reaction was performed in the absence of dichloromethane, as compound **11** remains unreacted in the presence of solvent. Finally, iodoarene **12h** reacted with (*S*)-methyl- and (*S*)-ethyl lactate in a Mitsunobu reaction to afford **13i** and **13j** in high yields (Scheme 3).

To achieve the synthesis of the proposed nitrogen- and oxygen-linked chiral iodoarenes **18a** and **18b**, the key building block 2-iodo-3-aminophenol **15** is required. Using a modified literature procedure,^24,25^ compound **15** was synthesized starting from commercially available 2-amino-3-nitrophenol via diazotation and nitro group reduction in 74% yield over 2 steps (Scheme 4). With the key intermediate **15** in hand, the synthesis of the target catalysts could be achieved. Initially, iodoarene **15** was reacted with TsCl and pyridine to produce the protected amine **17a** in 90% yield. This was followed by the Mitsunobu reaction with (*S*)-methyl or (*S*)-ethyl lactate, producing the catalysts **18a** and **18b** in very good yields (Scheme 4).

**Scheme 4:**
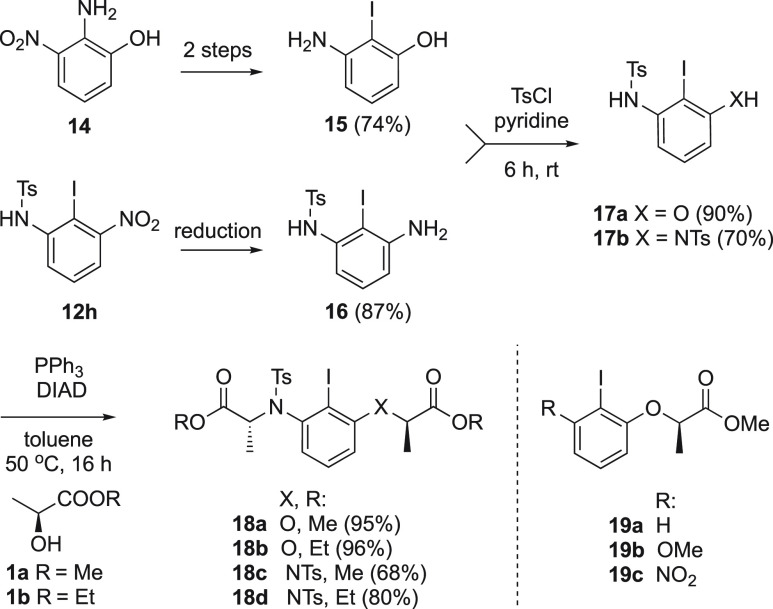
Synthesis of Novel Disubstituted Chiral Iodoaniline Catalysts

2-Iodobenzene-1,3-diamine was used to synthesize chiral iodoarene catalysts **18c** and **18d**. Several methods have been attempted to reduce both nitro groups of 2-iodo-1,3-dinitrobenzene to obtain the corresponding diamine. However, all such attempts were unsuccessful. Partial reductions and/or side reactions including deiodination were observed instead of the target product. Consequently, an alternative indirect route was devised to obtain the target 2-iodobenzene-1,3-diamine and subsequently the desired catalysts. The nitro group in compound **12h** (Scheme 3) was effectively reduced with tin chloride monohydrate to produce **16** in high yields. Subsequent tosyl protection under the same conditions as stated earlier allowed the formation of compound **17b** in 70% yield. The target catalysts **18c** and **18d** were prepared in good yields of 68% and 80%, respectively (Scheme 4).

After the successful synthesis of the target catalysts, the absolute configuration of the prepared iodoarenes **13a**, **13c**, **13d**, **13f**, **13h**, **13j**, and **18c** were confirmed through analysis of the X-ray crystallographic structures; some of them are shown in Figure 1.^26^

**Figure 1:**
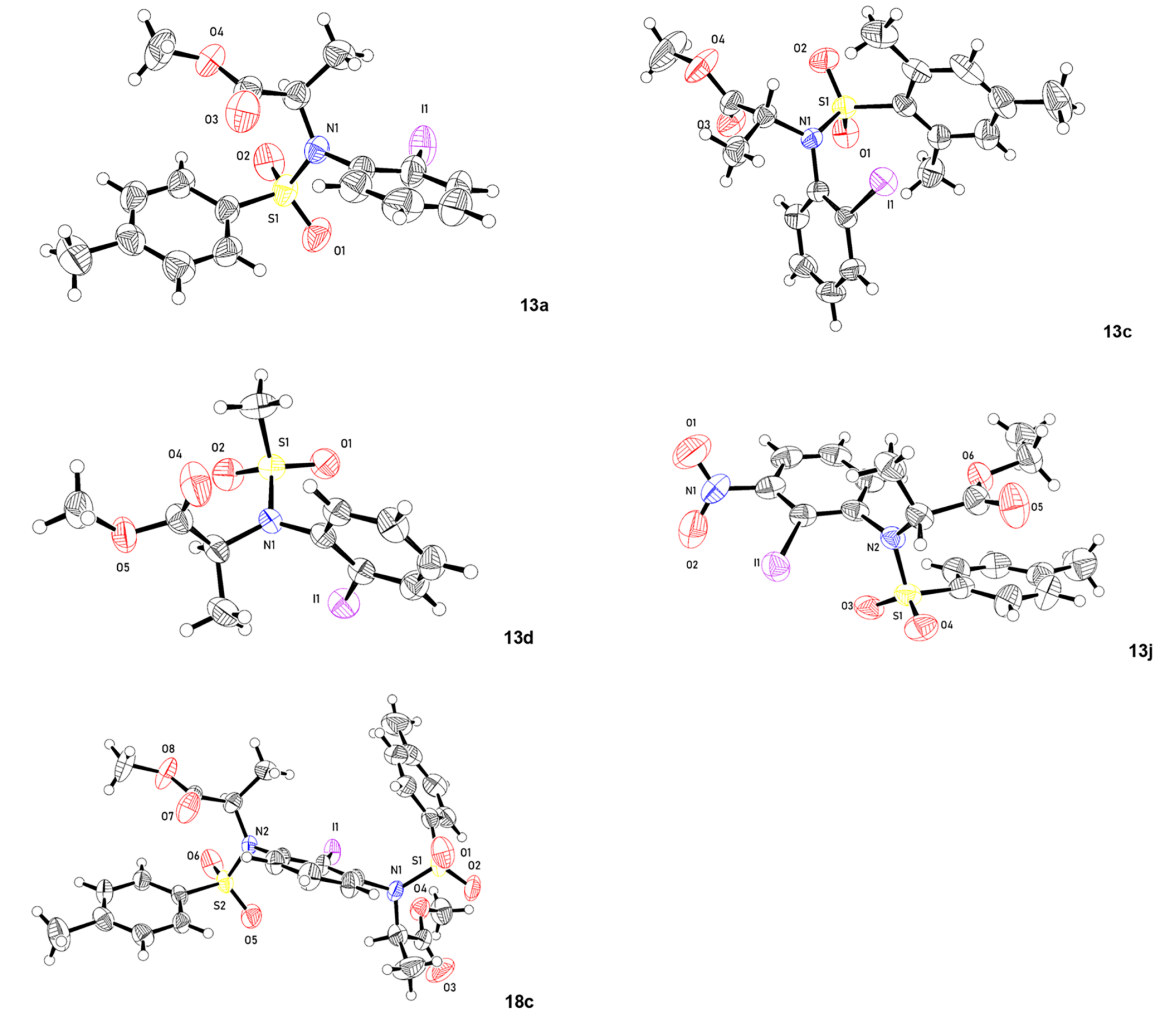
X-ray structures of some chiral iodoarenes, ellipsoid probability 50%.

When attempting the characterization of the catalysts, conformational isomers were observed. Duplicated signals for the protons and carbons in ^1^H and ^13^C NMR spectra were observed in various ratios, indicating the presence of conformational isomers. According to Karnik and Hasan, conformers are one compound with different rotations about single bonds.^27^ In the chiral molecules, this is due to the hindered amide rotation, which changes the dihedral angles between the vicinal groups.^28^

The catalyst **13j** was selected as a model to demonstrate the conformational isomers and substantiate this phenomenon. Initially, the ^1^H NMR analysis of **13j** was performed in different deuterated solvents at room temperature (Figure S1A, see SI). The conformers were found in all solvents, with almost identical peak splitting, signal ratios, *J* coupling constants, and integration values. However, the chemical shifts of the two conformers were slightly different, as expected given the properties of the solvents. For further analysis, detailed studies for the hydrogen atoms H^a^ and H^a^′ of **13j** at the chiral center were performed. The *J* coupling values were calculated in all solvents and gave approximately identical values of H^a^ and H^a^′, both being quartets with *J* = 7.0 Hz, giving a total integration of one proton. The ratio of the conformers was about 1:2.3 (Figure S1A, see SI). Moreover, the hindered amide rotation was further confirmed by temperature-variable ^1^H NMR analysis (Figure S1B, see SI). The ratio of H^a^:H^a^′ and chemical shifts of the peaks changed gradually from high to low temperatures. At a temperature of 65 °C, the rate of interconversion becomes faster, and conformers were observed with a ratio of 1:2. To reduce the interconversion between the conformers, the ^1^H NMR was performed at −25 °C. The peak ratio changed slightly to (1:2.4), and some overlapping peaks were detected. Finally, in solid state ^13^C NMR (SS ^13^C NMR), only singlet signals were detected, which supported the hypothesis that both conformers can be observed in solution, while only one conformer can be seen in the solid state (Figure S1C, see SI). In addition, only one conformer was identified using X-ray crystallography as presented in Figure 1. Alternatively, the observed conformers could also be due to atropisomerism as this has been observed for sulfonamides of comparable structures.^29^

After having prepared the iodoarene catalysts of type **7**, **13**, **18**, and **19**, the focus was directed toward studying their reactivity and their stereocontrol. The α-oxytosylation of ketones was selected for an initial screening. According to the literature conditions,^10,22,30^ propiophenone was chosen as the ketone substrate, the chiral iodoarenes of type **7**, **13**, **18**, and **19** were used as the organocatalysts in the presence of *m*CPBA as the terminal oxidant, *p*-toluenesulfonic acid monohydrate (TsOH·H_2_O) as the nucleophile, and acetonitrile as the solvent.

The results in Table 1 summarize the screening in the α-oxytosylation of propiophenone. The iodoarenes were able to mediate the introduction of the tosylate nucleophile at the α-position of propiophenone, and the desired product (*S*)-1-oxo-1-phenylpropan-2-yl 4-methylbenzenesulfonate **20a** was formed in good yields (up to 99%) using 10 mol % of **13a**–**h** or **18a**–**c**. The presence of a nitro group in the *ortho*-position of iodoarene catalysts **13i**–**j** and catalyst **18d** reduced the yields of **20a** significantly.

**Table 1:**
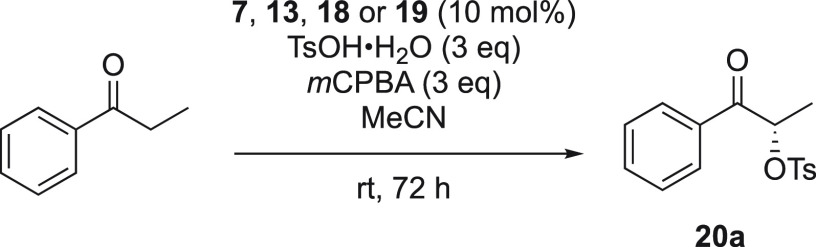
Screening of Pre-Catalysts in the Enantioselective α-Oxytosylation of Propiophenone^a^

entry	catalyst (ArI)	yield **20a** [%]^b^	ee [%]^c^
1	**13a**	95	34
2	**13b**	94	36
3	**13c**	89	14
4	**13d**	91	8
5	**13e**	88	22
6	**13f**	90	19
7	**13g**	75	1
8	**13h**	88	20
9	**13i**	30	27
10	**13j**	22	27
11	**18a**	97	33
12	**18b**	99	47
13	**18c**	91	16
14	**18d**	18	3
15	**19a**	96	16
16	**19b**	71	8
17	**19c**	50	17
18	**7**	87	15

a General method: propiophenone (0.37 mmol), ArI (0.037 mmol), *m*CPBA (1.12 mmol), and TsOH·H_2_O (1.12 mmol) in MeCN (2 mL), stirred at rt for 72 h.

b Isolated yields.

c Determined by chiral HPLC.

Following these results, a variety of substituted alkyl and aryl sulfonyl groups in catalysts **13a**–**g** were introduced to probe the influence of electron-donating, electron-withdrawing, and steric bulk on the reaction rate and product selectivity. Aryl groups attached to the sulfonamide sulfur atom in catalysts **13a**–**c**, **13e**, and **13f** showed higher catalyst reactivity than alkyl groups at that position, such as catalysts **13d** and **13g** (entries 1–7, Table 1). After examining the catalysts **13a**–**g** as presented in Table 1, we observed that a tosyl (Ts) group was the best sulfonyl protecting group of the nitrogen atom in this type of catalyst. Notably, the presence of a methoxy, nitro group or having a second chiral aminolactate in the *ortho-*position of iodine decreased the selectivity of the desired product **20a** slightly (entries 8–10 and 13–14, Table 1). On the other hand, the highest reactivity and selectivity was observed with catalyst **18b** that resulted in a quantitative formation of **20a** with 47% ee (entry 12, Table 1).

Further investigations were conducted to study the effect of the presence of an oxygen atom in place of a nitrogen atom in several catalysts, such as **19a**, **19b**, **19c**, and **7** (Scheme 4). These catalysts were synthesized as described above and were investigated using similar reaction conditions (entries 15–18, Table 1). The enantioselectivity of product **20a** was improved by utilizing the novel catalysts that contained a protected nitrogen rather than an oxygen atom. Chiral iodoarene **18a** was found to be the most efficient catalyst in the series that showed an improvement over previously reported catalysts. The rigidity of the sulfonamide in iodoarene **18a** resulted in an increased selectivity toward the formation of the product. The reactivities of catalysts **13a**, **13h**, **13i**, and **18c** were compared to their oxygen analogues through the obtained yield of product **20a**, and by cyclic voltammetry measurements (Figure S3, see SI). The catalysts **19a**, **19b**, **19c**, and **7** showed slightly lower oxidation potential values than **13a**, **13h**, **13i**, **18a**, and **18c**. The small difference of oxidation potentials is reflected in the reactivities for the formation of **20a** which resulted in almost similar yields (entries 1, 8–9, 11, 13, 15–18, Table 1).

We then attempted the optimization of the α-oxytosylation of propiophenone to improve the low selectivities obtained (Table 1). It was found that catalyst **18b** was highly reactive and gave the highest selectivity for **20a** and was therefore selected for the optimization study of the α-oxysulfonylation reaction. Many attempts have been performed for the α-oxytosylation reaction of ketones, and it was observed that short reaction times lead to the formation of the product in high yield. Interestingly, the optimal procedure was premixing aryl iodide catalyst **18b**, TsOH·H_2_O, and *m*CPBA for 1 h in a dry solvent under nitrogen atmosphere, followed by addition of propiophenone and stirring the resulting reaction mixture for 15 h. This approach was a successful method for forming the desired product in good yield and moderate enantioselectivity (entry 2, Table 2).

**Table 2:**
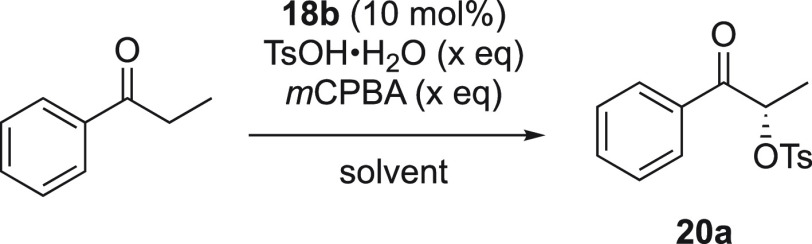
Screening of Reaction Parameters^a^

entry	solvent	time [h]	X [equiv]	temperature [°C]	yield **20a** [%]^b^	ee [%]^c^
1	MeCN	48	3	20	89	47
2	MeCN	15	3	20	80	44
3	HFIP	15	3	20	NP	–
4	acetone	15	3	20	Trace	–
5	toluene	15	3	20	23	33
6	CHCl_3_	15	3	20	38	37
7	DCE	15	3	20	46	36
8	CH_2_Cl_2_	15	3	20	53	36
9	TFE	15	3	20	60	26
10	THF	15	3	20	65	54
11	Et_2_O	15	3	20	76	37
12	EtOAc	15	3	20	99	58
13	EtOAc	15	2	20	91	58
14	EtOAc	15	3^d^	20	99	56
15	EtOAc	15	3	–20	99	58
16	EtOAc	15	3	–78^e^	96	57
17	EtOAc	18	3	0	99	60

a General method: **18b** (10 mol %), *m*CPBA (x equiv), and TsOH·H_2_O (x equiv), stirred for 1 h. Then, propiophenone (1 equiv) was added under N_2_ atmosphere and stirred for 18 h.

b ^1^H NMR yield determined using 1,3,5-trimethoxybenzene as internal standard.

c Determined by chiral HPLC.

d Anhydrous TsOH and recrystallized *m*CPBA used.

e Reaction time 5 h, then 10 h at 20 °C.

Furthermore, different solvents were investigated in the formation of **20a**. The results are presented in Table 2, and it was found that ethyl acetate was the best reaction medium. It afforded the product with 99% yield and 58% ee (entry 12, Table 2). The reaction proceeded with increased selectivity of 60% ee when conducted at 0 °C for 18 h (entry 17, Table 2). Lowering the reaction temperature to −20 °C or lower did not enhance the selectivity (entries 15 and 16, Table 2). Halogenated polar solvents did dissolve the reaction components well, but they reduced the reaction rate dramatically as most of the starting materials were recovered. Moreover, a decrease in the product enantioselectivity was observed (entries 6–9, Table 2). Because of the low nucleophilicity of 1,1,1,3,3,3-hexafluoro-2-propanol (HFIP) and its ability to stabilize the cation in the reaction medium,^31^ we assumed that HFIP could be an ideal solvent to improve the reactivity and selectivity of the reaction. However, no product formation was observed, and the starting material decomposed (entry 3, Table 2).

Following this, the focus was directed to study and make the applied method more environmentally friendly. Reducing the equivalents of *m*CPBA and TsOH·H_2_O reduced the yield and enantioselectivity slightly (entry 13, Table 2). On the other hand, using anhydrous TsOH and recrystallized *m*CPBA enhanced neither efficiency nor selectivity (entry 15, Table 2). Based on these results, we determined the optimized conditions with 10 mol % catalyst **18b** are *m*CPBA (3 equiv), TsOH·H_2_O (3 equiv) in ethyl acetate at 0 °C for 18 h.

A substrate scope for the α-oxysulfonylation of ketones was investigated. Propiophenone with electron-withdrawing substituents such as Cl, Br, or NO_2_ at the *meta*-position gave the desired products in good yields and selectivities. However, electron-withdrawing groups at the *para*-position reduced the reaction rate and selectivity slightly (entries 2–8, Table 3). Substituents with a NO_2_ or CF_3_ group were highly reactive at room temperature, producing the desired products **20d**–**e** and **20h** with good yield and enantiomeric excess (entries 4, 5, and 8, Table 3). On the other hand, electron-donating substituents at the *para*-position of ketones, such as Me and *t*Bu groups, formed the desired products **20k** and **20i** in good yields and moderate ee, whereas having a OMe group (**20j**) reduced the yield to 34% and the selectivity to 41% ee (entries 9–11, Table 3). Having a long alkyl chain at the α-position of the ketone produced the desired product in good yields without enhancement in selectivity (entries 12 and 13, Table 3). However, the yield was decreased to 50%, and the enantioselectivity was lost with a phenyl substituent at the α-position (entry 14, Table 3). Interestingly, substrate **20o** was found to be unreactive, even when increasing the reaction time to 48 h and performing the reaction at room temperature (entry 15, Table 3). Cyclic ketones such as indanone and tetralone were investigated and gave the desired products **20p** and **20q** in moderate yields and low enantioselectivities, while **20r** is produced as a racemic product in poor yield (entries 16–18, Table 3). A more sterically hindered ketone, where the phenyl group has been replaced with a 1-naphthyl group was also investigated, which afforded the oxytosylated product **20s** in 67% yield with 46% ee (entry 19, Table 3). Ketones containing aromatic heterocyclic five-membered ring substituents such as furan and thiophene were investigated as well. Remarkably, the thiophene substrate showed higher reactivity and selectivity compared to the furan derivative, and **20u** was formed in medium yield and enantioselectivity. In contrast, **20t** was obtained in lower yield and selectivity (entries 20 and 21, Table 3).

**Table 3:**
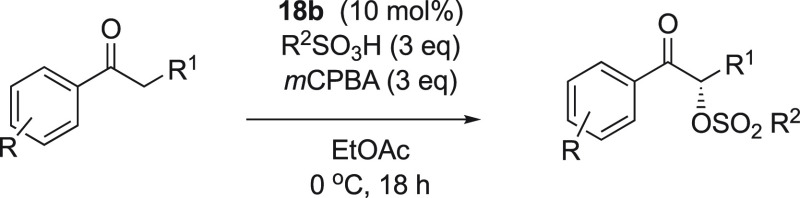

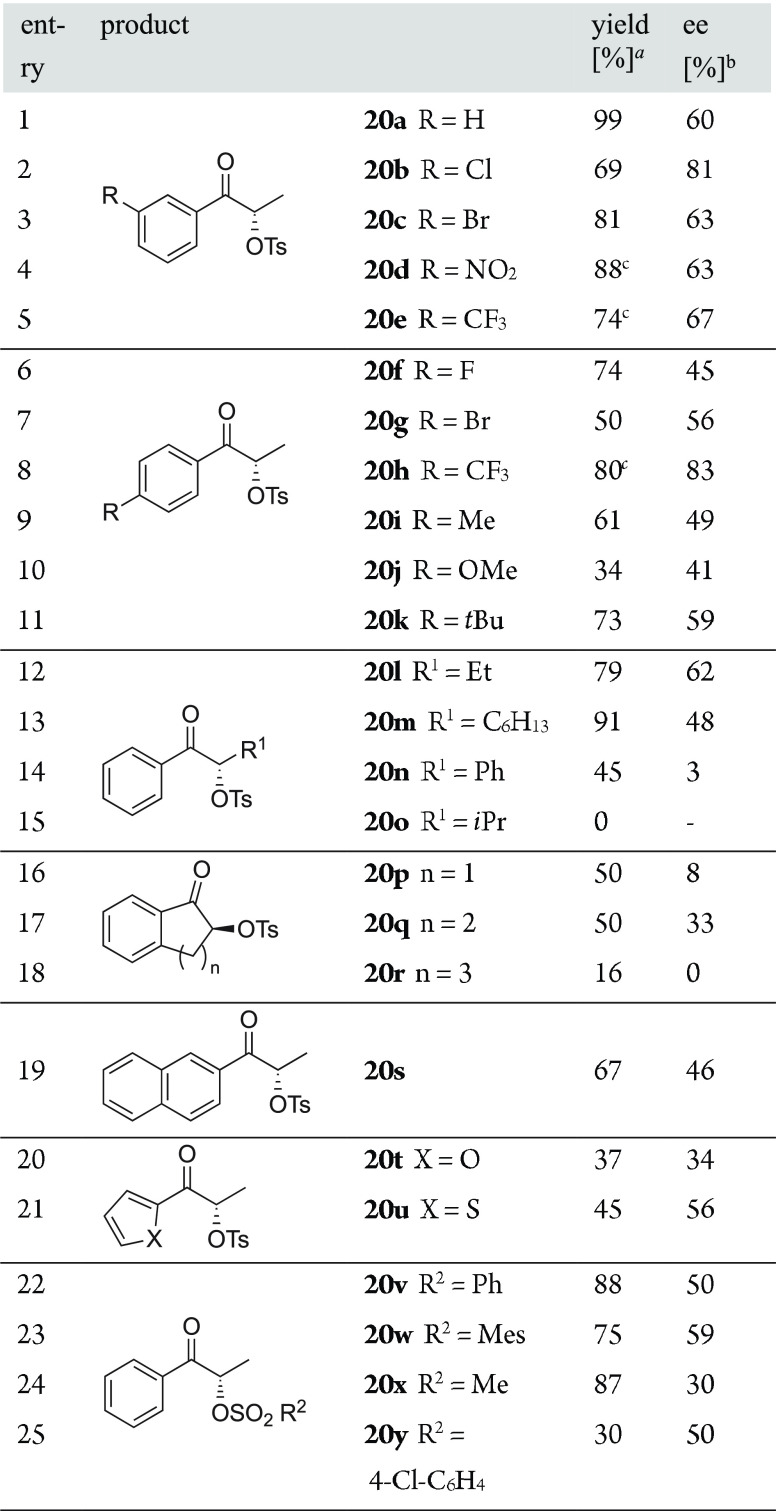
Substrate Scope of the Enantioselective α-Oxysulfonylation of Ketones

a Isolated yields.

b Determined by chiral HPLC.

c Reaction performed at room temperature.

Finally, a variety of sulfonic acids as nucleophiles were investigated. Benzenesulfonic acid was subjected to the reaction mixture, and the corresponding sulfoxylated product **20v** was obtained in high yield and medium selectivity (entry 22, Table 3). A sterically congested nucleophile such 2-mesitylenesulfonic acid dihydrate was also used, and the desired product **20w** was obtained in 75% with 59% ee (entry 23, Table 3). In contrast, methanesulfonic acid was utilized as a less sterically demanding nucleophile which decreased the selectivity of the corresponding product **20x** to 30% ee, while forming the product in 87% yield (entry 24, Table 3). 4-Chloro benzenesulfonic acid hydrate was also used and the corresponding product **20y** was obtained in low yield and with only moderate enantioselectivity (entry 25, Table 3).

The mechanism of the α-tosyloxylation of ketones has also been investigated with the help of quantum chemical calculations.^32^ The hypervalent iodine reagent **21** is generated in situ in the presence of *m*CPBA as an oxidant. The acid-catalyzed enolization reaction of ketone **23** enables two possible reaction pathways (Scheme 5). The enol form **22** can react with **21** via ligand exchange, producing the *O*-bonded intermediate **24**. The ketone **23** can also react with reagent **21** via ligand exchange to generate the *C*-bonded intermediate **24**. Subsequently, the intermediates react with an oxytosylate anion to form the chiral α-oxytosylated ketones **20**. According to mechanistic studies of Beaulieu and Legault,^32^ low selectivities for this transformation could originate from an equilibration between the two intermediates or from the distance between the chiral moieties and the newly generated stereocenter.

**Scheme 5:**
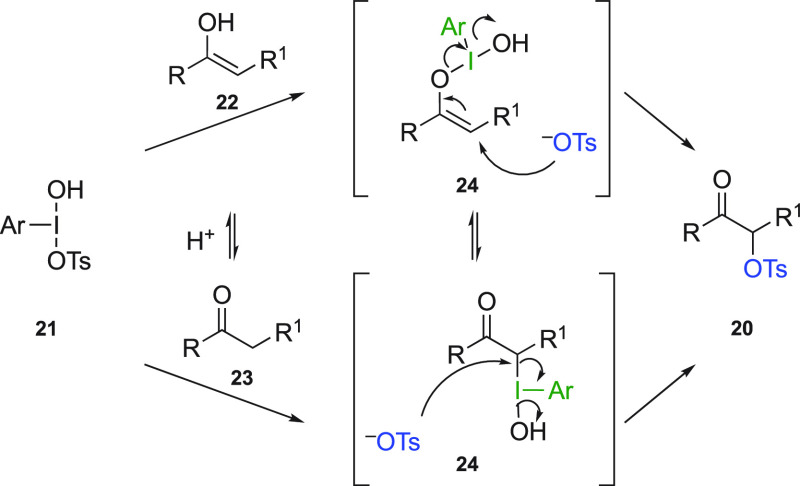
Mechanism of Oxysulfonylation with Iodine(III) Reagents

## Conclusion

Several chiral iodoarenes were successfully synthesized with good yields and assessed as catalysts in the α-oxysulfonylation of ketones. Comparisons of reactivities and selectivities between the described catalysts are made. Catalyst **18b** was found to be the most effective one in this series for achieving the α-oxysulfonylation in a short time and without the requirement to pre-form the enol of the starting materials. The products were obtained in good yields and enantiomeric excesses. A variety of ketones and sulfonyl nucleophiles have been used for screening and producing the targeted products with different yields and enantioselectivities.

## Experimental Section

The two different methods for the α-oxytosylation of ketones are shown below. For all other procedures referring to the synthesis of the chiral iodoarene catalysts, see the Supporting Information.

### Method A

In a 10 mL round-bottom flask, a chiral iodine catalyst (0.028 mmol), *m*CPBA (77% purity, 190 mg, 0.84 mmol), and a sulfonic acid (0.84 mmol) were dissolved in ethyl acetate (1 mL) and stirred for 1 h at room temperature, followed by the addition of the appropriate ketone (0.3 mmol). The reaction mixture was stirred at 0 °C for 18 h. Then, the mixture was washed with sat. aq. NaHCO_3_ (10 mL) solution and sat. aq. Na_2_S_2_O_3_ solution (10 mL) and extracted with ethyl acetate (3 × 20 mL). The combined organic layers were dried over MgSO_4_ (5 g), filtered, and concentrated under reduced pressure. The crude products were purified by flash chromatography on silica gel (petroleum ether/ethyl acetate: 9:1). The purification solvent was evaporated to afford the desired pure products.

### Method B

In a dried round-bottom flask, a chiral iodine catalyst (0.028 mmol), *m*CPBA (77% purity, 190 mg, 0.84 mmol), and a sulfonic acid (0.84 mmol) were dissolved in dry ethyl acetate (1 mL) and stirred for 1 h at room temperature, followed by the addition of the appropriate ketone (0.3 mmol). The reaction mixture was stirred at room temperature for 18 h. Then, the mixture was washed with sat. aq. NaHCO_3_ (10 mL) solution and sat. aq. Na_2_S_2_O_3_ solution (10 mL) and extracted with ethyl acetate (3 × 20 mL). The combined organic layers were dried over MgSO_4_ (5 g), filtered, and concentrated under reduced pressure. The crude products were purified by flash chromatography on silica gel (petroleum ether/ethyl acetate: 9:1). The purification solvent was evaporated to afford the desired pure products.

## Data Availability

The data underlying this study are available in the published article and its online Supporting Information.

## Supporting Information Available

Reaction optimization studies, synthetic procedures, and characterization data, spectroscopic data for new compounds, and copies of NMR spectra (PDF)
