# Supplementary material for: Synthesis of Chiral Iodoaniline-Lactate Based Catalysts for the α-Functionalization of Ketones
Source: ACS Org Inorg Au. 2023 May 9;3(4):209–16. doi: 10.1021/acsorginorgau.3c00012 (PMC10401694; doi:10.1021/acsorginorgau.3c00012)

Supporting Information

Synthesis of Chiral Iodoaniline-Lactate Based Catalysts for the α-Functionalization of Ketones

Rawiyah Alkahtani[a,b], and Thomas Wirth*[a]

[a] School of Chemistry, Cardiff University, Main Building, Park Place, CF10 3AT, Cardiff, United Kingdom.

[b] Chemistry Department, College of Science, Princess Nourah bint Abdulrahman University, 11671, Riyadh, Saudi Arabia.

*E-mail: wirth@cf.ac.uk

Table of contents

General methods	**S3**
General procedures	**S4**
Synthesis of novel C_1 −symmetric and asymmetric chiral iodoaniline catalysts	**S6**
Synthesis of novel C_1−asymmetric chiral iodoaniline catalysts bearing withdrawing group	**S18**
Synthesis of novel C_2−asymmetric chiral iodoaniline catalysts	**S21**
Synthesis of novel C_2−symmetric chiral iodoaniline catalysts	**S25**
Synthesis chiral iodophenol catalysts	**S28**
Substrate scope of the enantioselective α-oxysulfonylation of ketone derivatives	**S31**
Structural analysis of **13j** catalyst	**S52**
Cyclic voltammograms (CV)	**S55**
X-ray data	**S61**
References	**S75**
^{1}H and ^{13}C NMR spectra	**S77**

General methods:

All Reactions involving air and moisture sensitive reagents were carried out in flame dried glass wares under a dry nitrogen atmosphere using Schlenk technique or under or Argon using balloon. All reactions were stirred using magnetic stirring, and for the need of heating, over a hotplate with a temperature probe control and an adapted heating block. Lower temperatures reactions were achieved by using ice/water bath (0 °C), dry ice/acetone bath (– 78 °C) or using a chiller (0 to – 20 °C). Büchi rotavapors were used for solvent evaporations, and a high vacuum apparatus was used to further dry the products.
Reagents were purchased from Acros Organics, Alfa Aesar, Fisher Scientific, FluoroChem, Merck, Sigma Aldrich and TCI were used as received without purification.

Dry solvent of diethyl ether, tetrahydrofuran, toluene, and acetonitrile were collected from a solvent purification system (SPS) stored under a nitrogen atmosphere, which is from the company M BRAUN (MB SPS-800). Dry dichloromethane was freshly distilled from P_2O_5 under a or dried nitrogen atmosphere.

Thin layer chromatography (TLC) was performed to monitor the reactions using precoated aluminium sheets of Merck silica gel 60 F254 (0.20 m), and detection of compounds was performed under UV light (254 nm) or dipping into a solution of KMnO4 (1.5 g in 200 mL H_2O, 5 g $NaHCO_3$).

Flash column chromatography was performed using Merck silica gel 60 (40-63 µm) to purify products applying air pressure of about 0.2 bar or on a Biotage Isolera Four using Biotage cartridges SNAP Ultra 10 g, SNAP Ultra 25 g, SNAP Ultra 50 g and SNAP Ultra 100 g. The eluting solvents used for the purification are indicated in the text.

High resolution mass spectra (HRMS) were obtained from Cardiff University on a Waters GC-TOF spectrometer. Ions were generated using electron ionisation (EI), chemical ionisation (CI) atmospheric pressure chemical ionisation (APCI), or atmospheric-solid-analysis-probe (ASAP) techniques. All signals are reported with a mass-to-charge (m/z) ratio unit. Software: MassLynx Mass Spectrometry Software (Waters).

General procedures (GP):

GP1 for the sulfonation of 2-iodoaniline derivatives:

Method A:

To a stirred solution of 2-iodoaniline derivative (1 equiv.) and the appropriate sulfonyl chloride derivative (1.1 equiv.) in dry dichloromethane (solvent volume indicated in substrate detail), pyridine (1.5 equiv.) was added at 0 °C, under nitrogen gas. The reaction mixture was stirred at ambient temperature. The reaction was monitored by TLC until the reaction was complete, then the reaction was washed with HCl (1M, 3 × 30 mL). The combined acid wash was extracted with dichloromethane (4 × 25 mL). The combined organic layers were dried over $MgSO_4$, filtered, and concentrated under reduced pressure. The crude product was purified by flash column chromatography on silica gel (petroleum ether/ethyl acetate: 8:2).[1]

Method B:

To a stirred solution of 2-iodoaniline (1.0 equiv.) and sulfonyl chloride derivative (1.1 equiv.) in pyridine (20 ml) at 0 °C. The reaction mixture was stirred at room temperature and the reaction was monitored by TLC until it completed (from 2 to 6 h). Then, the reaction was quenched with 3M HCl until pH<7 (2 × 30 mL). The solution was extracted with Ethyl acetate or dichloromethan (3 × 30 mL), and the combined organic dried over anhydrous Mg_2SO_4, filtered and concentrated under reduced pressure. The crude mixture was purified by column chromatography on silica gel (petroleum ether/ethyl acetate: 8:2) to afford the pure product.

GP2 for the Mitsunobu reaction:

Method A:

Protected 2-iodoaniline derivatives (1 equiv.), methyl or ethyl (*S*)-lactate (1.5 equiv.) and triphenylphosphine PPh_3 (1.5 equiv.) were dissolved in dry toluene (solvent volume indicated in substrate detailed) under nitrogen atmosphere. The reaction mixture was cooled to 0 °C and diisopropyl azodicarboxylate (DIAD) (1.5 equiv.) was added dropwise. After 1 h at 0 °C, the mixture was warmed up to 50 ºC temperature and stirred for 16 h. The solvent was removed under reduced pressure and Et_2O was added. Triphenylphosphine oxide, which precipitated, was removed by filtration and the filtrate was concentrated under vacuum. The crude mixture was purified by flash column chromatography with (petroleum ether/ethyl acetate: 8:2).[1]

Method B:

In dried round bottom flask, the protected 2-iodoaniline derivative (1 equiv.), lactate derivatives (2.5 equiv.) and PPh_3 (2.5 equiv.) were dissolved in dry toluene (solvent volume indicated in

substrate detailed) under nitrogen. The reaction mixture was cooled to 0 °C and DIAD (2.5 equiv.) was added dropwise. After 1 h at 0 °C, the mixture was warmed up to 50 °C temperature and stirred for 16 h. The solvent was removed under reduced pressure and Et_2O was added. Triphenylphosphine oxide, which precipitated, was removed by filtration and the filtrate was concentrated under vacuum. The crude mixture was purified by flash column chromatography with of (petroleum ether/ethyl acetate: 8:2).[1]

Method C:

In dried round bottom flask, 2-iodophenol derivatives (1 equiv.), lactate derivatives (1.5 equiv.) and PPh_3 (1.5 equiv.) were dissolved in dry tetrahydrofuran (THF) (solvent volume indicated in substrate detailed) under nitrogen atmosphere. The reaction mixture was cooled to 0 °C and DIAD (1.5 equiv.) was added dropwise. After 1 h at 0 °C, the mixture was warmed up to room temperature and stirred for 16 h. The solvent was removed under reduced pressure and Et_2O was added. Triphenylphosphine oxide, which precipitated, was removed by filtration and the filtrate was concentrated under vacuum. The crude mixture was purified by flash column chromatography with (petroleum ether/ethyl acetate: 8:2).[2]

Method D:

In dried round bottom flask, the protected 2-iodobenzene-1,3-diol (1 equiv.), lactate derivatives (2.5 equiv.) and PPh_3 (2.5 equiv.) were dissolved in dry THF under nitrogen. The reaction mixture was cooled to 0 °C and DIAD (2.5 equiv.) was added dropwise. After 1 h at 0 °C, the mixture was warmed up to room temperature for 16 h. The solvent was removed under reduced pressure and Et_2O was added. Triphenylphosphine oxide, which precipitated, was removed by filtration and the filtrate was concentrated under vacuum. The crude mixture was purified by flash column chromatography with of (petroleum ether/ethyl acetate: 8:2).[3]

GP3 for the α-oxysulfonylation of ketones:

Method A:

In 10 ml round bottom flask, chiral iodine catalyst (0.1 equiv.), *m*CPBA (3 equiv.), and a sulfonic acid (3 equiv.) were dissolved in ethyl acetate (1 mL) and stirred for one hour at room temperature followed by the addition of the appropriate ketone (1 equiv.). The reaction mixture was stirred at 0 °C for 18 h. Then, the mixture was washed with sat. aq. $NaHCO_3$ (10 mL) solution and sat. aq. $Na_2S_2O_3$ solution (10 mL) and extracted with ethyl acetate (3 × 20 mL). The combined organic layers were dried over $MgSO_4$ (5 g), filtered, and concentrated under reduced pressure. The crude products were purified by flash chromatography on silica gel (petroleum ether/ethyl acetate: 9:1). The purification solvent was evaporated to afford the desired pure products.

Method B:

In a dried round bottom flask, chiral iodine catalyst (0.1 equiv.), *m*CPBA (3 equiv.), and a sulfonic acid (3 equiv.) were dissolved in dry ethyl acetate (1 mL) and stirred for one hour followed by the

addition of the appropriate ketone (1 equiv.). The reaction mixture was stirred at room temperature for 18 h. Then, the mixture was washed with sat. aq. $NaHCO_3$ (10 mL) solution and sat. aq. $Na_2S_2O_3$ solution (10 mL) and extracted with ethyl acetate (3 × 20 mL). The combined organic layers were dried over $MgSO_4$ (5 g), filtered, and concentrated under reduced pressure. The crude products were purified by flash chromatography on silica gel (petroleum ether/ethyl acetate: 9:1). The purification solvent was evaporated to afford the desired pure products.

Scheme 1: Synthesis of novel C_1–symmetric and asymmetric chiral iodoaniline catalysts:

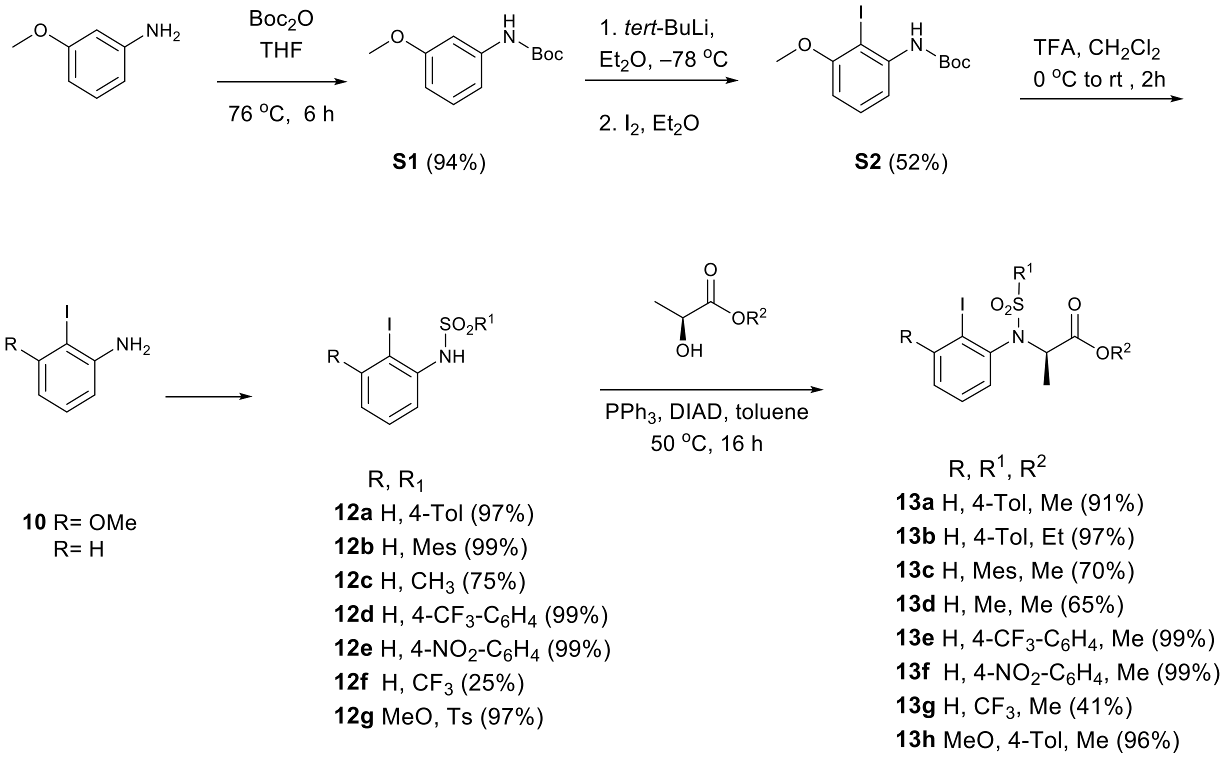

tert-Butyl 3-methoxyphenylcarbamate (S1)

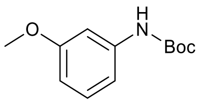

According to the literature,[4,5] a solution of 3-methoxyaniline (10 g, 81.2 mmol) and di-*tert*-butyl dicarbonate (17.7 g, 81.2 mmol) in dry THF (60 mL) was refluxed for 5 h. The solvent was evaporated under reduced pressure. To residue was partitioned between 0.1 N aqueous HCl (50 mL) and Et_2O (50 mL). The aqueous layer was extracted with Et_2O (2 × 50 mL). The Et_2O extract was washed with a saturated aqueous NaCl solution (50 mL), dried over $MgSO_4$ and evaporated under reduced pressure. The residue was purified by column chromatography (petroleum ether/ethyl acetate: 10:1) to give a colorless solid (18 g, 98% yield).

Mp 57–58 °C.

^{1}H NMR (500 MHz, $CDCl_3$) δ = 7.17 (t, *J* = 8 Hz, 1H), 7.10 (s, 1H), 6.84 (dd, *J* = 8.0, 1 Hz, 1H), 6.59 (ddd, *J* = 8, 2.5, 0.5 Hz, 1H), 6.52 (s, 1H), 3.79 (s, 3H), 1.52 (s, 9H) ppm.

^{13}C{^{1}H} NMR (126 MHz, $CDCl_3$) δ = 160.4, 152.8, 139.8, 129.8, 110.8, 109.0, 104.2, 80.6, 55.4, 28.5 ppm.

LRMS (ESP-TOF): m/z $[M+H]^+$ calcd for $C_{12}H_{18}NO_3$ 224.1207; found 224.0022.

Data are in agreement with the literature.[2]

tert-Butyl 2-iodo-3-methoxyphenylcarbamate (S2)

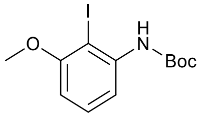

According to the literature,[4,5] *tert*-butyllithium (138mL of 1.3M, 179.4mmol) was added to a solution of **S1** (10.0 g, 44.8 mmol) in dry diethyl ether at -78 °C slowly. The reaction mixture was warmed to −20 °C and stirred for 3 h. The solution was again cooled to –78 °C and quenched by the dropwise addition of a solution of iodine (17.2 g in 100 mL of dry ether, 67.2 mmol), ensuring the reaction temperature did not rise above −65 °C. After 1 h, the reaction mixture was warmed to room temperature slowly overnight. After addition of a saturated aqueous $Na_2S_2O_3$ solution (100 mL), the mixture was extracted with ether (3 × 80 mL). The ether extract was washed with a saturated aqueous NaCl solution (150 mL), dried over Na_2SO_4, and evaporated under reduced pressure. The residue was purified by silica gel column chromatography (petroleum ether/ethyl acetate: 30:1) to give a light-yellow solid (10.5 g, 67% yield).

Mp 73–75 °C.

^{1}H NMR (400 MHz, $CDCl_3$) δ = 7.74-7.72 (m, 1H), 7.24 (t, *J* = 8.0 Hz, 1H), 7.05 (br, 1H), 6.52-6.50 (m, 1H), 3.85 (s, 3H), 1.53 (s, 9H) ppm.

^{13}C{^{1}H} NMR (100 MHz, $CDCl_3$) δ = 158.2, 152.4, 140.2, 129.5, 112.6, 105.5, 81.1, 80.8, 56.4, 28.2 ppm.

HRMS (ESI- TOF): m/z $[M+H]^+$ calcd for $C_{12}H_{17}INO_3$ 349.0173; found 349.0129.

Data are in agreement with the literature.[2]

2-Iodo-3-methoxybenzenamine (10)

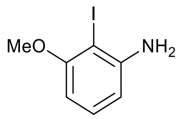

According to the literature,[5] trifluoroacetic acid (20 mL) was added dropwise to a solution of *tert*-Butyl 2-iodo-3-methoxyphenylcarbamate **S2** (5 g, 14.3 mmol) in dichloromethane (60 mL) at 0 °C. The reaction mixture was warmed to room temperature and stirred for 2 h in dark. The solution was concentrated by evaporation and diluted by ethyl acetate (150 mL). A sat. aq. $NaHCO_3$ solution was added slowly at 0 °C until pH 7–8. The aqueous layer was extracted with ethyl acetate (2 × 80 mL). The organic extract was washed with a saturated aqueous NaCl solution (150 mL), dried over $MgSO_4$ and evaporated under reduced pressure. The residue was purified by silica gel column chromatography (petroleum ether/ethyl acetate: 10:1) to give a title product as colorless viscous (3.2 g, 90% yield). This product is air and light sensitive, so it should be stored under nitrogen at low temperature –5 °C. Due to problems of stability, the obtained compound was immediately used in the following step.

^{1}H NMR (500 MHz, $CDCl_3$) δ = 7.07 (t, *J* = 8.0 Hz, 1H), 6.41 (dd, *J* = 8.0, 1.5 Hz, 1H), 6.22 (dd, *J* = 8.0, 1.5 Hz, 1H), 4.22 (sbr, 2H), 3.86 (s, 3H) ppm.

^{13}C{^{1}H} NMR (126 MHz, $CDCl_3$) δ = 159.0, 148.5, 129.7, 107.9, 100.6, 76.1, 56.5 ppm.

HRMS (EC-CI) m/z $[M]^+$ calcd for C_7H_8NOI 248.9645; found 248.9646.

Data are in agreement with the literature.[2]

N-(2-Iodo-3-methoxyphenyl)-4-methylbenzenesulfonamide (12g)

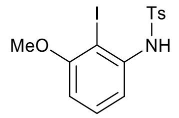

Following GP1 method A, 2-iodo-3-methoxybenzenamine **10** (0.8 g, 3.21 mmol, 1 equiv.) was reacted with *p*-toluenesulfonyl chloride (0.67 g, 3.53 mmol, 1.1 equiv.) in dry dichloromethane (20 mL). Then, pyridine (0.39 mL, 4.81 mmol, 1.5 equiv.) was added. The crude product was purified to afford title product as colorless solid (1.25 g, 97% yield).

Mp 100–105 °C.

^{1}H NMR (500 MHz, $CDCl_3$) δ = 7.67 (d, *J* = 8.5 Hz, 2H), 7.30 (dd, *J* = 8, 1.5 Hz, 1H), 7.21 (m, 3H), 7.05 (s, 1H), 6.54 (dd, *J* = 8, 1.5 Hz, 1H), 3.82 (s, 3H), 2.36 (s, 3H) ppm.

^{13}C{^{1}H} NMR (126 MHz, $CDCl_3$) δ = 158.7, 144.3, 139.1, 136.1, 130.0, 129.8, 127.6, 114.0, 107.3, 84.2, 56.7, 21.7 ppm.

IR (neat): $\tilde{\nu}$ = 3255, 1465, 1433, 1323, 1265, 1161, 1072, 520 cm^{-1}.

HRMS (ESI- TOF) m/z: [M+H] $^{+}$ calcd for $C_{14}H_{15}NO_3IS$ 403.9817; found 403.9815.

Methyl *N*-(2-iodo-3-methoxyphenyl)-*N*-tosyl-*D*-alaninate (13h)

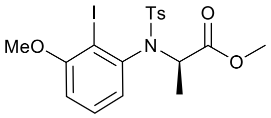

Following GP2 method A, *N*-(2-iodo-3-methoxyphenyl)-4-methylbenzenesulfonamide **12g** (0.98 g, 2.43 mmol, 1 equiv.), methyl (*S*)-(–)-lactate (0.38 g, 3.65 mmol, 1.5 equiv.), triphenylphosphine (0.95 g, 3.65 mmol, 1.5 equiv.) and diisopropyl azodicarboxylate (0.72 mL, 3.65 mmol, 1.5equiv.) were reacted in toluene (30 mL) to give product **13h** as colorless solid (1.3 g, 96% yield).

[α]$_D$^{20} = +28.57 (c = 0.28, $CHCl_3$).

Mp 150–153 °C.

^{1}H NMR (500 MHz, $CDCl_3$) δ = 7.62 and 7.57 (d and d, *J* = 8.5 and 8.5 Hz, 2H), 7.30 – 7.26 (m, 1H), 7.26 – 7.22 and 7.01 (m, and dd, *J* = 8.0, 1.5 Hz, 3H), 6.84 and 6.81 (dd and dd, *J* = 7.5, 2.0 and 8.5, 1.5 Hz, 1H), 4.97 and 4.41 (q and , *J* = 7.5 and 7.5 Hz, 1H), 3.90 and 3.88 (s and s, 3H), 3.77 and 3.58 (s and s, 3H), 2.42 (s, 3H), 1.55 and 1.28 (d and d, *J* = 7.0 and 7.5 Hz, 3H) ppm.

^{13}C{^{1}H} NMR (126 MHz, $CDCl_3$) δ = 172.9, 171.6, 159.7, 159.7, 143.8, 143.7, 142.9, 141.0, 137.4, 129.6, 129.3, 129.2, 129.0, 128.6, 128.4, 126.1, 125.7, 111.5, 111.1, 99.6, 96.5, 60.2, 57.7, 56.9, 52.6, 52.2, 21.8, 17.5, 16.7 ppm.

IR (neat): $\tilde{\nu}$ = 3284, 1924, 1581, 1471, 1327, 1157, 1085, 908, 709, 659, 542 cm^{-1}.

HRMS (ESI-TOF) m/z: [M+H]$^+$ calcd for $C_{18}H_{21}NO_5IS$ 490.0185; found 490.0180.

***N*-(2-Iodophenyl)-4-methylbenzenesulfonamide (12a)**

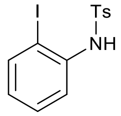

Following GP1 method A, iodoaniline (2.55 g, 11.64 mmol, 1 equiv.), *p*-toluenesulfonyl chloride (2.44 g, 12.80 mmol, 1.1 equiv.), dry dichloromethane (50 mL) and pyridine (1.41 mL, 17.4637 mmol, 1.5 equiv.). The crude product was purified to afford product **12a** as a colorless solid (4.3 g, 97% yield).

Mp 89–91 °C.

^{1}H NMR (500 MHz, $CDCl_3$) δ = 7.68 – 7.61 (m, 4H), 7.33 – 7.28 (m, 1H), 7.21 (d, *J* = 8.0 Hz, 2H), 6.84 – 6.81 (m, 1H), 6.79 (s, 1H), 2.38 (s, 3H) ppm.

^{13}C{^{1}H} NMR (126 MHz, $CDCl_3$) δ = 144.4, 139.4, 137.7, 136.0, 129.8, 129.7, 127.6, 127.0, 122.6, 92.4, 21.7 ppm.

IR (neat) $\tilde{\nu}$ = 3246, 2925, 1597, 1467, 1381, 1335, 1167, 1091, 1017, 891, 808 cm^{-1}.

HRMS (CI) m/z: $[M]^+$ calcd for $C_{13}H_{12}NO_2IS$ 372.9627; found 372.9631.

Data are in agreement with the literature.[6]

***N*-(2-Iodophenyl)-2,4,6-trimethylbenzenesulfonamide (12b)**

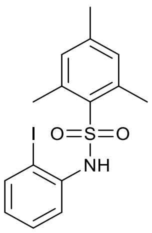

Following GP1 method A, iodoaniline (0.50 g, 2.28 mmol, 1 equiv.), $MesSO_2Cl$ (0.55 g, 2.51 mmol, 1.1 equiv.), dry dichloromethane (20 mL) and pyridine (0.28 mL, 3.42 mmol, 1.5 equiv.) were reacted. The reaction completed in 6 h at room temperature. The crude product was purified to afford product **12b** as a colorless solid (0.73 g, 99% yield).

Mp 115–118 °C.

^{1}H NMR (500 MHz, $CDCl_3$) δ = 7.69 (dd, *J* = 7.5, 1 Hz, 1H), 7.30 (dd, *J* = 8, 1.5 Hz, 1H), 7.24 – 7.18 (m, 1H), 6.95 (s, 1H), 6.93 (s, 2H), 6.77 (m, 1H), 2.63 (s, 6H), 2.27 (s, 3H) ppm.

^{13}C{^{1}H} NMR (126 MHz, $CDCl_3$) δ = 143.1, 139.6, 139.5, 138.0, 133.7, 132.4, 129.5, 126.1, 120.5, 90.8, 23.5, 21.1 ppm.

IR (neat) $\tilde{\nu}$ = 3277, 2926, 1601, 1461, 1380, 1324, 1163, 1016, 906, 756 cm^{-1}.

HRMS (ESI-TOF) m/z: $[M+H]^+$calcd for $C_{15}H_{17}NO_2IS$ 402.0025; found 402.0024.

Data are in agreement with the literature.[6]

***N*-(2-Iodophenyl)methanesulfonamide (12c)**

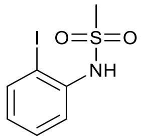

Following GP1 method A, 2-iodoaniline (0.50 g, 2.28 mmol, 1 equiv.), MsCl (0.29 g, 2.51 mmol, 1.1 equiv.), dry dichloromethane (20 mL) and pyridine (0.28 mL, 3.42 mmol, 1.5 equiv.) were reacted. The reaction completed in 4 h at room temperature. The crude product was purified to afford product **12c** as colorless solid (0.5 g 75% yield).

Mp 95–97 °C.

^{1}H NMR (300 MHz, $CDCl_3$) δ = 7.83 (dd, *J* = 7.8, 1.2 Hz, 1H), 7.65 (dd, *J* = 8.4, 1.5 Hz, 1H), 7.38 (t, *J* = 7.3 Hz, 1H), 6.94 (t, *J* = 7.5 Hz, 1H), 6.63 (br, 1H), 3.01 (s, 3H) ppm.

^{13}C{^{1}H} NMR (126 MHz, $CDCl_3$) δ = 139.6, 137.8, 130.1, 127.4, 122.5, 92.2, 40.3 ppm.

LRMS (APCI) m/z: $[M-H]^-$ calcd for $C_7H_7INO_2S$ 295.90; found 295.81.

Data are in agreement with the literatures.[6,7]

N-(2-Iodophenyl)-4-(trifluoromethyl)benzenesulfonamide (12d)

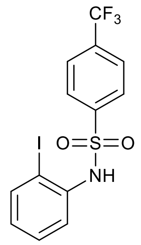

Following GP1 method A, 2-iodoaniline (0.50 g, 2.28 mmol, 1 equiv.), 4-(trifluoromethyl)benzenesulfonyl chloride (0.62 g, 2.51 mmol, 1.1 equiv.), dry dichloromethane (20 mL) and pyridine (0.28 mL, 3.42 mmol, 1.5 equiv.). The reaction completed in 3 h at room temperature. The crude product was purified to afford product **12d** as yellow solid (0.96 g 99% yield).

Mp 122–124 °C.

^{1}H NMR (400 MHz, $CDCl_3$) δ = 7.85 (d, *J* = 8.8 Hz, 2H), 7.68– 7.65 (m, 4H), 7.35 (t, *J* = 8.0 Hz, 1H), 6.91 – 6.87 (m, 2H) ppm.

^{13}C{^{1}H} NMR (126 MHz, $CDCl_3$) δ = 142.5, 139.4, 136.9, 135.0 (q, *J* = 130 Hz), 129.8, 128.0, 127.8, 126.3 (q, *J* =15 Hz), 123.7, 93.2 ppm.

HRMS (ESI-TOF) m/z: $[M-H]^-$ calcd for $C_{13}H_8NO_2F_3IS$ 425.9273; found 425.9265.

N-(2-Iodophenyl)-4-nitrobenzenesulfonamide (12e)

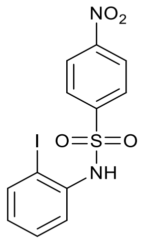

Following GP1 method A, 2-iodoaniline (0.50 g, 2.28 mmol, 1 equiv.), 4-nitrobenzenesulfonyl chloride (0.6 g, 2.51 mmol, 1.1 equiv.), dry dichloromethane (20 mL) and pyridine (0.28 mL, 3.42 mmol, 1.5 equiv.) were reacted. The reaction completed in 3 h at room temperature. The crude product was purified to afford product **12e** as yellow solid (0.92 g 99% yield).

Mp 122–124 °C.

^{1}H NMR (300 MHz, $CDCl_3$) δ = 8.27 (d, *J* = 9 Hz, 2H), 7.89 (d, *J* = 9 Hz, 2H), 7.74- 7.765 (m, 2H), 7.42 – 7.35 (m, 1H), 6.93 (td, *J* = 7.8, 1.5 Hz, 1H), 6.83 (s, 1H) ppm.

^{13}C{^{1}H} NMR (126 MHz, $CDCl_3$) δ = 150.6, 144.6, 139.5, 136.5, 130.0, 128.9, 128.3, 124.4, 124.2, 93.6 ppm.

HRMS (ESI-TOF)m/z: $[M+Na]^+$calc. for $C_{12}H_9O_4N_2ISNa$ 426.9220; found 426.9224.

Data are in agreement with the literature.[8]

1,1,1-Trifluoro-*N*-(2-iodophenyl)methanesulfonamide (12f)

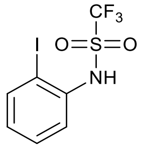

2-Iodoaniline (0.5 g, 2.28 mmol, 1 equiv.) was dissolved in 10 mL dry CH_2Cl_2 under N_2, followed by addition of triethyl amine (0.46 g, 4.57 mmol, 2 equiv.). The solution was cooled to 0 °C and trifluoromethanesulfonic acid anhydride (0.77 g, 2.74 mmol, 1.2 equiv.) added dropwise. The reaction mixture was stirred at 0 °C for 2 h, allowed to warm to rt. After completion of the reaction 18 h, water (20 mL) was added, the aqueous phase extracted with dichloromethane (3 × 20 mL), the combined organic layers washed with brine (20 mL), dried over $MgSO_4$ and concentrated in vacuo. The crude purified by flash chromatography (petroleum ether/ethyl acetate: 2:1) and provided the product as yellow crystals (0.21 g, 25%).

Mp 53–55 °C.

^{1}H NMR (400 MHz, $CDCl_3$): δ = 7.84 (dd, *J* = 8.2, 1.4 Hz, 1H), 7.59 (dd, *J* = 8.3, 1.3 Hz, 1H), 7.38 (ddd, *J* = 8.5, 7.0, 1.4 Hz, 1H), 7.03-6.98 (m, 1H) ppm.

^{13}C{^{1}H} NMR (100 MHz, $CDCl_3$): δ = 139.8, 135.8, 130.0, 128.9, 123.7, 120.0 (q, *J* = 322 Hz), 93.1 ppm.

HRMS (ASAP-TOF) m/z: [M^+] calcd for $C_7H_5NO_2F_3IS$ 350.9038; found 350.9032.

Data are in agreement with the literature.[9]

Methyl *N*-(2-iodophenyl)-*N*-tosyl-*L*-alaninate (13a)

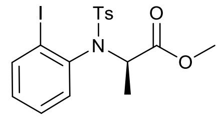

Following GP2 method A, N-(2-iodophenyl)-4-methylbenzenesulfonamide (2.18 g, 7.53 mmol, 1 equiv.), PPh_3 (2.96 g, 11.29 mmol, 1.5 equiv.), (*S*)-lactic acid methyl ester (1.18 g, 1.08 mL, 11.29 mmol, 1.5 equiv.), and DIAD (2.28 g, 2.22 mL, 11.29 mmol, 1.5 equiv.) were reacted in toluene (60 mL) to afford the product **13a** as colorless solid (3.3 g 91% yield). Conformational isomer ratio is 3:1.

$[\alpha]_D^{20}$ = +13.0 (c = 1.38, $CHCl_3$).

Mp 80–82 ˚C.

^{1}H NMR (500 MHz, $CDCl_3$) δ = 7.89 and 7.84 (dd, *J* = 8, 1.5 and 8, 1.5 Hz, 1H), 7.59 – 7.50 (m, 3 H), 7.32 – 7.20 (m, 3H), 7.06 – 7.0 and 7.01 – 6.98 (m and m, 1H), 4.95 and 4.41 (q and q, *J* = 7.5 and 7.5 Hz, 1H), 3.75 and 3.55 (s and s, 3H), 2.39 (s, 3H), 1.49 and 1.26 (d, *J* = 7.5 and 7.5 Hz, 3H) ppm.

^{13}C{^{1}H} NMR (126 MHz, $CDCl_3$) δ = 172.5, 171.2, 143.8, 143.6, 141.0, 140.3, 139.2, 136.9, 133.6, 133.1, 130.4, 130.2, 129.4, 129.1, 128.8, 128.5, 128.2, 128.1, 106.4, 103.3, 59.8, 57.4, 52.4, 52.1, 21.5, 17.3, 16.5 ppm.

IR (neat): ν = 2980, 1745, 1355, 1167, 714 cm^{-1}.

HRMS (ESI-TOF) m/z: $[M+Na]^+$ calcd for $C_{17}H_{18}NO_4ISNa$ 481.9899; found 481.9913.

Ethyl *N*-(2-iodophenyl)-*N*-tosyl-*D*-alaninate (13b)

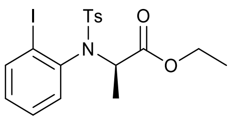

Following GP2 method A, *N*-(2-iodophenyl)-4-methylbenzenesulfonamide (1 g, 2.68 mmol, 1 equiv.), PPh_3 (1.05 g, 4.02 mmol, 1.5 equiv.), (*S*)-lactic acid ethyl ester (0.47 g, 0.45 mL, 4.02 mmol, 1.5 equiv), and DIAD (0.81 g, 0.8 mL, 4.02 mmol, 1.5 equiv) were reacted in toluene (30 mL) to afford product **13b** as colouless solid (1.2 g, 97% yield). Conformational isomer ratio is 3:1.

$[\alpha]_D^{20}$ = + 6.16 (c = 2.6, $CHCl_3$).

Mp 83–85 °C.

^{1}H NMR (500 MHz, $CDCl_3$) δ = 7.89 (dd and dd, *J* = 26.3, 8, 1.5 and 7.5, 1 Hz, 1H), 7.64 and 7.43 (dd and dd, *J* = 8.0, 2 and 8.0, 2 Hz, 1H), 7.57 (dd and dd, *J* = 8.5 and 8.5 Hz, 2H), 7.35-7.30 (m, 1H), 7.27 – 7.22 (m, 2H), 7.09 – 7.01 (m, 1H), 4.96 and 4.36 (q and q, *J* = 7 and 7 Hz, 1H), 4.28 – 4.20 and 4.10 – 3.98 (m, 2H), 2.42 (s, 3H), 1.57 and 1.29 (d and d, *J* = 7 and 7.5 Hz, 1H), 1.31 and 1.22 (t and t, *J* = 7.25 and 7.25 Hz, 3H) ppm.

^{13}C{^{1}H} NMR (126 MHz, $CDCl_3$) δ = 172.3, 171.1, 143.8, 143.7, 141.5, 140.5, 140.5, 139.4, 137.3, 137.3, 133.9, 133.6, 130.5, 130.2, 129.5, 129.2, 128.9, 128.6, 128.5, 128.3, 106.6, 103.2, 77.3, 61.7, 61.3, 60.5, 57.8, 21.7, 17.6, 16.8, 14.2, 14.1 ppm.

IR (neat) $\tilde{\nu}$ = 2986, 1732, 1464, 1342, 1157, 1018, 713, 657, 545 cm^{-1}.

HRMS (ESI-TOF) m/z: $[M+H]^+$ calcd for $C_{18}H_{21}NO_4IS$ 474.0236; found 474.0236.

Methyl *N*-(2-iodophenyl)-*N*-(mesitylsulfonyl)-*D*-alaninate (13c)

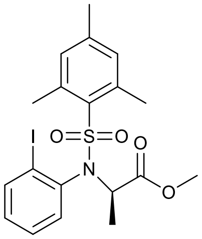

Following GP2 method A, *N*-(2-iodophenyl)-2,4,6-trimethylbenzenesulfonamide (2.1 g, 5.23 mmol, 1 equiv.), PPh_3 (2.1 g, 7.85 mmol, 1.5 equiv.), (*S*)-lactic acid methyl ester (0.55 g, 0.50 ml, 7.85 mmol, 1.5 equiv.), and DIAD (1.6 g, 1.55 mL, 7.85 mmol, 1.5 equiv.) were reacted in toluene (60 mL) to afford product **13c** as colouless solid (2 g, 70% yield). Conformational isomer ratio is 5:1.

$[\alpha]_D^{20}$ = 10.35 (c= 0.56, $CHCl_3$).

Mp 162–163 °C.

^{1}**H NMR** (500 MHz, $CDCl_3$) δ = 8.22 and 7.66 (dd and dd, *J* = 8, 1.5 and 8, 1.5 Hz, 1H), 7.76 and 7.74 (dd, *J* = 8, 1.5 and 8, 1.5 Hz, 1H), 7.45 - 7 .41 and 7.40 – 7.37 (m and m, 1H), 7.05 – 7.01 (m, 1H), 6.85 and 6.84 (sbr and sbr, 2H), 5.16 and 5.11 (q and q, *J* = 7 and 7.5 Hz, 1H), 3.80 and 3.72 (s and s, 3H), 2.30 and 2.26 (sbr and s, 9 H), 1.50 and 1.31 (d and d, *J* = 7 and 7.5 Hz, 3H) ppm.

^{13}**C{^{1}H} NMR** (126 MHz, $CDCl_3$) δ = 173.5, 170.6, 143.0, 142.6, 141.2, 140.8, 140.6, 140.2, 138.7, 138.6, 136.6, 135.4, 134.7, 133.4, 132.1, 130.5, 130.3, 129.2, 128.4, 104.9, 103.9, 58.9, 57.8, 52.5, 52.5, 29.8, 23.8, 23.5, 21.1, 21.0, 18.1, 16.3 ppm.

IR (neat) $\tilde{\nu}$ = 2920, 2850, 1747, 1463, 1336, 1103, 640, 536 cm^{-1}.

HRMS (ASAP-TOF) m/z: $[M+H]^+$ calcd for $C_{19}H_{23}NO_4SI$ 488.0392; found 488.0401.

Methyl *N*-(2-iodophenyl)-*N*-(methylsulfonyl)-*D*-alaninate (13d)

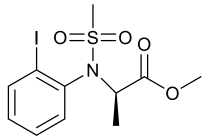

Following GP2 method A, *N*-(2-iodophenyl)methanesulfonamide (0.5 g, 1.68 mmol, 1 equiv.), PPh_3 (0.66 g, 2.52 mmol, 1.5 equiv.), (*S*)-lactic acid methyl ester (0.26 g, 0.24 ml, 2.52 mmol, 1.5 equiv.), and DIAD (0.58 g, 2.52 mmol, 1.5 equiv.) were reacted in toluene (20 mL) to afford the product **13d** as colorless solid (2 g, 65% yield). Conformational isomer ratio is 4:1.

$[\alpha]_D^{20}$ = 20 (c = 2.2, $CHCl_3$).

Mp 105–107 °C.

^{1}**H NMR** (500 MHz, $CDCl_3$) δ= 7.98 and 7.93 (dd and dd, *J* = 8.0, 1.5 and 8.0, 1.5 Hz, 1H), 7.85 and 7.70 (dd and dd, *J* = 8.0, 1.5 and 8.0, 1.5 Hz, 1H), 7.44- 7.38 (m, 1H), 7.12- 7.05 (m , 1H), 4.98 and 4.37 (q and q, *J* = 7 and 7 Hz, 1H), 3.80 and 3.79 (s and s, 3H), 3.22 and 3.12 (s and s, 3H), 1.73 and 1.32 (d and d, *J* = 7.5 and 7 Hz, 3H) ppm.

^{13}**C{^{1}H} NMR** (126 MHz, $CDCl_3$) δ= 173.6, 171.8, 141.9, 141.0, 140.4, 139.3, 134.3, 133.0, 130.8, 130.6, 129.4, 129.2, 106.3, 101.8, 61.0, 58.5, 52.7, 52.6, 43.8, 40.7, 17.9, 16.4 ppm.

IR (neat) $\tilde{\nu}$ = 3315, 1728, 1462, 1334, 1155, 765, 543 cm^{-1}.

HRMS (ESI-TOF) m/z: $[M+Na]^+$ calcd for $C_{11}H_{14}NO_4ISNa$ 405.9586; found 405.9592.

Methyl *N*-(2-iodophenyl)-*N*-((4-(trifluoromethyl)phenyl)sulfonyl)-*D*-alaninate (13e)

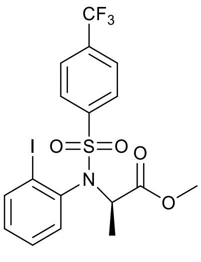

Following GP2 method A, *N*-(2-iodophenyl)-4(trifluoromethyl) benzene sulfonamide (0.89 g, 2.08 mmol, 1 equiv.), PPh_3 (0.82 g, 3.13 mmol, 1.5 equiv.), (*S*)-lactic acid methyl ester (0.32 g, 0.30 ml, 3.13 mmol, 1.5 equiv.), and DIAD (0.72 g, 3.13 mmol, 1.5 equiv.) were reacted in toluene (30 mL) to afford the product **13e** as colorless sticky viscous liquid (1.00 g, 99% yield). Conformational isomer ratio is 4:1.

$[\alpha]_D^{20}$ = + 1.82 (c = 2.2, $CHCl_3$).

^{1}H NMR (500 MHz, $CDCl_3$) δ= 7.94 and 7.86 (dd and dd, *J* = 8, 1.5 and 8, 1.5 Hz, 1H), 7.82 and 7.78 (d and d, *J* = 8.5 and 8.5 Hz, 2H), 7.73 (t, *J* = 7.5 Hz, 2H), 7.6- 7.54 (m, 1H), 7.42-7.36 and 7.35- 7.32 (m and m, 1H), 7.13- 7. 04 (m, 1 H), 5.01 and 4.37 (q and q, *J* = 7.5 and 7 Hz, 1H), 3.80 and 3.61 (s and s, 3H), 1.70 and 1.31 (d and d, *J* = 7.5 and 7.5 Hz, 3H) ppm.

^{13}C{^{1}H} NMR (126 MHz, $CDCl_3$) δ= 172.5, 171.3, 143.9, 143.4, 141.4, 140.9, 140.6, 138.9, 134.7, 134.4, 134.1, 133.6, 130.9, 130.6, 129.2, 129.0, 129.0, 128.7, 126.1 q (J = 15 Hz) 125.7 q (J = 14.5 Hz), 124.5, 122.3, 106.0, 101.9, 61.4, 58.1, 52.8, 52.4, 17.7, 16.6 ppm.

IR (neat) $\tilde{\nu}$ = 1743, 1321, 1157, 1107, 1060, 717, 597 cm^{-1}.

HRMS (ESP- TOF) m/z: $[M+H]^+$ calcd for $C_{17}H_{16}NO_4F_3IS$ 513.9797; found 513.9799.

Methyl *N*-(2-iodophenyl)-*N*-((4-nitrophenyl)sulfonyl)-*D*-alaninate (13f)

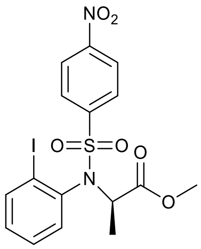

Following GP2 method A, *N*-(2-iodophenyl)-4-nitrobenzenesulfonamide (0.89 g, 2.21 mmol, 1 equiv.), PPh_3 (0.90 g, 3.30 mmol, 1.5 equiv.), (*S*)-lactic acid methyl ester (0.34 g, 0.31 ml, 3.30 mmol, 1.5 equiv.), and DIAD (0.67 g, 0.65 ml, 3.30 mmol, 1.5 equiv.) were reacted in toluene (30 mL) to afford the product **13f** as colorless solid (1.02 g, 99% yield). Conformational isomer ratio is 4:1.

[α]$_D^{20}$ = 5 (c = 1.2, $CHCl_3$).

Mp 118–120 °C

^{1}H NMR (500 MHz, $CDCl_3$) δ= 8.34 and 8.32 (d and d, *J* = 9 and 9 Hz, 2H), 7.98 and 7.87 (dd and dd, *J* = 8, 1.5 and 8, 1.5 Hz, 1H), 7.89 and 7.83 (d and d, *J* = 9.0 Hz, 2H), 7.69 and 7.58 (dd and dd, *J* = 8.0, 1.5 and 8.0, 1.5 Hz, 1H), 7.46 – 7.42 and 7.40 – 7.36 (m and m, 1H), 7.16 – 7.12 and 7.11 – 7.09 (m, 1H), 5.06 and 4.38 (q and q, *J* = 7.5 and 7.4 Hz, 1H), 3.83 and 3.69 (s and s, 3H), 1.79 and 1.34 (d and d, *J* = 7.4 and 7.5 Hz, 3H) ppm.

^{13}C{^{1}H} NMR (126 MHz, $CDCl_3$) δ= 172.5, 171.3, 150.2, 146.2, 145.5, 141.4, 141.0, 140.6, 138.6, 134.4, 133.5, 131.1, 130.8, 129.8, 129.5, 129.3, 129.2, 124.2, 123.8, 105.9, 101.4, 61.9, 58.4, 52.8, 52.6, 17.8, 16.6 ppm.

IR (neat) $\tilde{\nu}$ = 3329, 1747, 1521, 1346, 1211, 740, 607, 565 cm^{-1}.

HRMS (ESP-TOF) m/z: $[M+Na]^+$ calcd for $C_{16}H_{15}N_2O_6SINa$ 512.9593; found 512.9592.

Methyl *N*-(2-iodophenyl)-*N*-((trifluoromethyl)sulfonyl)-*D*-alaninate (13g)

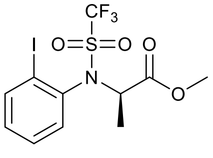

Following GP2 method A, 1,1,1-trifluoro-*N*-(2-iodophenyl)methanesulfonamide (0.40 g, 1.14 mmol, 1 equiv.), PPh_3 (0.44 g, 1.71 mmol, 1.5 equiv.), (*S*)-lactic acid methyl ester (0.17 g, 0.16 ml, 1.71 mmol, 1.5 equiv.), and DIAD (0.36 g, 0.32 ml, 1.71 mmol, 1.5 equiv.) were reacted in toluene (20 mL) to afford a desired product as yellow solid (0.20 g, 41% yield). Conformational isomer ratio is 3:1.

[α]$_D^{20}$ = 5 (c = 2.0, $CHCl_3$).

Mp 71–74 °C.

^{1}H NMR (500 MHz, MeOD) δ = 8.05 – 7.91 and 7.79 – 7.71 (m and m, 2H), 7.51 – 7.42 (m, 1H), 7.23 – 7.13 (m, 1H), 5.07 and 4.60 (q and q, *J* = 7.5 Hz, 1H), 3.79 and 3.77 (s and s, 3H), 1.84 and 1.43 (d and d, *J* = 7.5 Hz, 3H) ppm.

^{13}C{^{1}H} NMR (126 MHz, MeOD) δ = 172.6, 171.4, 142.1, 142.0, 139.9, 139.2, 134.4, 134.1, 132.5, 132.0, 130.4, 129.8, 125.2, 122.6, 120.1, 106.0, 60.8, 53.3, 53.1, 17.9, 16.9 ppm.

IR (neat) $\tilde{\nu}$ = 1741, 1624, 1388, 1190, 1116, 1016, 771, 574 cm^{-1}.

HRMS (ESP-TOF) m/z: $[M+H]^+$ calcd for $C_{11}H_{12}NO_4F_3IS$ 437.9484; found 437.9490.

Scheme 2: Synthesis of novel C_1 −asymmetric chiral iodoaniline catalysts bearing withdrawing group.

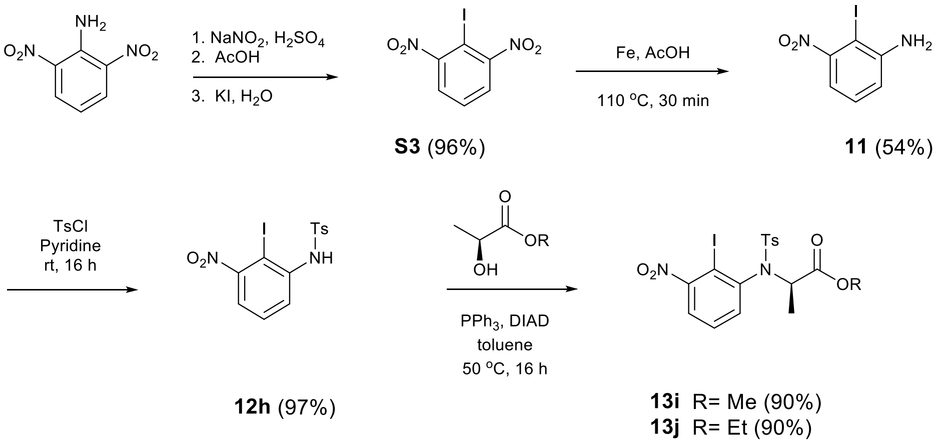

2-Iodo-1,3-dinitrobenzene (S3)

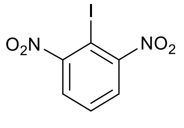

Based on a literature procedure,[10] sodium nitrite (1.66 g, 24.03 mmol, 1.1 equiv.) was added portion-wise to sulfuric acid (conc., 20 mL), and the resulting suspension was warmed to 70 ºC until the solids were completely dissolved. The solution was allowed to cool to below 40 ºC, and 2,6- dinitroaniline (4 g, 21.85 mmol, 1 equiv.) in acetic acid (150 mL) was added dropwise at such a rate as to keep the temperature of the reaction mixture below 40 ºC. The solution was stirred for a further 30 min, then poured into a stirred 70 ºC solution of potassium iodide (4 g, 24.03 mmol, 1.1 equiv.) in water (100 mL). After 15 min the reaction mixture was poured into water (500 mL), and the crude product was collected by filtration. The crude solid was taken up in dichloromethane (250 mL) and washed with sodium thiosulfate solution (2 × 100 mL). The organic layer was dried, filtered and concentrated by vacuo to give the title compound as a pale brown solid (6.15 g, 96% yield).

^{1}H NMR (500 MHz, $CDCl_3$) δ = 7.83 (d, J = 8.0 Hz, 2H), 7.66 (t, J = 8.0 Hz, 1H) ppm.

^{13}C{^{1}H} NMR (126 MHz, $CDCl_3$) δ = 156.3, 130.5, 127.0, 80.3 ppm.

HRMS (CI): m/z $[M+H]^+$ calcd for $C_6H_4N_2O_4I$ 294.9210; found 294.9211.

Data are in agreement with the literature.[10]

2-Iodo-3-nitroaniline (11)

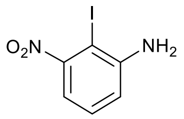

Based on a literature procedure,[10] iron powder (0.55 g, 10 mmol, 3.0 equiv.) was added cautiously in portions to a vigorously stirred 110 ˚C solution of 2-iodo-1,3-dinitrobenzene (1.0 g, 3.41 mmol, 1.0 equiv.) in glacial acetic acid (13 mL). After complete addition of the iron, the suspension was heated to reflux. After 30 min the reaction mixture was poured directly into cold water (100 mL). The aqueous layer was extracted with dichloromethane (3 × 50 mL), and the combined organic layers dried over anhydrous magnesium sulfate, filtered, and concentrated in vacuo. The residue was purified by flash column chromatography (petroleum ether/ethyl acetate: 8:2). The title compound obtained as a golden yellow solid (2.42 g, 54% yield).

Mp 84–85 °C.

^{1}H NMR (300 MHz, $CDCl_3$) δ = 7.21 (t, *J* = 7.8 Hz, 1H), 7.05 (dd, *J* = 8.1, 1.5 Hz, 1H), 6.88 (dd, *J* = 8.1, 1.5 Hz, 1H), 4.56 (s, 2H) ppm.

^{13}C{^{1}H} NMR (126 MHz, $CDCl_3$) δ = 155.3, 149.2, 129.6, 117.1, 114.4, 75.2 ppm.

HRMS (CI) m/z $[M]^+$ calcd for $C_6H_5O_2N_2I$ 263.9390; found 263.9394.

Data are in agreement with the literature.[10]

N-(2-Iodo-3-nitrophenyl)-4-methylbenzenesulfonamide (12h)

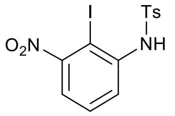

Following GP1 method B, iodoaniline (1.36 g, 5.15 mmol, 1 equiv.), TsCl (1.1 g, 5.66 mmol, 1.1 equiv.), and dry pyridine (10 mL). The reaction completed in 6 h at room temperature. The crude product was purified to afford product **12h** as yellow solid (1.9 g, 97% yield).

Mp 133–135 °C.

^{1}H NMR (500 MHz, $CDCl_3$) δ = 7.88 (dd, *J* = 7.5, 1.5 Hz, 1H), 7.66 (d, *J* = 8.5 Hz, 2H), 7.49 – 7.39 (m, 2H), 7.26 (d, *J* = 2 Hz, 1H), 7.25 (d, *J* = 0.5 Hz, 1H), 7.21 (s, 1H), 2.40 (s, 3H) ppm.

^{13}C{^{1}H} NMR (126 MHz, $CDCl_3$) δ = 145.1, 140.3, 135.6, 130.1, 130.1, 127.6, 124.7, 121.4, 85.1, 21.8 ppm.

IR (neat) $\tilde{\nu}$ = 3315, 1530, 1458, 1261, 1157, 812, 657, 561 cm^{-1}.

HRMS (ESN-TOF): $[M-H]^-$ calcd for $C_{13}H_{10}N_2O_4IS^-$ 416.9406; found 416.9391.

Methyl *N*-(2-iodo-3-nitrophenyl)-*N*-tosyl-*L*-alaninate (13i)

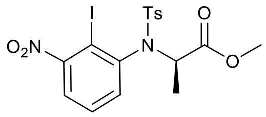

Following GP2 method A, *N*-(2-iodo-3-nitrophenyl)-4-methylbenzenesulfonamide (0.25 g, 0.60 mmol, 1 equiv.), PPh_3 (0.24 g, 0.90 mmol, 1.5 equiv.), (*S*)-lactic acid methyl ester (0.1 g, 0.90 mmol, 1.5 equiv.), and DIAD (0.19 g, 0.90 mmol, 1.5 equiv.) were reacted in toluene (20 mL) to afford product **13i** as yellow viscous liquid (0.28 g, 90% yield).

$[\alpha]_D^{20}$ = +15.0 (c = 2.0, $CHCl_3$).

^{1}H NMR (500 MHz, $CDCl_3$) δ = 7.97 and 7.76 (dd and dd, *J* = 8.0, 1.5 and 8.0, 1.5 Hz, 1H), 7.66 – 7.44 (m, 4H), 7.28 (d, *J* = 8.0 Hz, 2H), 4.99 and 4.36 (q and q, *J* = 7.5 and 7.5 Hz, 1H), 3.78 and 3.6 3 (s and s, 3H), 2.44 (s, 3H), 1.66 and 1.29 (d and d, *J* = 7 and 7 Hz, 3H) ppm.

^{13}C{^{1}H} NMR (126 MHz, $CDCl_3$) δ = 172.8, 171.1, 156.4, 156.2, 144.6, 144.5, 144.4, 142.4, 137.1, 136.9, 136.7, 136.6, 129.8, 129.6, 129.5, 129.2, 128.4, 128.2, 124.9, 124.6, 100.5, 96.9, 60.9, 57.6, 52.8, 52.5, 21.8, 21.7, 17.5, 16.7 ppm.

HRMS (ESP-TOF) m/z: $[M+H]^+$ calcd for $C_{17}H_{18}N_2O_6IS$ 504.9930; found 504.9939.

Ethyl *N*-(2-iodo-3-nitrophenyl)-*N*-tosyl-*D*-alaninate (13j)

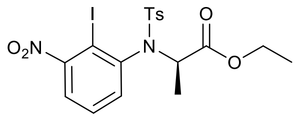

Following GP2 method A, *N*-(2-iodo-3-nitrophenyl)-4-methylbenzenesulfonamide (1.68 g, 4.02 mmol, 1 equiv.), PPh_3 (1.6 g, 6.03 mmol, 1.5 equiv.), (*S*)-lactic acid ethyl ester (0.71 g, 0.70 mL, 6.03 mmol, 1.5 equiv.), and DIAD (1.22 g, 1.20 mL, 6.03 mmol, 1.5 equiv.) were reacted in toluene (50 mL) to afford product **13j** as yellow solid (1.9 g, 90% yield).

$[\alpha]_D^{20}$ = + 17.9 (c = 2.9, $CHCl_3$).

Mp 132–134 °C.

^{1}H NMR (300 MHz, $CDCl_3$) δ= 7.99 and 7.78 (dd and dd, *J* = 8.0, 1.5 and 8.0, 1.5 Hz, 1H), 7.67 – 7.42 (m, 4 H), 7.30 – 7.24 (m, 2 H), 4.96 and 4.31 (q and q, *J* = 7.2 and 7.5 Hz, 1H), 4.26 – 4.17 and 4.11 – 3.99 (m, and m, 2H), 2.43 (s, 3H), 1.67 and 1.34 – 1.20 (d and m, 6 H) ppm.

$^{13}C\{^1H\}$ **NMR** (75 MHz, $CDCl_3$) δ= 172.3, 170.7, 156.4, 156.3, 144.4, 142.4, 137.1, 136.9, 136.8, 136.6, 129.8, 129.5, 129.4, 129.2, 128.4, 128.2, 124.9, 124.5, 100.6, 99.3, 62.0, 61.6, 61.1, 57.7, 21.7, 17.5, 16.7, 14.1, 14.1 ppm.

SS ^{13}C NMR (101 MHz) δ = 172.3, 156.9, 145.3, 144.4, 138.3, 133.1, 130.7, 129.9, 129.5, 126.9, 122.7, 62.5, 57.3, 23.0, 19.0, 13.2 ppm.

IR (neat) $\tilde{\nu}$ = 1699, 1190, 1170, 927, 808, 705, 653, 551 cm^{-1}.

HRMS (APCI-TOF) m/z: $[M+H]^+$ calcd for $C_{18}H_{20}N_2O_6IS$ 519.0087; found 519.0090.

Scheme 3: Synthesis of novel *C*$_2$–asymmetric chiral iodoaniline catalysts

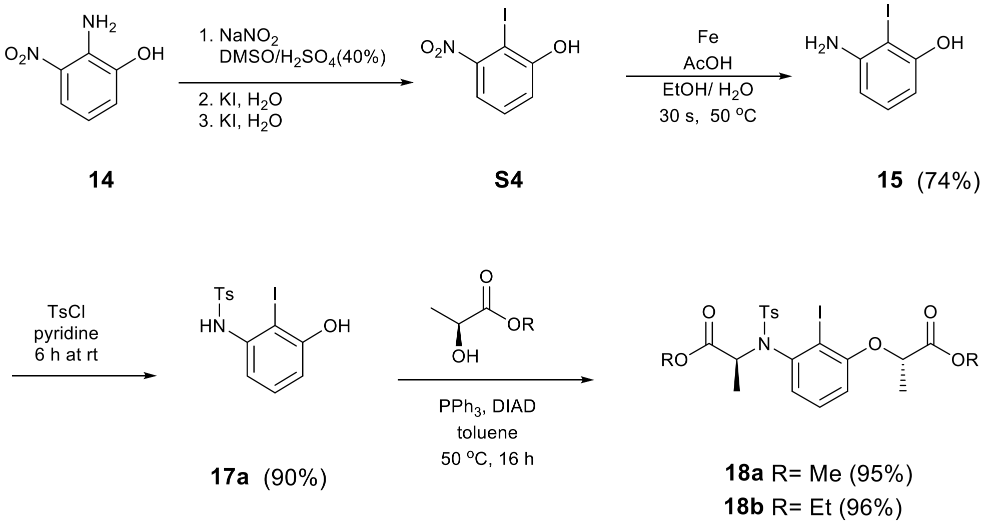

2-Iodo-3-nitrophenol (S4)

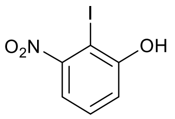

Following the modified procedure of literature,[11] under nitrogen atmosphere 2-amino-3-nitrophenol (4 g, 26 mmol, 1 equiv.) was added to a mixture of DMSO (40 mL) and sulfuric acid (50 mL of a 50% aqueous solution) and the resulting mixture was stirred at 50 °C for 2 h then cooled to 0 °C. The mixture was treated, over 5 min, with a solution of sodium nitrite (5.5 g, 77.86 mmol, 3 equiv.) in water (17 mL). The reaction was stirred at 0 °C for 1 h and then treated, in one portion, with potassium iodide (13 g, 77.86 mmol, 3 equiv.) in water (17 mL). After a further 1 h the reaction mixture was warmed to room temperature, and another portion of potassium iodide (13 g, 77.86 mmol, 3 equiv.) in water (17 mL) was added and the mixture was stirred at room temperature for one hour. The solution was then extracted with ethylacetate (3 × 200 mL). The

separated organic phase was washed with saturated aqueous sodium thiosulfate (2 × 100 mL), water (2 × 100 mL) then brine (100 mL). the crude was dried over ($MgSO_4$) and concentrated *in vacuo*. Purification with flash column chromatography (petroleum ether: ethyl acetate: 5:1) afforded 2-iodo-3-nitrophenol as an orange solid (6.23 g, 92% yield).

Mp 122–123 °C.

^{1}H NMR (500 MHz, $CDCl_3$) δ = 7.43 (dd, *J* = 8.0, 1.5 Hz, 1H), 7.37 (t, *J* = 8.5 Hz, 1H), 7.23 (dd, *J* = 8, 1.5 Hz, 1H), 5.97 (s, 1H) ppm.

^{13}C{^{1}H} NMR (126 MHz, $CDCl_3$) δ 156.9, 153.6, 130.4, 119.0, 118.0, 79.7 ppm.

HRMS (CI) m/z $[M+H]^+$ calcd for $C_6H_5NO_3I$ 265.9308; found 265.9309.

Data are in agreement with the literature.[12]

3-Amino-2-iodophenol (15)

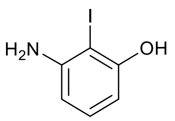

Following the literature procedure,[13] to a solution of 2-iodo-3-nitro phenol (2.00 g, 7.55 mmol, 1.00 eq.) in EtOH/H_2O (1/1, 60 mL) and conc. acetic acid (6 mL), iron powder (1.69 g, 30.3 mmol, 4.02 equiv.) was added. The reaction mixture was heated to 50 °C until the suspension was colored dark brown. After 30 s, the mixture was cooled in an ice bath and added with 28% NH_4OH solution (30 mL). The mixture was filtered and washed with EtOAc (4 × 100 mL). The filtrate was washed with sat. NaCl solution (200 mL), the phases were separated, and the aqueous phase was extracted with EtOAc (3 × 30 mL). The organic phases were combined, dried over $MgSO_4$ and the solvent was removed in vacuum. The crude was purified using column chromatography (petroleum ether/ethyl acetate: 8:2) afforded the desired product as a colorless solid (1.25 g, 74%).

Mp 95–98 °C.

^{1}H NMR (400 MHz, $CDCl_3$) δ = 7.01 (t, *J* = 8.0 Hz, 1H), 6.40 - 6.32 (m, 2H), 5.26 (br s, 1H), 4.12 (br s, 2H) ppm.

^{13}C{^{1}H} NMR (100 MHz, $CDCl_3$) δ = 155.5, 147.9, 130.1, 107.1, 104.7, 76.8 ppm.

HRMS (ASAP-TOF) m/z: $[M+H]^+$ calcd for C_6H_7NOI 235.9572; found 235.9567.

Data are in agreement with the literature.[12]

***N*-(3-Hydroxy-2-iodophenyl)-4-methylbenzenesulfonamide (17a)**

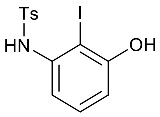

Following GP1 method A, 3-amino-2-iodophenol (1.66 g, 7.07 mmol, 1 equiv.), *p*-toluenesulfonyl chloride (2.44 g, 7.42 mmol, 1.1 equiv.), dry dichloromethane (30 mL) and pyridine (2.3 mL, 28.28 mmol, 4 equiv.). The crude product was purified to afford product as colorless solid (2.1 g, 90% yield).

Mp 127–129 °C.

^{1}H NMR (500 MHz, $CDCl_3$) δ = 7.65 (d, *J* = 8.3 Hz, 2H), 7.23 – 7.11 (m, 4H), 6.83 (s, 1H), 6.70 (dd, *J* = 7.9, 1.6 Hz, 1H), 5.76 (s, 1H), 2.37 (s, 3H) ppm.

^{13}C{^{1}H} NMR (126 MHz, $CDCl_3$) δ = 155.7, 144.4, 138.3, 135.9, 130.2, 129.7, 127.6, 114.5, 112.0, 83.9, 21.2 ppm.

IR (neat) $\tilde{\nu}$ = 3001, 1700, 1357, 1170, 929, 810, 653, 551 cm^{-1}.

HRMS (ESI) m/z: $[M\text{-}H]^-$ calcd for $C_{13}H_{11}NO_3IS$ 387.9504; found 387.9504.

Methyl *N*-(2-iodo-3-(((*R*)-1-methoxy-1-oxopropan-2-yl)oxy)phenyl)-*N*-tosyl-*D*-alaninate (18a)

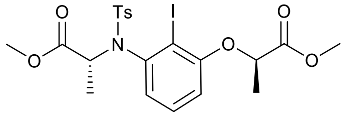

Following GP2 method B, **17a** (0.48 g, 1.23 mmol, 1 equiv.), PPh_3 (0.81 g, 3.08 mmol, 2.5 equiv.), (*S*)-lactic acid methyl ester (0.32 g, 0.83 mL, 0.29 mmol, 2.5 equiv.), DIAD (0.62 g, 0.61 mL, 3.08 mmol, 2.5 equiv.) were reacted in toluene (30 mL) to afford product **18a** as colorless viscus liquid (0.66 g, 95% yield).

$[\alpha]_D^{20}$ = + 5.8 (c = 2.4, $CHCl_3$).

^{1}H NMR (300 MHz, $CDCl_3$) δ= 7.58 and 7.53 (d and d, *J* = 8.4 and 8.4 Hz, 2H), 7.25 – 7.16 and 7.01 (m and d, *J* = 7.0 Hz, 4H), 6.70 and 6.65 (d and d, *J* = 8.1 and 8.1 Hz, 1H), 4.93 and 4.35 (q and q, *J* = 7.5 and 7.2 Hz, 1H), 4.72 (q, *J* = 7.0 Hz, 1H), 3.76 and 3.74 (s and s, 3H), 3.73 and 3.56 (s and s, 3H), 2.39 (s, 3H), 1.68 and 1.55 (d and d, *J* = 7.0 and 7.2 Hz, 3H), 1.25 (d, *J* = 7.5 Hz, 3H) ppm.

$^{13}C\{^{1}H\}$ NMR (126 MHz, $CDCl_3$) δ = 172.9, 172.0, 172.0, 171.5, 158.2, 158.2, 143.8, 143.7, 141.3, 137.5, 137.3, 129.5, 129.2, 129.1, 128.8, 128.5, 128.4, 127.3, 126.6, 114.1, 113.4, 101.1, 97.7, 75.0, 74.7, 60.5, 57.7, 52.6, 52.2, 22.0, 21.7, 18.7, 18.7, 17.5, 16.6 ppm.

IR (neat) $\tilde{\nu}$ = 2989, 1749, 1456, 1209, 1136, 711 cm^{-1}.

HRMS (ESI-TOF) m/z: $[M+H]^+$ calcd for $C_{21}H_{25}NO_7IS$ 562.0396; found 562.0399.

Ethyl (*R*)-2-(3-((*N*-((*R*)-1-ethoxy-1-oxopropan-2-yl)-4-methylphenyl)sulfonamido)-2-iodophenoxy)propanoate (18b)

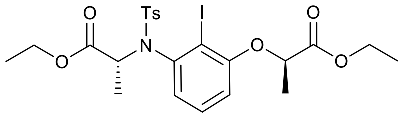

Following GP2 method B, **17a** (2.3 g, 5.91 mmol, 1 equiv.), PPh_3 (3.8 g, 14.77 mmol, 2.5 equiv.), (*S*)-lactic acid ethyl ester (1.75 g, 1.70 mL, 14.77 mmol, 2.5 equiv.), DIAD (3 g, 2.9 mL, 14.77 mmol, 2.5 equiv.) and toluene (100 mL) to afford product **18b** as colorless viscus liquid (2.6 g, 96% yield).

$[\alpha]_D^{20}$ = + 7.1 (c = 3.4, $CHCl_3$).

^{1}H NMR (500 MHz, $CDCl_3$) δ= 7.62 and 7.56 (d and d, *J* = 8.0 and 8.0 Hz, 2H), 7.33 and 7.11 (dd and dd, *J* = 8.0, 1.5 and 8.0, 1.0 Hz, 1H), 7.25 – 7.18 (m, 3H), 6.73 and 6.67 (dd and dd, *J* = 8.5, 1.5 and 8.0, 1.0 Hz, 1H), 4.97- 4.69 and 4.33- 3.99 (m and m , 6 H), 2.42 and 2.41 (s and s, 3H), 1.71 – 1.59 (m, 3 H), 1.32– 1.19 (m, 9 H) ppm.

$^{13}C\{^{1}H\}$ NMR (126 MHz, $CDCl_3$) δ= 172.5, 171.5, 171.5, 171.1, 158.2, 158.1, 143.7, 143.6, 141.3, 137.6, 137.4, 129.5, 129.2, 129.0, 128.8, 128.5, 128.4, 128.2, 127.4, 126.7, 114.0, 113.2, 101.1, 97.5, 75.0, 74.6, 61.7, 61.6, 61.5, 61.3, 60.9, 57.8, 21.7, 18.7, 18.6, 17.6, 16.7, 14.2, 14.1, 14.1 ppm.

IR (neat) $\tilde{\nu}$ = 1735, 1456, 1348, 1163, 1085, 711, 657 cm^{-1}.

HRMS (ASAP-TOF) m/z: $[M+H]^+$ calcd for $C_{23}H_{29}NO_7IS$ 590.0709; found 590.0714.

Scheme 4: Synthesis of novel C_2–symmetric chiral iodoaniline catalysts

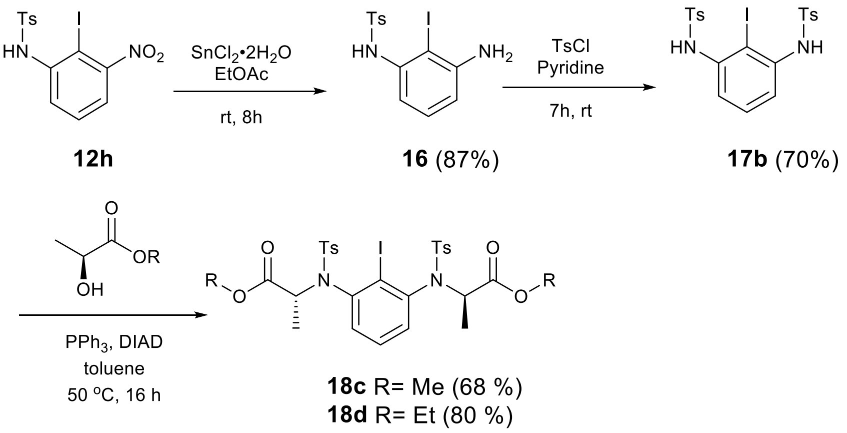

N-(3-Amino-2-iodophenyl)-4-methylbenzenesulfonamide (16)

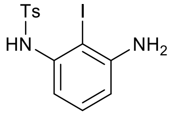

Following the reported procedure,[14] to a 25 mL two neck round bottom flask *N*-(2-iodo-3-nitrophenyl)-4-methylbenzenesulfonamide (2.4 g, 5.74 mmol, 1.0 equiv.) was added, dry ethyl acetate (30 mL), and $SnCl_2$•$2H_2O$ (6.47 g, 28.70 mmol, 5 equiv.). This solution was placed under a nitrogen atmosphere and stirred for 8 h. After this time the white suspension was quenched with sat aq. $NaHCO_3$, diluted with ethyl acetate (40 mL) and filtered over celite or layer of silica and cotton. The celite or silica was washed with ethyl acetate and the filtrate was washed with more $NaHCO_3$ (3 × 50 mL), brine, then dried and concentrated. The crude product was purified by flash column chromatography (petroleum ether/ethyl acetate: 8:2) to produce pure product as light brown solid (1.83 g, 87% yield).

Mp 140–142 °C.

^{1}H NMR (300 MHz, $CDCl_3$) δ = 7.66 (d, *J* = 8.4 Hz, 1H), 7.21 (d, *J* = 8.0 Hz, 1H), 7.09 – 6.99 (m, 2H), 6.84 (s, 1H), 6.42 (dd, *J* = 6.4, 1.8 Hz, 1H), 4.08 (s, 1H), 2.37 (s, 1H) ppm.

^{13}C{^{1}H} NMR (75 MHz, $CDCl_3$) δ 147.8, 144.2, 138.0, 136.1, 135.5, 129.7, 127.6, 111.8, 111.5, 82.6, 21.7 ppm.

IR (neat) $\tilde{\nu}$ =3203, 1527, 1346, 1153, 854, 734, 611, 549 cm^{-1}.

HRMS (ESI-TOF) m/z: [M+Na]$^+$ calcd for $C_{13}H_{13}N_2O_2SINa$ 410.9640; found 410.9644.

***N,N'*-(2-Iodo-1,3-phenylene)bis(4-methylbenzenesulfonamide) (17b)**

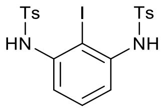

Following GP1 method A, *N*-(3-amino-2-iodophenyl)-4-methylbenzenesulfonamide (0.93 g, 3.23 mmol, 1 equiv.) reacted with *p*-toluenesulfonyl chloride (0.68 g, 3.55 mmol, 1.1 equiv.) in dry dichloromethane (20 mL). Then, pyridine (0.39 mL, 4.85 mmol, 1.5 equiv.) was added. The crude product was purified by column chromatography (petroleum ether/ethyl acetate: 8:2) to afford product **17b** as pink solid (1.25 g, 70% yield).

Mp 178–180 °C.

^{1}H NMR (500 MHz, $CDCl_3$) δ= 7.58 (d, *J* = 8.5 Hz, 4H), 7.37 (d, *J* = 8.0 Hz, 2H), 7.23 (t, *J* = 8.0 Hz, 1H), 7.19 (d, *J* = 8.5 Hz, 4H), 6.69 (s, 2H), 2.40 (s, 6H) ppm.

^{13}C{^{1}H} NMR (126 MHz, $CDCl_3$) δ= 144.5, 138.5, 135.9, 130.0, 129.8, 127.5, 119.0, 91.3, 21.7 ppm.

IR (neat) $\tilde{\nu}$ = 3000, 1735, 1151, 1085, 719, 657, 550 cm^{-1}.

HRMS (ESP-TOF) m/z: $[M+H]^+$ calcd for $C_{20}H_{20}N_2O_4S_2I$ 542.9909; found: 542.9910.

Dimethyl 2,2'-((2-iodo-1,3-phenylene)bis(tosylazanediyl))(2*R*,2'*R*)-dipropionate (18c)

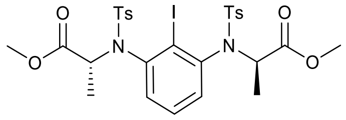

Following GP2 method B, *N*,*N'*-(2-iodo-1,3-phenylene)bis(4-methylbenzenesulfonamide) (0.35 g, 0.65 mmol, 1 equiv.), PPh_3 (0.42 g, 1.61 mmol, 2.5 equiv.), (*S*)-lactic acid ethyl ester (0.17 g, 0.16 ml, 1.61 mmol, 2.5 equiv.), DIAD (0.33 g, 0.32 ml, 1.61 mmol, 2.5 equiv.) and toluene (30 mL) to afford product **18c** as colorless solid (0.32 g, 68% yield).

$[\alpha]_D^{20}$ = +71.74 (c= 0.92, $CHCl_3$).

Mp 173–175 °C.

^{1}H NMR (500 MHz, $CDCl_3$) δ 7.79 – 7.49 (m, 6H), 7.34 (t, *J* = 8.0 Hz, 1H), 7.28 (t, *J* = 8.0 Hz, 1H), 7.21 (d, *J* = 7.5 Hz, 3H), 4.92 and 4.37 (q and q, *J* = 7.0 and 7.5 Hz, 2H), 3.76 and 3.75 (s and s, 1H), 3.63 and 3.60 (s and s 5H), 2.44, 2.42, and 2.41 (s, s and s, 6H), 1.61 and 1.54 (d and d, *J* = 7.0 and 7.0 Hz, 1H), 1.26 and 1.20 (d and d, *J* = 7.5 and 7.5 Hz, 5H) ppm.

^{13}C{^{1}H} NMR (126 MHz, $CDCl_3$) δ 173.0, 172.9, 171.5, 171.3, 143.9, 143.9, 143.9, 143.6, 143.3, 141.3, 137.6, 137.0, 134.6, 134.5, 134.2, 133.5, 129.8, 129.6, 129.4, 129.3, 128.8, 128.5, 128.3,

128.2, 121.3, 117.4, 60.5, 60.3, 57.6, 57.5, 52.6, 52.6, 52.3, 52.3, 21.7, 21.7, 21.7, 17.4, 16.7, 16.6 ppm.

IR (neat) $\tilde{\nu}$ = 3000, 1760, 1452, 1332, 1140, 721, 653, 578 cm^{-1}.

HRMS (ASAP-TOF) m/z: $[M+H]^+$ calcd for $C_{28}H_{32}N_2O_8S_2I$ 715.0645; found 715.0679.

Diethyl 2,2'-((2-iodo-1,3-phenylene)bis(tosylazanediyl))(2*R*,2'*R*)-dipropionate (18d)

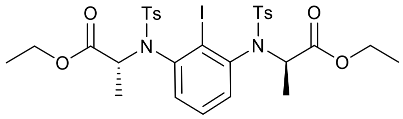

Following GP2 method B, *N,N'*-(2-iodo-1,3-phenylene)bis(4-methylbenzenesulfonamide) (1 g, 1.84 mmol, 1 equiv.), PPh_3 (1.21 g, 4.61 mmol, 2.5 equiv.), (*S*)-lactic acid ethyl ester (0.54 g, 0.52 ml, 4.61 mmol, 2.5 equiv.), DIAD (0.93 g, 0.91 ml, 4.61 mmol, 2.5 equiv.) and toluene (70 ml) to afford product **18d** as white foam (1.1 g, 80% yield).

$[\alpha]_D^{20}$ = +28.33 (c=1.2, $CHCl_3$).

^{1}H NMR (300 MHz, $CDCl_3$) δ= 7.83 – 7.28 (m, 8 H), 7.20 (d, *J* = 8.5 Hz, 3H), 4.89 and 4.28 (q and q, *J* = 7.2 and 7.2 Hz, 2H), 4.23 – 3.97 (m, 4 H), 2.43, 2.41 and 2.40 (s, s and s, 6H), 1.65 and 1.56 (dand d, *J* = 7.2 and 7.2 Hz, 1H), 1.32 – 1.17 (m, 11H) ppm.

^{13}C{^{1}H} NMR (126 MHz, $CDCl_3$) δ = 172.4, 172.4, 171.1, 170.9, 143.8, 143.7, 143.7, 143.5, 141.2, 137.6, 137.1, 137.1, 134.6, 134.6, 134.1, 133.5, 129.7, 129.5, 129.4, 129.4, 129.3, 129.2, 128.7, 128.4, 128.3, 128.2, 128.1, 128.1, 121.3, 117.2, 114.5, 61.6, 61.4, 61.3, 60.9, 60.6, 57.7, 57.5, 21.7, 21.7, 21.6, 21.6, 17.4, 17.4, 16.7, 16.6, 14.2, 14.1, 14.0, 14.0 ppm.

IR (neat) $\tilde{\nu}$ = 3000, 1737, 1344, 1161, 1087, 711, 657, 572 cm^{-1}.

HRMS (ESP-TOF) m/z: $[M+H]^+$ calcd for $C_{30}H_{36}N_2O_8S_2I$ 743.0958; found 743.0959.

Scheme 5: Synthesis of chiral iodophenol catalysts

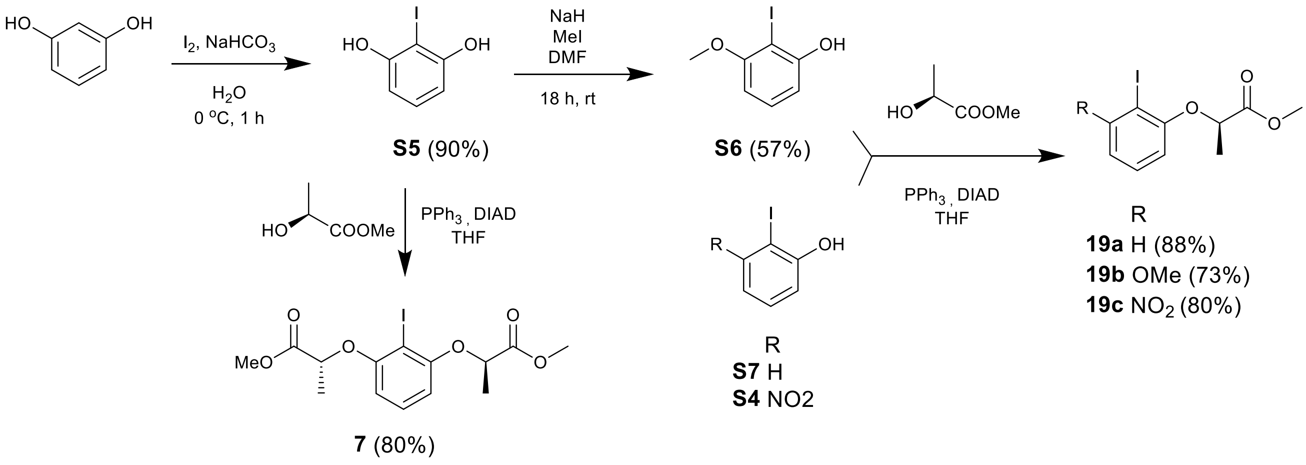

2-Iodobenzene-1,3-diol (S5)

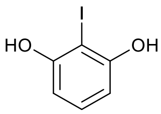

Following reported procedure,[3] resorcinol (10.0 g, 90.8 mmol) was dissolved in 100 mL H_2O. The reaction mixture was cooling to 0 °C, iodine (24.2 g, 95.36 mmol, 1.05 equiv.) was added followed by slow addition of $NaHCO_3$ (8.39 g, 99.9 mmol, 1.1 equiv.). The resulting reaction mixture was stirred for 1 hour at 0 °C and then allowed to warm to room temperature over a period of 1 hour. The reaction was then quenched by addition of saturated aqueous $Na_2S_2O_3$ solution. After that the reaction was extracted with ethyl acetate, the combined organic phases were washed with brine, dried over anhydrous $MgSO_4$ and concentrated under reduced pressure. The crude product was purified by column chromatography with (petroleum ether/ethyl acetate: 1:9) followed by recrystallization from $CHCl_3$ afforded the title product as colorless solid (19 g, 90% yield).

^{1}H NMR (300 MHz, $CDCl_3$) δ = 7.11 (t, *J* = 8.1 Hz, 1H), 6.56 (d, *J* = 8.1 Hz, 2H), 5.31 (s, 2H) ppm.

^{13}C{^{1}H} NMR (125 MHz; $CDCl_3$): δ = 155.8, 130.5, 107.5, 77.8 ppm.

Data are in agreement with the literature.[3]

2-Iodo-3-methoxyphenol (S6)

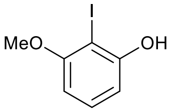

Following the modified reported procedure,[15] Under nitrogen atmosphere, a mixture of 2-iodoresorcinol (3.5 g, 14.83 mmol, 2 eq.) and NaH (0.3 g, 7.41 mmol, 1 eq) in DMF (50 mL) was stirred at room temperature for 30 mins, until the solution dissolved and get clear. Then, the reagent MeI (1.06 g, 0.47 mL, 7.48 mmol, 1.01 eq) was added to the reaction mixture dropwise. The reaction was stirred at room temperature overnight. After completing the reaction, the solvent of reaction was evaporated under reduced pressure. The residue was dissolved in ethyl acetate (50 mL) and washed with water (4 × 30 mL) and brine (2 × 30 mL). Then, the organic layer was dried over anhydrous $MgSO_4$ and concentrated under reduced pressure. The crude was purified by flash column chromatography using (petroleum ether/ethyl acetate: 8:2) to afford product **S6** as colorless oil (2.1 g, 57% yield).

^{1}H NMR (300 MHz, $CDCl_3$) δ = 7.19 (t, *J* = 8.2 Hz, 1H), 6.67 (dd, *J* = 8.2, 1.2 Hz, 1H), 6.40 (dd, *J* = 8.2, 1.1 Hz, 1H), 5.49 (s, 1H), 3.88 (s, 3H) ppm.

^{13}C{^{1}H} NMR (101 MHz, $CDCl_3$) δ = 158.7, 156.1, 130.1, 107.9, 103.0, 78.1, 56.5 ppm.

NMR data are in agreement with the literature.[16]

Methyl (*R*)-2-(2-iodophenoxy)propanoate (19a)

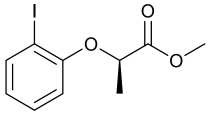

Following GP2 method C, 2-iodophenol (5 g, 22.73 mmol, 1 equiv.), PPh3 (8.9 g, 34.1 mmol, 1.5 equiv.), methyl (*S*)-lactate (3.55 g, 3.26 mL, 34.1 mmol, 1.5 equiv.), DIAD (6.9 g, 6.72 mL, 34.1 mmol, 1.5 equiv.) and THF (100 mL) to afford product **19a** as colorless oil (6 g, 88% yield).

^{1}H NMR (500 MHz, $CDCl_3$) δ = 7.77 (d, *J* = 7.6 Hz, 1H), 7.23 (t, *J* = 7.6 Hz, 1H), 6.72 (t, *J* = 7.6 Hz, 1H), 6.69 (d, *J* = 7.6 Hz, 1H), 4.74 (q, *J* = 6.9 Hz, 1H), 3.74 (s, 3H), 1.68 (d, *J* = 6.9 Hz, 3H) ppm.

^{13}C{^{1}H} NMR (126 MHz, $CDCl_3$) δ = 172.2, 156.7, 139.9, 129.5, 123.6, 113.5, 87.4, 74.2, 52.5, 18.7 ppm.

NMR data are in agreement with the literature.[2]

Methyl (*R*)-2-(2-iodo-3-methoxyphenoxy)propanoate (19b)

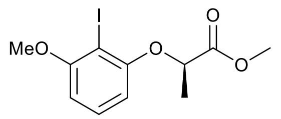

Following GP2 method C, 2-iodophenol (5 g, 22.7 mmol), PPh_3 (8.9 g, 34.1 mmol, 1.5 equiv.), methyl (*S*)-lactate (3.55 g, 3.26 ml, 34.1 mmol, 1.5 equiv.), DIAD (6.9 g, 6.72 mL, 34.1 mmol, 1.5 equiv.) were reacted in THF (80 mL) to afford the product **19b** as colorless oil (3.5 g, 73% yield).

^{1}H NMR (500 MHz, $CDCl_3$) δ = 7.18 (t, *J* = 8.2 Hz, 1H), 6.50 (d, *J* = 8.2 Hz, 1H), 6.34 (d, *J* = 8.2 Hz, 1H), 4.76 (q, *J* = 6.9 Hz, 1H), 3.87 (s, 3H), 3.73 (s, 3H), 1.68 ppm (d, *J* = 6.9 Hz, 3H) ppm.

^{13}C{^{1}H} NMR (126 MHz, $CDCl_3$) δ = 172.3, 159.9, 158.1, 129.8, 106.3, 105.0, 79.3, 74.4, 56.7, 52.5, 18.7 ppm.

NMR data are in agreement with the literature.[17]

Methyl (*R*)-2-(2-iodo-3-nitrophenoxy)propanoate (19c)

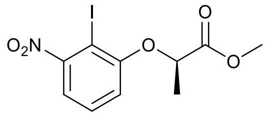

Following GP2 method C, 2-iodophenol (0.19 g, 0.71 mmol), PPh_3 (0.28 g, 1.07 mmol, 1.5 equiv.), methyl (*S*)-lactate (0.11 g, 1.0 mL, 1.07 mmol, 1.5 equiv.), DIAD (0.22 g, 0.21 mL, 1.07 mmol, 1.5 equiv.) and THF (30 mL) to afford a title product as yellow solid (0.2 g, 80% yield).

$[\alpha]_D^{20}$ = − 13.3 (c =0.6, $CHCl_3$).

Mp 128–130 °C.

^{1}H NMR (500 MHz, $CDCl_3$) δ = 7.37 (t, *J* = 8.0 Hz, 1H), 7.32 (dd, *J* = 8.0, 1.5 Hz, 1H), 6.84 (dd, *J* = 8.0, 1.5 Hz, 1H), 4.83 (q, *J* = 6.5 Hz, 1H), 3.77 (s, 3H), 1.75 (d, *J* = 7 Hz, 3H) ppm.

^{13}C{^{1}H} NMR (126 MHz, $CDCl_3$) δ = 171.3, 158.3, 156.0, 130.0, 117.8, 115.5, 81.3, 77.1, 74.8, 52.7, 18.6 ppm.

IR (neat) $\tilde{\nu}$ = 1728, 1531, 1452, 1350, 1230, 1109, 788, 734 cm^{-1}.

HRMS (ESP-TOF) m/z: $[M]^+$ calcd for $C_{10}H_{10}NO_5I$ 350.9598; found 350.9599.

Dimethyl 2,2'-((2-iodo-1,3-phenylene)bis(oxy))(2*R*,2'*R*)-dipropionate (7)

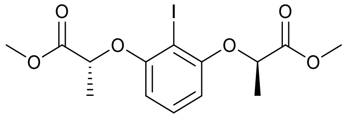

Following GP2 method D, 2-iodoresorcinol (5.5 g, 23.30 mmol), PPh_3 (14.06 g, 58.25 mmol, 2.5 equiv.), methyl (*S*)-lactate (0.11 g, 1.0 mL, 58.25 mmol, 2.5 equiv.), DIAD (0.22 g, 0.21 mL, 58.25 mmol, 2.5 equiv.) were reacted in THF (150 mL) to afford the product **7** as colorless solid (0.2 g, 80% yield).

^{1}H NMR (500 MHz, $CDCl_3$) δ = 7.12 (t, *J* = 8.2 Hz, 1H), 6.34 (d, *J* = 8.2 Hz, 2H), 4.74 (q, *J* = 6.9 Hz, 2H), 3.72 (s, 6H), 1.68 (d, *J* = 6.9 Hz, 6H) ppm.

^{13}C{^{1}H} NMR (126 MHz, $CDCl_3$) δ = 172.3, 158.4, 129.7, 107.0, 80.8, 74.3, 52.5, 18.7 ppm.

NMR data are in agreement with the literature.[3]

Substrate scope of the enantioselective α-oxysulfonylation of ketone derivatives:

(*S*)-1-Oxo-1-phenylpropan-2-yl 4-methylbenzenesulfonate (20a)

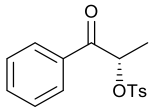

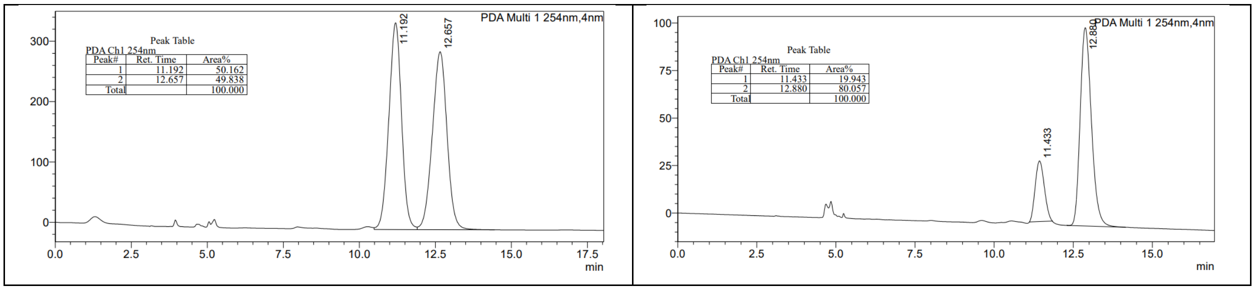

Following GP3 Method A: chiral iodine catalyst **18b** (22 mg, 0.037 mmol, 10 mol%, 0.1 equiv.), *m*CPBA 77% pure (250 mg, 1.12 mmol, 3 equiv.), *p*-TsOH•H_2O (190 mg, 1.12 mmol, 3 equiv.), and propiophenone (50 mg, 0.37 mmol, 1 equiv.) in 1 mL of ethyl acetate to afford product **20a** as a colorless solid (110 mg, 0.37 mmol, 99% yield).

Scale up reaction for synthesis (**20a**):

Following GP3 Method A: chiral iodine catalyst **18b** (0.44 g, 0.745 mmol, 10 mol%, 0.1 equiv.), *m*CPBA 77% pure (5 g, 22.36 mmol, 3 equiv.), *p*-TsOH•H_2O (4.25 g, 22.36 mmol, 3 equiv.), and propiophenone (1 g, 0.37 mmol, 1 equiv.) in (20 mL) of ethyl acetate to afford product **20a** as a colorless solid (2.1 g, 6.90 mmol, 92% yield, 60% *ee*).

$[\alpha]_D^{20}$ = –18.3 (c =1.2, $CHCl_3$).

Mp 64–65 °C.

Enantiomeric excess is determined by HPLC CHIRALPAK® IA: 5 μm, (*n*-hexane/*i*-PrOH = 90/10, flow rate = 1.0 mL/min, 254 nm). Minor isomer: t_R = 11.34 min, major isomer: t_R = 12.88 min, *ee* = 60%.

^{1}H NMR (300 MHz, $CDCl_3$) δ = 7.87 (dd, *J* = 8.7, 1.5 Hz, 2H), 7.75 (d, *J* = 8.1 Hz, 2H), 7.62 – 7.55 (m, 1H), 7.45 (t, *J* = 7.6 Hz, 2H), 7.26 (dd, *J* = 8.5, 1 Hz, 2H), 5.78 (q, *J* = 7 Hz, 1H), 2.40 (s, 3H), 1.59 (d, *J* = 7 Hz, 3H) ppm.

^{13}C{^{1}H} NMR (75 MHz, $CDCl_3$) δ = 194.9, 145.1, 133.9, 133.7, 133.5, 129.8, 128.8, 128.8, 128.0, 77.5, 21.7, 18.8 ppm.

The data of this compound are in agreement with the literature.[1]

(*S*)-1-(3-Chlorophenyl)-1-oxopropan-2-yl 4-methylbenzenesulfonate (20b)

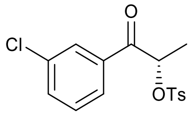

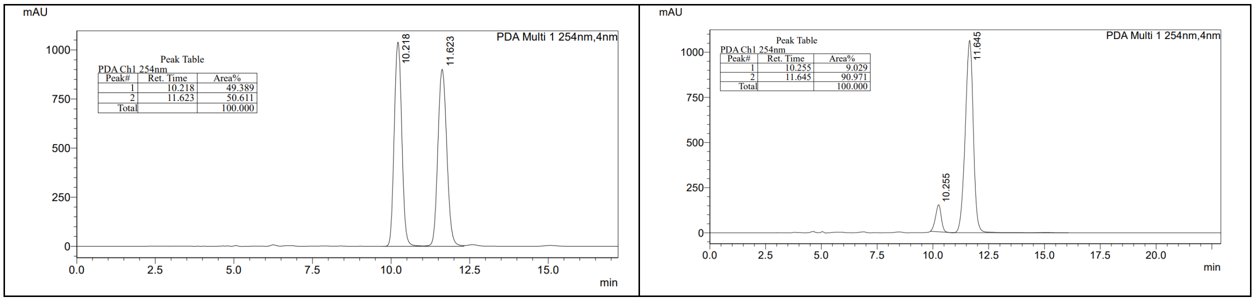

Following GP3 Method A: chiral iodine catalyst **18b** (18 mg, 0.029 mmol, 10 mol%, 0.1 equiv.), *m*CPBA 77% pure (200 mg, 0.89 mmol, 3 equiv.), *p*-TsOH•H_2O (170 mg, 0.89 mmol, 3 equiv.), and 3-chloropropiophenone (50 mg, 0.29 mmol, 1 equiv.) in ethyl acetate (1 mL) to afford the product **20b** as a colorless solid (69 mg, 0.20 mmol, 69% yield).

$[\alpha]_D^{20}$ = –5 (c= 1.2, $CHCl_3$).

Mp 145–147 °C.

Enantiomeric excess is determined by HPLC CHIRALPAK® IA: 5 μm, (*n*-hexane/*i*-PrOH = 90/10, flow rate = 1.0 mL/min, 254 nm). Minor isomer: t_R = 10.25 min, major isomer: t_R = 11.64 min, *ee* = 81%.

^{1}H NMR (500 MHz, $CDCl_3$) δ = 7.80 – 7.76 (m, 2H), 7.73 (d, *J* = 8 Hz, 2H), 7.54 -7.56 (m, 1H), 7.39 (t, *J* = 8 Hz, 1H), 7.27 (d, *J* = 8 Hz, 2H), 5.68 (q, *J* = 7 Hz, 1H), 2.42 (s, 3H), 1.59 (d, *J* = 7.0 Hz, 3H) ppm.

$^{13}C\{^1H\}$ **NMR** (126 MHz, $CDCl_3$) δ = 194.1, 145.3, 135.3, 135.2, 133.8, 133.3, 130.1, 129.9, 128.9, 128.0, 126.9, 77.5, 21.7, 18.7 ppm.

The data of this compound are in agreement with the literature.[18]

(*S*)-1-(3-Bromophenyl)-1-oxopropan-2-yl 4-methylbenzenesulfonate (20c)

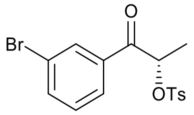

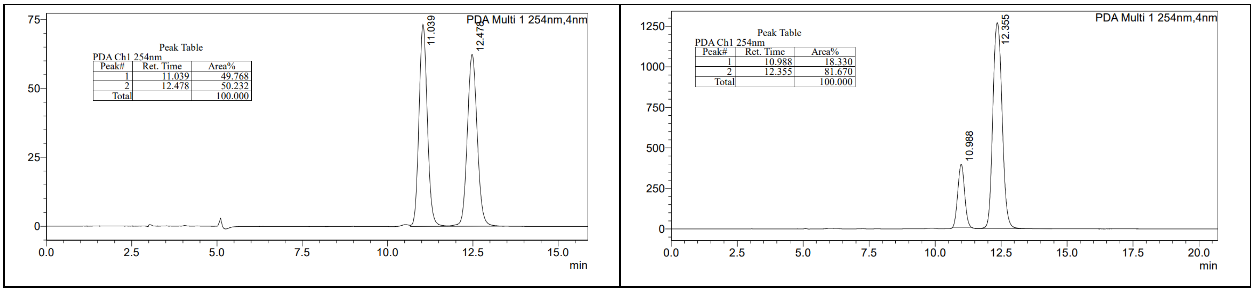

Following GP3 Method A: chiral iodine catalyst **18b** (14 mg, 0.023 mmol, 10 mol%, 0.1 equiv.), *m*CPBA 77% pure (160 mg, 0.70 mmol, 3 equiv.), *p*-TsOH•H_2O (130 mg, 0.70 mmol, 3 equiv.), and 3-bromopropeophenone (50 mg, 0.23 mmol, 1 equiv.) in (1 mL) of ethyl acetate to afford product **20c** as a colorless solid (73 mg, 0.19 mmol, 81% yield). $[\alpha]_D^{20}$ = - 27.5 (c= 0.8, $CHCl_3$).

Enantiomeric excess is determined by HPLC CHIRALPAK® IA: 5 *µ*m, (*n*-hexane/*i*-PrOH = 90/10, flow rate = 1.0 mL/min, 254 nm). Minor isomer: t_R = ~~10.89~~ 10.98 min, major isomer: t_R = 12.35 min, *ee* = 63%.

^1H **NMR** (500 MHz, $CDCl_3$) δ= 7.94 (t, *J* = 1.5 Hz, 1H), 7.82 (ddd, *J* = 8, 2, 1.0 Hz, 1H), 7.73 (d, *J* = 8.5 Hz, 2H), 7.70 (ddd, *J* = 8.0, 2.0, 1.0 Hz, 1H), 7.33 (t, *J* = 8 Hz, 1H), 7.27 (dd, *J* = 8.5, 0.5 Hz, 2H), 5.68 (q, *J* = 7.0 Hz, 1H), 2.42 (s, 3H), 1.60 (d, *J* = 7.0 Hz, 3H) ppm.

$^{13}C\{^1H\}$ **NMR** (126 MHz, $CDCl_3$) δ= 194.0, 145.4, 136.7, 135.5, 133.3, 131.8, 130.4, 129.9, 128.1, 127.4, 123.2, 77.5, 21.8, 18.7 ppm.

IR (neat) $\tilde{\nu}$ = 2326, 2252, 1440, 1037, 918, 445 cm^{-1}.

HRMS (ESI-TOF) m/z: $[M+H]^+$ calcd for $C_{16}H_{16}O_4SBr$ 382.9953; found 382.9945.

(*S*)-1-(3-Nitrophenyl)-1-oxopropan-2-yl 4-methylbenzenesulfonate (20d)

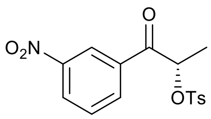

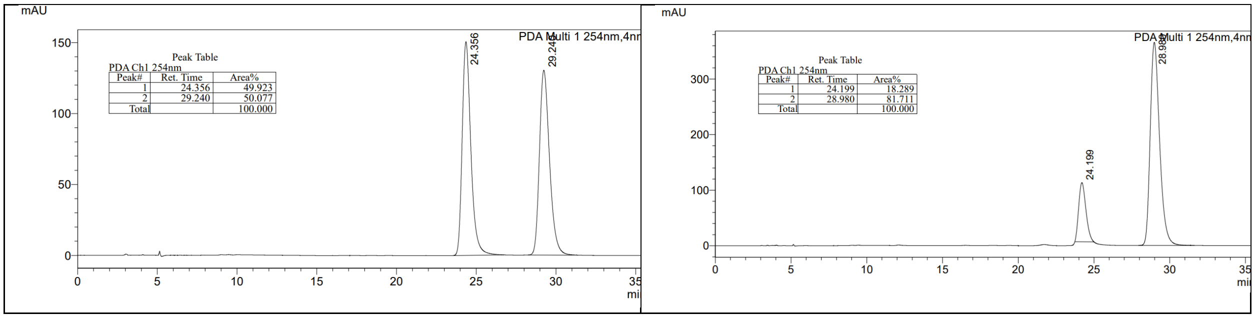

Following GP3 method B: chiral iodine catalyst **18b** (16 mg, 0.028 mmol, 10 mol%, 0.1 equiv.), *m*CPBA 77% pure (190 mg, 0.84 mmol, 3 equiv.), *p*-TsOH•H_2O (160 mg, 0.84 mmol, 3 equiv.), and 1-(3-nitrophenyl)propan-1-one (50 mg, 0.28 mmol, 1 equiv.) in 1 ml of ethyl acetate to afford product **20d** as a colorless solid (80 mg, 0.23 mmol, 88% yield).

$[\alpha]_D^{20}$ = –20.0 (c= 1.2, $CHCl_3$).

Mp 80–82 °C.

Enantiomeric excess is determined by HPLC CHIRALPAK® IA: 5 μm, (*n*-hexane/*i*-PrOH = 90/10, flow rate = 1.0 mL/min, 254 nm). Minor isomer: t_R = 24.19 min, major isomer: t_R = 28.98 min, *ee* = 63%.

^{1}H NMR (400 MHz, $CDCl_3$) δ = 8.67 (t, *J* = 2.0 Hz, 1H), 8.46 -8.41 (m, 1H), 8.29 - 8.25 (m, 1H), 7.74 (d, *J* = 8.5 Hz, 2H), 7.69 (t, *J* = 8.0 Hz, 1H), 7.29 (dd, *J* = 8.4, 0.5 Hz, 2H), 5.68 (q, *J* = 6.8 Hz, 1H), 2.42 (s, 3H), 1.63 (d, *J* = 6.8 Hz, 3H) ppm.

^{13}C{^{1}H} NMR (101 MHz, $CDCl_3$) δ = 193.6, 148.5, 145.6, 135.1, 134.5, 133.1, 130.1, 130.0, 128.0, 128.0, 123.8, 77.7, 21.7, 18.5 ppm.

The data of this compound are in agreement with the literature.[1]

(*S*)-1-Oxo-1-(3-(trifluoromethyl)phenyl)propan-2-yl 4-methylbenzenesulfonate (20e)

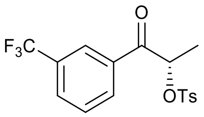

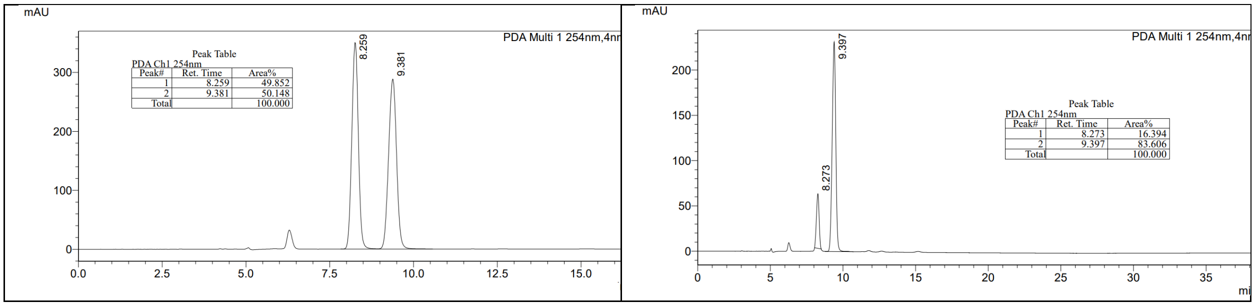

Following GP3 Method B: chiral iodine catalyst **18b** (18 mg, 0.029 mmol, 10 mol%, 0.1 equiv.), *m*CPBA 77% pure (200 mg, 0.89 mmol, 3 equiv.), *p*-TsOH•H_2O (170 mg, 0.89 mmol, 3 equiv.), and 1-(3-(trifluoromethyl)phenyl)propan-1-one (0.5 ml, 60 mg, 0.29 mmol, 1 equiv.) in 1 ml of ethyl acetate to afford product **20e** as a colouless solid (79 mg, 0.21 mmol, 74% yield).

$[\alpha]_D^{20}$ = –17.8 (c= 1.4, $CHCl_3$).

Mp 100–101 °C.

Enantiomeric excess is determined by HPLC CHIRALPAK® IA: 5 μm, (*n*-hexane/*i*-PrOH = 90/10, flow rate = 1.0 mL/min, 254 nm). Minor isomer: t_R = 8.27 min, major isomer: t_R = 9.39 min, *ee* = 67%.

^{1}H NMR (500 MHz, $CDCl_3$) δ 8.10 (d, *J* = 9 Hz, 2H), 7.83 (d, *J* = 7.5 Hz, 1H), 7.72 (d, *J* = 8 Hz, 2H), 7.61 (t, *J* = 7.7 Hz, 1H), 7.28 – 7.19 (m, 2H), 5.71 (q, *J* = 7.0 Hz, 1H), 2.40 (s, 3H), 1.61 (d, *J* = 7.0 Hz, 3H) ppm.

^{13}C{^{1}H} NMR (126 MHz, $CDCl_3$) δ 194.2, 145.4, 134.3, 133.2, 132.0, 130.2 (q, *J* = 3.5 Hz), 129.9, 129.5, 128.0, 125.8 (q, *J* = 3.8 Hz), 77.7, 21.7, 18.6 ppm.

The data of this compound are in agreement with the literature.[1,18]

(*S*)-1-(4-Fluorophenyl)-1-oxopropan-2-yl 4-methylbenzenesulfonate (20f)

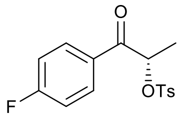

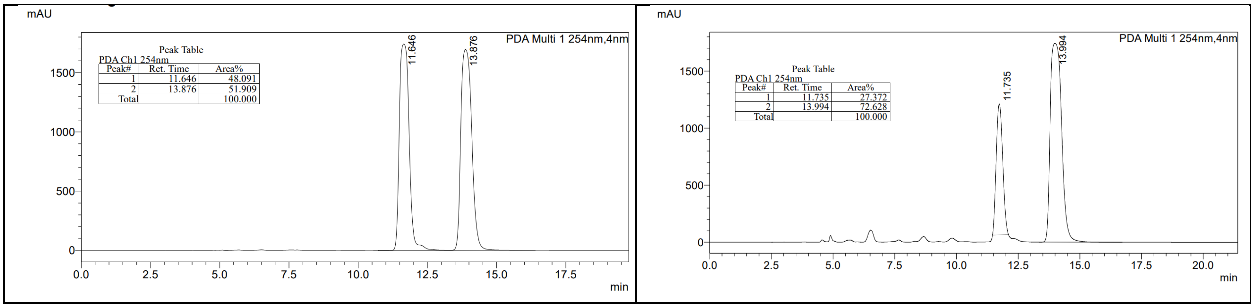

Following GP3 Method A: chiral iodine catalyst **18b** (22 mg, 0.036 mmol, 10 mol%, 0.1 equiv.), *m*CPBA 77% pure (240 mg, 1.08 mmol, 3 equiv.), *p*-TsOH•H_2O (210 mg, 1.08 mmol, 3 equiv.), and 4-fluoropropeophenone (0.5 ml, 54 mg, 0.36 mmol, 1 equiv.) in 1 ml of ethyl acetate to afford product **20f** as a colorless solid (85 mg, 0.26 mmol, 74 yield).

$[\alpha]_D$ ^{20} = –10 (c= 1.4, $CHCl_3$).

Mp 68–70 °C.

Enantiomeric excess is determined by HPLC CHIRALPAK® IA: 5 μm, (*n*-hexane/*i*-PrOH = 90/10, flow rate = 1.0 mL/min, 254 nm). Minor isomer: t_R = 11.73 min, major isomer: t_R = 13.99 min, *ee* = 45%.

^{1}H NMR (500 MHz, $CDCl_3$) δ = 7.95 – 7.92 (m, 2H), 7.74 (d, *J* = 8.5 Hz, 2H), 7.27 (d, *J* = 8 Hz, 2H), 7.12 (t, *J* = 9 Hz, 2H), 5.70 (q, *J* = 7 Hz, 1H), 2.41 (s, 3H), 1.58 (d, *J* = 7 Hz, 3H) ppm.

^{13}C{^{1}H} NMR (126 MHz, $CDCl_3$) δ = 193.5, 167.2, 165.1, 145.3, 133.5, 131.8, 131.7, 129.9, 128.0, 116.2, 116.0, 77.6, 21.7, 18.7 ppm.

The data of this compound are in agreement with the literature.[19]

(*S*)-1-(4-Bromophenyl)-1-oxopropan-2-yl 4-methylbenzenesulfonate (20g)

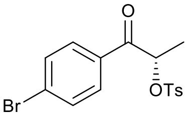

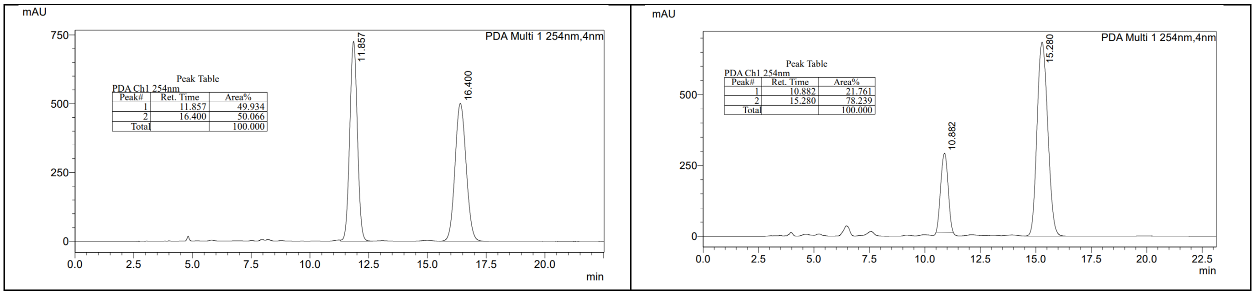

Following GP3 Method A: chiral iodine catalyst **18b** (14 mg, 0.023 mmol, 10 mol%, 0.1 equiv.), *m*CPBA 77% pure (160 mg, 0.70 mmol, 3 equiv.), *p*-TsOH•H_2O (130 mg, 0.70 mmol, 3 equiv.), and 4-bromopropeophenone (50 mg, 0.23 mmol, 1 equiv.) in 1 ml of ethyl acetate to afford product **20g** as a colorless solid (45 mg, 0.12 mmol, 50% yield).

Mp 66–68 °C.

$[\alpha]_D^{20}$ = –37.5 (c= 0.8, $CHCl_3$).

Enantiomeric excess is determined by HPLC YMC Chiral Amylose-C S-5μm (25 cm), (*n*-hexane/*i*-PrOH = 80/20, flow rate = 1.0 mL/min, 254 nm). Minor isomer: t_R = 10.88 min, major isomer: t_R = 15.28 min, *ee* = 56%.

^{1}H NMR (500 MHz, $CDCl_3$) δ = 7.74 (dd, *J* = 11, 8.5 Hz, 4H), 7.59 (d, *J* = 9 Hz, 2H), 7.28 (d, *J* = 8 Hz, 2H), 5.67 (q, *J* = 7 Hz, 1H), 2.42 (s, 3H), 1.58 (d, *J* = 7 Hz, 3H) ppm.

^{13}C{^{1}H} NMR (126 MHz, $CDCl_3$) δ = 194.2, 145.3, 133.4, 132.5, 132.2, 130.4, 129.9, 129.3, 128.0, 77.6, 21.8, 18.7 ppm.

The data of this compound are in agreement with the literature.[20]

(*S*)-1-Oxo-1-(4-(trifluoromethyl)phenyl)propan-2-yl 4-methylbenzenesulfonate (20h)

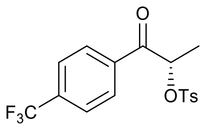

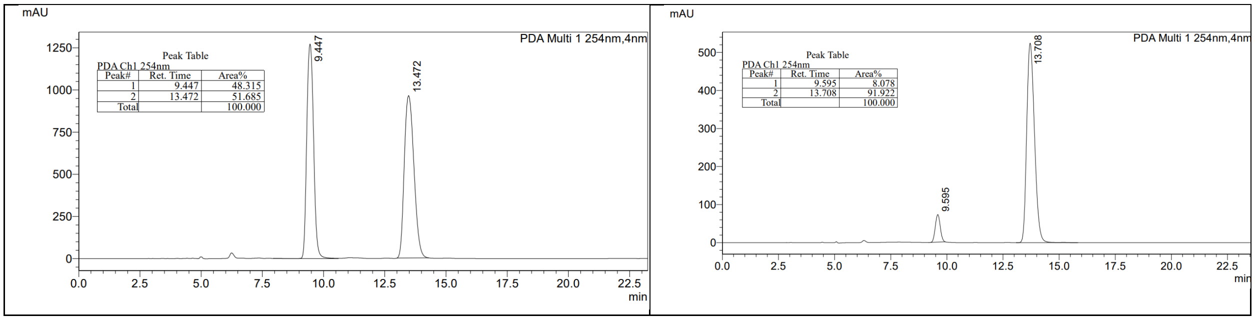

Following GP3, method B: chiral iodine catalyst **18b** (18 mg, 0.029 mmol, 10 mol%, 0.1 equiv.), *m*CPBA 77% pure (200 mg, 0.89 mmol, 3 equiv.), *p*-TsOH•H_2O (170 mg, 0.89 mmol, 3 equiv.), and 1-(4-(trifluoromethyl)phenyl)propan-1-one (0.5 mL, 60 mg, 0.29 mmol, 1 equiv.) in ethyl acetate (1 mL) to afford product **20h** as a colorless solid (85 mg, 0.22 mmol, 80% yield).

Mp 88 °C.

$[\alpha]_D^{20}$ = –20.0 (c = 1.6, $CHCl_3$).

Enantiomeric excess is determined by HPLC CHIRALPAK® IA: 5 μm, (*n*-hexane/*i*-PrOH = 90/10, flow rate = 1.0 mL/min, 254 nm). Minor isomer: t_R = 9.59 min, major isomer: t_R = 13.70 min, *ee* = 83%.

^{1}H NMR (500 MHz, $CDCl_3$) δ = 7.99 (d, *J* = 8.5 Hz, 2H), 7.72 (t, *J* = 8 Hz, 4H), 7.29 – 7.25 (m, 2H), 5.70 (q, *J* = 6.5 Hz, 1H), 2.42 (s, 3H), 1.60 (d, *J* = 7.0 Hz, 3H) ppm.

^{13}C{^{1}H} NMR (126 MHz, $CDCl_3$) δ = 194.5, 145.4, 136.6, 135.0 (q, *J* = 32.9 Hz), 133.3, 130.0, 129.3, 128.0, 125.8 (q, *J* = 3.7 Hz), 77.8, 21.7, 18.5 ppm.

The data of this compound are in agreement with the literature.[21]

(*S*)-1-Oxo-1-(p-tolyl)propan-2-yl 4-methylbenzenesulfonate (20i)

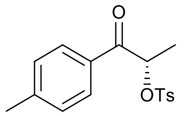

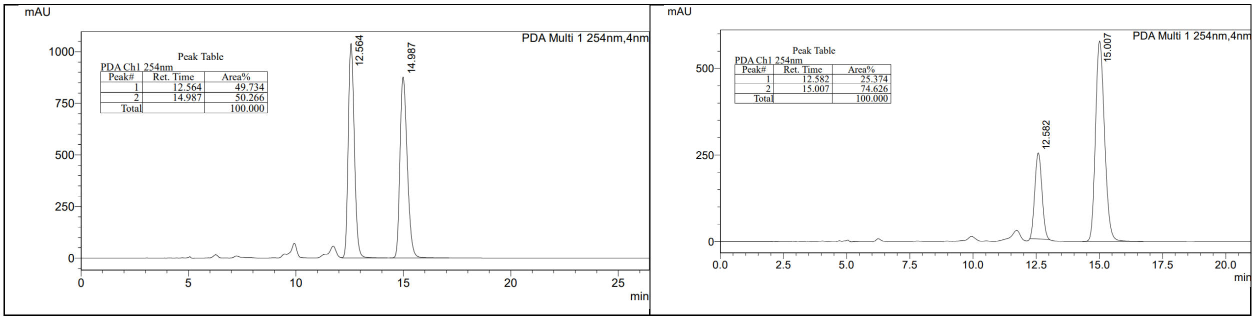

Following GP3, method A: chiral iodine catalyst **18b** (20 mg, 0.033 mmol, 10 mol%, 0.1 equiv.), *m*CPBA 77% pure (230 mg, 1.012 mmol, 3 equiv.), *p*-TsOH•H_2O (190 mg, 1.012 mmol, 3 equiv.), and 4-methylpropiophenone (50 mg, 0.33 mmol, 1 equiv) in ethyl acetate (1 mL) to afford compound **20i** as a colorless solid (64 mg, 0.20 mmol, 61% yield).
Enantiomeric excess is determined by HPLC CHIRALPAK® IA: 5 μm, (*n*-hexane/*i*-PrOH = 90/10, flow rate = 1.0 mL/min, 254 nm). Minor isomer: t_R = 12.58 min, major isomer: t_R = 15.00 min, *ee* = 49%.

Mp 85–86 °C.

^{1}H NMR (500 MHz, $CDCl_3$): δ = 7.79-7.75 (m, 4H), 7.27 – 7.24 (m, 4H), 5.77 (q, J = 7 Hz, 1H), 2.41 (s, 3H), 2.41 (s, 3H), 1.58 (d, J = 7 Hz, 3H) ppm.

^{13}C{^{1}H} NMR (126 MHz, $CDCl_3$) δ = 194.4, 145.0, 145.0, 133.6, 131.3, 129.8, 129.6, 129.0, 128.0, 77.5, 21.8, 21.7, 18.9 ppm.

The data of this compound are in agreement with the literature.[1]

(*S*)-1-(4-Methoxyphenyl)-1-oxopropan-2-yl 4-methylbenzenesulfonate (20j)

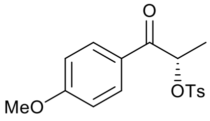

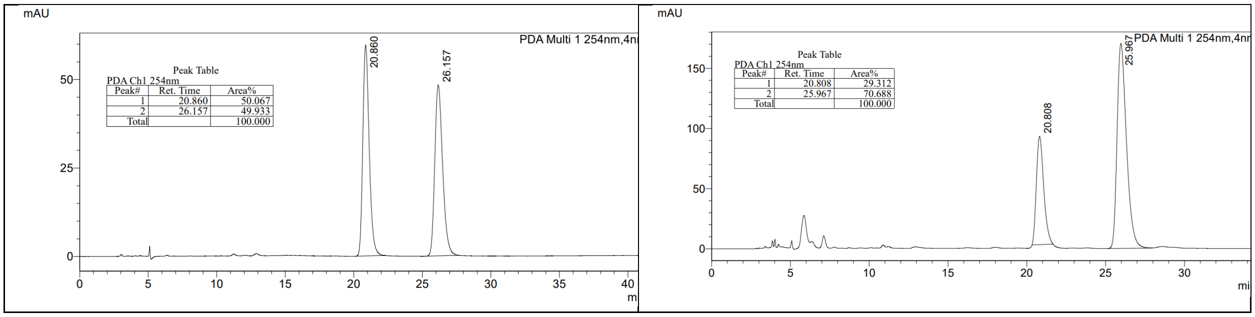

Following GP3, method A: chiral iodine catalyst **18b** (17 mg, 0.029 mmol, 10 mol%, 0.1 equiv.), *m*CPBA 77% pure (200 mg, 0.86 mmol, 3 equiv.), *p*-TsOH•H_2O (160 mg, 0.86 mmol, 3 equiv.), and 4-methoxypropiophenone (50 mg, 0.29 mmol, 1 equiv) in ethyl acetate (1 mL) to afford the product **20j** as a colorless solid (30 mg, 0.09 mmol, 34% yield).

$[\alpha]_D^{20}$ = –15.0 (c= 1.6, $CHCl_3$).

M.p.: 77–78 °C.

Enantiomeric excess is determined by HPLC CHIRALPAK® IA: 5 μm, (*n*-hexane/*i*-PrOH = 90/10, flow rate = 1.0 mL/min, 254 nm). Minor isomer: t_R = 20.80 min, major isomer: t_R = 25.96 min, *ee* = 41%.

^{1}H NMR (500 MHz, $CDCl_3$) δ = 7.88 (d, *J* = 9.0 Hz, 2H), 7.75 (d, *J* = 8.5 Hz, 2H), 7.27 (d, *J* = 0.5 Hz, 1H), 7.26 (d, *J* = 0.5 Hz, 1H), 6.92 (d, *J* = 9.0 Hz, 2H), 5.73 (q, *J* = 7 Hz, 1H), 3.88 (s, 3H), 2.41 (s, 3H), 1.58 (d, *J* = 7 Hz, 3H) ppm.

^{13}C{^{1}H} NMR (126 MHz, $CDCl_3$) δ = 193.2, 164.2, 145.0, 133.7, 131.3, 129.8, 128.1, 126.6, 114.1, 77.5, 55.7, 21.7, 19.0 ppm.

The data of this compound matches with previously reported literature data.[1]

(*S*)-1-(4-(*tert*-Butyl)phenyl)-1-oxopropan-2-yl 4-methylbenzenesulfonate (20k)

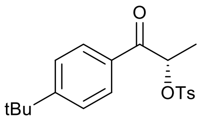

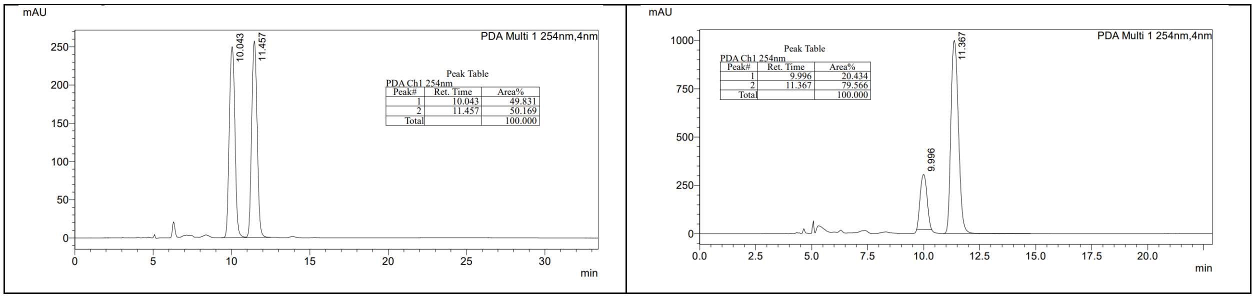

Following GP3, method A: chiral iodine catalyst **18b** (16 mg, 0.026 mmol, 10 mol%, 0.1 equiv.), *m*CPBA 77% pure (180 mg, 0.78 mmol, 3 equiv.), *p*-TsOH•H_2O (150 mg, 0.78 mmol, 3 equiv.), and 4-*tert*-butylpropeophenone (0.052 ml, 50 mg, 0.26 mmol, 1 equiv.) in ethyl acetate (1 mL) to afford product **20k** as a colorless solid (65 mg, 0.18 mmol, 73% yield).

$[\alpha]_D^{20}$ = –11.6 (c= 1.2, $CHCl_3$).

Mp 60–63 °C.

Enantiomeric excess is determined by HPLC CHIRALPAK® IA: 5 μm, (*n*-hexane/*i*-PrOH = 90/10, flow rate = 1.0 mL/min, 254 nm). Minor isomer: t_R = 9.99 min, major isomer: t_R = 11.36 min, *ee* = 59%.

^{1}H NMR (500 MHz, $CDCl_3$) δ = 7.82 (d, *J* = 9 Hz, 2H), 7.75 (d, *J* = 8.5 Hz, 2H), 7.46 (d, *J* = 9 Hz, 2H), 7.25 (d, *J* = 8.5 Hz, 2H), 5.77 (q, *J* = 7 Hz, 1H), 2.40 (s, 3H), 1.59 (d, *J* = 7 Hz, 3H), 1.34 (s, 9H) ppm.

^{13}C{^{1}H} NMR (101 MHz, $CDCl_3$) δ = 194.4, 157.9, 145.0, 133.6, 131.1, 129.8, 128.8, 128.0, 125.8, 77.5, 35.3, 31.1, 21.7, 18.9 ppm.

The data of this compound are in agreement with the literature.[1]

(*S*)-1-Oxo-1-phenylbutan-2-yl 4-methylbenzenesulfonate (20l)

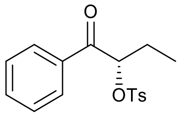

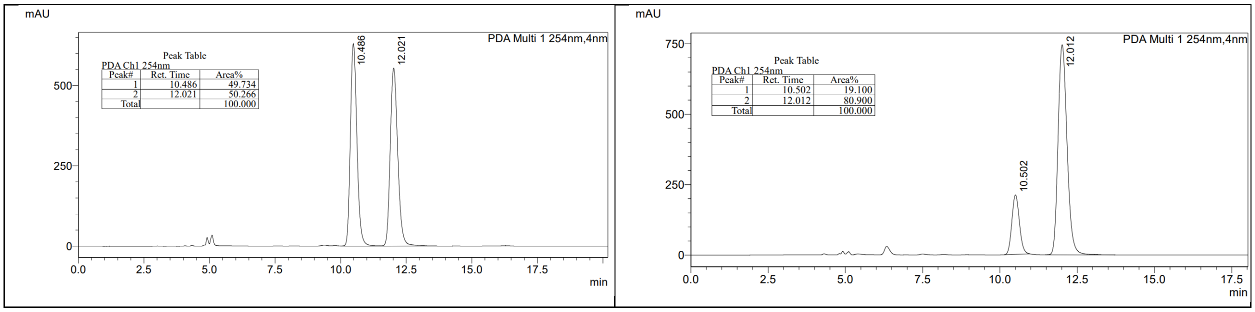

Following GP3, method A: chiral iodine catalyst **18b** (16 mg, 0.028 mmol, 10 mol%, 0.1 equiv.), *m*CPBA 77% pure (190 mg, 0.84 mmol, 3 equiv.), *p*-TsOH•H_2O (160 mg, 0.84 mmol, 3 equiv.), and 1-phenylbutan-1-one (0.42 ml, 41 mg, 0.28 mmol) in ethyl acetate (1 mL) to afford a colorless solid (62 mg, 0.19 mmol, 79% yield).

Enantiomeric excess is determined by HPLC CHIRALPAK® IA: 5 μm, (*n*-hexane/*i*-PrOH = 90/10, flow rate = 1.0 mL/min, 254 nm). Minor isomer: t_R = 10.50 min, major isomer: t_R = 12.01 min, *ee* = 62 %.
[α]$_D^{20}$ = −18.3 (c = 1.2, $CHCl_3$).

Mp 63–65 °C.

^{1}H NMR (300 MHz, $CDCl_3$) δ = 7.85 (dd, *J* = 8.4, 1.2 Hz, 2 H), 7.74 (d, *J* = 8.1 Hz, 2H), 7.62 – 7.55 (m, 1H), 7.48 – 7.41 (m, 2H), 7.24 (dd, *J* = 8.7, 0.6 Hz, 2H), 5.55 (dd, *J* = 7.8, 5.4 Hz, 1H), 2.40 (s, 3H), 2.04 – 1.84 (m, 2H), 0.98 (t, *J* = 7.5 Hz, 3H) ppm.

^{13}C{^{1}H} NMR (126 MHz, $CDCl_3$) δ = 195.0, 145.0, 134.3, 133.8, 133.4, 129.8, 128.8, 128.7, 128.1, 82.6, 26.3, 21.7, 9.6 ppm.

The data of this compound are in agreement with the literature.[1]

(*S*)-1-Oxo-1-phenyloctan-2-yl 4-methylbenzenesulfonate (20m)

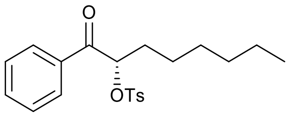

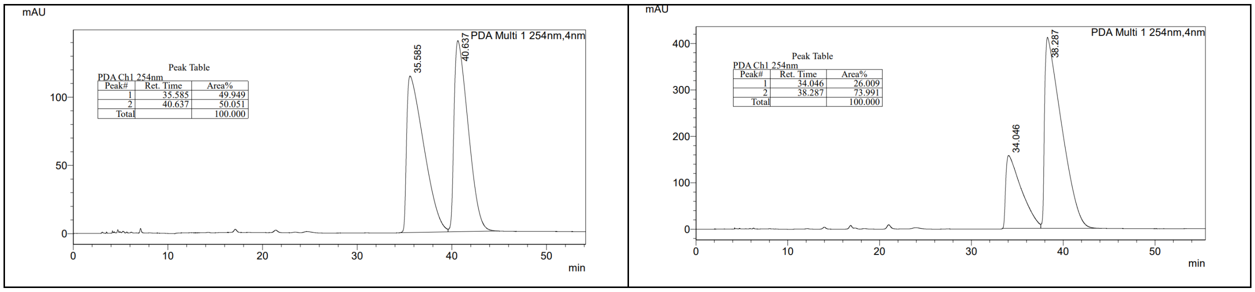

Following GP3, method A: chiral iodine catalyst 18b (14 mg, 0.029 mmol, 10 mol%, 0.1 equiv.), *m*CPBA 77% pure (200 mg, 0.88 mmol, 3 equiv.), *p*-TsOH•H_2O (170 mg, 0.88 mmol, 3 equiv.), and 1-phenyloctan-1-one (60 mg, 0.29 mmol, 1 equiv.) in ethyl acetate (1 mL) to afford the product **20m** as a colorless solid (97 mg, 0.26 mmol, 91% yield).

[α]$_D^{20}$ = –6.25 (c = 1.6, $CHCl_3$).

Mp 56–59 °C.

Enantiomeric excess is determined by HPLC CHIRALPAK® IA: 5 μm, (*n*-hexane/*i*-PrOH = 99/1, flow rate = 1.0 mL/min, 254 nm). Minor isomer: t_R = 34.04 min, major isomer: t_R = 38.28 min, *ee* = 48 %.
^{1}H NMR (300 MHz, $CDCl_3$) δ = 7.85 (dd, *J* = 8.5, 1.3 Hz, 2H), 7.76 – 7.71 (m, 2H), 7.62 – 7.54 (m, 1H), 7.45 (tt, *J* = 6.7, 1.1 Hz, 2H), 7.24 (dd, *J* = 8.6, 0.7 Hz, 2H), 5.58 (dd, *J* = 7.8, 5.2 Hz, 1H), 2.40 (s, 3H), 2.04 – 1.78 (m, 2 H), 1.47 – 1.09 (m, 8H), 0.84 (t, *J* = 6.8 Hz, 3H) ppm.

^{13}C{^{1}H} NMR (75 MHz, $CDCl_3$) δ = 195.2, 145.0, 134.2, 133.8, 133.3, 129.8, 128.8, 128.8, 128.1, 81.5, 32.8, 31.5, 28.6, 25.1, 22.5, 21.7, 14.1 ppm.

The data of this compound are in agreement with the literature.[19]

(*S*)-2-Oxo-1,2-diphenylethyl 4-methylbenzenesulfonate (20n)

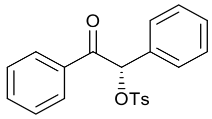

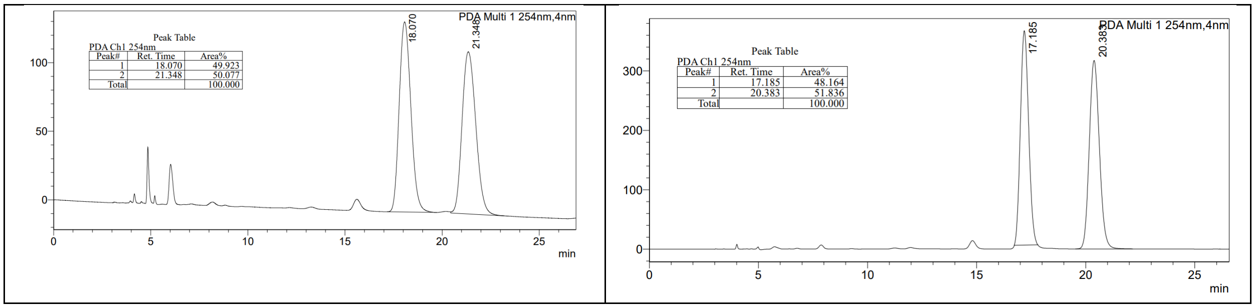

Following GP3 ,method A: chiral iodine catalyst **18b** (15 mg, 0.031 mmol, 10 mol%, 0.1 equiv.), *m*CPBA 77% pure (200 mg, 0.91 mmol, 3 equiv.), *p*-TsOH•H_2O (170 mg, 0.91 mmol, 3 equiv.), and 1,2-diphenylethan-1-one (60 mg, 0.31 mmol, 1 equiv.) in ethyl acetate (1 mL) to afford the product **20n** as a colorless solid (50 mg, 14 mmol, 45% yield).

Mp 100 °C.

Enantiomeric excess is determined by HPLC CHIRALPAK® IA: 5 μm, (*n*-hexane/*i*-PrOH = 90/10, flow rate = 1.0 mL/min, 254 nm). Minor isomer: t_R = 17.18 min, major isomer: t_R = 20.38 min, *ee* = 3%.
^{1}H NMR (500 MHz, $CDCl_3$) δ = 7.84 (dd, *J* = 8.5, 1 Hz, 2H), 7.72 (d, *J* = 8 Hz, 2H), 7.54 – 7.49 (m, 1H), 7.41 – 7.35 (m, 4H), 7.31 – 7.28 (m, 3H), 7.22 (d, *J* = 8.0 Hz, 2H), 6.67 (s, 1H), 2.39 (s, 3H) ppm.

^{13}C{^{1}H} NMR (126 MHz, $CDCl_3$) δ = 192.1, 145.0, 134.1, 133.8, 133.7, 132.8, 129.7, 129.7, 129.2, 129.1, 128.8, 128.3, 128.1, 82.4, 21.7 ppm.

The data of this compound are in agreement with the literature.[1]

(*S*)-1-Oxo-2,3-dihydro-1H-inden-2-yl 4-methylbenzenesulfonate (20p)

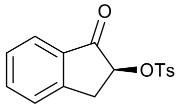

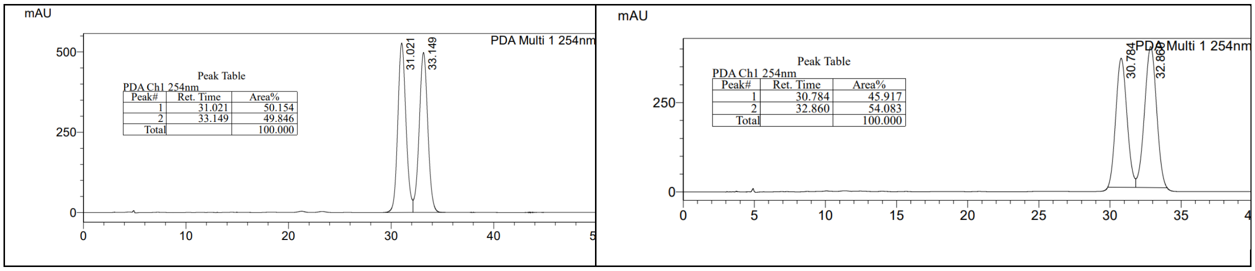

Following GP3, Method A: chiral iodine catalyst **18b** (22 mg, 0.038 mmol, 10 mol%, 0.1 equiv.), *m*CPBA 77% pure (250 mg, 1.35 mmol, 3 equiv.), *p*-TsOH•H_2O (220 mg, 1.35 mmol, 3 equiv.), and 2,3-dihydro-1H-inden-1-one (50 mg, 0.38 mmol, 1 equiv.) in ethyl acetate (1 mL) to afford the product **20p** as a colorless solid (65 mg, 0.21 mmol, 50% yield).

$[\alpha]_D^{20}$ = –4.0 (c= 1.0, $CHCl_3$).

Mp 108–109 °C.

Enantiomeric excess is determined by HPLC YMC Chiral Amylose-C S-5μm (25 cm), (*n*-hexane/*i*-PrOH = 90/10, flow rate = 1.0 mL/min, 254 nm). Minor isomer: t_R = 30.78 min, major isomer: t_R = 32.86 min, *ee* = 8%.

^{1}H NMR (500 MHz, $CDCl_3$) δ = 7.86 (d, *J* = 8.5 Hz, 2H), 7.67-7.56 (m, 2H), 7.37 – 7.31 (m, 1H), 5.06 (dd, *J* = 8.0, 5 Hz, 1H), 3.59 (dd, *J* = 17.5, 8 Hz, 1H), 3.21 (dd, *J* = 17, 4.5 Hz, 1H), 2.40 (s, 3H) ppm.

^{13}C{^{1}H} NMR (126 MHz, $CDCl_3$) δ = 197.6, 150.0, 145.3, 136.4, 133.7, 133.3, 130.0, 128.5, 128.3, 126.8, 124.8, 78.3, 34.0, 21.8 ppm.

The data of this compound are in agreement with the literature.[1]

(*S*)-1-Oxo-1,2,3,4-tetrahydronaphthalen-2-yl 4-methylbenzenesulfonate (20q)

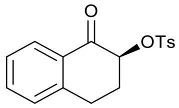

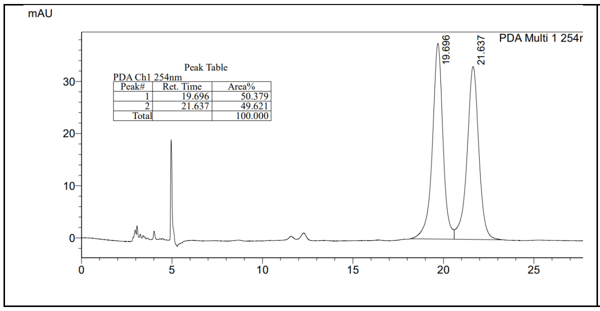

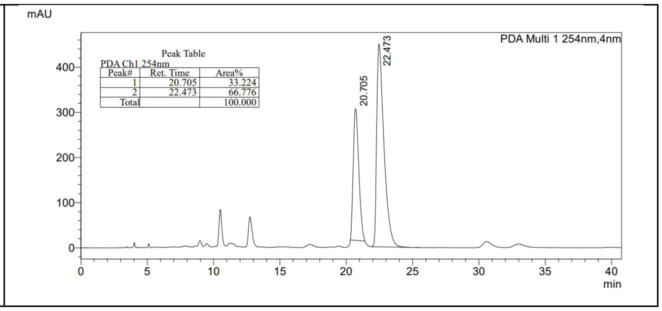

Following GP3, method A: chiral iodine catalyst **18b** (16 mg, 0.028 mmol, 10 mol%, 0.1 equiv.), *m*CPBA 77% pure (190 mg, 0.84 mmol, 3 equiv.), *p*-TsOH•H_2O (160 mg, 0.84 mmol, 3 equiv.), and 3,4-dihydronaphthalen-1(2*H*)-one (0.036 ml, 40 mg, 0.38 mmol, 1 equiv.) in ethyl acetate (1 mL) to afford the product **20q** as a colorless oil (40 mg, 0.13 mmol, 50% yield).

$[\alpha]_D^{20}$ = –13.3 (c= 0.60, $CHCl_3$).

Enantiomeric excess is determined by HPLC CHIRALPAK® IA: 5 μm, (*n*-hexane/*i*-PrOH = 90/10, flow rate = 1.0 mL/min, 254 nm). Minor isomer: t_R = 20.70 min, major isomer: t_R = 22.47 min, *ee* = 33%.

^{1}H NMR (400 MHz, $CDCl_3$) δ = 7.98 – 7.89 (m, 3H), 7.50 (td, *J* = 7.6, 1.2Hz, 1H), 7.36 (d, *J* = 8.0 Hz, 2H), 7.31 (t, *J* = 8 Hz, 1H), 7.24 (d, *J* = 7.6 Hz, 1H), 5.16 (dd, *J* = 12.4, 5.2 Hz, 1H), 3.13 (dd, *J* = 10.0, 4.0 Hz, 1H), 2.59 – 2.52 (m, 1H), 2.45 (s, 3H), 2.44 – 2.36 (m, 1H) ppm.

^{13}C{^{1}H} NMR (101 MHz, $CDCl_3$) δ = 190.6, 145.0, 143.0, 134.3, 133.8, 131.3, 129.8, 128.8, 128.2, 128.1, 127.2, 80.2, 30.7, 27.4, 21.8 ppm.

The data of this compound are in agreement with the literature.[1]

(*S*)-5-Oxo-6,7,8,9-tetrahydro-5H-benzo[7]annulen-6-yl 4-methylbenzenesulfonate (20r)

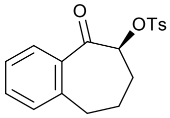

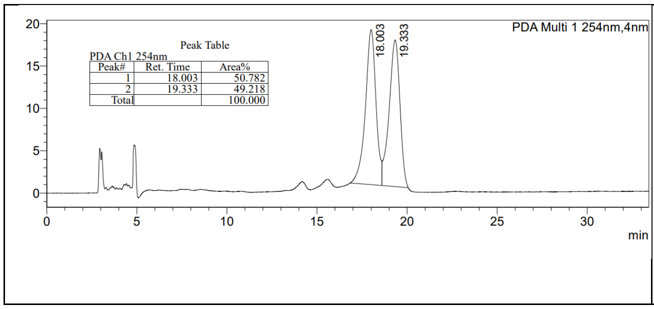

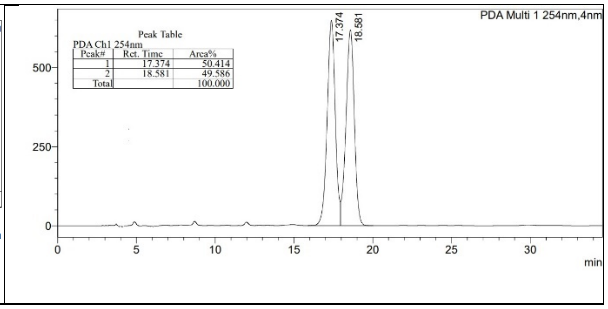

Following GP3, method A: chiral iodine catalyst **18b** (15 mg, 0.031 mmol, 10 mol%, 0.1 equiv.), *m*CPBA 77% pure (210 mg, 0.94 mmol, 3 equiv.), *p*-TsOH•H_2O (180 mg, 0.94 mmol, 3 equiv.), and 6,7,8,9-tetrahydro-5H-benzo[7]annulen-5-one (50 mg, 0.31 mmol, 1 equiv.) in ethyl acetate (1 mL) to afford the product **20r** as a colorless oil (16 mg, 0.05mmol, 16% yield).

Enantiomeric excess is determined by HPLC YMC Chiral Amylose-C S-5μm (25 cm), (*n*-hexane/*i*-PrOH = 90/10, flow rate = 1.0 mL/min, 254 nm). Minor isomer: t_R = 17.37 min, major isomer: t_R = 18.58 min, *ee* = 0%.

^{1}H NMR (500 MHz, $CDCl_3$) δ = 7.73 (d, *J* = 8 Hz, 2H), 7.44 (dd, *J* = 8, 1.5 Hz, 1H), 7.40 (td, *J* = 7.5, 1.5 Hz, 1H), 7.30 (dd, *J* = 8.5, 0.5 Hz, 2H), 7.25 (td, *J* = 7.5, 1 Hz, 1H), 7.19 (d, *J* = 7.5 Hz, 1H), 5.26 (dd, *J* = 8.5, 5.5 Hz, 1H), 3.06- 2.90 (m,21H), 2.45 (s, 3H), 2.32 – 2.24 (m, 1H), 2.15 – 2.07 (m, 1H), 2.05 – 1.96 (m, 1H), 1.93 – 1.83 (m, 1H) ppm.

^{13}C{^{1}H} NMR (126 MHz, $CDCl_3$) δ = 198.4, 145.0, 141.5, 136.4, 133.5, 132.3, 130.1, 129.9, 129.3, 128.1, 126.7, 83.1, 34.0, 30.9, 23.3, 21.8 ppm.

The data of this compound are in agreement with the literature.[22]

(*S*)-1-(Naphthalen-2-yl)-1-oxopropan-2-yl 4-methylbenzenesulfonate (20s)

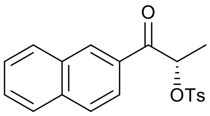

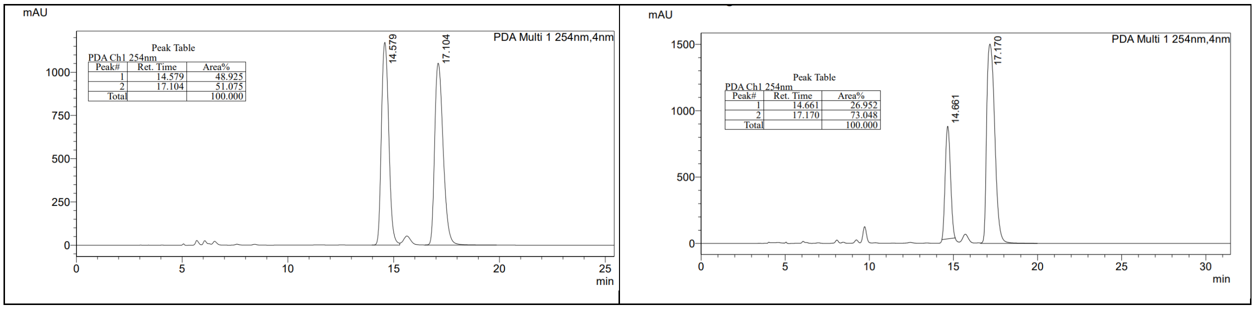

Following GP3, method A: chiral iodine catalyst **18b** (16 mg, 0.027 mmol, 10 mol%, 0.1 equiv.), *m*CPBA 77% pure (180 mg, 0.81 mmol, 3 equiv.), *p*-TsOH•H_2O (150 mg, 0.81 mmol, 3 equiv.), and 1-(naphthalen-2-yl)propan-1-one (50 mg, 0.27 mmol, 1 equiv.) in ethyl acetate (1 mL) to afford the product **20s** as a colorless solid (64 mg, 0.18 mmol, 67% yield).

$[\alpha]_D^{20}$ = –20.0 (c= 0.8, $CHCl_3$).

Enantiomeric excess is determined by HPLC CHIRALPAK® IA: 5 μm, (*n*-hexane/*i*-PrOH = 90/10, flow rate = 1.0 mL/min, 254 nm). Minor isomer: t_R = 14.66 min, major isomer: t_R = 17.17 min, *ee* = 46%.

^{1}H NMR (400 MHz, $CDCl_3$) δ = 8.40 (s, 1H), 7.96 – 7.85 (m, 4H), 7.75 (d, *J* = 8.4 Hz, 2H), 7.66-7.54 (m, 2H), 7.20 (d, *J* = 8.0 Hz, 1H), 5.94 (q, *J* = 6.8 Hz, 1H), 2.35 (s, 1H), 1.67 (d, *J* = 7.2 Hz, 1H) ppm.

^{13}C{^{1}H} NMR (101 MHz, $CDCl_3$) δ = 194.8, 145.1, 135.9, 133.6, 132.4, 131.1, 130.8, 129.8, 129.8, 129.1, 128.8, 128.0, 127.9, 127.1, 124.1, 77.5, 21.7, 19.0 ppm.

The data of this compound are in agreement with the literature.[1]

(*S*)-1-(Furan-2-yl)-1-oxopropan-2-yl 4-methylbenzenesulfonate (20t)

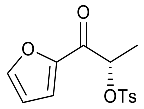

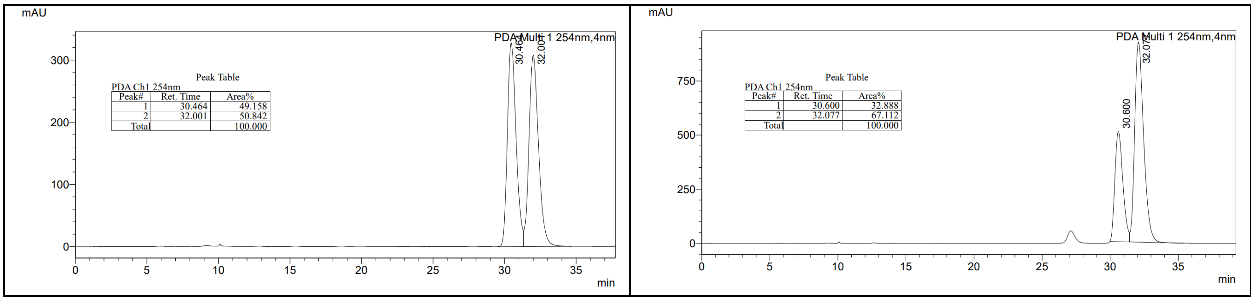

Following GP3, method A: chiral iodine catalyst **18b** (24 mg, 0.04 mmol, 10 mol%, 0.1 equiv.), *m*CPBA 77% pure (270 mg, 1.20 mmol, 3 equiv.), *p*-TsOH•H_2O (230 mg, 1.20 mmol, 3 equiv.), and 1-(furan-2-yl)propan-1-one (50 mg, 0.4 mmol, 1 equiv.) in ethyl acetate (1 mL) to afford the product **20t** as a colorless solid (40 mg, 0.14 mmol, 37% yield).

$[\alpha]_D^{20}$ = –10.0 (c= 0.6, $CHCl_3$).

Enantiomeric excess is determined by HPLC CHIRALPAK® IA: 5 μm, (*n*-hexane/*i*-PrOH = 90/10, flow rate = 0.5 mL/min, 254 nm). Minor isomer: t_R = 30.60 min, major isomer: t_R = 32.07 min, *ee* = 34%.

^{1}H NMR (500 MHz, $CDCl_3$) δ = 7.78 (d, *J* = 8.5 Hz, 2H), 7.61 (d, *J* = 1 Hz, 1H), 7.36 (d, *J* = 4 Hz, 1H), 7.30 (d, *J* = 8 Hz, 2H), 6.57 (dd, *J* = 3.5, 1.5 Hz, 1H), 5.51 (q, *J* = 7 Hz, 1H), 2.42 (s, 3H), 1.55 (d, *J* = 7 Hz, 3H) ppm.

^{13}C{^{1}H} NMR (126 MHz, $CDCl_3$) δ = 183.5, 149.7, 147.5, 145.2, 133.5, 129.9, 128.1, 120.2, 112.8, 77.5, 21.8, 18.5 ppm.

The data of this compound are in agreement with the literature.[1]

(*S*)-1-Oxo-1-(thiophen-2-yl)propan-2-yl 4-methylbenzenesulfonate (20u)

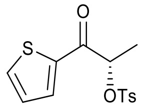

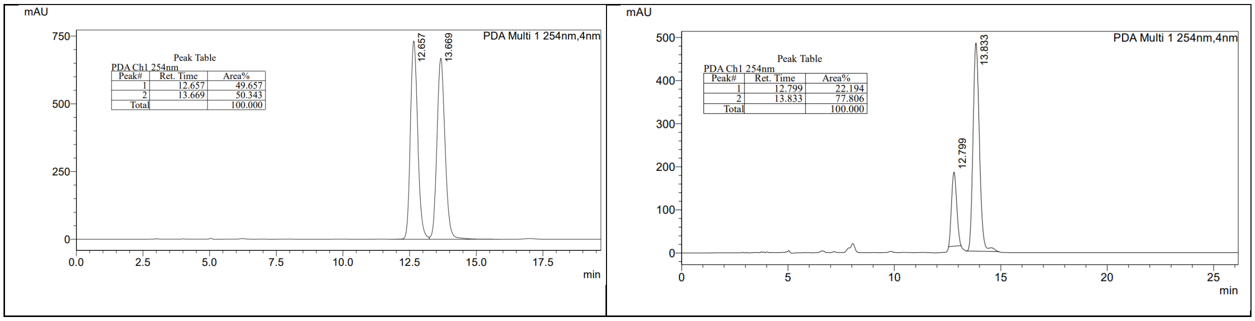

Following GP3, method A: chiral iodine catalyst **18b** (21 mg, 0.035 mmol, 10 mol%, 0.1 equiv.), *m*CPBA 77% pure (240 mg, 1.07 mmol, 3 equiv.), *p*-TsOH•H_2O (210 mg, 1.07 mmol, 3 equiv.), and 1-(thiophen-2-yl)propan-1-one (50 mg, 0.35 mmol, 1 equiv.) in ethyl acetate (1 mL) to afford the product **20u** as a colorless solid (50 mg, 0.16 mmol, 45% yield).

$[\alpha]_D^{20}$ = –12.5 (c = 0.8, $CHCl_3$).

Enantiomeric excess is determined by HPLC CHIRALPAK® IA: 5 *µ*m, (*n*-hexane/*i*-PrOH = 90/10, flow rate = 1.0 mL/min, 254 nm). Minor isomer: t_R = 12.79 min, major isomer: t_R = 13.83 min, *ee* = 56%.

^{1}H NMR (500 MHz, $CDCl_3$) δ = 7.88 (dd, *J* = 4, 1 Hz, 1H), 7.76 (d, *J* = 8.5 Hz, 2H), 7.70 (dd, *J* = 5, 1 Hz, 1H), 7.28 (d, *J* = 8.5 Hz, 2H), 7.15 (dd, *J* = 4.5, 4 Hz, 1H), 5.45 (q, *J* = 6.5 Hz, 1H), 2.42 (s, 3H), 1.60 (d, *J* = 7 Hz, 3H) ppm.

^{13}C{^{1}H} NMR (126 MHz, $CDCl_3$) δ = 188.1, 145.3, 140.0, 135.3, 133.9, 133.3, 129.9, 128.5, 128.2, 78.6, 21.8, 19.2 ppm.

The data of this compound are in agreement with the literature.[1]

(*S*)-1-Oxo-1-phenylpropan-2-yl benzenesulfonate (20v)

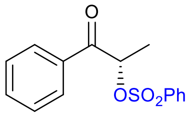

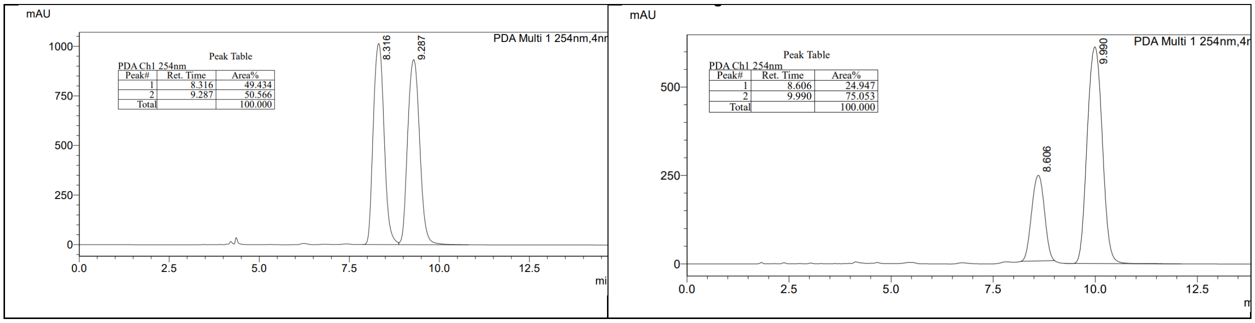

Following GP3, method A: chiral iodine catalyst **18b** (22 mg, 0.037 mmol, 10 mol%, 0.1 equiv.), *m*CPBA 77% pure (250 mg, 1.12 mmol, 3 equiv.), phSO_2OH (180 mg, 1.12 mmol, 3 equiv.), and propiophenone (50 mg, 0.37 mmol, 1 equiv.) in ethyl acetate (1 mL) to afford the product **20v** as a colorless solid (88 mg, 0.30 mmol, 88% yield).

$[\alpha]_D^{20}$ = –11.25 (c= 1.60, $CHCl_3$).

Enantiomeric excess is determined by HPLC CHIRALPAK® IA: 5 μm, (*n*-hexane/*i*-PrOH = 80/20, flow rate = 1.0 mL/min, 254 nm). Minor isomer: t_R = 8.606 min, major isomer: t_R = 9.99 min, *ee* = 50 %.

^{1}H NMR (500 MHz, $CDCl_3$) δ = 7.90 – 7.85 (m, 4H), 7.63 – 7.55 (m, 2H), 7.50 – 7.42 (m, 4H), 5.83 (q, *J* = 7 Hz, 1H), 1.60 (d, *J* = 7 Hz, 3H) ppm.

^{13}C{^{1}H} NMR (126 MHz, $CDCl_3$) δ = 194.7, 136.6, 134.0, 134.0, 133.7, 129.2, 128.9, 128.8, 127.9, 77.6, 18.8 ppm.

The data of this compound are in agreement with the literature.[1]

(*S*)-1-Oxo-1-phenylpropan-2-yl 2,4,6-trimethylbenzenesulfonate (20w)

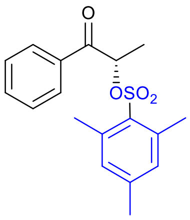

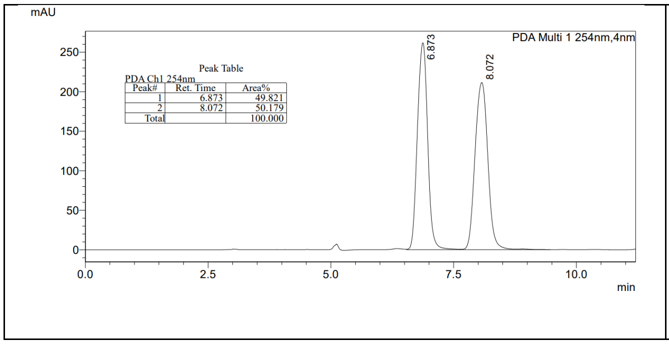

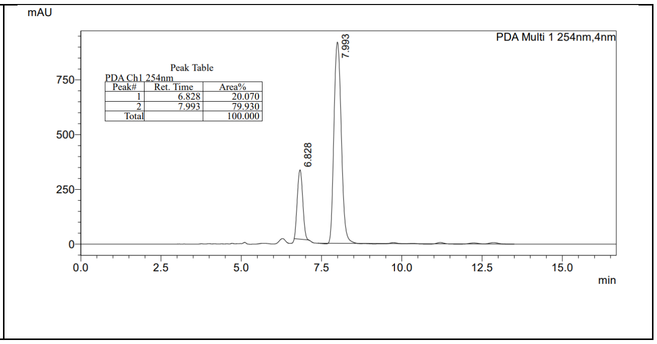

Following GP3, method A: chiral iodine catalyst **18b** (22 mg, 0.037 mmol, 10 mol%, 0.1 equiv.), *m*CPBA 77% pure (250 mg, 1.12 mmol, 3 equiv.), 2-mesitylenesulfonic acid dihydrate ($MesSO_2OH•2H_2O$) (260 mg, 1.12 mmol, 3 equiv.), and propiophenone (50 mg, 0.37 mmol, 1 equiv.) in ethyl acetate (1 mL) to afford the product **20w** as a colorless solid (90 mg, 0.27 mmol, 75% yield).

$[\alpha]_D^{20}$ = –35.0 (c= 0.4, $CHCl_3$).

Enantiomeric excess is determined by HPLC CHIRALPAK® IA: 5 μm, (*n*-hexane/*i*-PrOH = 90/10, flow rate = 1.0 mL/min, 254 nm). Minor isomer: t_R = 6.82 min, major isomer: t_R = 7.99 min, *ee* = 59%.
^{1}H NMR (500 MHz, $CDCl_3$) δ = 7.87 (dd, *J* = 8.5, 1 Hz, 2H), 7.62 – 7.54 (m, 1H), 7.44 (t, *J* = 8.5 Hz, 2H), 6.90 (s, 2H), 5.73 (q, *J* = 7 Hz, 1H), 2.59 (s, 6H), 2.27 (s, 3H), 1.59 (d, *J* = 7 Hz, 3H) ppm.

$^{13}C\{^1H\}$ NMR (126 MHz, $CDCl_3$) δ = 194.8, 143.6, 140.0, 133.9, 131.8, 131.4, 128.8, 128.8, 76.5, 22.7, 21.1, 18.7 ppm.

The data of this compound are in agreement with the literature.[23]

(*S*)-1-Oxo-1-phenylpropan-2-yl methanesulfonate (20x)

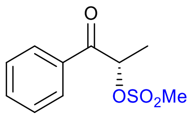

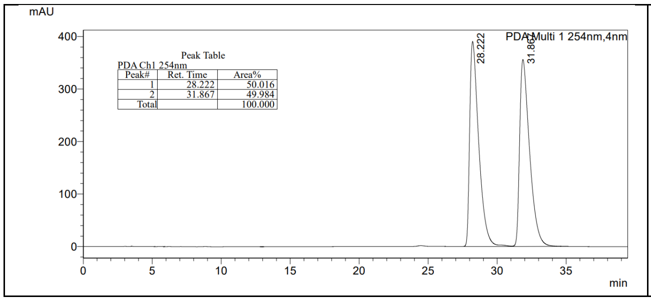

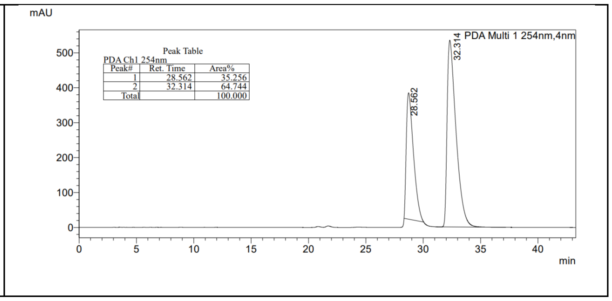

Following **GP 3** Method **A**: chiral iodine catalyst **18b** (22 mg, 0.037 mmol, 10 mol%, 0.1 equiv.), *m*CPBA 77% pure (250 mg, 1.12 mmol, 3 equiv.), $MeSO_2OH$ (110 mg, 1.12 mmol, 3 equiv.), and propiophenone (50 mg, 0.37 mmol, 1 equiv.) in ethyl acetate (1 mL) to afford the product **20x** as a colorless solid (70 mg, 0.31 mmol, 87% yield).

$[\alpha]_D^{20}$ = –20.0 (c= 1.1, $CHCl_3$).

Enantiomeric excess is determined by HPLC CHIRALPAK® IA: 5 μm, (*n*-hexane/*i*-PrOH = 98/2, flow rate = 1.0 mL/min, 254 nm). Minor isomer: t_R = 28.56 min, major isomer: t_R = 32.31 min, *ee* = 30%.

^{1}H NMR (500 MHz, $CDCl_3$) δ = 7.94 (dd, *J* = 8, 1.5 Hz, 2H), 7.66 – 7.60 (m, 1H), 7.54 – 7.48 (m, 2H), 6.05 (q, *J* = 7.0 Hz, 1H), 3.13 (s, 3H), 1.66 (d, *J* = 7.0 Hz, 3H) ppm.

^{13}C{^{1}H} NMR (126 MHz, $CDCl_3$) δ = 195.4, 134.3, 133.8, 129.1, 128.7, 77.2, 39.5, 18.8 ppm.

The data of this compound are in agreement with the literature.[1]

(*S*)-1-Oxo-1-phenylpropan-2-yl 4-chlorobenzenesulfonate (20y)

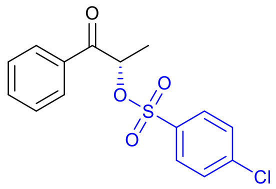

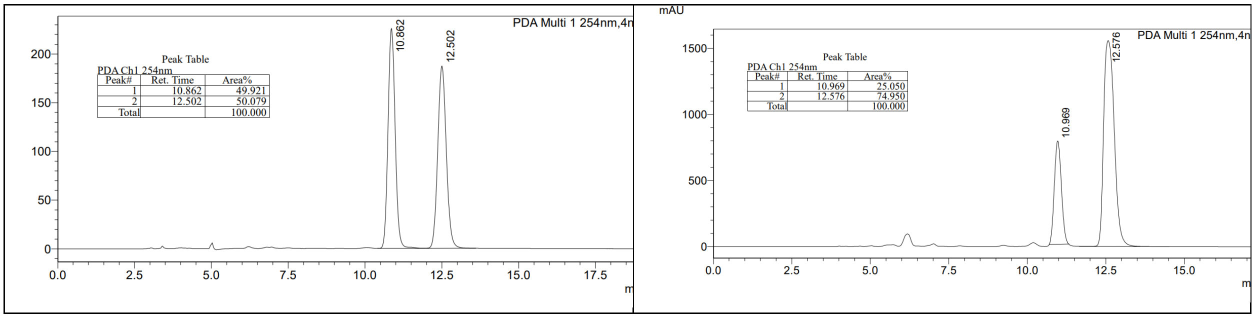

Following GP3, method A: chiral iodine catalyst **18b** (22 mg, 0.037 mmol, 10 mol%, 0.1 equiv.), *m*CPBA 77% pure (250 mg, 1.12 mmol, 3 equiv.), 4-chlorobenzenesulfonic acid (220 mg, 1.12 mmol, 3 equiv.), and propiophenone (50 mg, 0.37 mmol, 1 equiv.) in ethyl acetate (1 mL) to afford the product **20y** as a colorless oil (36 mg, 0.11 mmol, 30% yield).

$[\alpha]_D^{20}$ = –30.0 (c= 0.4, $CHCl_3$).

Enantiomeric excess is determined by HPLC CHIRALPAK® IA: 5 μm, (*n*-hexane/*i*-PrOH = 90/10, flow rate = 1.0 mL/min, 254 nm). Minor isomer: t_R = 10.96 min, major isomer: t_R = 12.57 min, *ee* = 50%.

^{1}H NMR (500 MHz, $CDCl_3$) δ = 7.86 (dd, 8.5, 1.5 Hz, 2H), 7.81 (d, 8.5 Hz, 2H), 7.62 – 7.58 (m, 1H), 7.50 – 7.43 (m, 4H), 5.86 (q, *J* = 7.0 Hz, 1H), 1.63 (d, *J* = 7.0 Hz, 3H) ppm.

^{13}C{^{1}H} NMR (126 MHz, $CDCl_3$) δ = 194.7, 140.8, 135.1, 134.1, 133.7, 129.6, 129.5, 129.0, 128.8, 77.9, 18.9 ppm.

IR (neat) $\tilde{\nu}$ =2315, 2320, 1697, 1363, 1188, 921, 682 cm^{-1}.

HRMS (ESI-TOF) m/z: $[M+Na]^+$ calcd for $C_{15}H_{13}O_4SClNa$ 347.0121; found 347.0127.

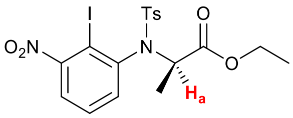

Figure S1: structural analysis of 13j

A) ^{1}H NMR spectroscopy of **13j** in different deuterated solvents at room temperature

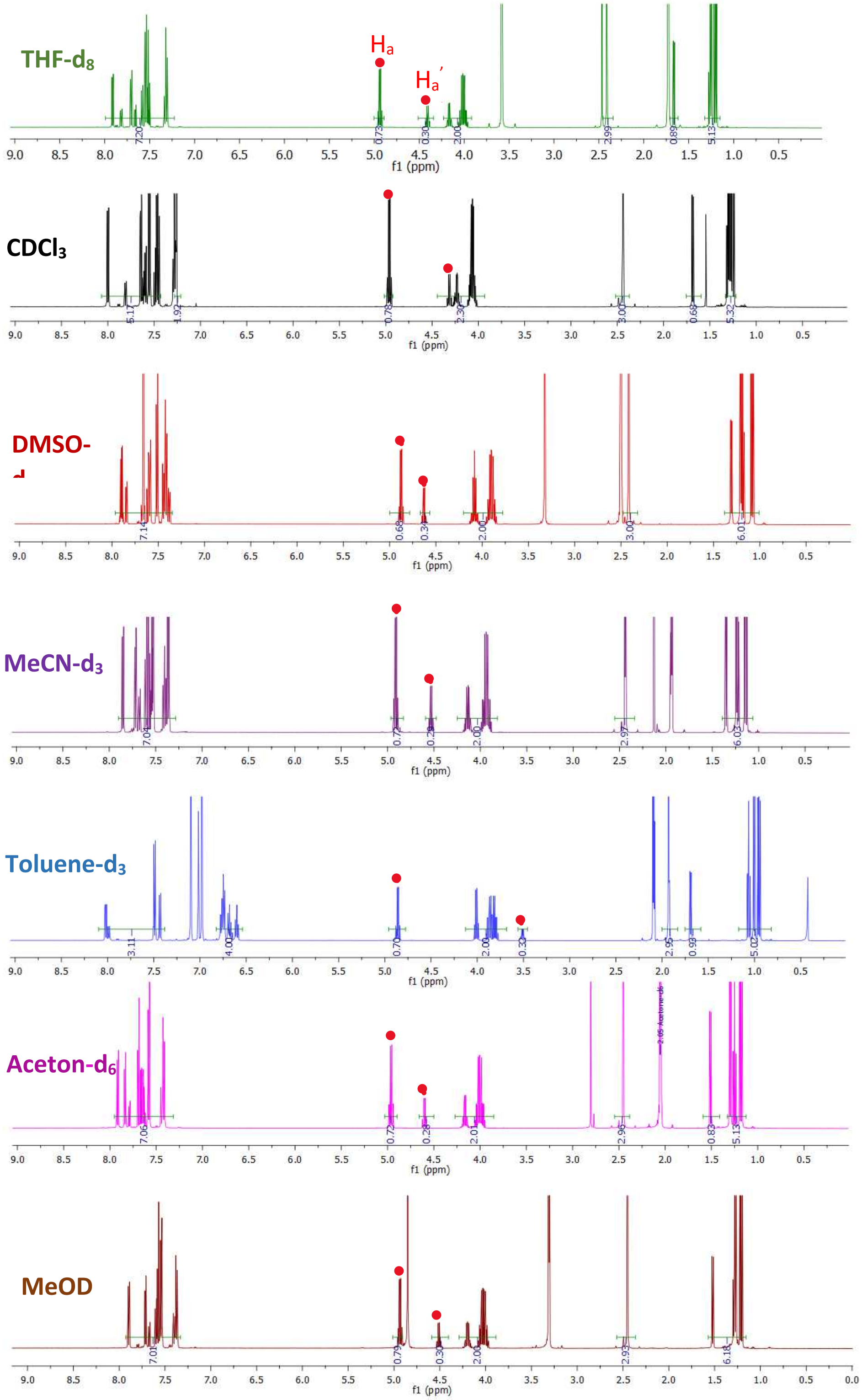

B) ^{1}H NMR spectroscopy of **13j** at different temperatures in MeCN-d_3 solvent. The ratio between (H_a : H_a\`) and peaks shifts is shown in spectra.

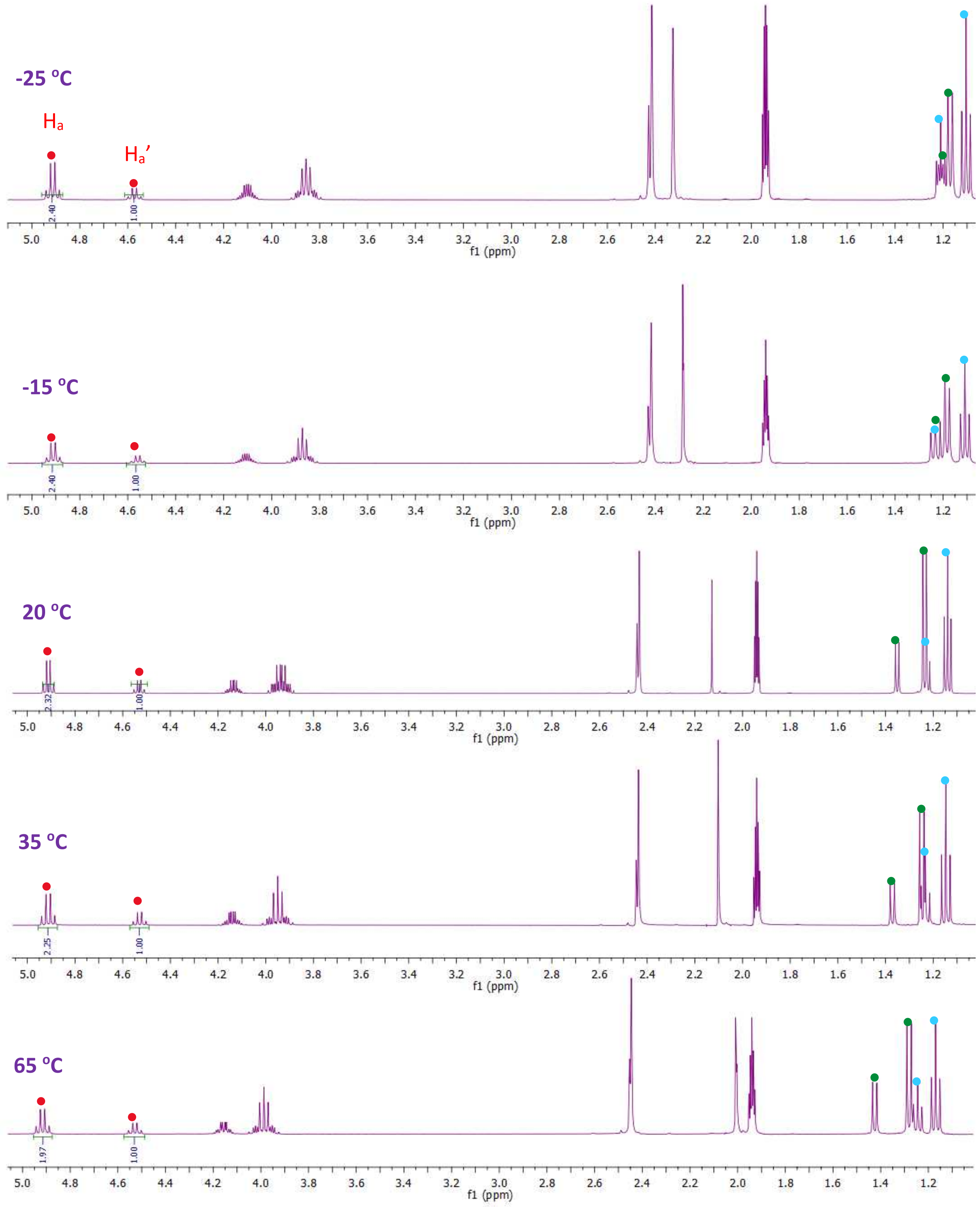

C) Compasion between ^{13}C NMR and ^{13}C SSNMR spectroscopy of **13j**

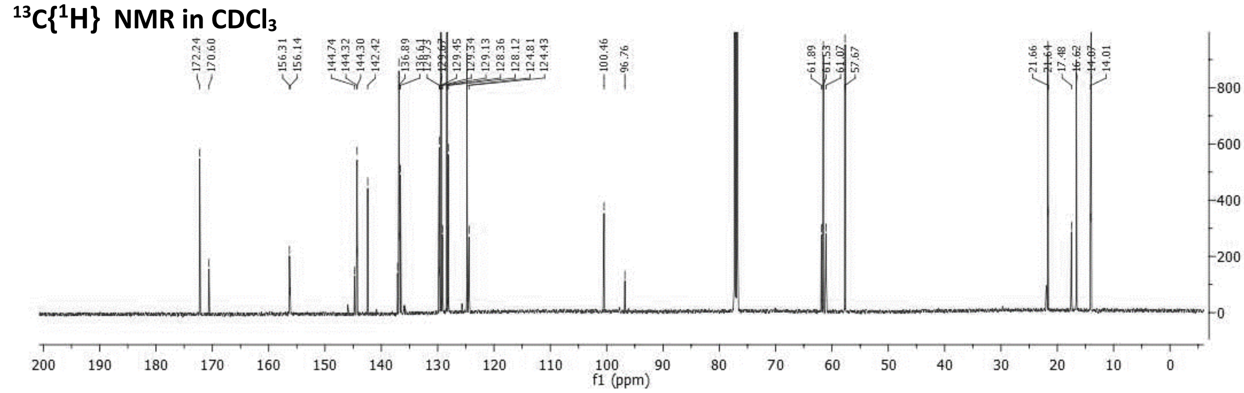

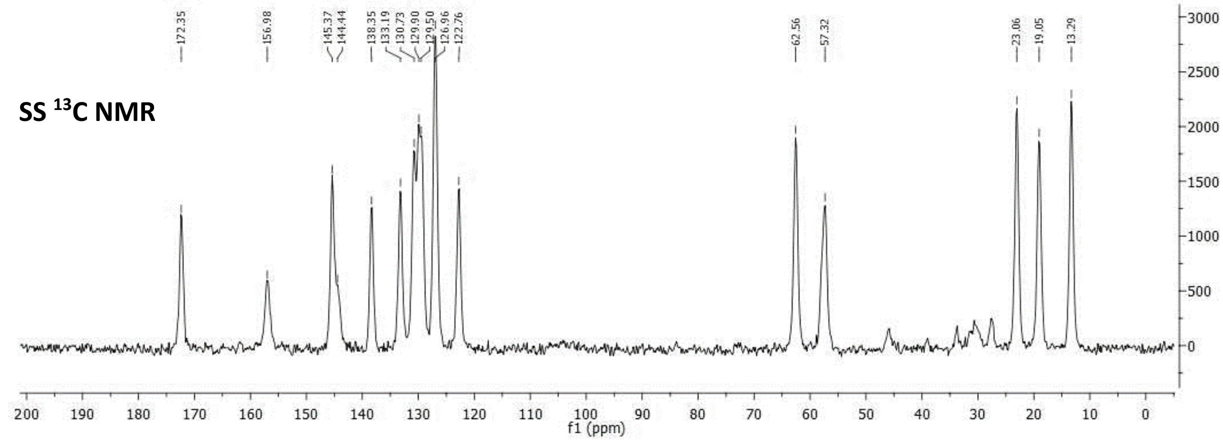

Cyclic voltammograms (CV):

The cyclic voltammogram studies were performed using an Orygalys OGF500 Potentiostat/ Galvanostat with OGFPWR power supply.

Figure S2 demonstrates electrochemical cell that was used for performing CV experiments. The electrodes and glass solution reservoir were polished with methanol once following with acetonitrile two times and then dried before running the samples. The system and solvent were deoxygenated using nitrogen gas.

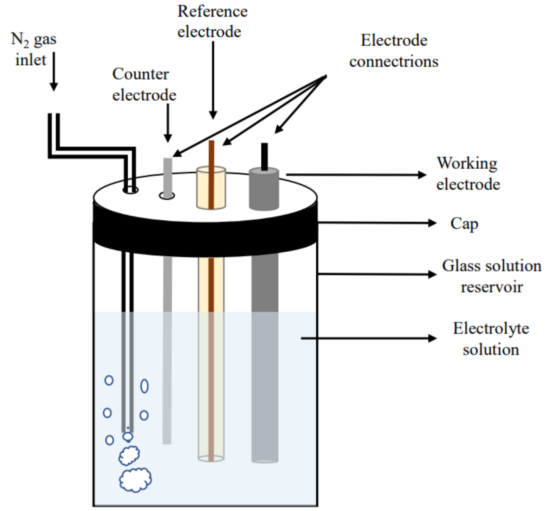

Figure S2: Diagram presentation of an electrochemical cell for CV experiments

Figures S3: Oxidative cyclic voltammograms of the catalysts **7**, **13a**, **13h**, **13i**, **18a**, **18c**, **19a**, **19b** and **19c.**

All experiments were performed at room temperature in acetonitrile solvent. The concentration of catalysts **7**, **13a**, **13h**, **13i**, **18a**, **18c**, **19a**, **19b** and **19c** is (4 mM). Electrolyte concentration is (0.1 M) of Bu_4NClO_4. Working electrode: glassy carbon (3 mm diameter). Counter electrode: platinum wire. Reference electrode: Ag/AgCl in 3M NaCl. Scan rate: 100 mV/s.

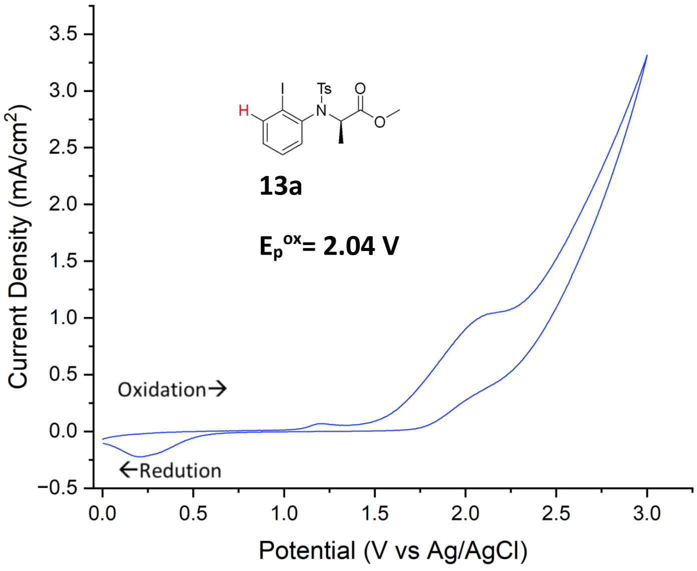

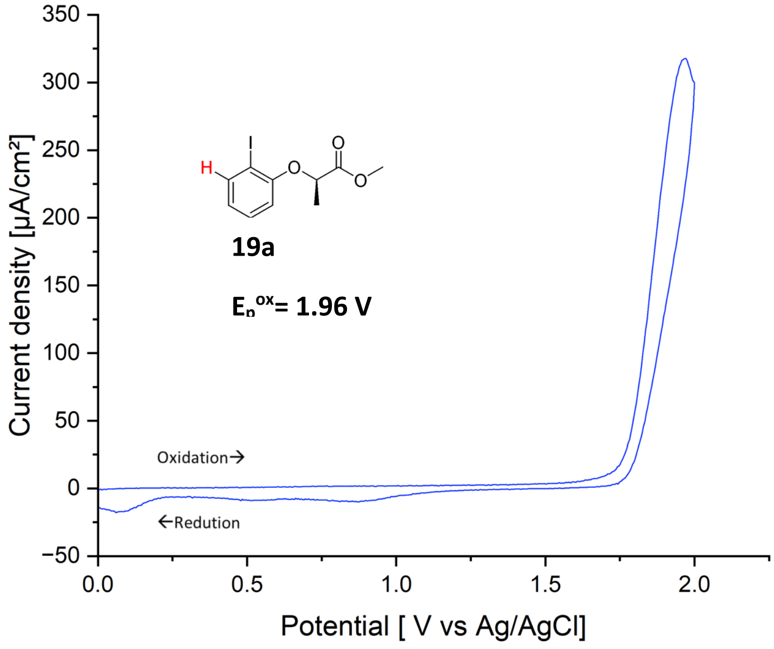

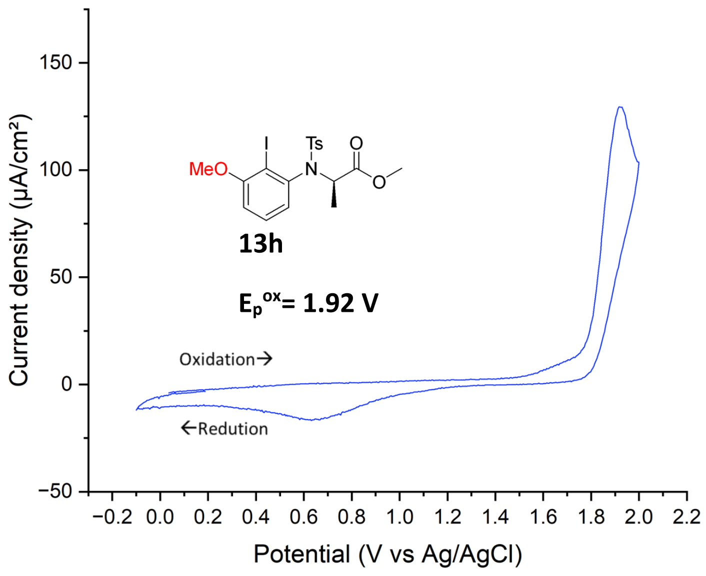
MeO
I
Ts
N
O
O
13h
E_p^{ox}= 1.92 V
Oxidation→
←Redution
Current density (μA/cm²)
Potential (V vs Ag/AgCl)
150
100
50
0
−50
−0.2
0.0
0.2
0.4
0.6
0.8
1.0
1.2
1.4
1.6
1.8
2.0
2.2

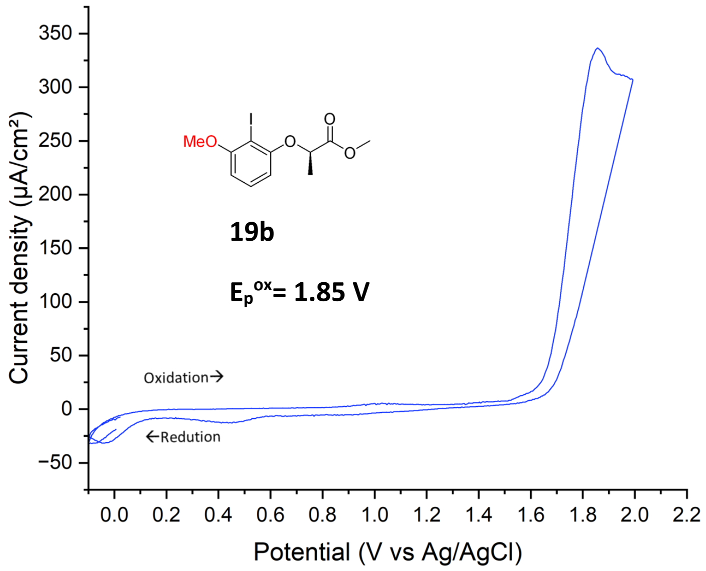
MeO
I
O
O
O
19b
E_p^{ox}= 1.85 V
Oxidation→
←Redution
Current density (μA/cm²)
Potential (V vs Ag/AgCl)
350
300
250
200
150
100
50
0
−50
0.0
0.2
0.4
0.6
0.8
1.0
1.2
1.4
1.6
1.8
2.0
2.2

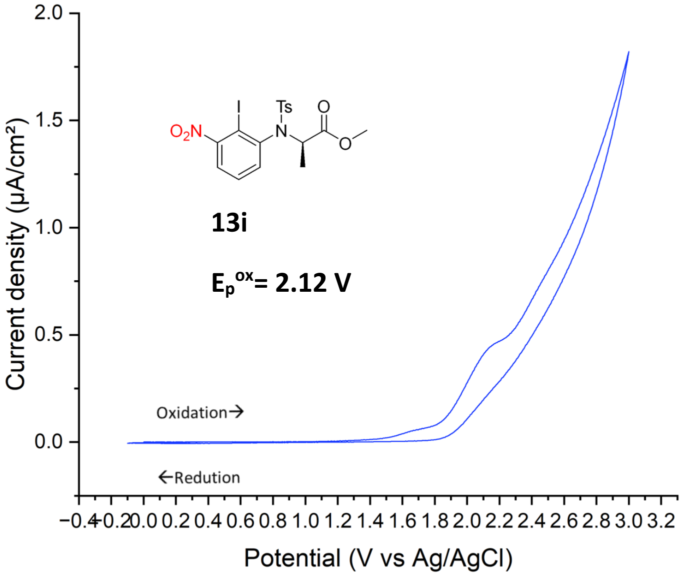

2.0
1.5
1.0
0.5
0.0
Current density (μA/cm²)
O2N
I
Ts
N
O
O
13i
Ep^ox= 2.12 V
Oxidation→
←Redution
−0.4 −0.2 0.0 0.2 0.4 0.6 0.8 1.0 1.2 1.4 1.6 1.8 2.0 2.2 2.4 2.6 2.8 3.0 3.2
Potential (V vs Ag/AgCl)

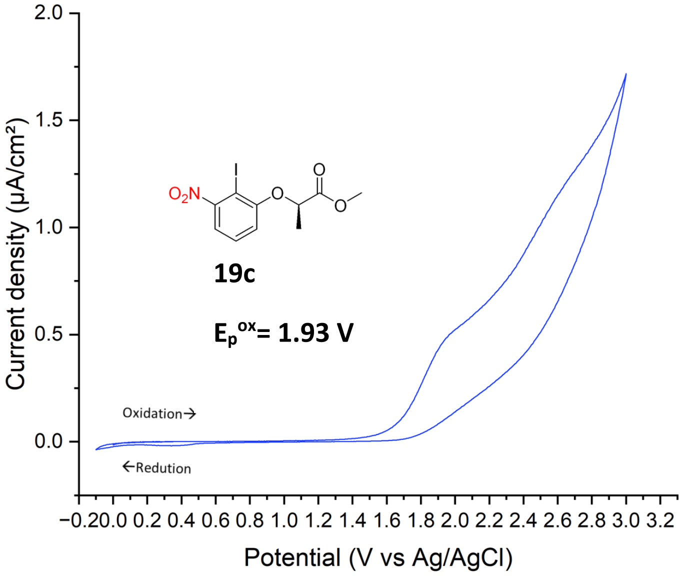

2.0
1.5
1.0
0.5
0.0
Current density (μA/cm²)
O2N
I
O
O
O
19c
Ep^ox= 1.93 V
Oxidation→
←Redution
−0.2 0.0 0.2 0.4 0.6 0.8 1.0 1.2 1.4 1.6 1.8 2.0 2.2 2.4 2.6 2.8 3.0 3.2
Potential (V vs Ag/AgCl)

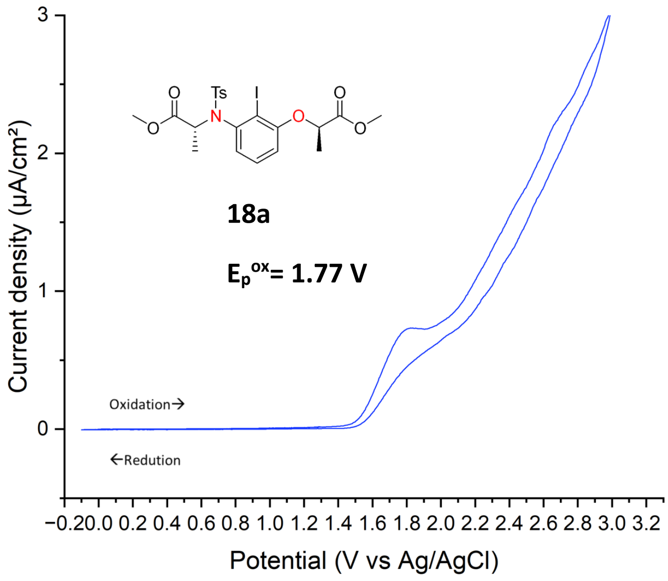
Ts
I
O
O
N
O
O
O
18a
E_p^{ox}= 1.77 V
Oxidation→
←Redution
Current density (μA/cm²)
Potential (V vs Ag/AgCl)

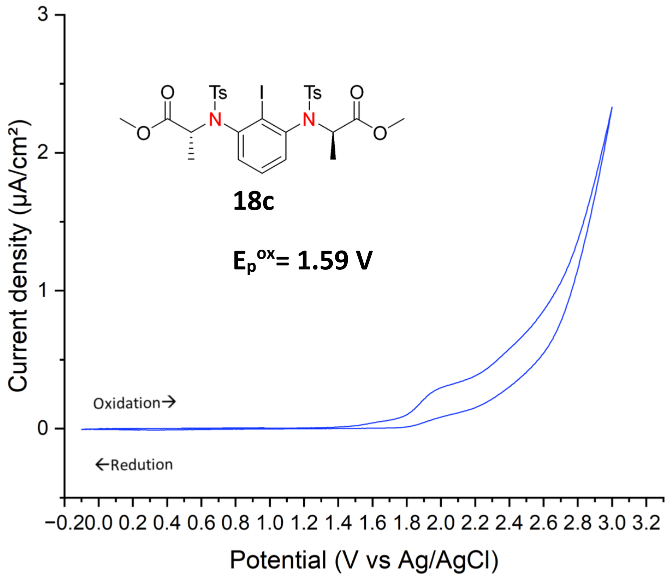
Ts
I
Ts
O
O
N
N
O
O
18c
E_p^{ox}= 1.59 V
Oxidation→
←Redution
Current density (μA/cm²)
Potential (V vs Ag/AgCl)

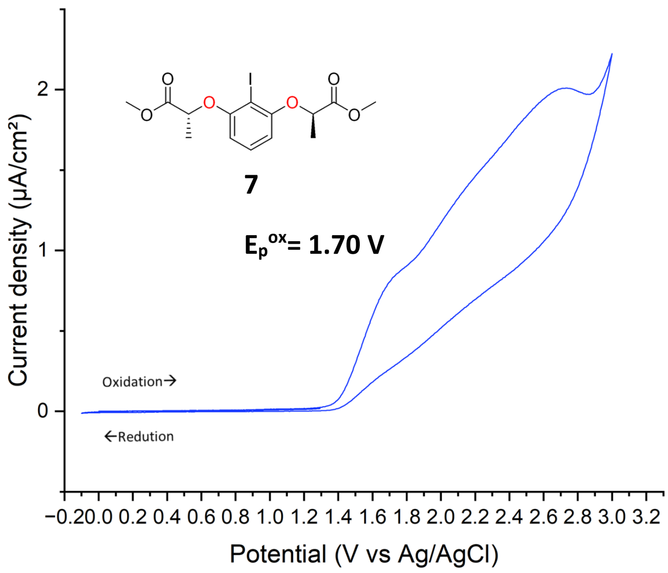
7
E_p^{ox}= 1.70 V
Oxidation→
←Redution
Current density (μA/cm²)
Potential (V vs Ag/AgCl)

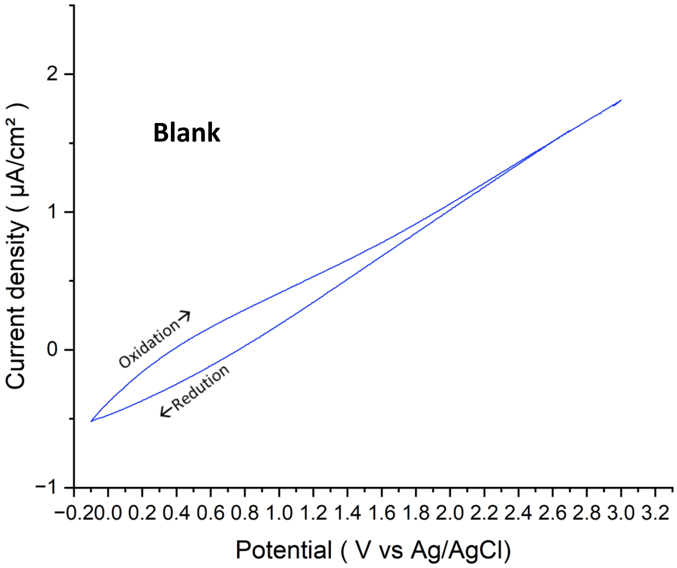
Blank
Oxidation→
←Redution
Current density (μA/cm²)
Potential (V vs Ag/AgCl)

X-ray data:

Crystalyzation method: In clean vial 0.5 g of compound was dissolved in (25 mL) ethyl acetate and left for slow evaporation at room temperature. The rate of evaporation was controled by

covering the vial with parafilm and making few small holes. For growing the crystals with good quality, the vial was placed in a way that you can check without disturbing.

Table S1: Crystal data and structure refinement for **13a** (CCDC 2247504).

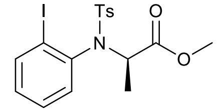

Empirical formula	$C_{17}H_{18}INO_4S$	
Formula weight	459.28	
Temperature	293(2) K	
Wavelength	0.71073 Å	
Crystal system	Orthorhombic	
Space group	$P2_12_12_1$	
Unit cell dimensions	a = 7.9892(4) Å	a= 90°.
	b = 9.9403(4) Å	b= 90°.
	c = 23.6563(10) Å	g = 90°.
Volume	1878.67(14) $Å^3$	
Z	4	
Density (calculated)	1.624 Mg/m^3	
Absorption coefficient	1.835 mm^{-1}	
F(000)	912	
Crystal size	0.430 x 0.280 x 0.250 mm^3	
Theta range for data collection	3.298 to 29.648°.	
Index ranges	-8<=h<=11, -12<=k<=13, -31<=l<=30	
Reflections collected	17863	
Independent reflections	4643 [R(int) = 0.0350]	
Completeness to theta = 25.242°	99.6 %	
Absorption correction	Gaussian	
Max. and min. transmission	1.000 and 0.375	
Refinement method	Full-matrix least-squares on F^2	
Data / restraints / parameters	4643 / 0 / 221	
Goodness-of-fit on F^2	1.048	
Final R indices [I>2sigma(I)]	R1 = 0.0430, wR2 = 0.0787	
R indices (all data)	R1 = 0.0721, wR2 = 0.0909	
Absolute structure parameter	-0.029(10)	
Extinction coefficient	0.0122(7)	

Largest diff. peak and hole 0.790 and -0.756 e.Å^{-3}

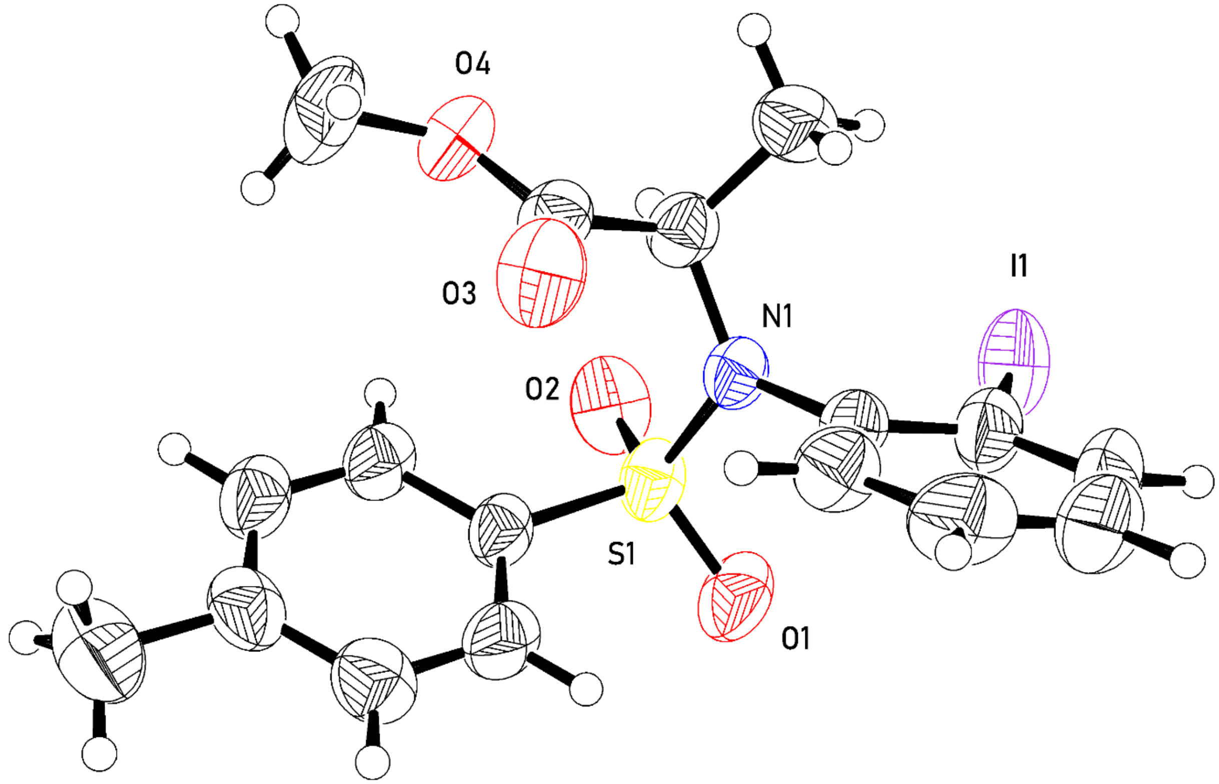

Figure S4: Solid state structure for **13a**. The ellipsoid probability level for the figure is 50%.

Table S2: Crystal data and structure refinement for **13c** (CCDC 2247506).

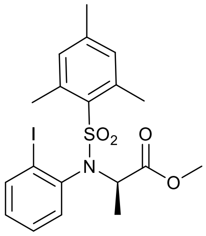

Empirical formula	$C_{19}H_{22}INO_4S$	
Formula weight	487.33	
Temperature	293(2) K	
Wavelength	0.71073 Å	
Crystal system	Orthorhombic	
Space group	$P2_12_12_1$	
Unit cell dimensions	a = 8.0228(2) Å	a= 90°.
	b = 8.9149(2) Å	b= 90°.
	c = 28.8375(9) Å	g = 90°.
Volume	2062.53(9) $Å^3$	
Z	4	
Density (calculated)	1.569 Mg/m^3	
Absorption coefficient	1.676 mm^{-1}	
F(000)	976	
Crystal size	0.290 x 0.230 x 0.160 mm^3	
Theta range for data collection	3.308 to 29.467°.	
Index ranges	-10<=h<=10, -12<=k<=11, -37<=l<=39	
Reflections collected	16605	
Independent reflections	5015 [R(int) = 0.0275]	
Completeness to theta = 25.242°	99.7 %	
Absorption correction	Gaussian	
Max. and min. transmission	1.000 and 0.513	
Refinement method	Full-matrix least-squares on F^2	
Data / restraints / parameters	5015 / 0 / 240	
Goodness-of-fit on F^2	1.040	
Final R indices [I>2sigma(I)]	R1 = 0.0288, wR2 = 0.0531	
R indices (all data)	R1 = 0.0375, wR2 = 0.0564	
Absolute structure parameter	-0.026(9)	
Largest diff. peak and hole	0.652 and -0.573 e.$Å^{-3}$	

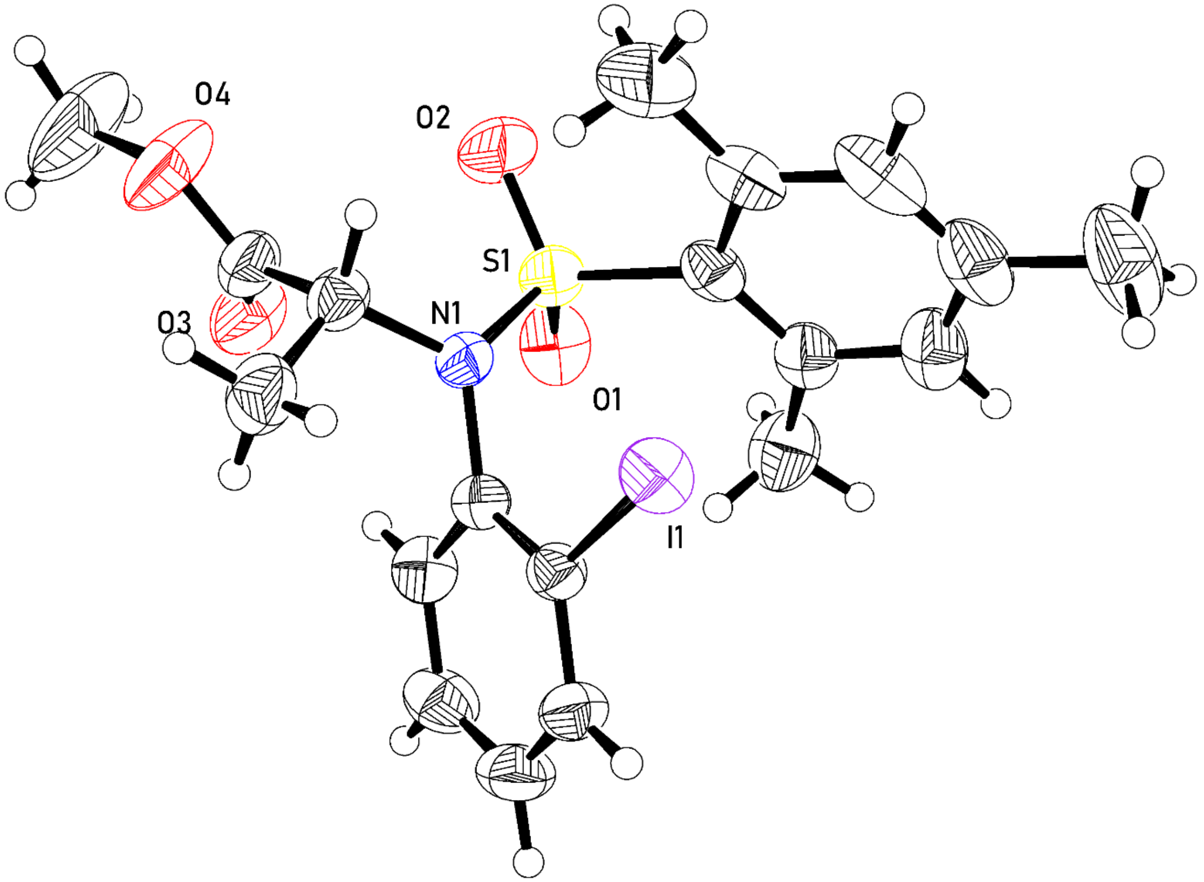

Figure S5: Solid state structure for **13c**. The ellipsoid probability level for the figure is 50%.

Table S3: Crystal data and structure refinement for **13d** (CCDC 2247510).

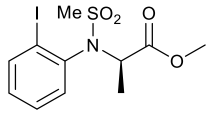

Empirical formula	$C_{11}H_{14}INO_4S$	
Formula weight	383.19	
Temperature	293(2) K	
Wavelength	0.71073 Å	
Crystal system	Monoclinic	
Space group	$P2_1/c$	
Unit cell dimensions	a = 8.1932(3) Å	a= 90°.
	b = 8.4140(3) Å	b= 92.915(4)°.
	c = 20.3265(10) Å	g = 90°.
Volume	1399.45(10) $Å^3$	
Z	4	
Density (calculated)	1.819 Mg/m^3	
Absorption coefficient	2.443 mm^{-1}	
F(000)	752	
Crystal size	0.300 x 0.280 x 0.210 mm^3	
Theta range for data collection	3.276 to 29.486°.	
Index ranges	-10<=h<=11, -11<=k<=11, -26<=l<=17	
Reflections collected	13169	
Independent reflections	3508 [R(int) = 0.0369]	
Completeness to theta = 25.242°	99.8 %	
Absorption correction	Gaussian	
Max. and min. transmission	1.000 and 0.603	
Refinement method	Full-matrix least-squares on F^2	
Data / restraints / parameters	3508 / 0 / 166	
Goodness-of-fit on F^2	1.089	
Final R indices [I>2sigma(I)]	R1 = 0.0348, wR2 = 0.0622	
R indices (all data)	R1 = 0.0605, wR2 = 0.0746	
Largest diff. peak and hole	0.779 and -1.027 $e.Å^{-3}$	

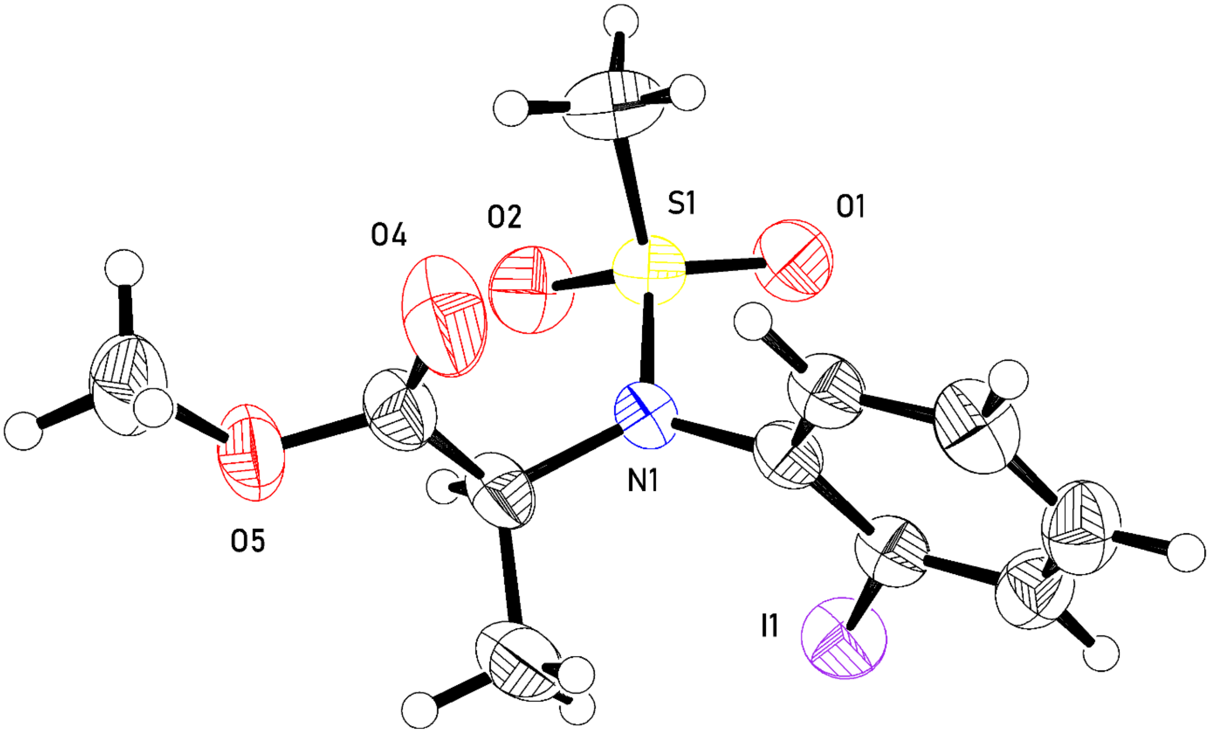

Figure S6: Solid state structure for **13d**. The ellipsoid probability level for the figure is 50%.

Table S4: Crystal data and structure refinement for **13f** (CCDC 2247505).

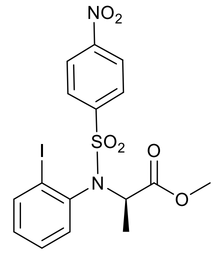

Empirical formula	$C_{16}H_{15}IN_2O_6S$	
Formula weight	490.26	
Temperature	293(2) K	
Wavelength	0.71073 Å	
Crystal system	Monoclinic	
Space group	$P2_1$	
Unit cell dimensions	a = 17.8610(6) Å	a= 90°.
	b = 11.1096(3) Å	b= 91.254(3)°.
	c = 18.8493(5) Å	g = 90°.
Volume	3739.34(19) $Å^3$	
Z	8	
Density (calculated)	1.742 Mg/m^3	
Absorption coefficient	1.859 mm^{-1}	
F(000)	1936	
Crystal size	0.270 x 0.250 x 0.160 mm^3	
Theta range for data collection	3.462 to 29.673°.	
Index ranges	-24<=h<=24, -15<=k<=14, -26<=l<=24	
Reflections collected	34138	
Independent reflections	17491 [R(int) = 0.0339]	
Completeness to theta = 25.242°	99.7 %	
Absorption correction	Gaussian	
Max. and min. transmission	1.000 and 0.719	
Refinement method	Full-matrix least-squares on F^2	
Data / restraints / parameters	17491 / 37 / 933	
Goodness-of-fit on F^2	1.023	
Final R indices [I>2sigma(I)]	R1 = 0.0538, wR2 = 0.1166	
R indices (all data)	R1 = 0.0841, wR2 = 0.1370	
Absolute structure parameter	-0.013(10)	
Largest diff. peak and hole	2.114 and -1.725 e.$Å^{-3}$	

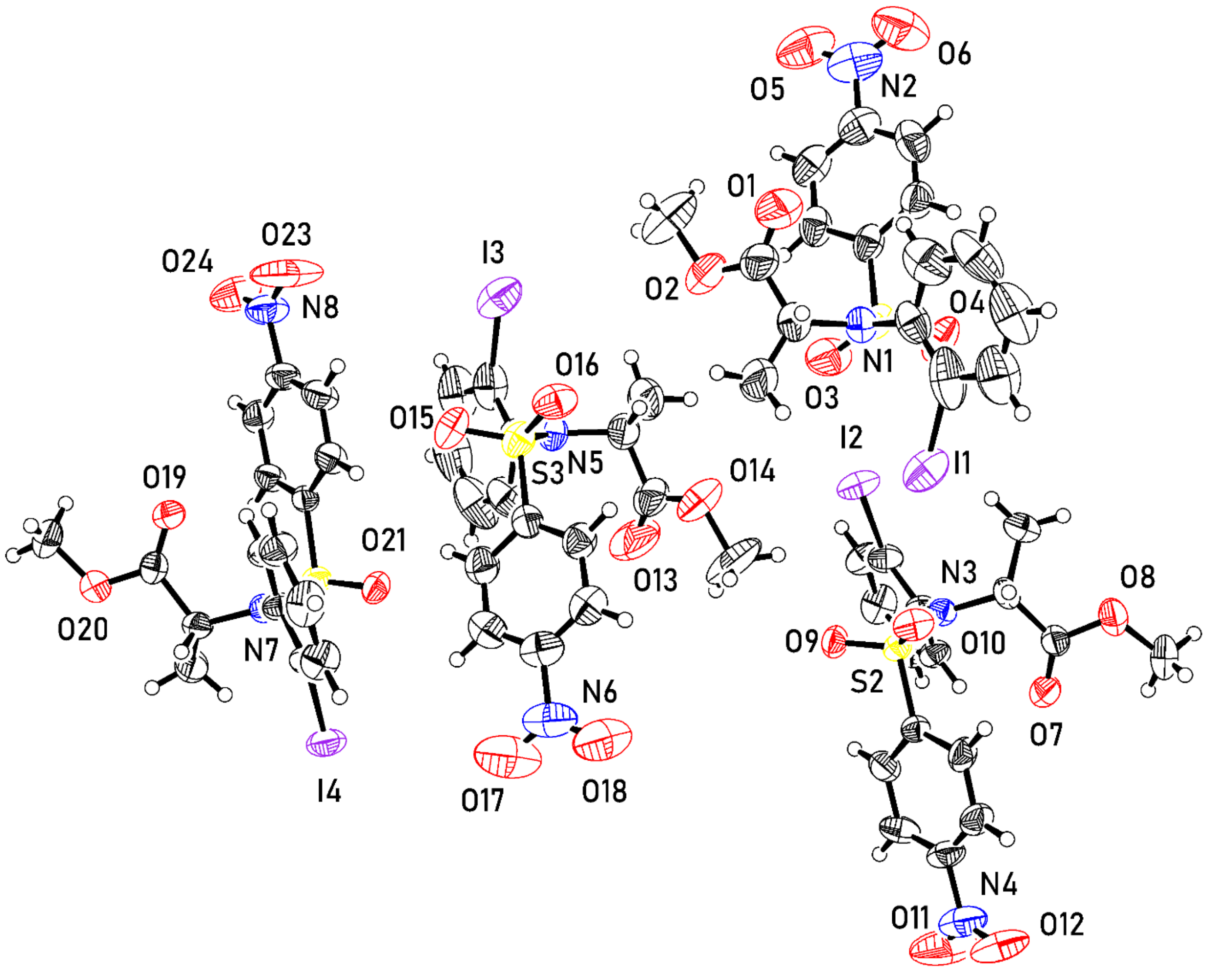

Figure S7: Solid state structure for **13f**. The ellipsoid probability level for the figure is 50%.

Table S5: Crystal data structure refinement for **13h** (CCDC 2247506).

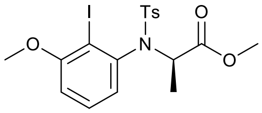

Empirical formula	$C_{18}H_{20}INO_5S$	
Formula weight	489.31	
Temperature	296(2) K	
Wavelength	1.54184 Å	
Crystal system	Monoclinic	
Space group	$P2_1$	
Unit cell dimensions	a = 7.5356(3) Å	a= 90°.
	b = 24.3613(5) Å	b= 108.534(3)°.
	c = 11.1773(3) Å	g = 90°.
Volume	1945.47(11) $Å^3$	
Z	4	
Density (calculated)	1.671 Mg/m^3	
Absorption coefficient	14.186 mm^{-1}	
F(000)	976	
Crystal size	0.330 x 0.070 x 0.030 mm^3	
Theta range for data collection	3.629 to 72.983°.	
Index ranges	-9<=h<=9, -30<=k<=29, -13<=l<=7	
Reflections collected	13939	
Independent reflections	7509 [R(int) = 0.0568]	
Completeness to theta = 67.684°	100.0 %	
Absorption correction	Gaussian	
Max. and min. transmission	1.000 and 0.680	
Refinement method	Full-matrix least-squares on F^2	
Data / restraints / parameters	7509 / 1 / 477	
Goodness-of-fit on F^2	1.100	
Final R indices [I>2sigma(I)]	R1 = 0.0491, wR2 = 0.1224	
R indices (all data)	R1 = 0.0543, wR2 = 0.1281	
Absolute structure parameter	-0.022(6)	
Largest diff. peak and hole	1.079 and -0.913 e.$Å^{-3}$	

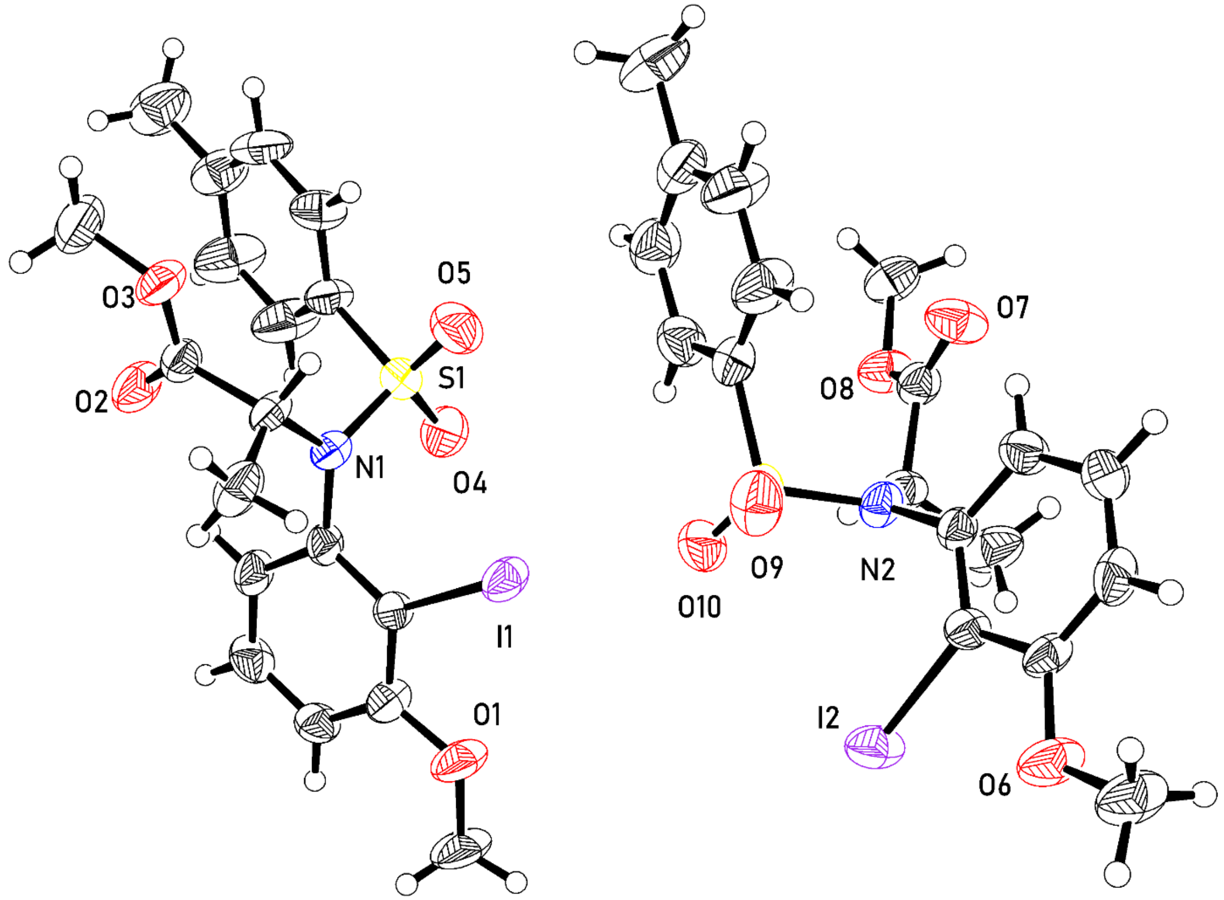

Figure S8: solid state structure for **13h**. The ellipsoid probability level for the figure is 50%.

Table S6. Crystal data and structure refinement for **13j** (CCDC 2247508).

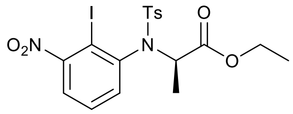

Empirical formula	$C_{18}H_{19}IN_2O_6S$	
Formula weight	518.31	
Temperature	293(2) K	
Wavelength	0.71073 Å	
Crystal system	Orthorhombic	
Space group	$P2_12_12_1$	
Unit cell dimensions	a = 7.7431(7) Å	a= 90°.
	b = 11.8770(10) Å	b= 90°.
	c = 22.517(2) Å	g = 90°.
Volume	2070.8(3) $Å^3$	
Z	4	
Density (calculated)	1.662 Mg/m^3	
Absorption coefficient	1.683 mm^{-1}	
F(000)	1032	
Crystal size	0.440 x 0.100 x 0.040 mm^3	
Theta range for data collection	3.548 to 29.671°.	
Index ranges	-7<=h<=10, -14<=k<=16, -31<=l<=20	
Reflections collected	9208	
Independent reflections	4862 [R(int) = 0.0272]	
Completeness to theta = 25.242°	99.5 %	
Absorption correction	Gaussian	
Max. and min. transmission	1.000 and 0.888	
Refinement method	Full-matrix least-squares on F^2	
Data / restraints / parameters	4862 / 0 / 256	
Goodness-of-fit on F^2	1.054	
Final R indices [I>2sigma(I)]	R1 = 0.0432, wR2 = 0.0884	
R indices (all data)	R1 = 0.0614, wR2 = 0.0981	
Absolute structure parameter	-0.016(14)	
Largest diff. peak and hole	0.693 and -0.760 e.$Å^{-3}$	

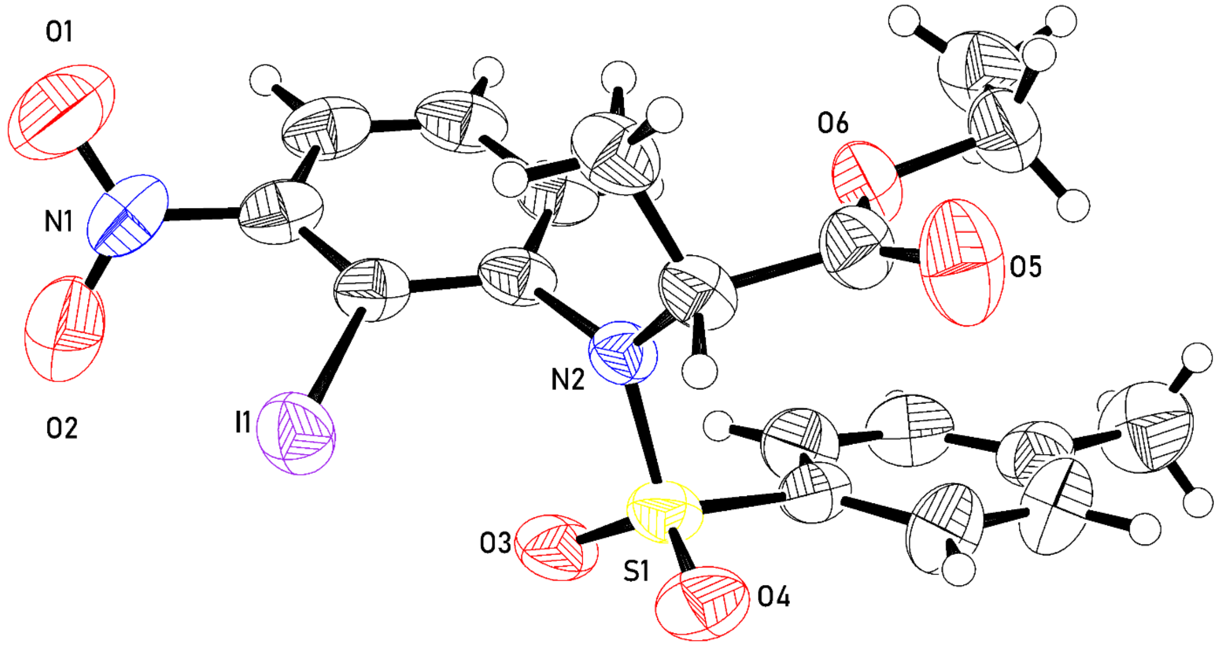

Figure S9: solid state structure for **13j**. The ellipsoid probability level for the figure is 50%.

Table S7. Crystal data structure refinement for **18c** (CCDC 2247509).

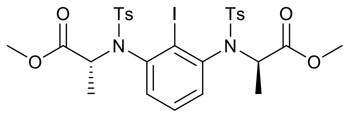

Empirical formula	$C_{28}H_{31}IN_2O\ S_2$	
Formula weight	714.57	
Temperature	293(2) K	
Wavelength	0.71073 Å	
Crystal system	Monoclinic	
Space group	P 21	
Unit cell dimensions	a = 9.9566(9) Å	a= 90°.
	b = 10.7075(7) Å	b= 93.818(8)°.
	c = 14.5745(10) Å	g = 90°.
Volume	1550.3(2) Å^3	
Z	2	
Density (calculated)	1.531 Mg/m^3	
Absorption coefficient	1.217 mm^{-1}	
F(000)	724	
Crystal size	0.540 x 0.440 x 0.090 mm^3	
Theta range for data collection	3.387 to 29.777°.	
Index ranges	-13<=h<=13, -14<=k<=14, -15<=l<=19	
Reflections collected	14209	
Independent reflections	7035 [R(int) = 0.0664]	
Completeness to theta = 25.242°	97.4 %	
Absorption correction	Semi-empirical from equivalents	
Max. and min. transmission	1.00000 and 0.28784	
Refinement method	Full-matrix least-squares on F^2	
Data / restraints / parameters	7035 / 1 / 376	
Goodness-of-fit on F^2	0.937	
Final R indices [I>2sigma(I)]	R1 = 0.0431, wR2 = 0.0842	
R indices (all data)	R1 = 0.0680, wR2 = 0.0906	
Absolute structure parameter	-0.031(18)	
Largest diff. peak and hole	0.627 and -0.441 e.Å^{-3}	

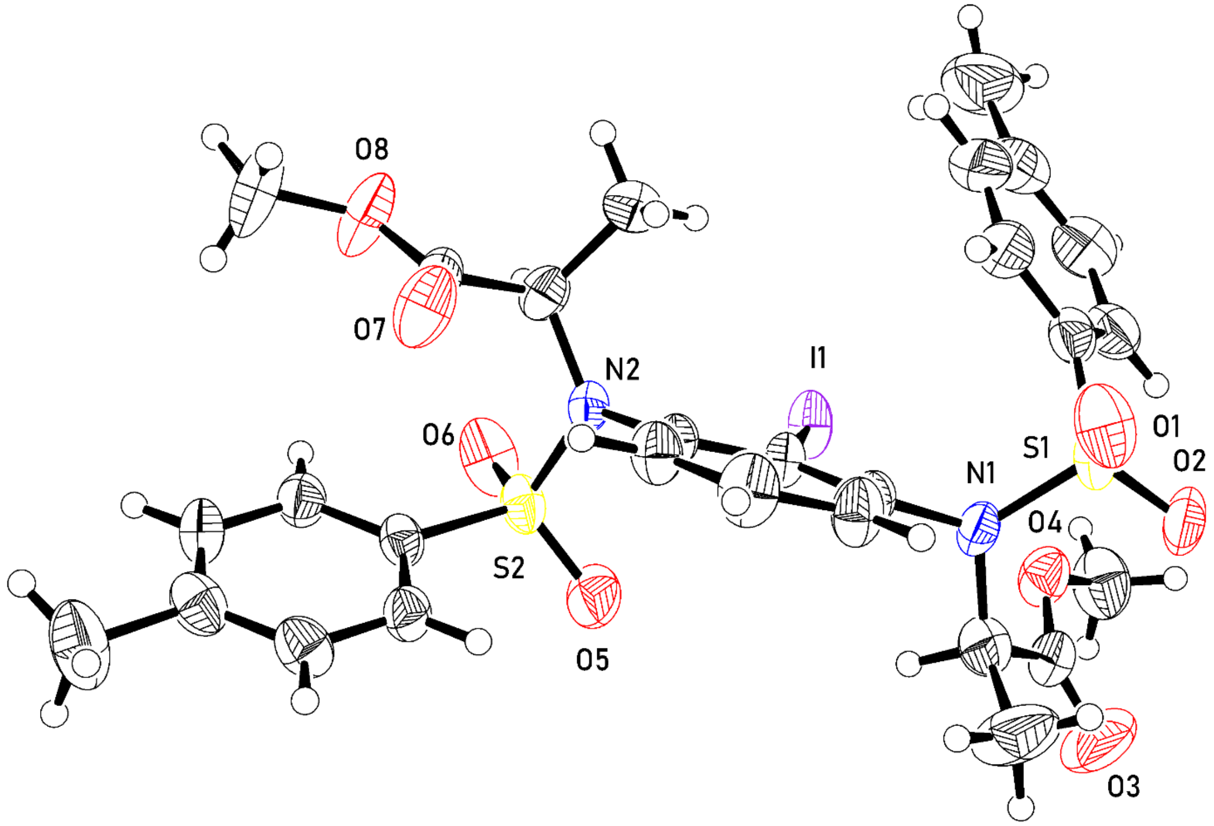

Figure S10: solid state structure for **18c**. The ellipsoid probability level for the figure is 50%.

References:

(1) Alharbi, H.; Elsherbini, M.; Qurban, J.; Wirth, T. C−N Axial Chiral Hypervalent Iodine Reagents: Catalytic Stereoselective α-Oxytosylation of Ketones. *Chem. Eur. J.* **2021**, *27*, 4317–4321. https://doi.org/10.1002/chem.202005253.

(2) Fujita, M.; Okuno, S.; Lee, H. J.; Sugimura, T.; Okuyama, T. Enantiodifferentiating Tetrahydrofuranylation of But-3-Enyl Carboxylates Using Optically Active Hypervalent Iodine(III) Reagents via a 1,3-Dioxan-2-Yl Cation Intermediate. *Tetrahedron Lett.* **2007**, *48*, 8691–8694. https://doi.org/10.1016/j.tetlet.2007.10.015.

(3) Haubenreisser, S.; Wöste, T. H.; Martínez, C.; Ishihara, K.; Muñiz, K. Structurally Defined Molecular Hypervalent Iodine Catalysts for Intermolecular Enantioselective Reactions. *Angew. Chem. Int. Ed.* **2016**, *55*, 413–417. https://doi.org/10.1002/anie.201507180.

(4) Kondo, Y.; Kojima, S.; Sakamoto, T. *General and Facile Synthesis of Indoles with Oxygen-Bearing Substituents at the Benzene Moiety*; 1997. https://pubs.acs.org/sharingguidelines.

(5) Wu, W.; Li, Z.; Zhou, G.; Jiang, S. Total Synthesis of Argyrins A and E. *Tetrahedron Lett.* **2011**, *52*, 2488–2491. https://doi.org/10.1016/J.TETLET.2011.03.021.

(6) Damrath, M.; Caspers, L. D.; Duvinage, D.; Nachtsheim, B. J. One-Pot Synthesis of Heteroatom-Bridged Cyclic Diaryliodonium Salts. *Org. Lett.* **2022**, *24*, 2562–2566. https://doi.org/10.1021/acs.orglett.2c00691.

(7) Flynn, A. R.; McDaniel, K. A.; Hughes, M. E.; Vogt, D. B.; Jui, N. T. Hydroarylation of Arenes via Reductive Radical-Polar Crossover. *J. Am. Chem. Soc.* **2020**, *142*, 9163–9168. https://doi.org/10.1021/jacs.0c03926.

(8) Xiong, X.; Yeung, Y.-Y. Highly *Ortho* -Selective Chlorination of Anilines Using a Secondary Ammonium Salt Organocatalyst. *Angew. Chem. Int. Ed.* **2016**, *55*, 16101–16105. https://doi.org/10.1002/anie.201607388.

(9) Kofink, C. C.; Blank, B.; Pagano, S.; Götz, N.; Knochel, P. Iron-Catalyzed Aryl–Aryl Cross-Coupling Reaction Tolerating Amides and Unprotected Quinolinones. *Chem. Commun.* **2007**, 1954–1956. https://doi.org/10.1039/B618617C.

(10) Knipe, P. C.; Lingard, H.; Jones, I. M.; Thompson, S.; Hamilton, A. D. A Lewis Acid-Mediated Conformational Switch. *Org. Biomol. Chem.* **2014**, *12*, 7937–7941. https://doi.org/10.1039/C4OB01556H.

(11) Sun, X.; Li, W.; Hou, G.; Zhou, L.; Zhang, X. Axial Chirality Control by 2,4-Pentanediol for the Alternative Synthesis of C_3 *-TunePhos Chiral Diphosphine Ligands and Their Applications in Highly

Enantioselective Ruthenium-Catalyzed Hydrogenation of β-Keto Esters. *Adv. Synth. Catal.* **2009**, *351*, 2553–2557. https://doi.org/10.1002/adsc.200900589.

(12) Xu, Z.; Hu, W.; Liu, Q.; Zhang, L.; Jia, Y. Total Synthesis of Clavicipitic Acid and Aurantioclavine: Stereochemistry of Clavicipitic Acid Revisited. *J. Org. Chem.* **2010**, *75*, 7626–7635. https://doi.org/10.1021/jo101506c.

(13) Mani, B.; Kathavarayan, S. Studies on photocrosslinking and flame-retardant properties of chalcone-based polyacrylamides. *Polym. Adv. Technol.* **2016**, *27*, 466–476. DOI:10.1002/pat.3692.

(14) Jones, I. M.; Hamilton, A. D. Anion-Dependent Switching: Dynamically Controlling the Conformation of Hydrogen-Bonded Diphenylacetylenes. *Angew. Chem. Int. Ed.* **2011**, *50*, 4597–4600. https://doi.org/10.1002/anie.201100144.

(15) Paduraru, P. M.; Popoff, R. T. W.; Nair, R.; Gries, R.; Gries, G.; Plettner, E. Synthesis of Substituted Alkoxy Benzene Minilibraries, for the Discovery of New Insect Olfaction or Gustation Inhibitors. *J. Comb. Chem.* **2008**, *10*, 123–134. https://doi.org/10.1021/cc700139y.

(16) García-López, J.-A.; Çetin, M.; Greaney, M. F. Double Heteroatom Functionalization of Arenes Using Benzyne Three-Component Coupling. *Angew. Chem. Int. Ed.* **2015**, *54*, 2156–2159. https://doi.org/10.1002/anie.201410751.

(17) Shimogaki, M.; Fujita, M.; Sugimura, T. Enantioselective Oxidation of Alkenylbenzoates Catalyzed by Chiral Hypervalent Iodine(III) To Yield 4-Hydroxyisochroman-1-Ones. *Eur. J. Org. Chem.* **2013**, 7128–7138. https://doi.org/10.1002/ejoc.201300959.

(18) Levitre, G.; Dumoulin, A.; Retailleau, P.; Panossian, A.; Leroux, F. R.; Masson, G. Asymmetric α-Sulfonyl-and α-Phosphoryl-Oxylation of Ketones by a Chiral Hypervalent Iodine(III). *J. Org. Chem.* **2017**, *82*, 11877–11883. https://doi.org/10.1021/acs.joc.7b01597.

(19) Guilbault, A. A.; Basdevant, B.; Wanie, V.; Legault, C. Y. Catalytic Enantioselective α-Tosyloxylation of Ketones Using Iodoaryloxazoline Catalysts: Insights on the Stereoinduction Process. *J. Org. Chem.* **2012**, *77*, 11283–11295. https://doi.org/10.1021/jo302393u.

(20) Lex, T. R.; Swasy, M. I.; Whitehead, D. C. Relative Rate Profiles of Functionalized Iodoarene Catalysts for Iodine(III) Oxidations. *J. Org. Chem.* **2015**, *80*, 12234–12243. https://doi.org/10.1021/acs.joc.5b02129.

(21) Brenet, S.; Berthiol, F.; Einhorn, J. 3,3′-Diiodo-BINOL-Fused Maleimides as Chiral Hypervalent Iodine(III) Organocatalysts. *Eur. J. Org. Chem.* **2013**, 8094–8096. https://doi.org/10.1002/ejoc.201301329.

(22) Basdevant, B.; Legault, C. Y. Enantioselective Iodine(III)-Mediated Synthesis of α-Tosyloxy Ketones: Breaking the Selectivity Barrier. *Org. Lett.* **2015**, *17*, 4918–4921, DOI: 10.1021/acs.orglett.5b02501.

(23) Altermann, S. M.; Richardson, R. D.; Page, T. K.; Schmidt, R. K.; Holland, E.; Mohammed, U.; Paradine, S. M.; French, A. N.; Richter, C.; Bahar, A. M.; Witulski, B.; Wirth, T. Catalytic Enantioselective α-Oxysulfonylation of Ketones Mediated by Iodoarenes. *Eur. J. Org. Chem.* **2008**, 5315–5328. https://doi.org/10.1002/ejoc.200800741.

NMR spectra:

***tert*-Butyl 3-methoxyphenylcarbamate (S1)**
^{1}H NMR (500 MHz, $CDCl_3$)
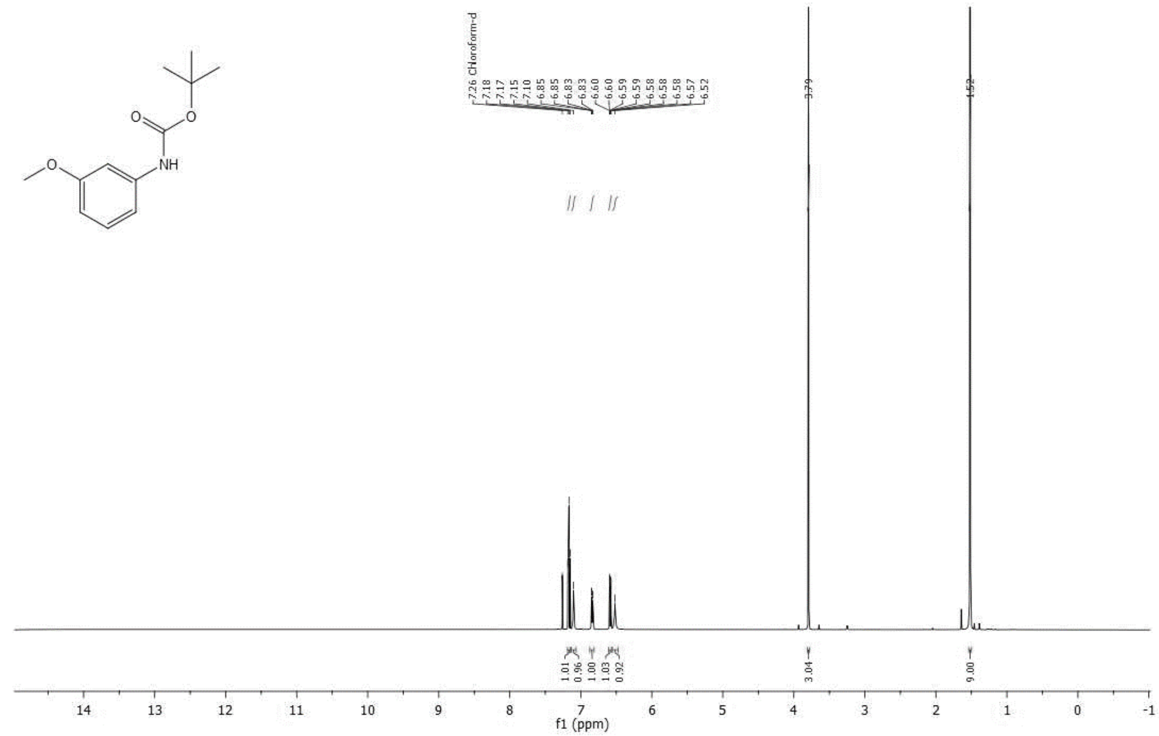
$^{13}C\{^{1}H\}$ NMR (126 MHz, $CDCl_3$)
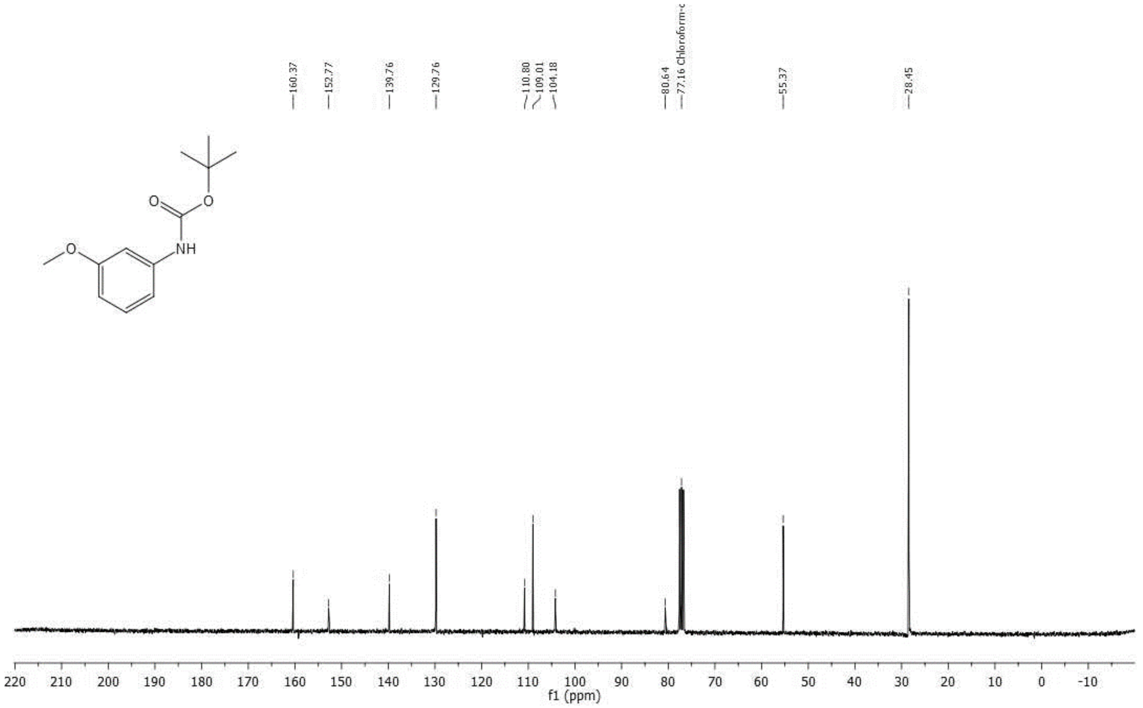

***tert*-Butyl 2-iodo-3-methoxyphenylcarbamate (S2)**

^{1}H NMR (400 MHz, $CDCl_3$)

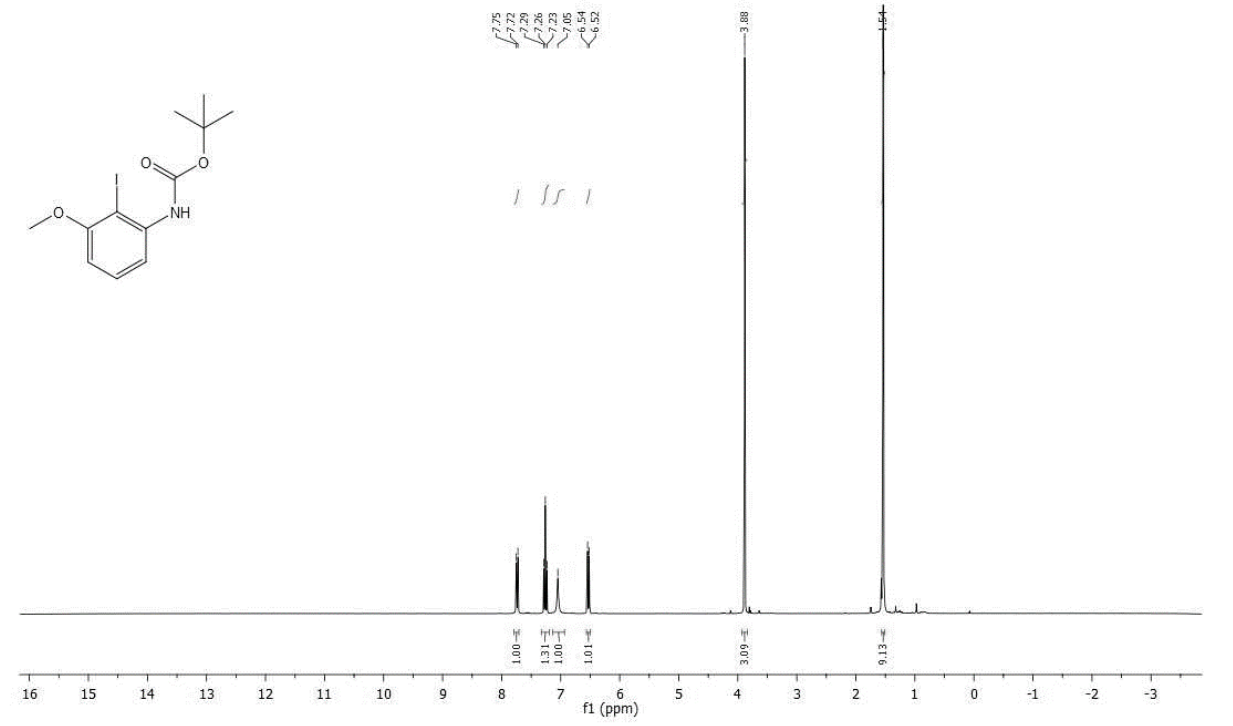

$^{13}C\{^{1}H\}$ NMR (100 MHz, $CDCl_3$)

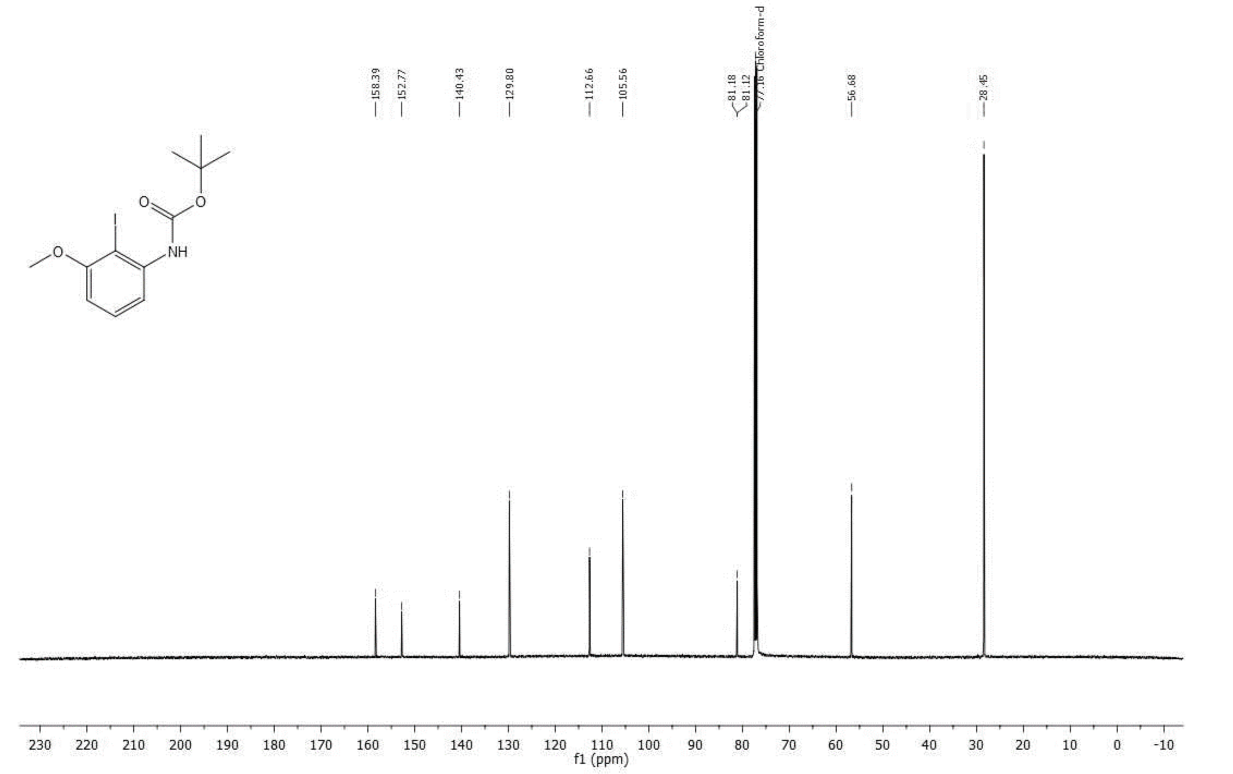

2-Iodo-1,3-dinitrobenzene (S3)

^{1}H NMR (500 MHz, $CDCl_3$)

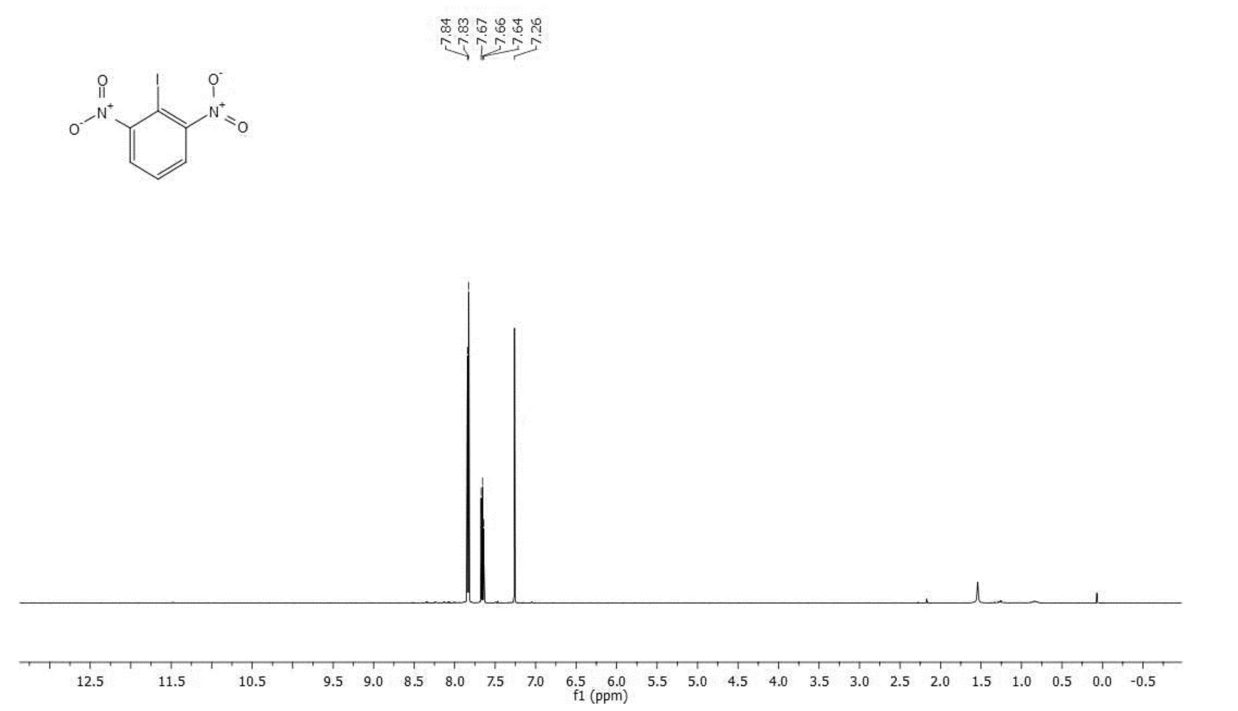

$^{13}C\{^{1}H\}$ NMR (126 MHz, $CDCl_3$)

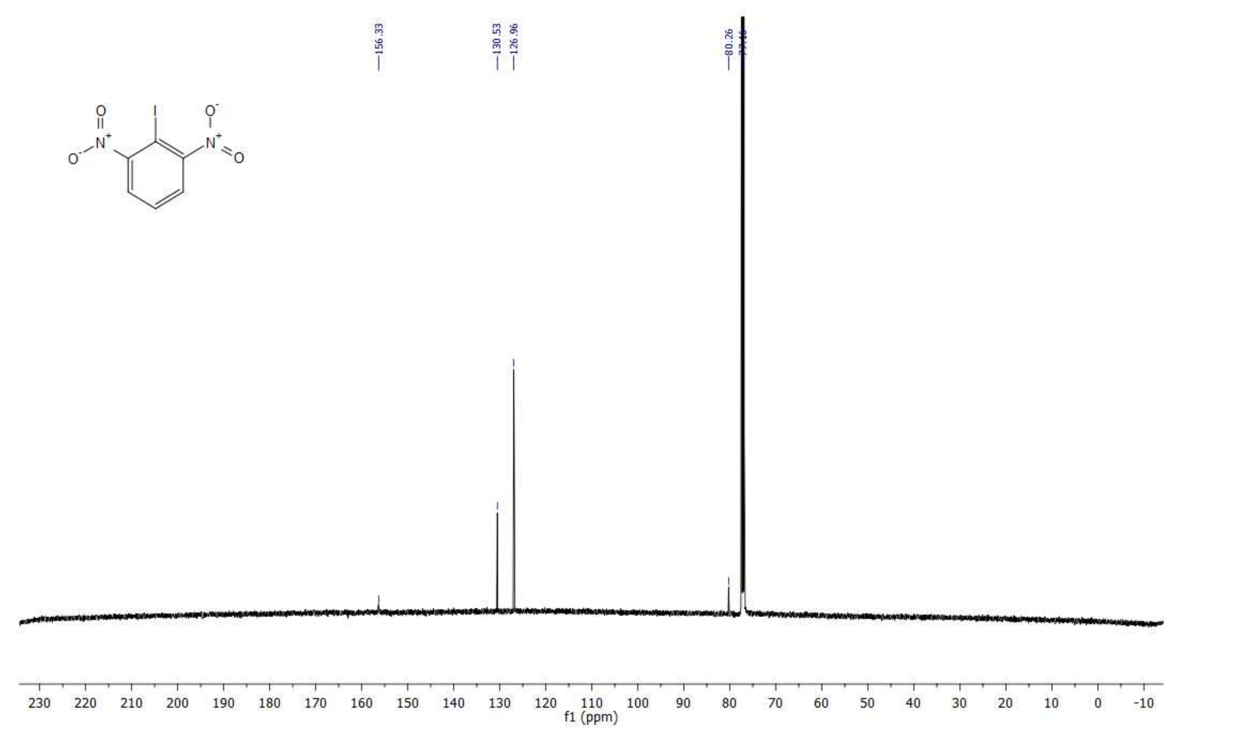

2-Iodo-3-nitrophenol (S4)

^{1}H NMR (500 MHz, $CDCl_3$)

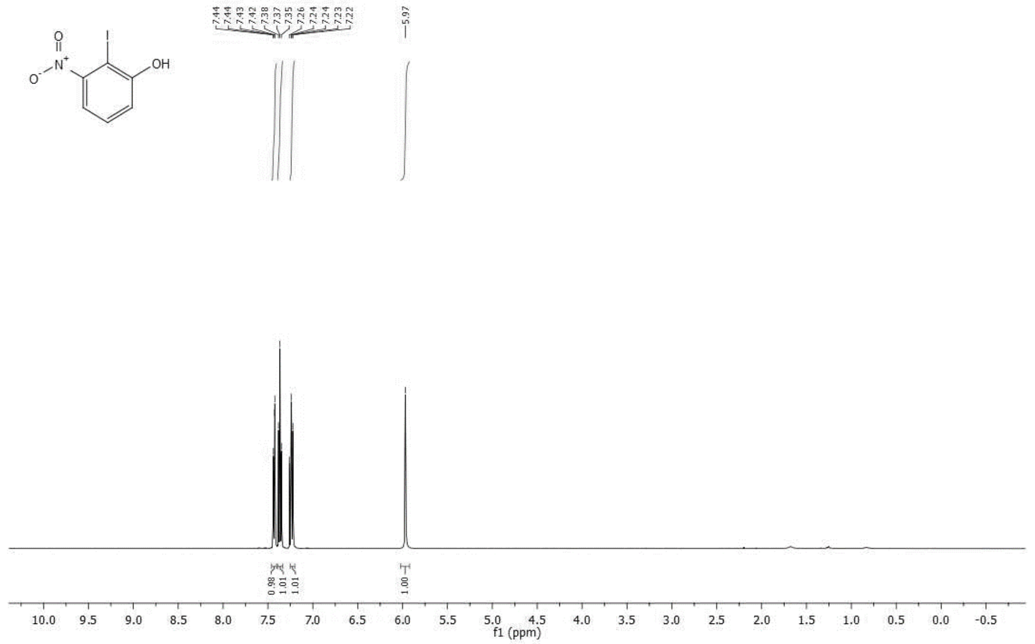

^{13}C{^{1}H} NMR (126 MHz, $CDCl_3$)

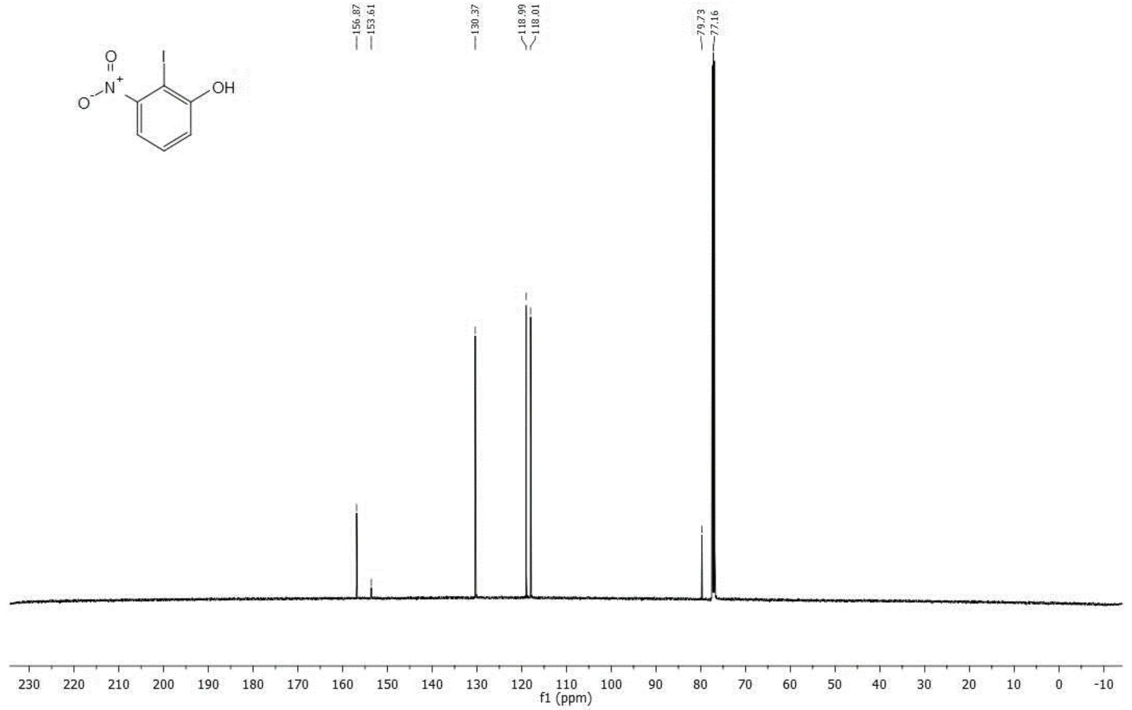

Dimethyl 2,2'-((2-iodo-1,3-phenylene)bis(oxy))(2*R*,2'*R*)-dipropionate (7)

^{1}H NMR (500 MHz, $CDCl_3$)

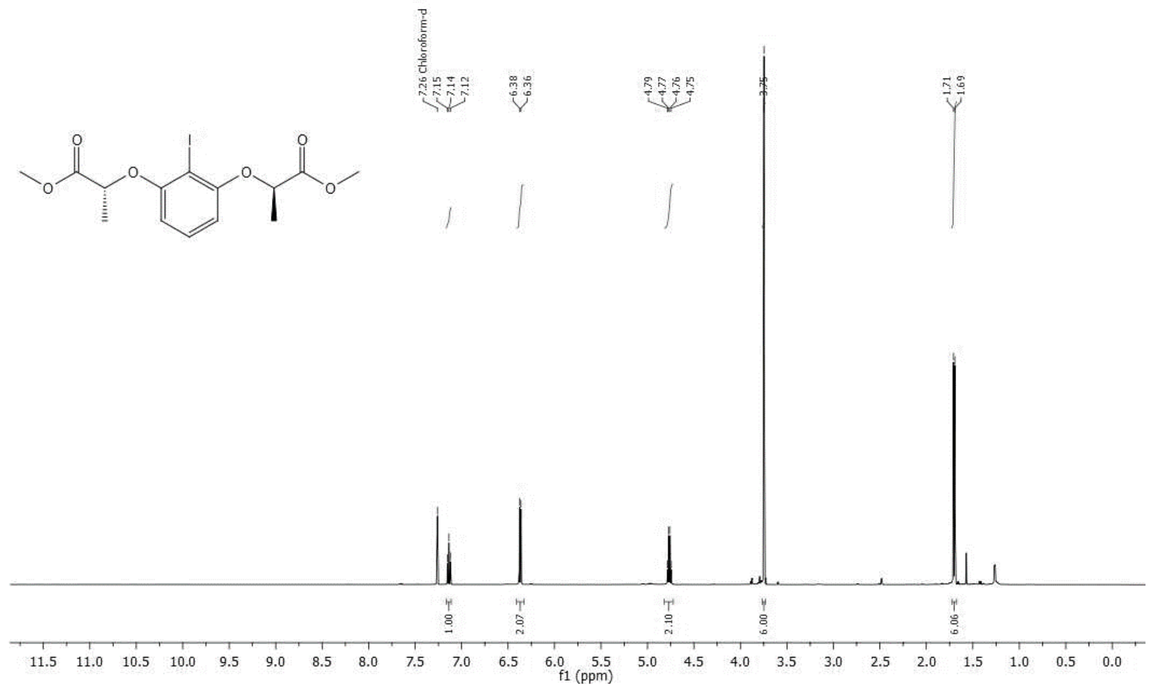

^{13}C{^{1}H} NMR (126 MHz, $CDCl_3$)

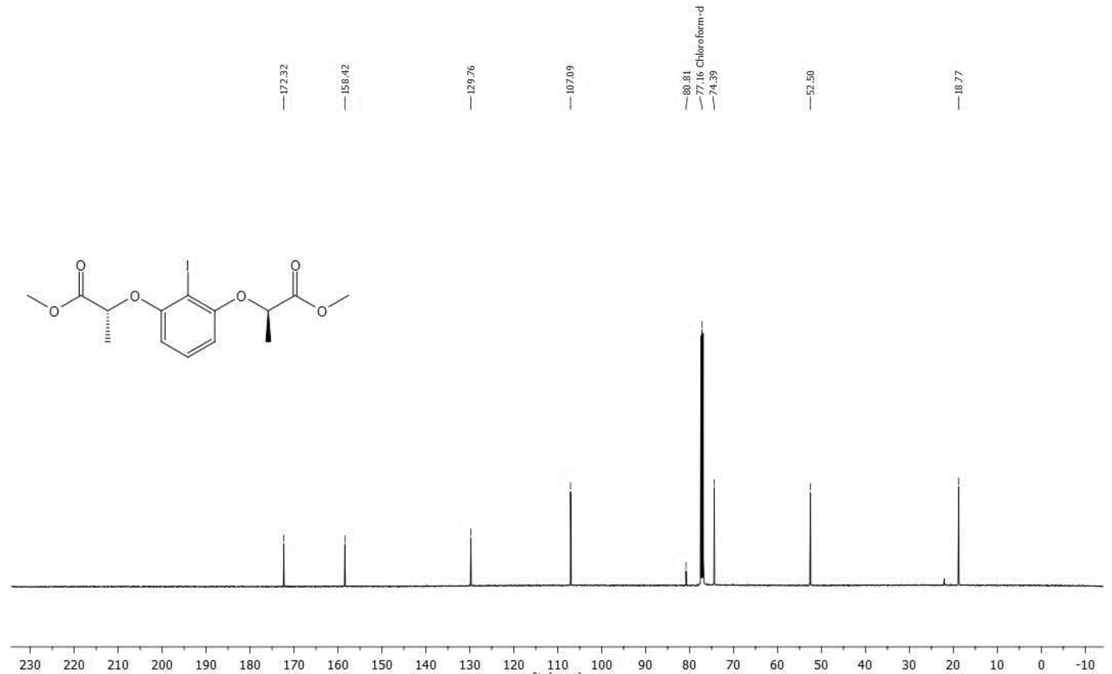

2-Iodo 3-methoxybenzenamine (10)

^{1}H NMR (500 MHz, $CDCl_3$)

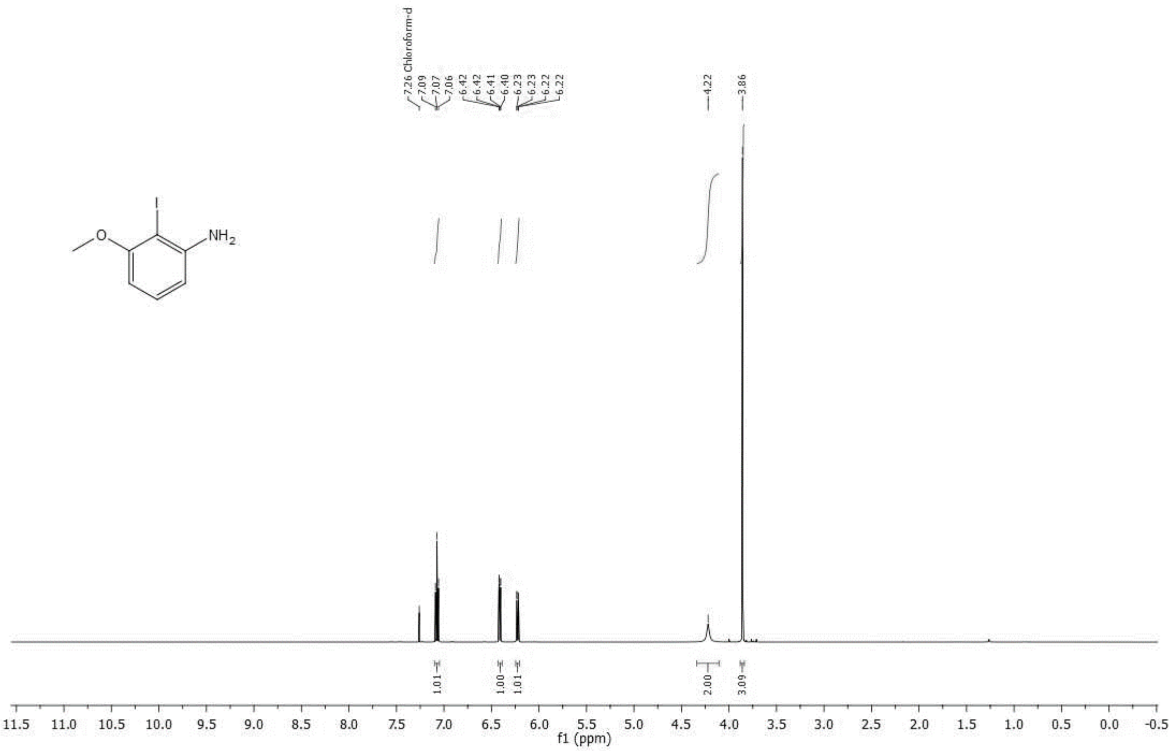

$^{13}C\{^{1}H\}$ NMR (126 MHz, $CDCl_3$)

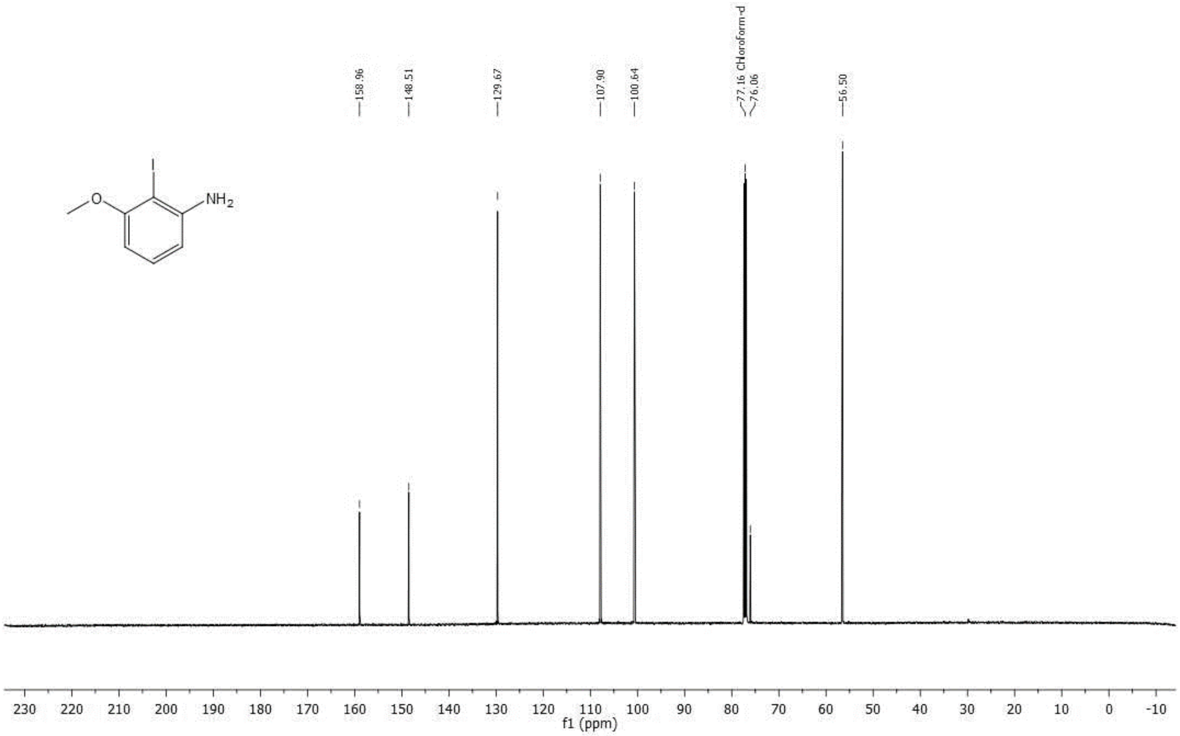

2-Iodo-3-nitroaniline (11)

^{1}H NMR (300 MHz, $CDCl_3$)

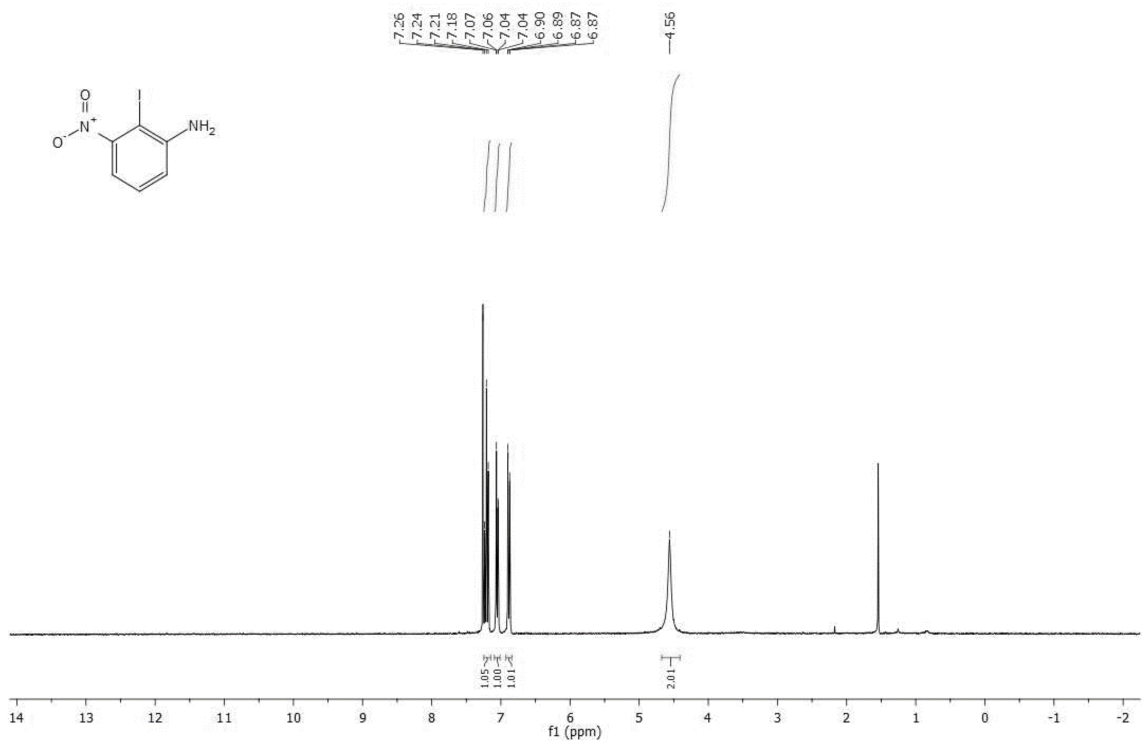

$^{13}C\{^{1}H\}$ NMR (126 MHz, $CDCl_3$)

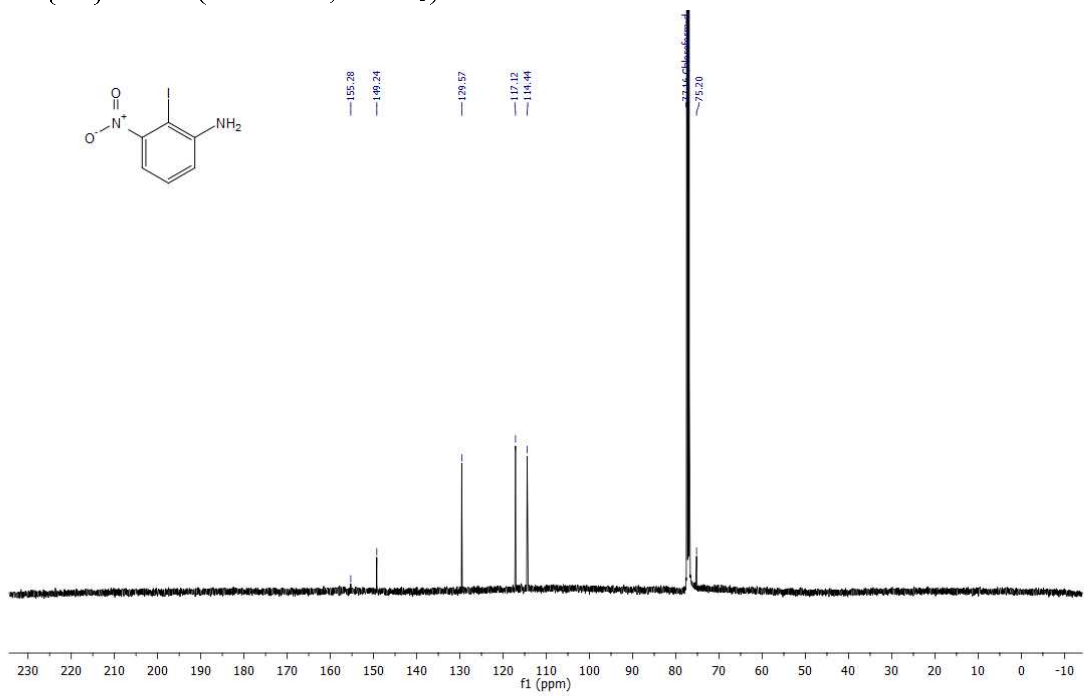

N-(2-Iodophenyl)-4-methylbenzenesulfonamide (12a)

^{1}H NMR (500 MHz, $CDCl_3$)

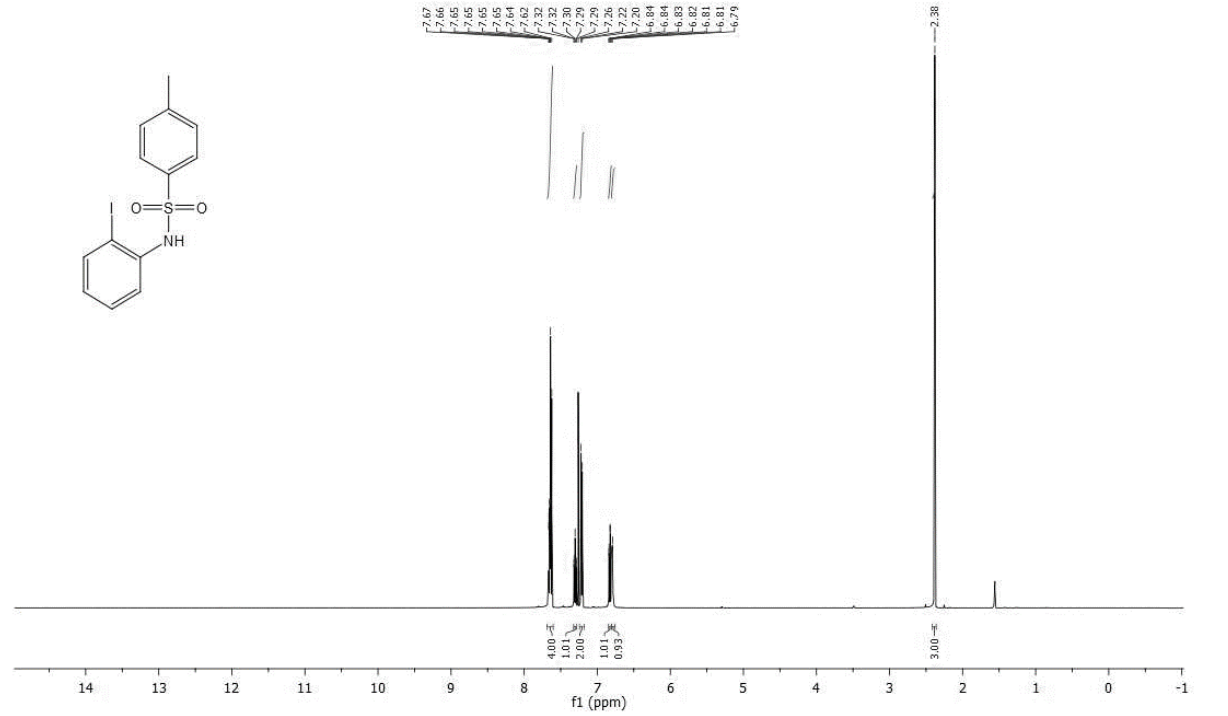

$^{13}C\{^{1}H\}$ NMR (126 MHz, $CDCl_3$)

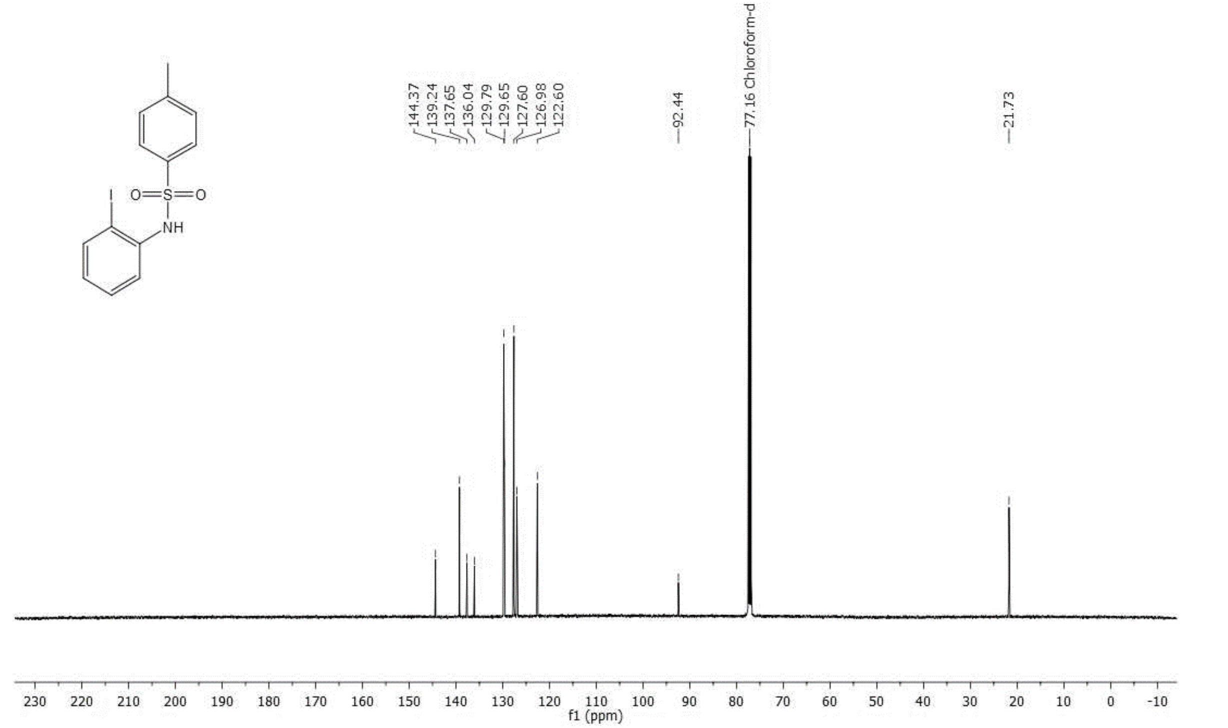

N-(2-Iodophenyl)-2,4,6-trimethylbenzenesulfonamide (12b)

^{1}H NMR (500 MHz, $CDCl_3$)

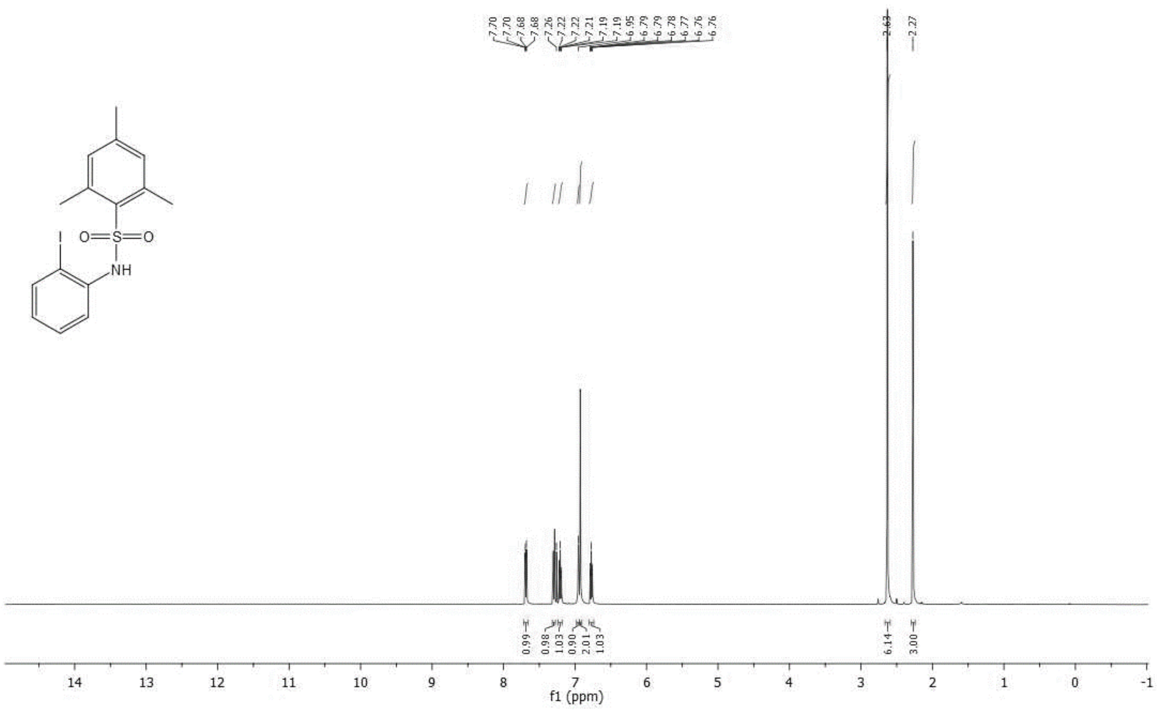

$^{13}C\{^{1}H\}$ NMR (126 MHz, $CDCl_3$)

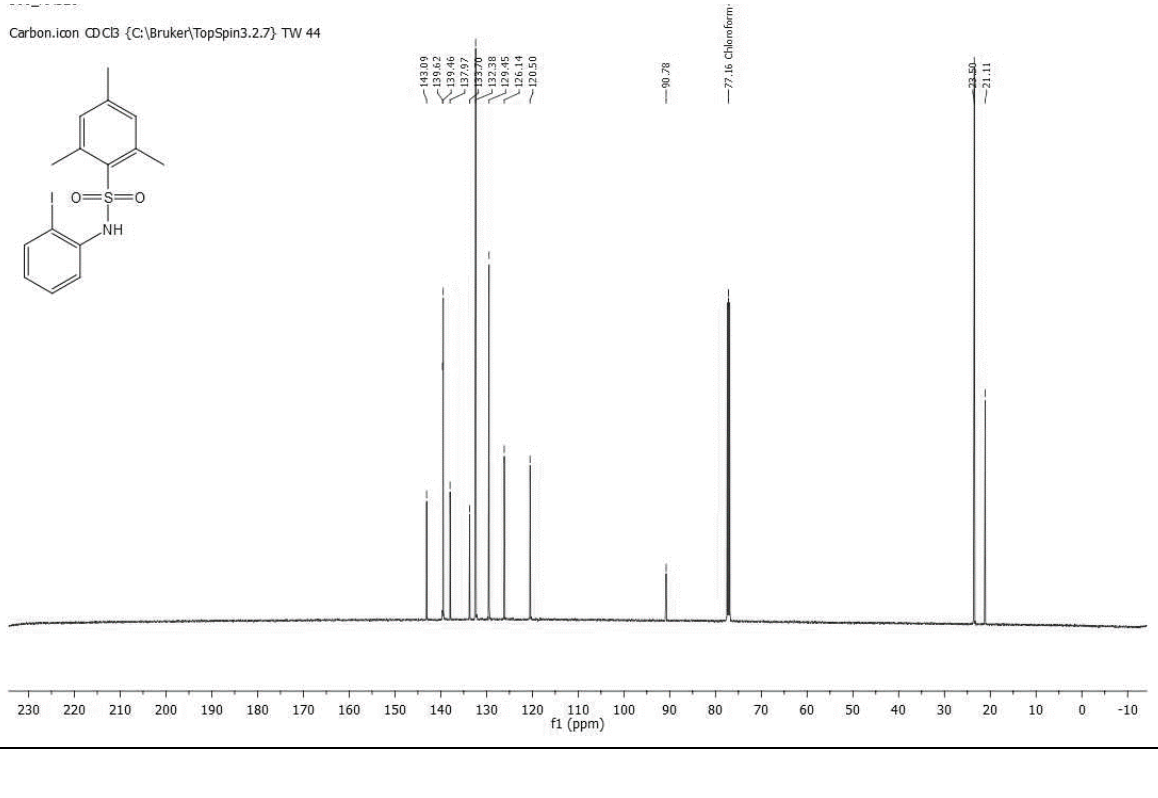

***N*-(2-Iodophenyl)methanesulfonamide (12c)**

^{1}H NMR (300 MHz, $CDCl_3$)

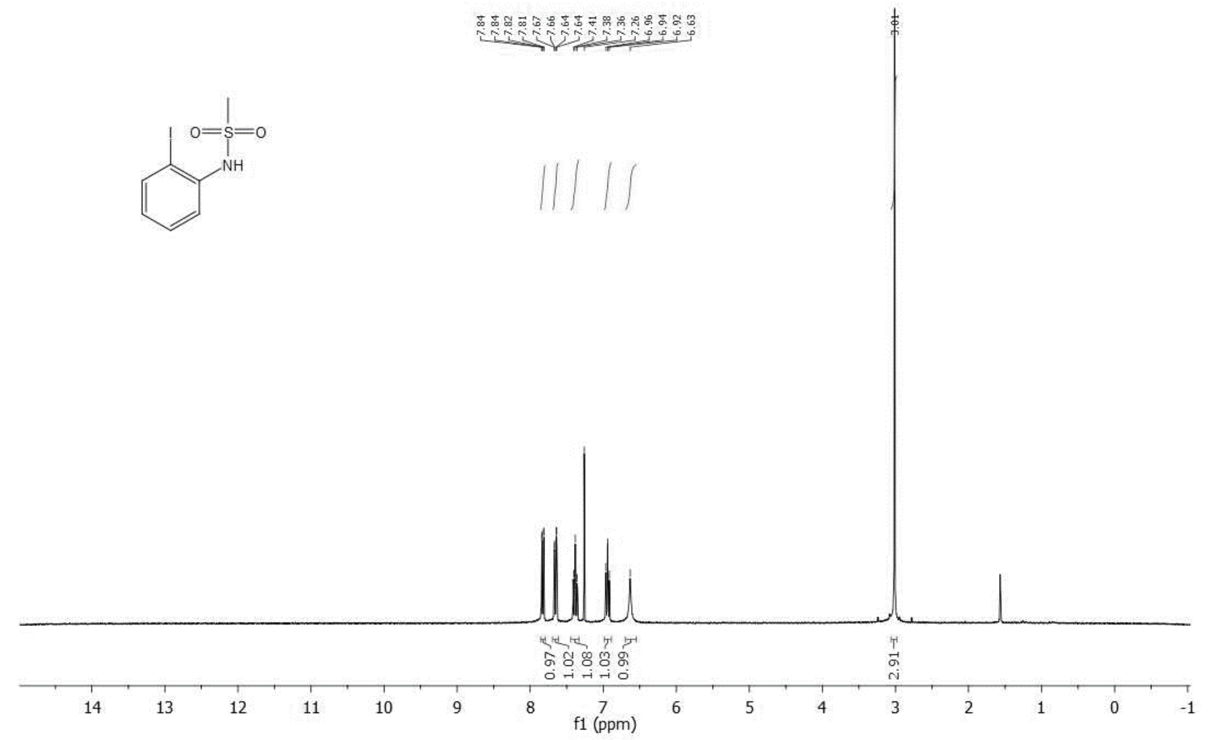

$^{13}C\{^{1}H\}$ NMR (126 MHz, $CDCl_3$)

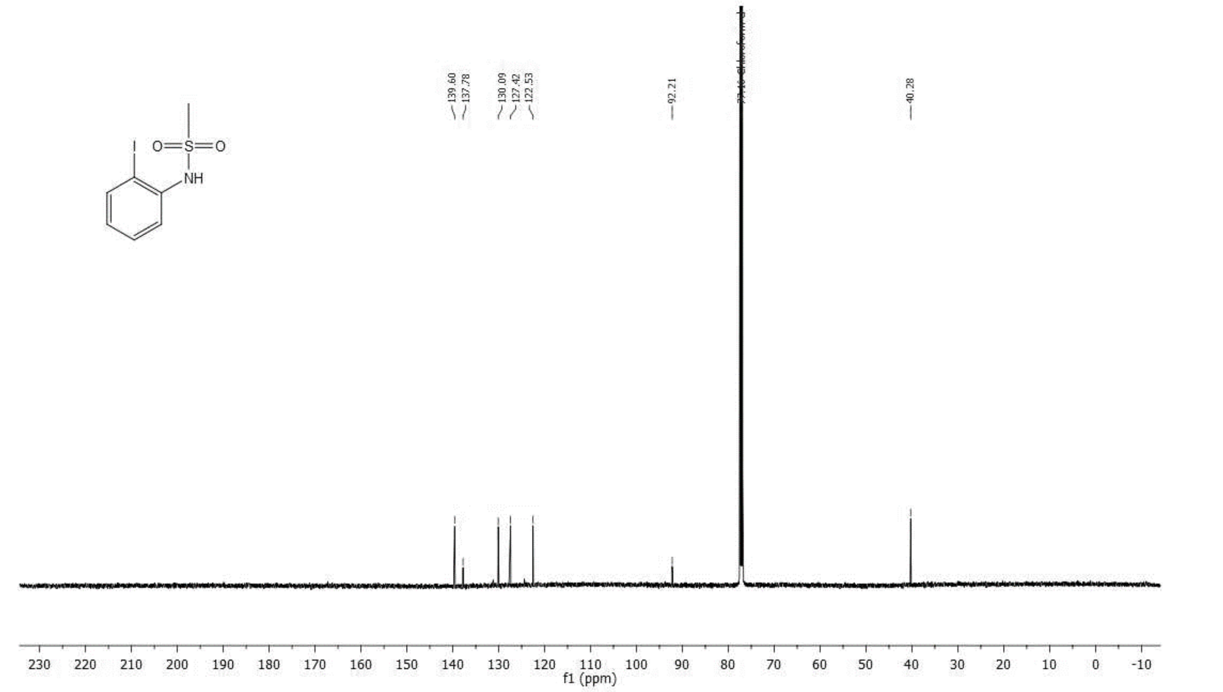

***N*-(2-Iodophenyl)-4-(trifluoromethyl)benzenesulfonamide (12d)**

^{1}H NMR (400 MHz, $CDCl_3$)

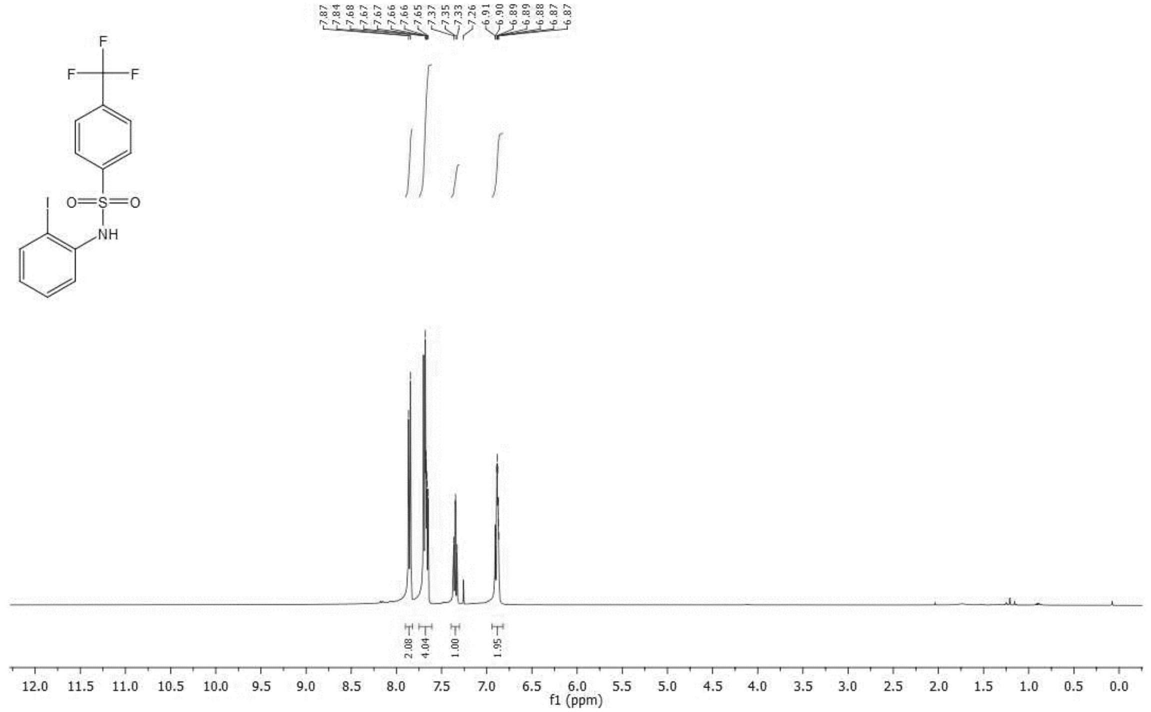

^{13}C{^{1}H} NMR (126 MHz, $CDCl_3$)

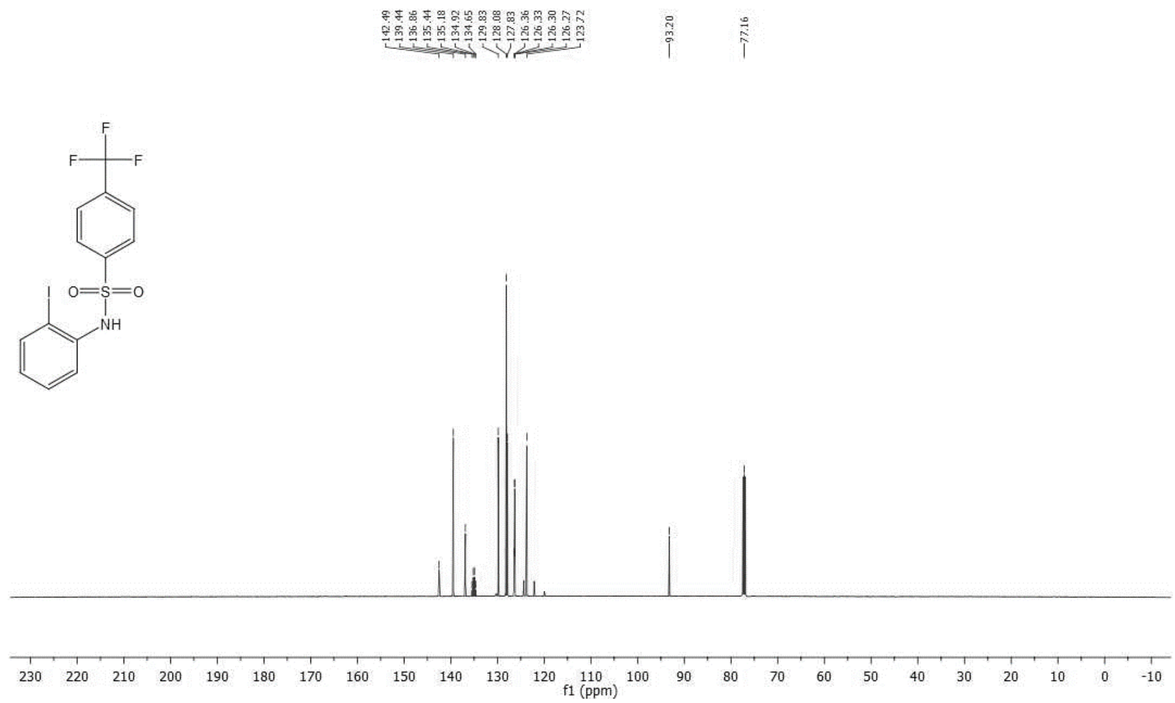

***N*-(2-Iodophenyl)-4-nitrobenzenesulfonamide (12e)**

^{1}H NMR (300 MHz, $CDCl_3$)

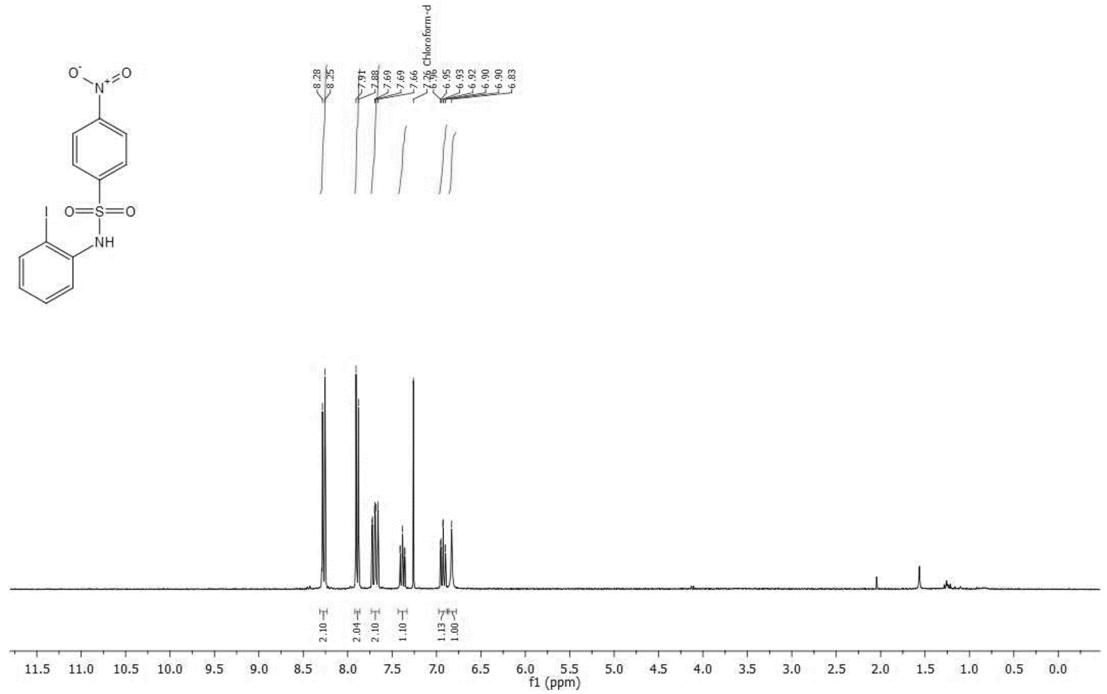

$^{13}C\{^{1}H\}$ NMR (126 MHz, $CDCl_3$)

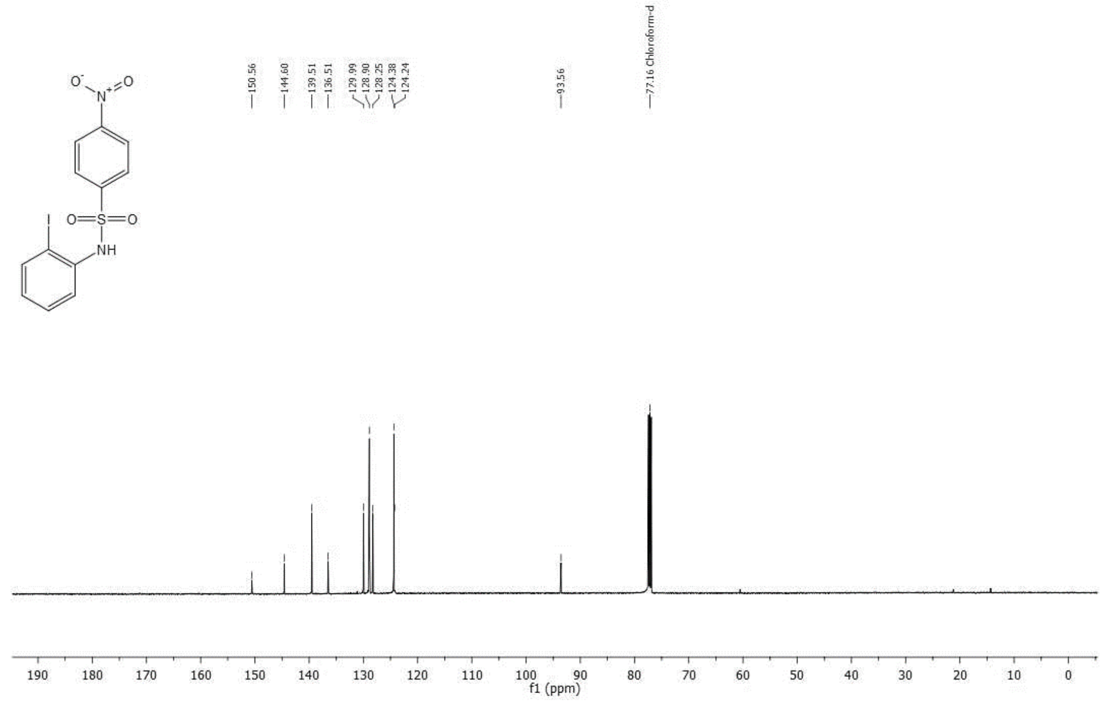

1,1,1-Trifluoro-*N*-(2-iodophenyl)methanesulfonamide (12f)

^{1}H NMR (400 MHz, $CDCl_3$)

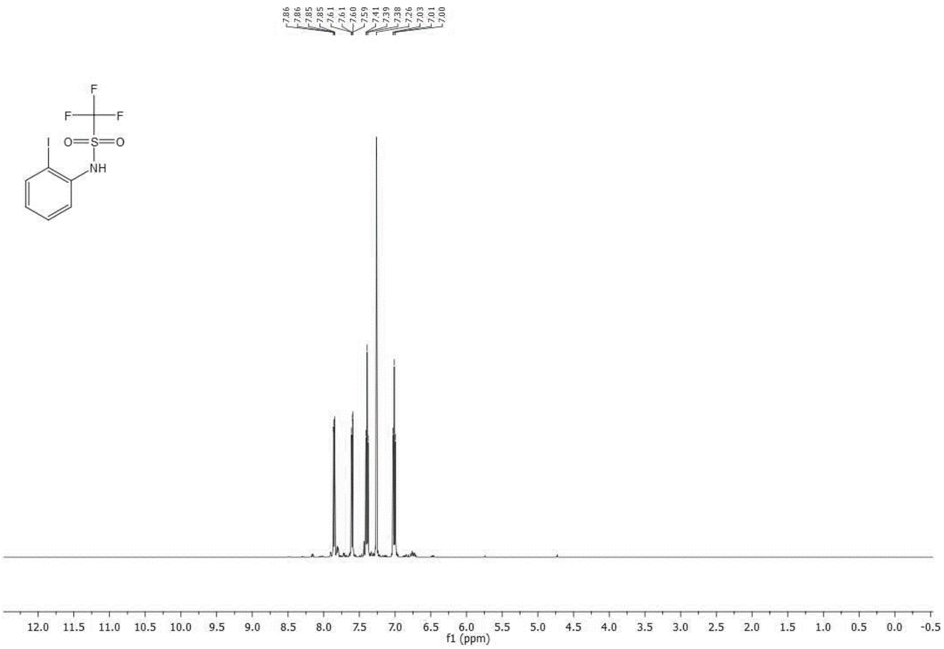

^{13}C{^{1}H} NMR (100 MHz, $CDCl_3$)

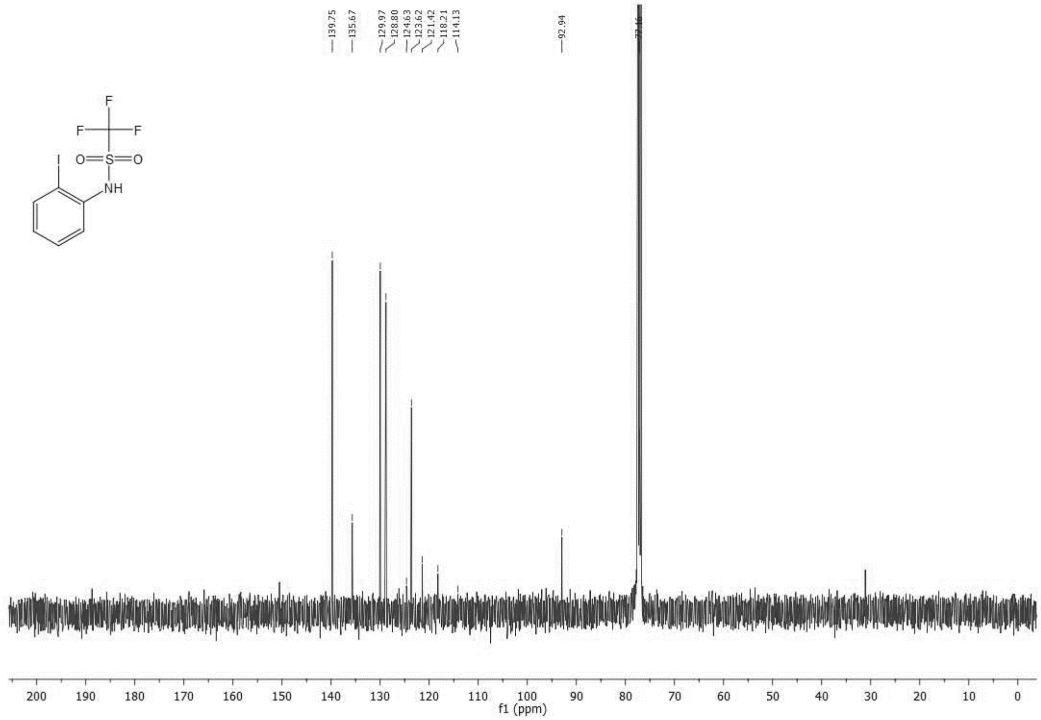

N-(2-Iodo-3-methoxyphenyl)-4-methylbenzenesulfonamide (12g)

^{1}H NMR (500 MHz, $CDCl_3$)

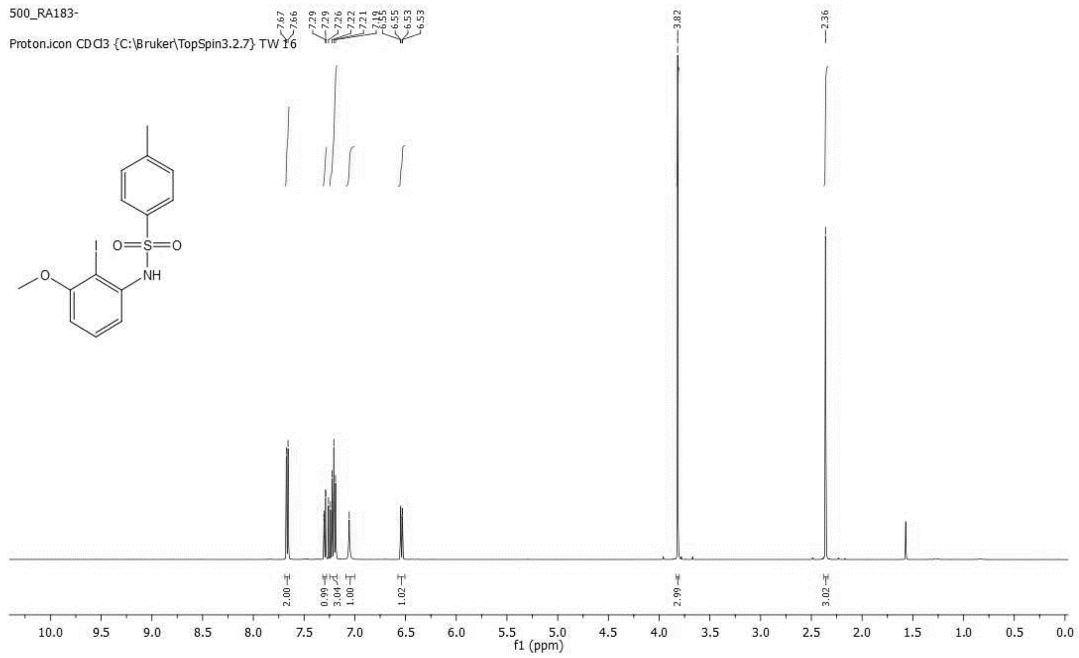

$^{13}C\{^{1}H\}$ NMR (126 MHz, $CDCl_3$)

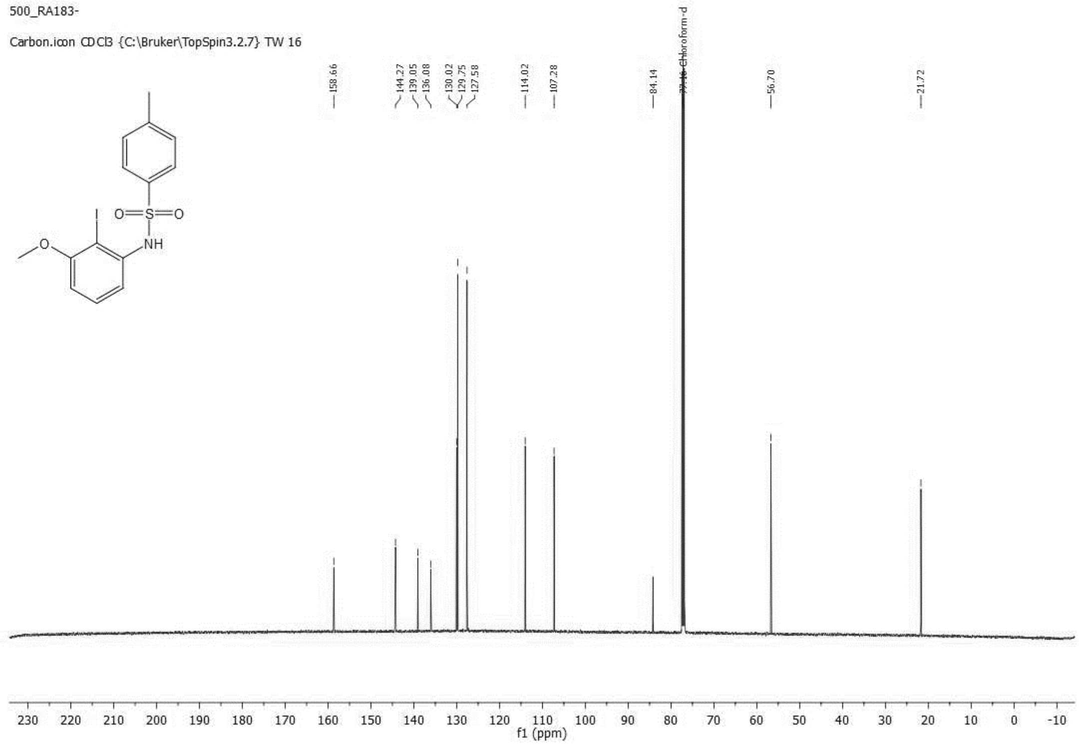

N-(2-Iodo-3-nitrophenyl)-4-methylbenzenesulfonamide (12h)

^{1}H NMR (500 MHz, $CDCl_3$)

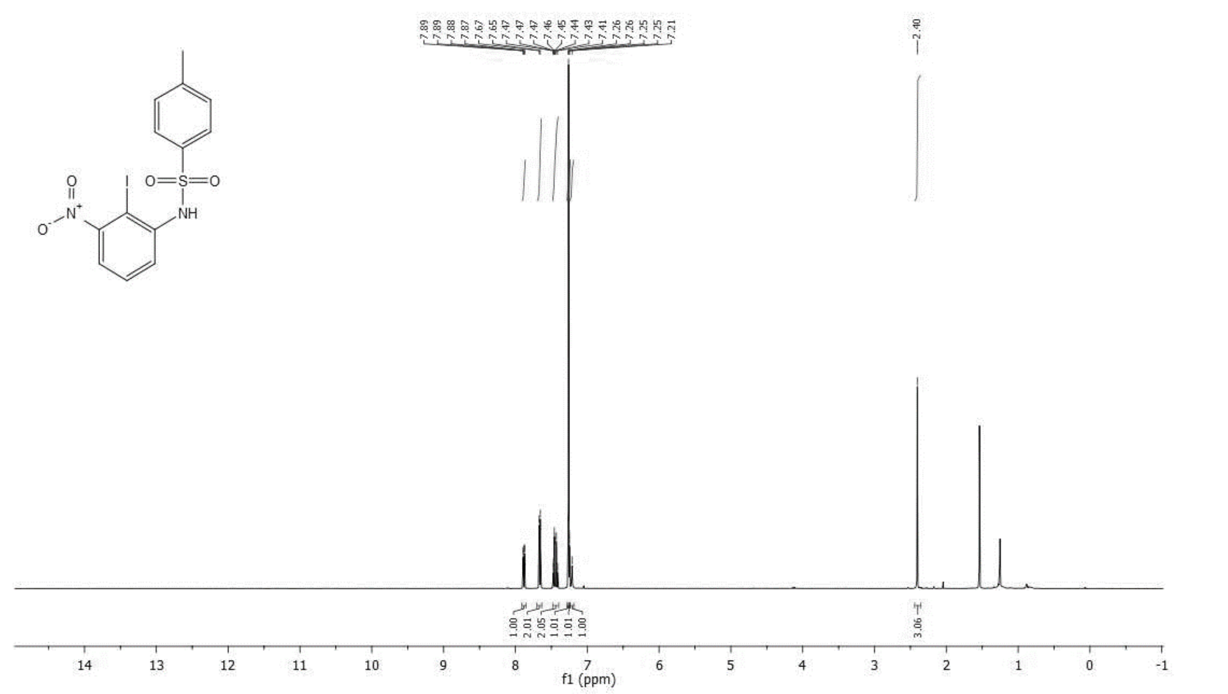

$^{13}C\{^{1}H\}$ NMR (126 MHz, $CDCl_3$)

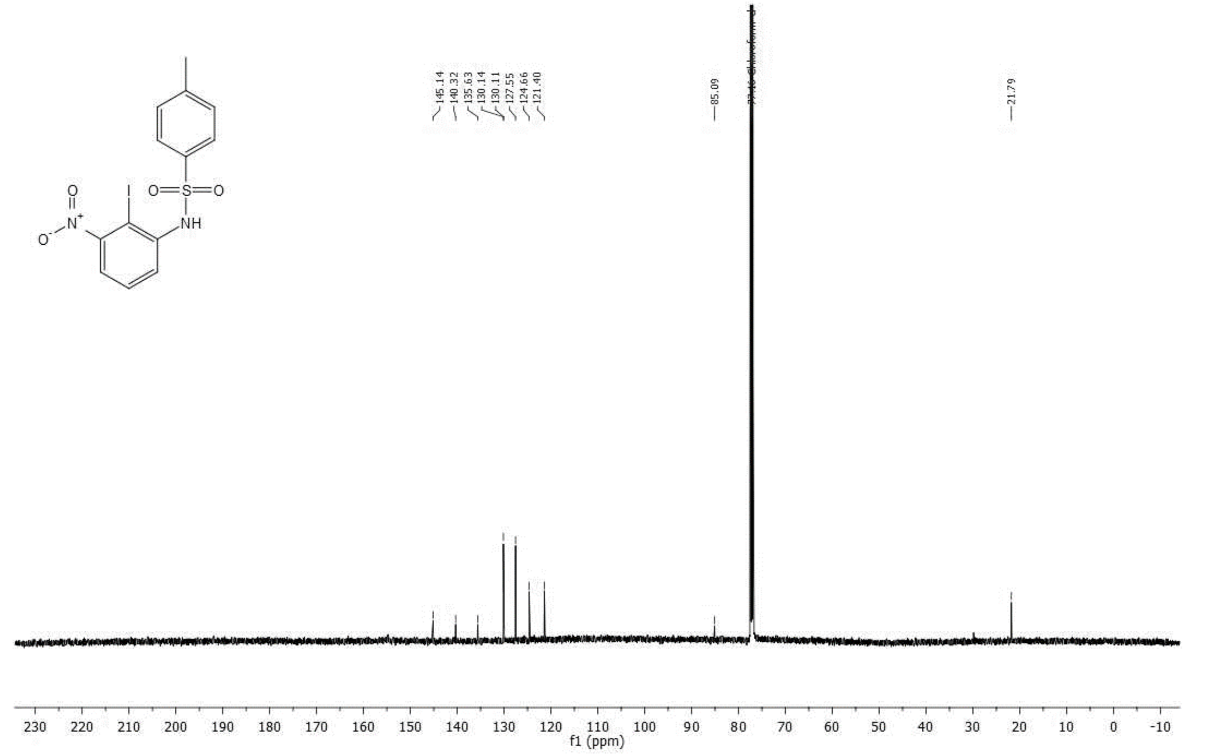

Methyl *N*-(2-iodophenyl)-*N*-tosyl-*D*-alaninate (13a)

^{1}H NMR (500 MHz, $CDCl_3$)

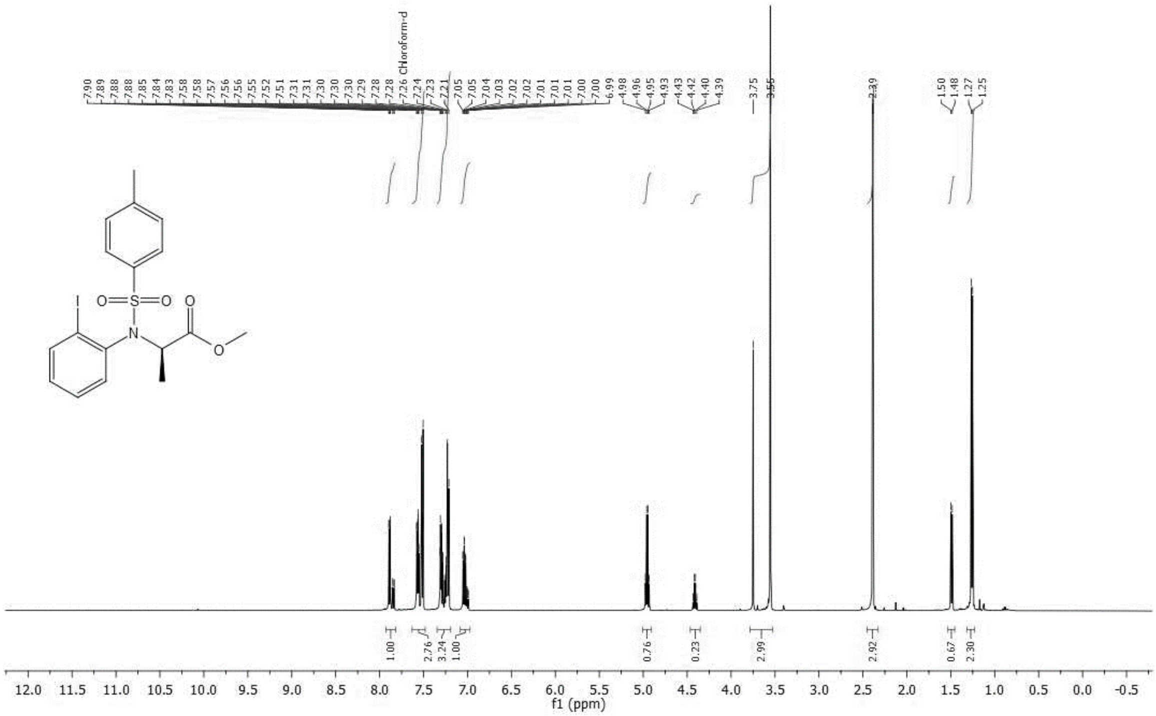

$^{13}C\{^{1}H\}$ NMR (126 MHz, $CDCl_3$)

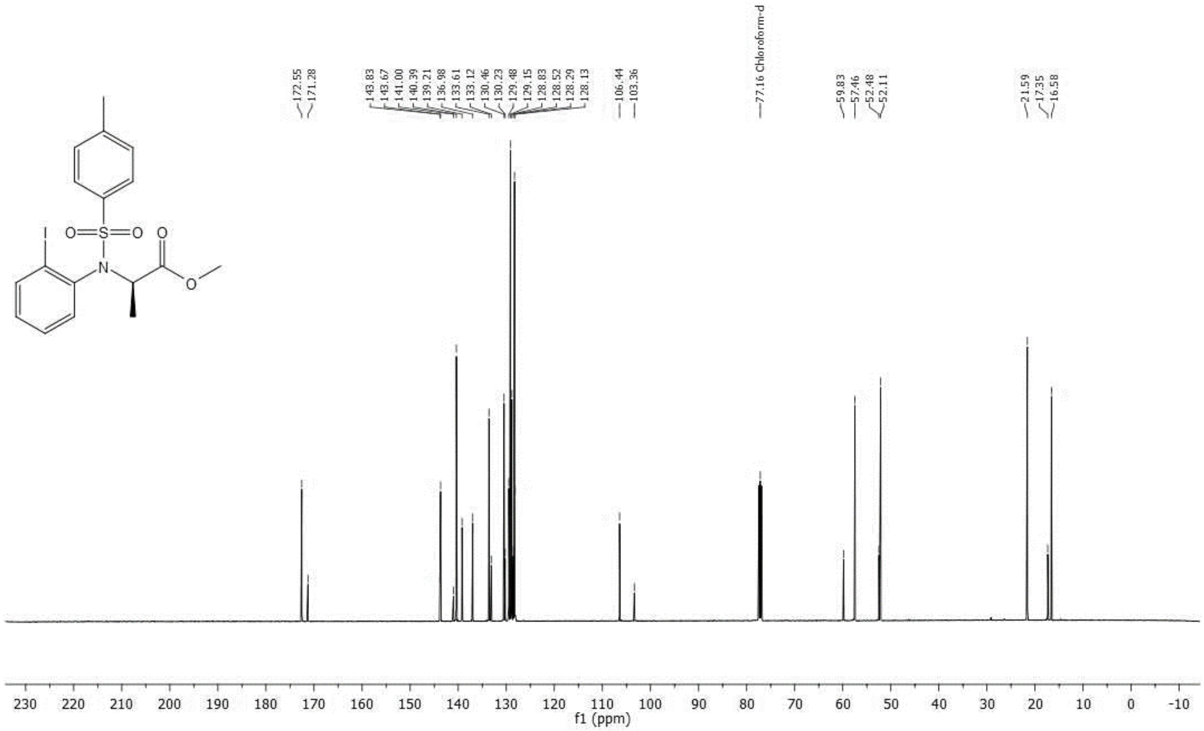

Ethyl *N*-(2-iodophenyl)-*N*-tosyl-*D*-alaninate (13b)

^{1}H NMR (500 MHz, $CDCl_3$)

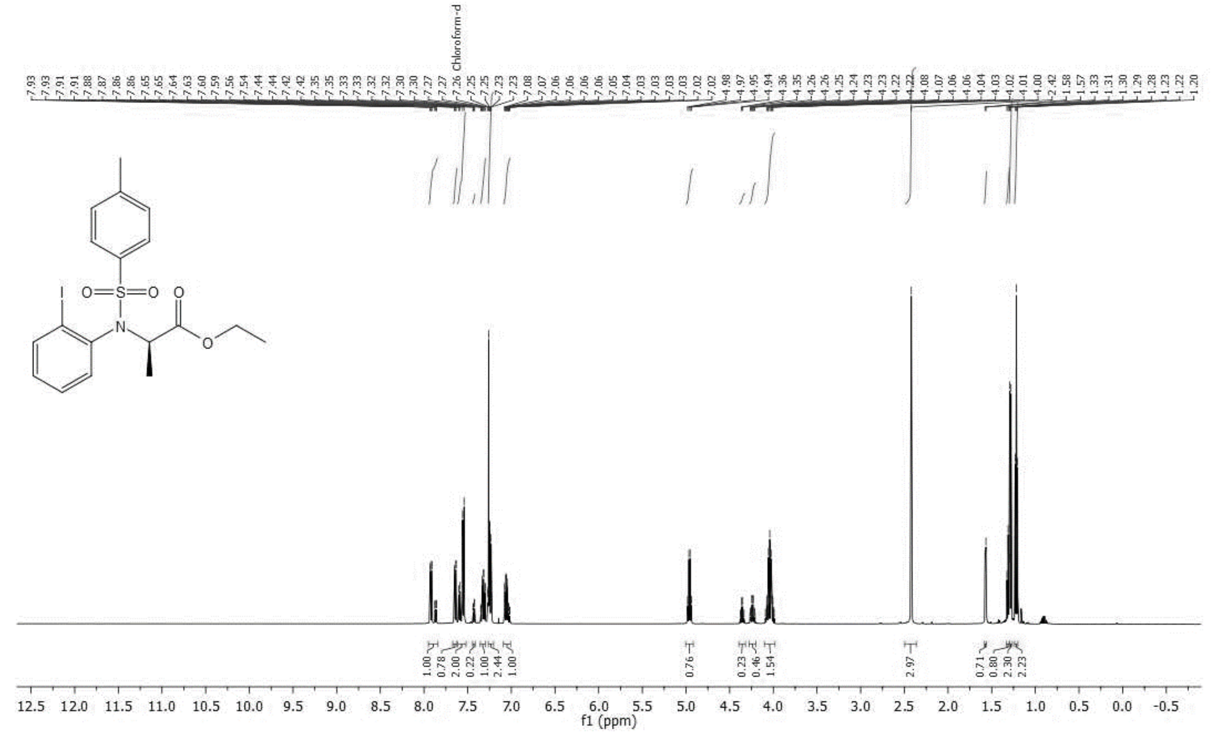

$^{13}C\{^{1}H\}$ NMR (126 MHz, $CDCl_3$)

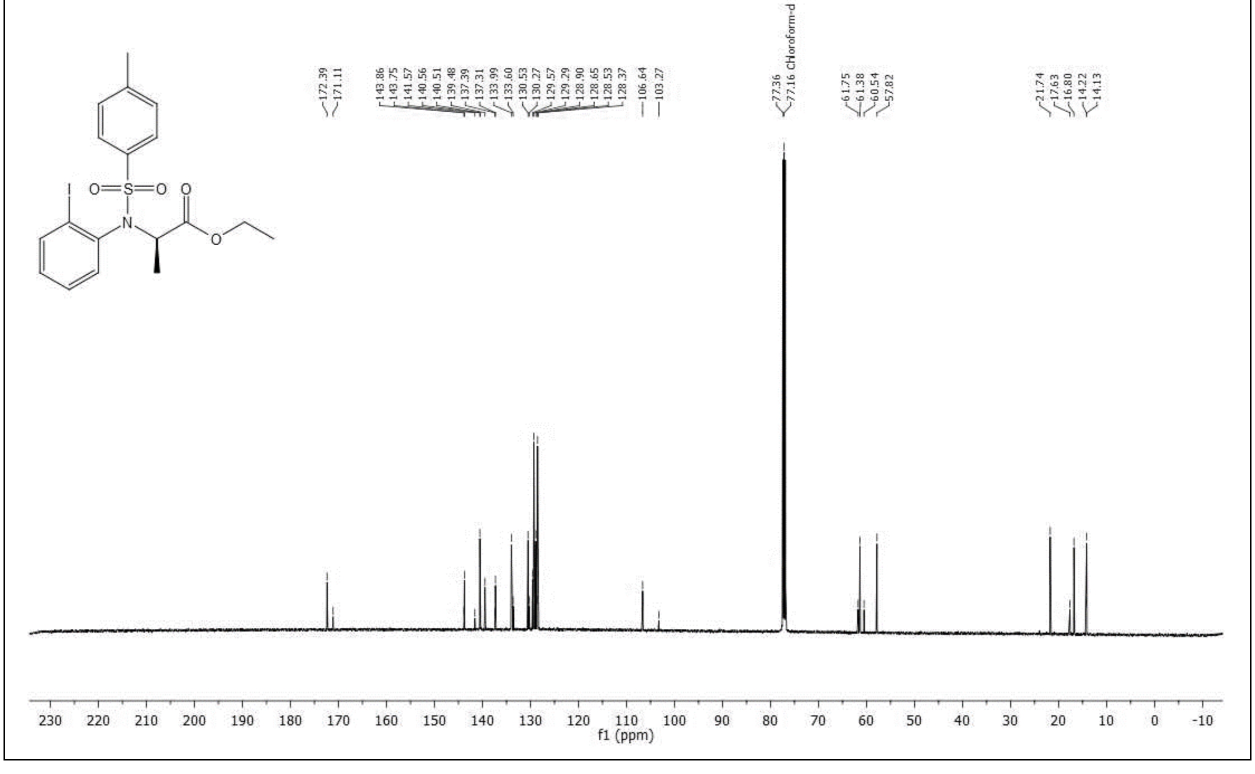

Methyl *N*-(2-iodophenyl)-*N*-(mesitylsulfonyl)-*D*-alaninate (13c)

^{1}H NMR (500 MHz, $CDCl_3$)

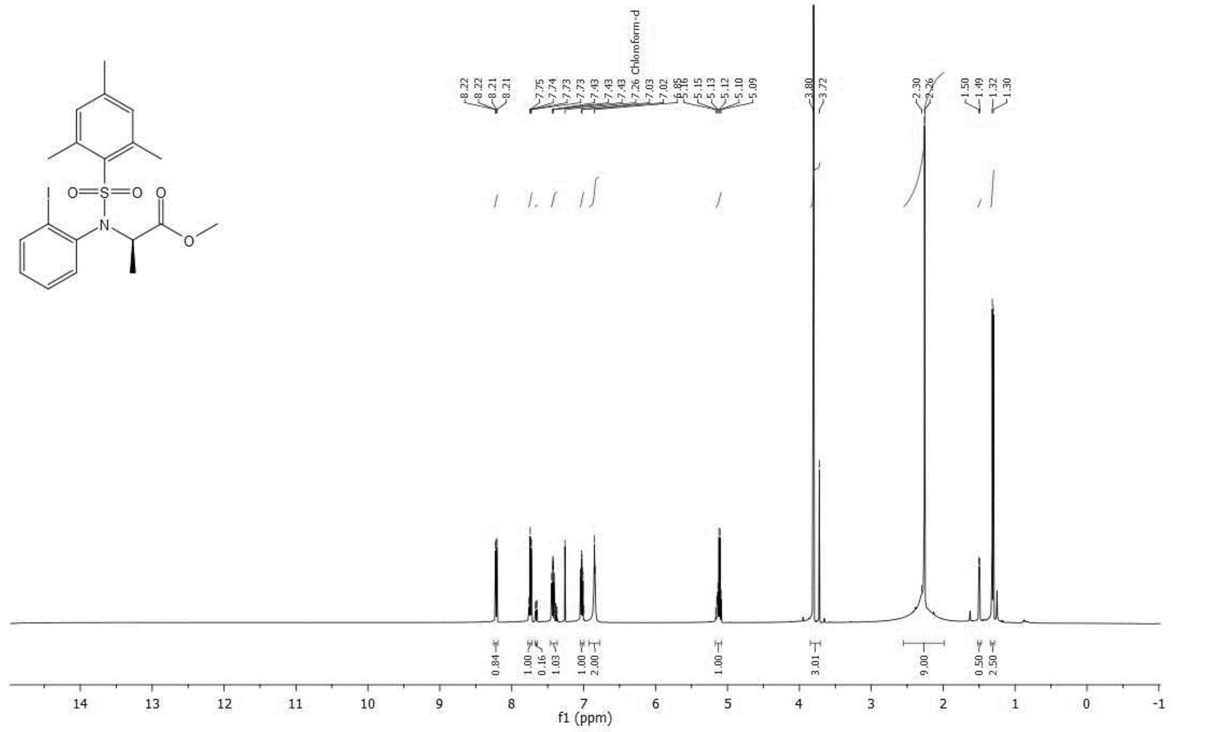

$^{13}C\{^{1}H\}$ NMR (126 MHz, $CDCl_3$)

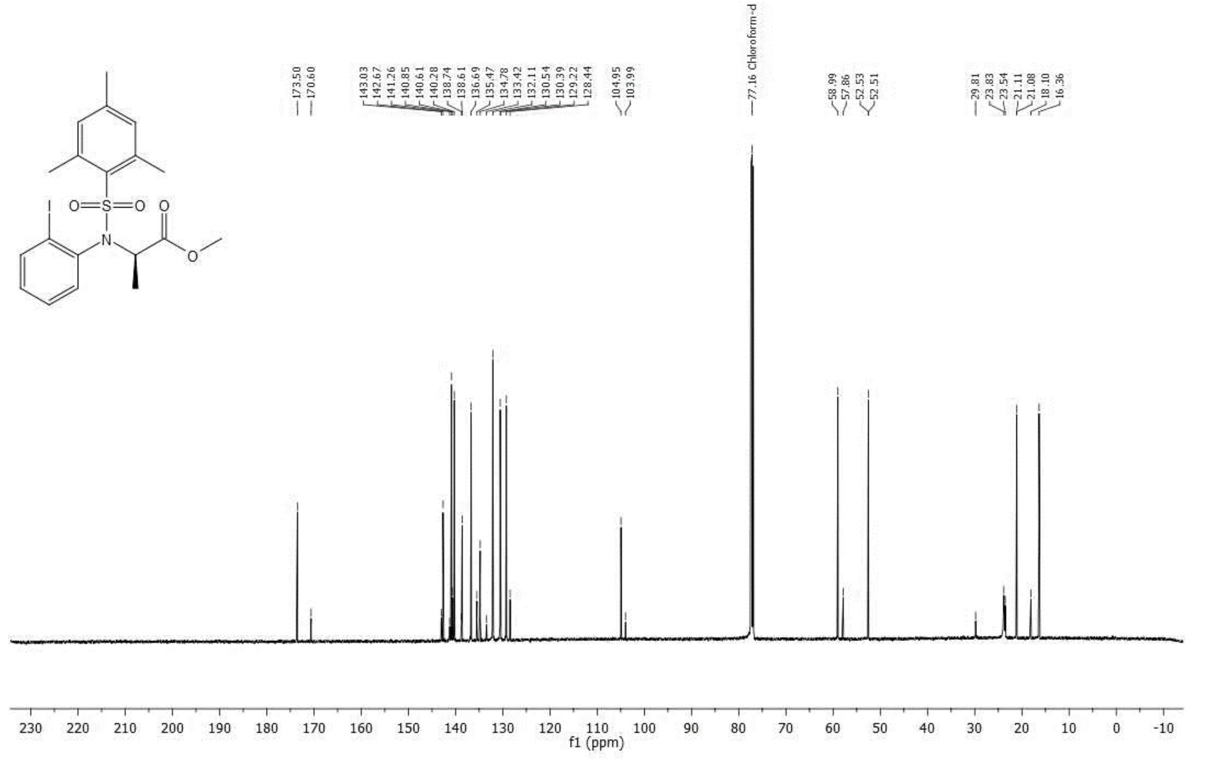

Methyl *N*-(2-iodophenyl)-*N*-(methylsulfonyl)-*D*-alaninate (13d)

^{1}H NMR (500 MHz, $CDCl_3$)

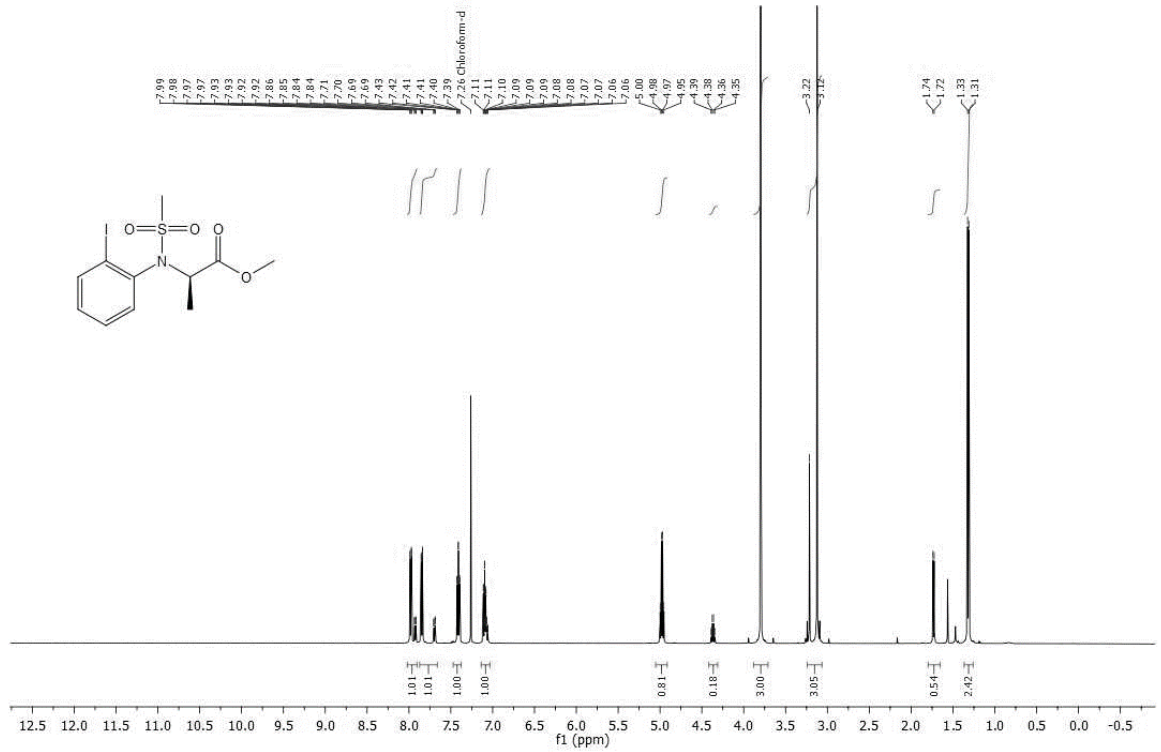

^{13}C{^{1}H} NMR (126 MHz, $CDCl_3$)

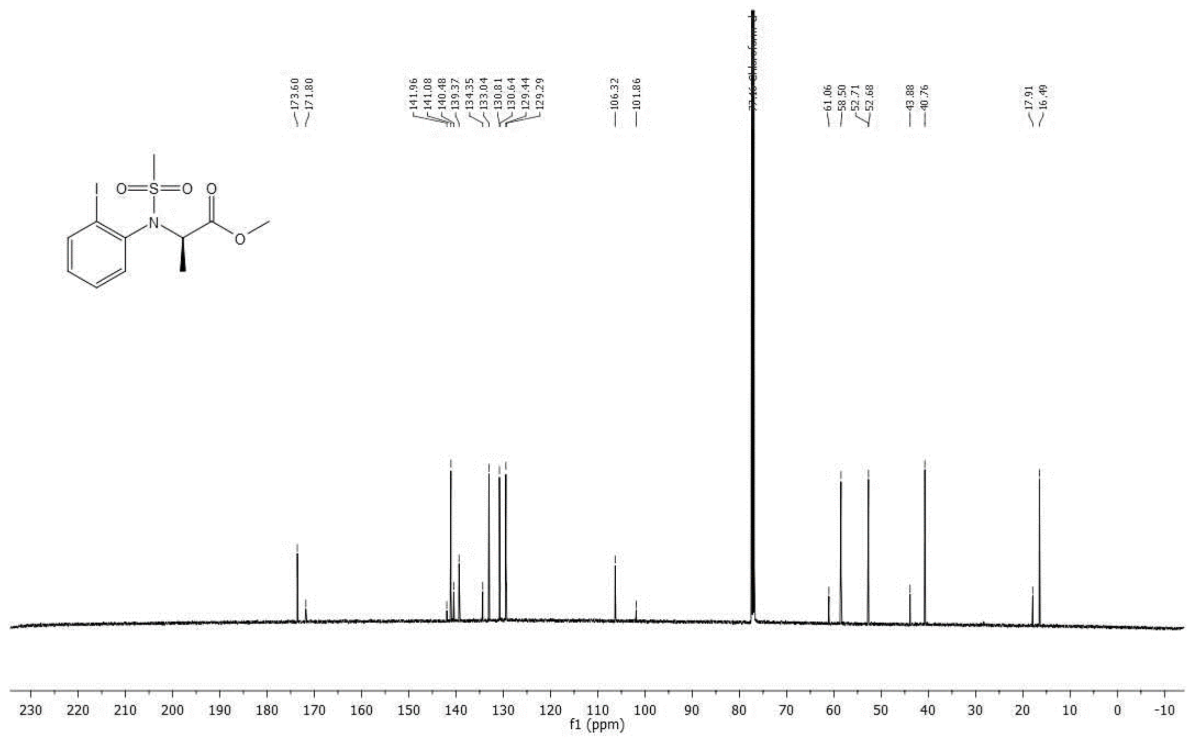

Methyl *N*-(2-iodophenyl)-*N*-((4-(trifluoromethyl)phenyl)sulfonyl)-*D*-alaninate (13e)

^{1}H NMR (500 MHz, $CDCl_3$)

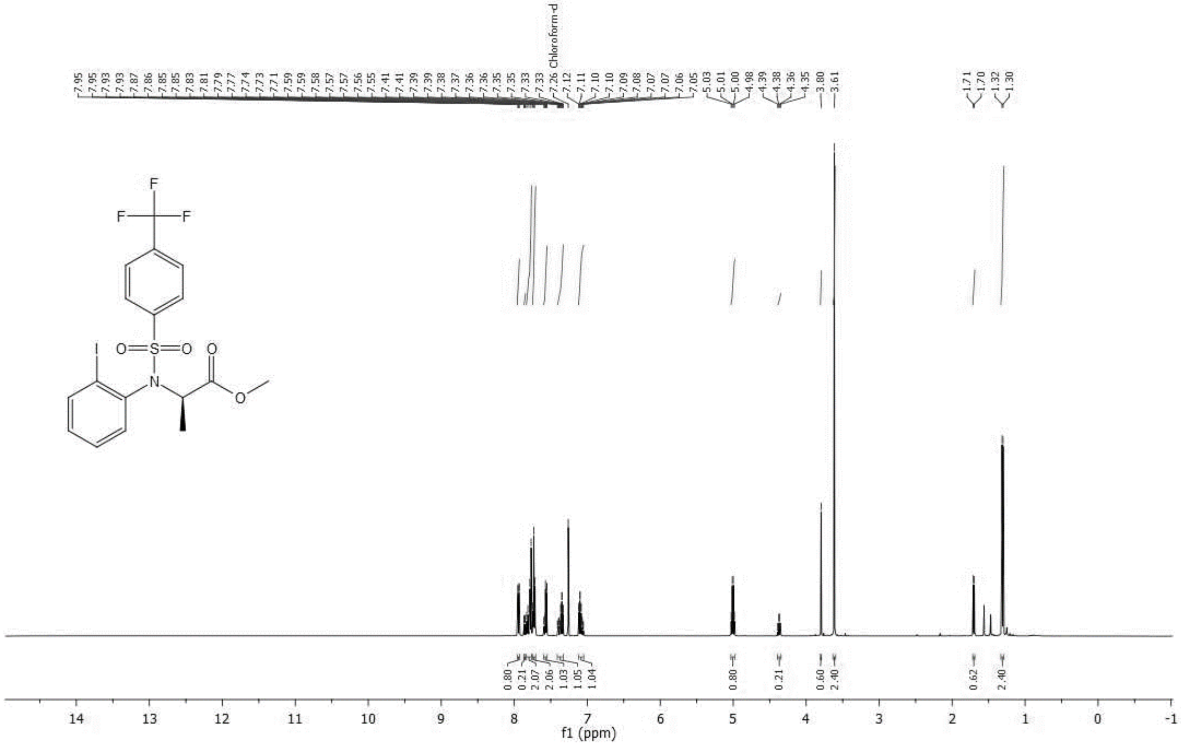

$^{13}C\{^{1}H\}$ NMR (126 MHz, $CDCl_3$)

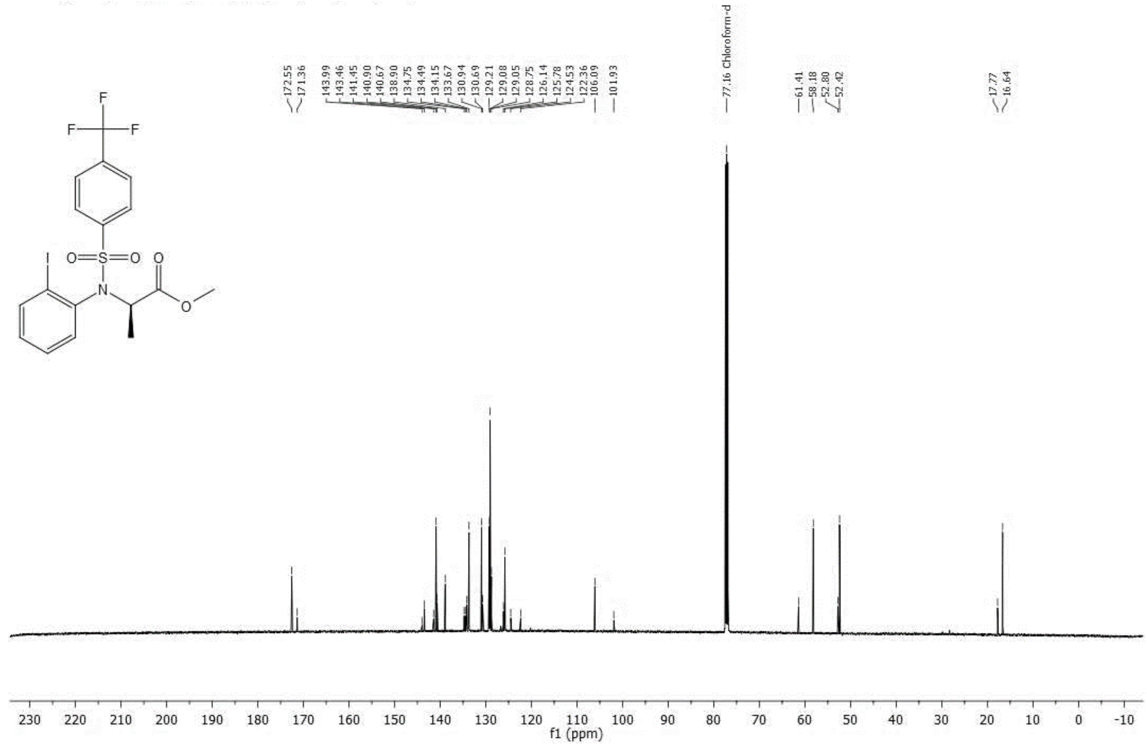

Methyl *N*-(2-iodophenyl)-*N*-((4-nitrophenyl)sulfonyl)-*D*-alaninate (13f)

^{1}H NMR (500 MHz, $CDCl_3$)

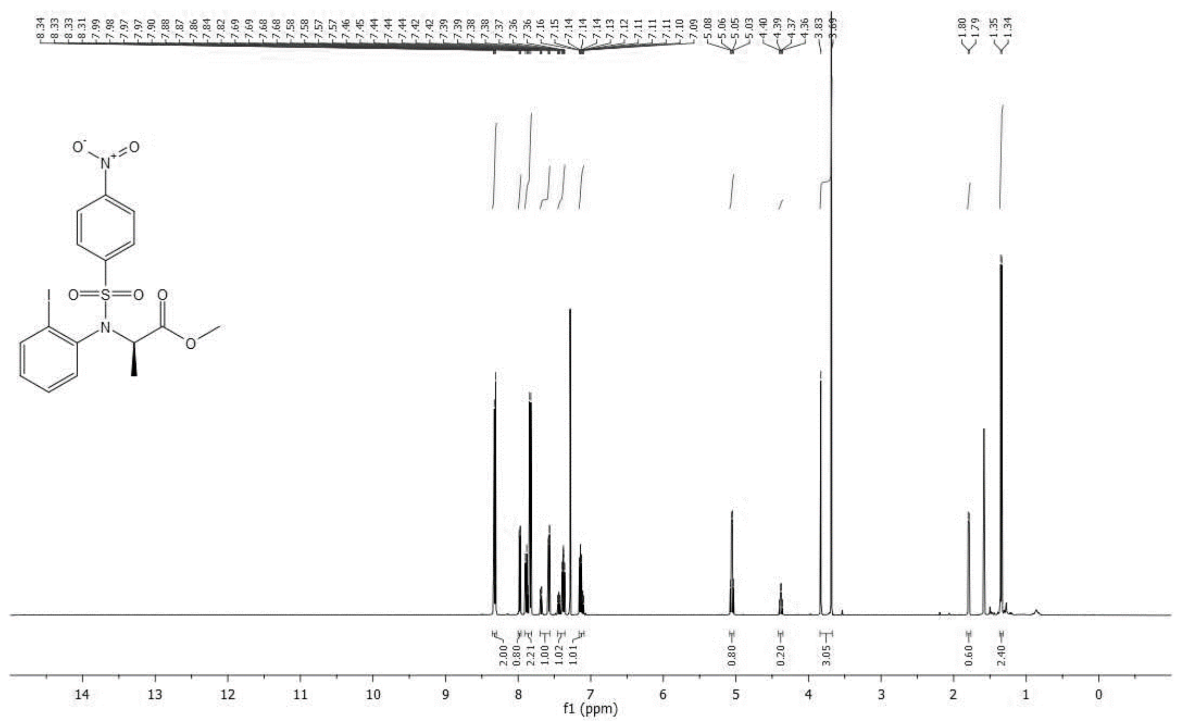

$^{13}C\{^{1}H\}$ NMR (126 MHz, $CDCl_3$)

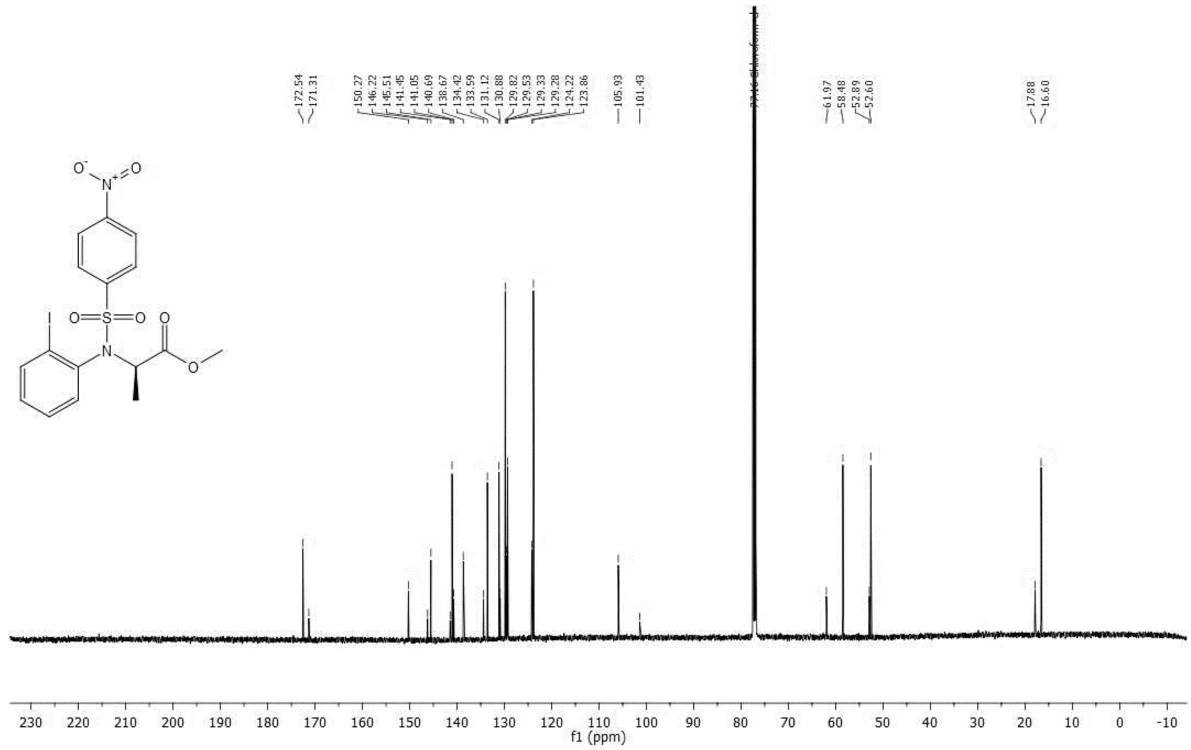

Methyl *N*-(2-iodophenyl)-*N*-((trifluoromethyl)sulfonyl)-*D*-alaninate (13g)

^{1}H NMR (500 MHz, MeOD)

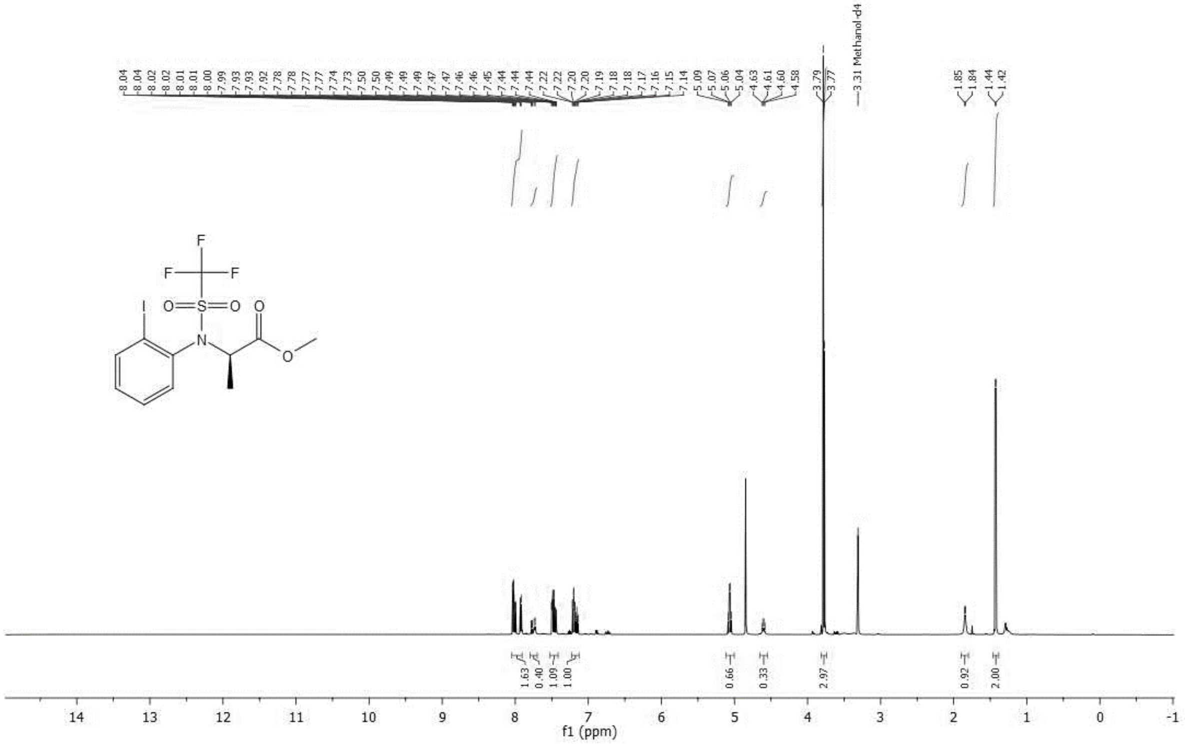

^{13}C{^{1}H} NMR (126 MHz, MeOD)

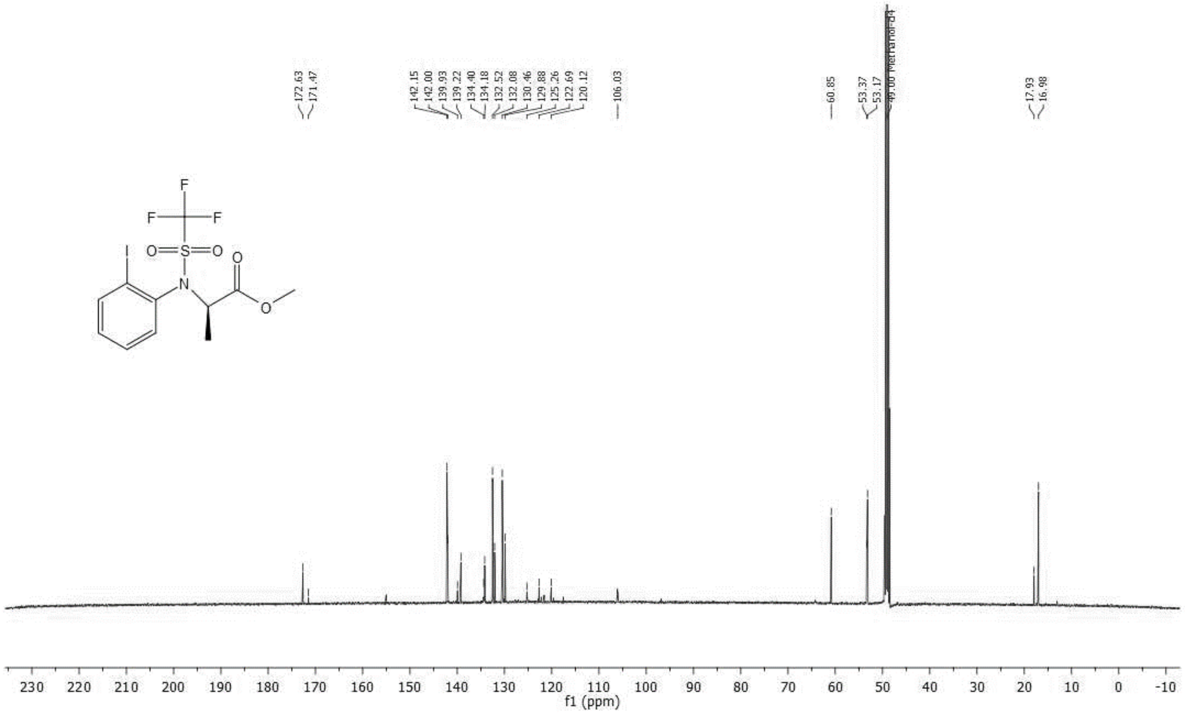

Methyl *N*-(2-Iodo-3-methoxyphenyl)-*N*-tosyl-*D*-alaninate (13h)

^{1}H NMR (500 MHz, $CDCl_3$)

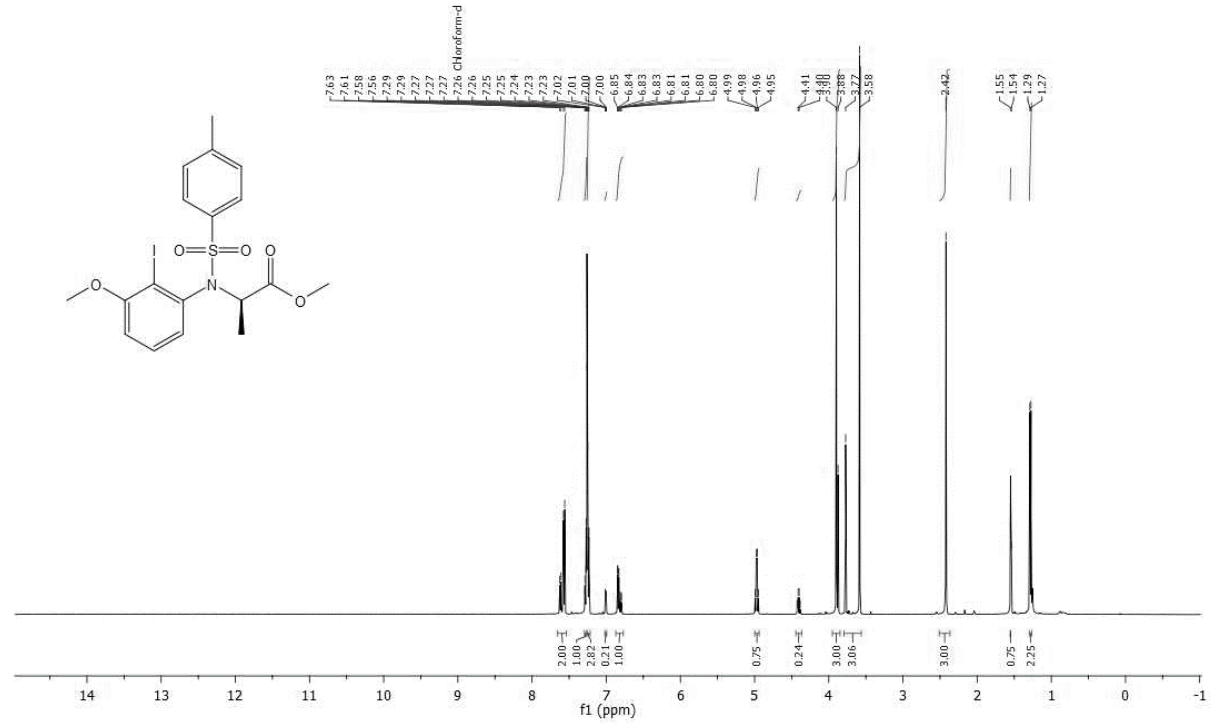

^{13}C{^{1}H} NMR (126 MHz, $CDCl_3$)

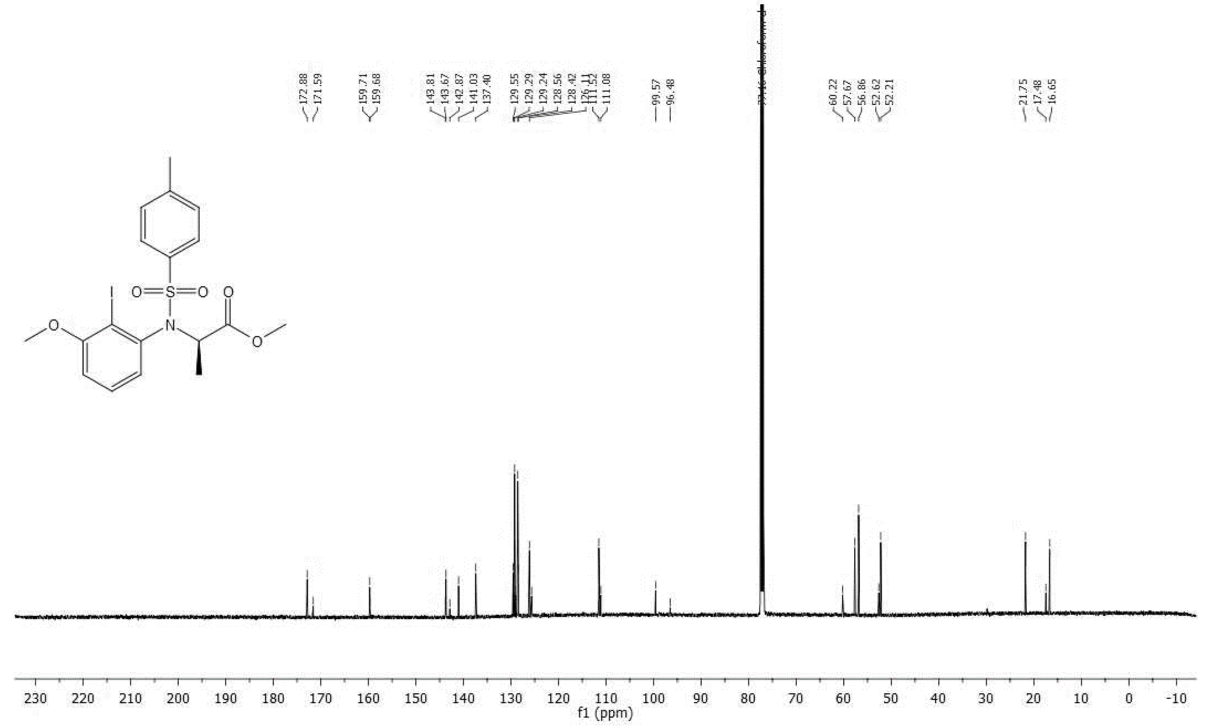

Methyl *N*-(2-iodo-3-nitrophenyl)-*N*-tosyl-*D*-alaninate (13i)

^{1}H NMR (500 MHz, $CDCl_3$)

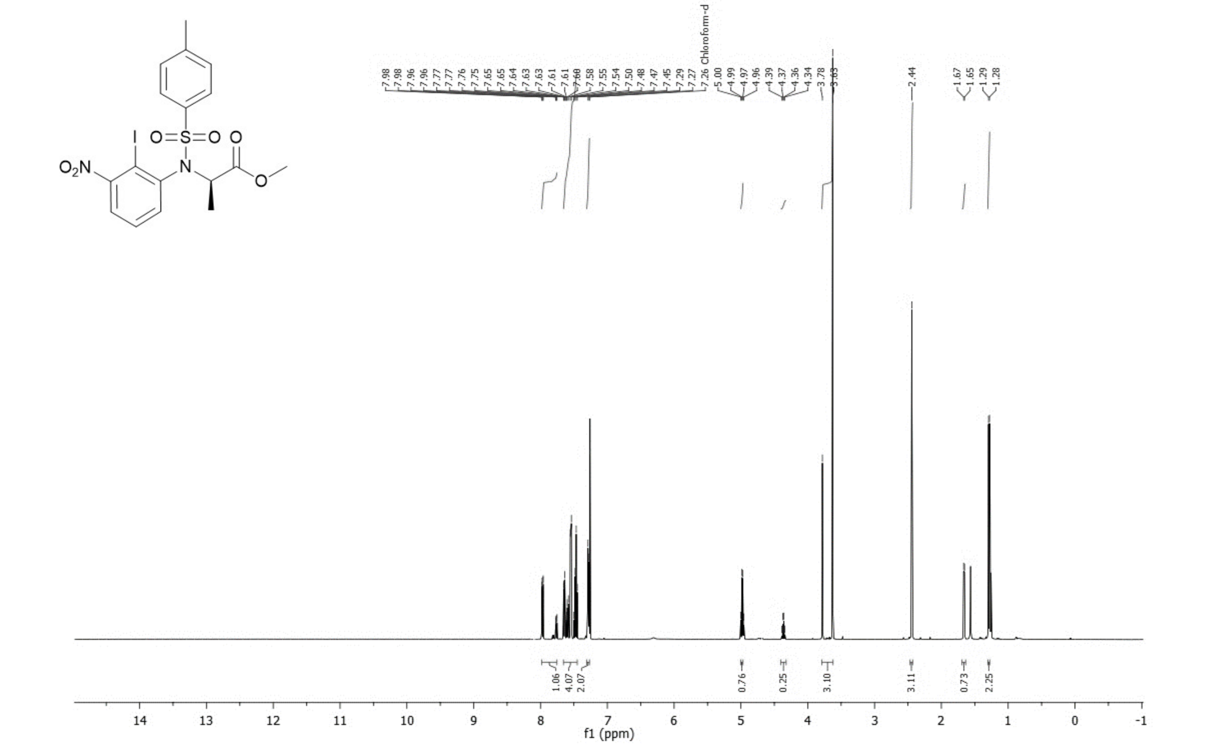

$^{13}C\{^{1}H\}$ NMR (126 MHz, $CDCl_3$)

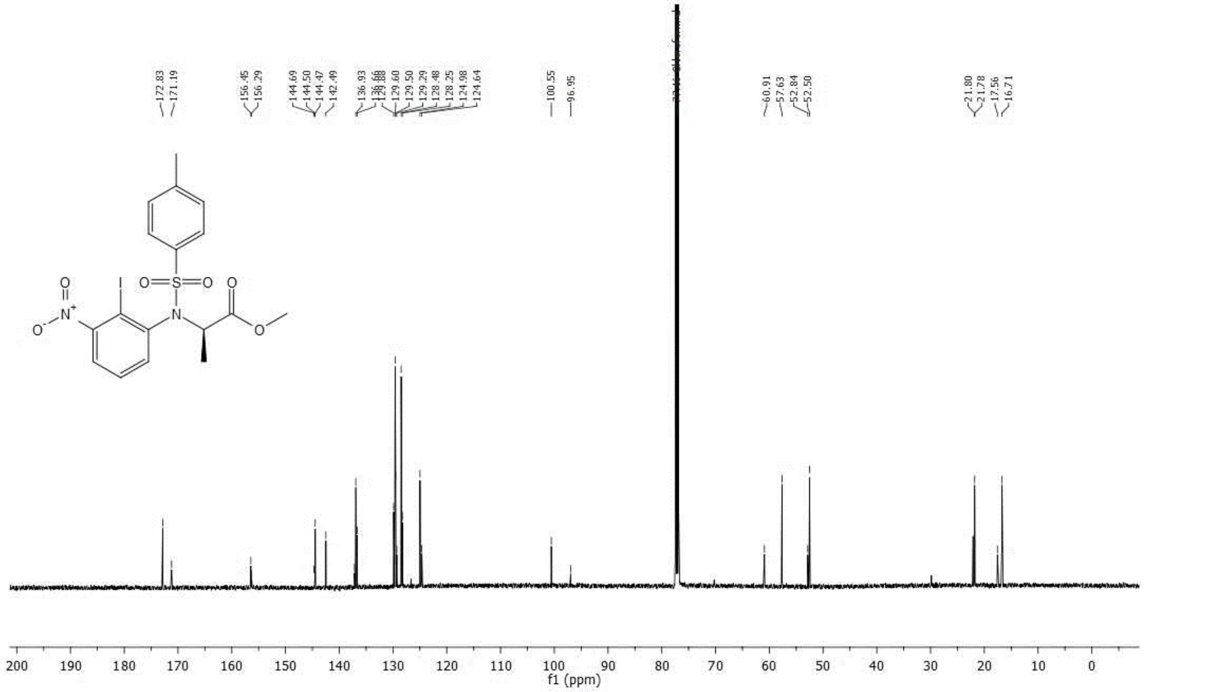

Ethyl *N*-(2-iodo-3-nitrophenyl)-*N*-tosyl-*D*-alaninate (13j)

^{1}H NMR (300 MHz, $CDCl_3$)

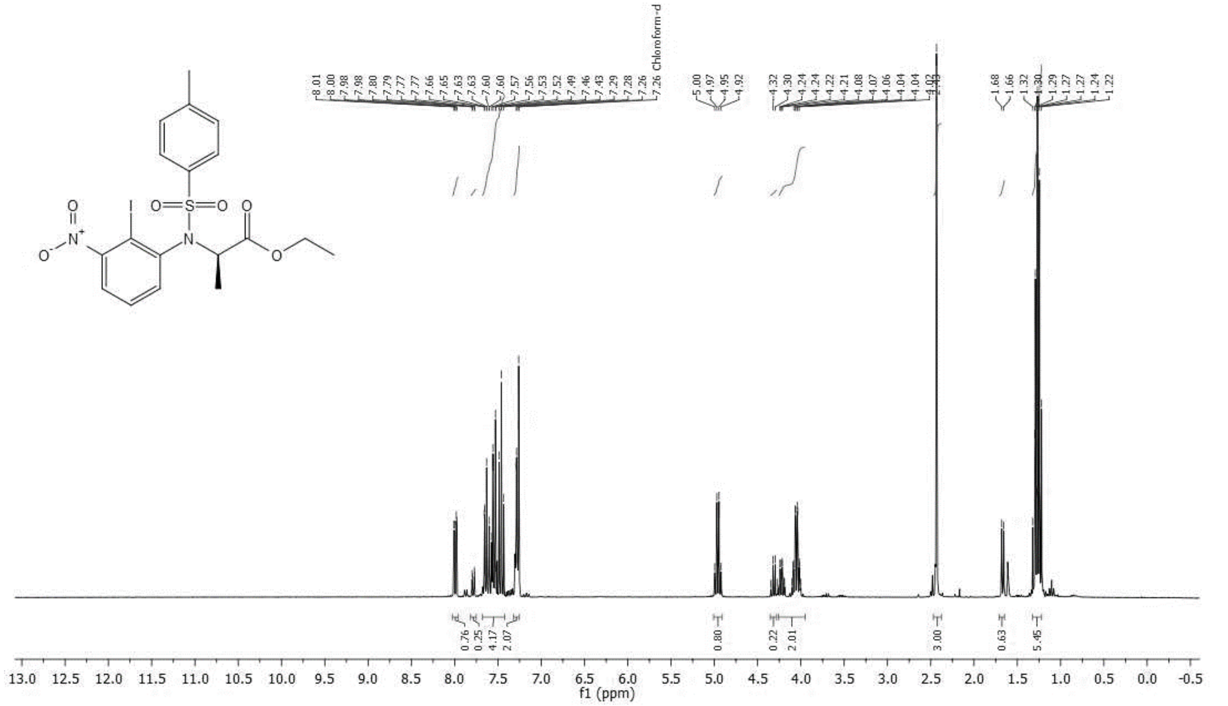

$^{13}C\{^{1}H\}$ NMR (75 MHz, $CDCl_3$)

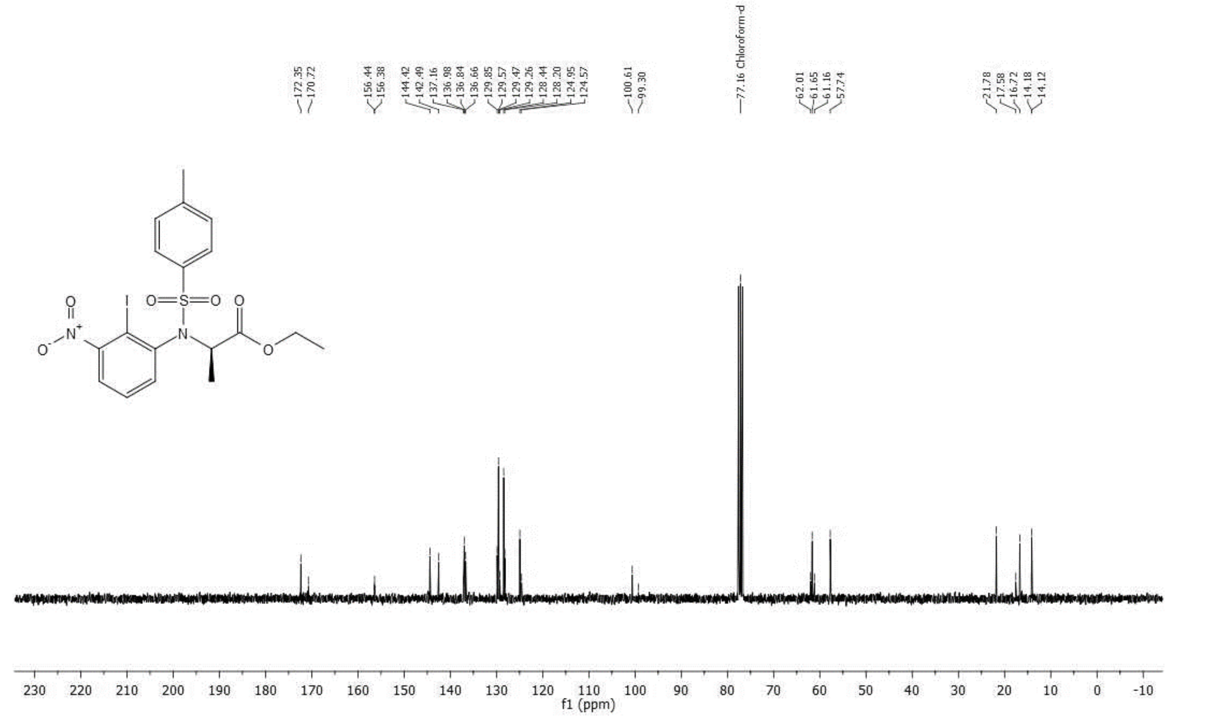

**3-Amino-2-iodophenol (15)**
^{1}H NMR (400 MHz, $CDCl_3$)

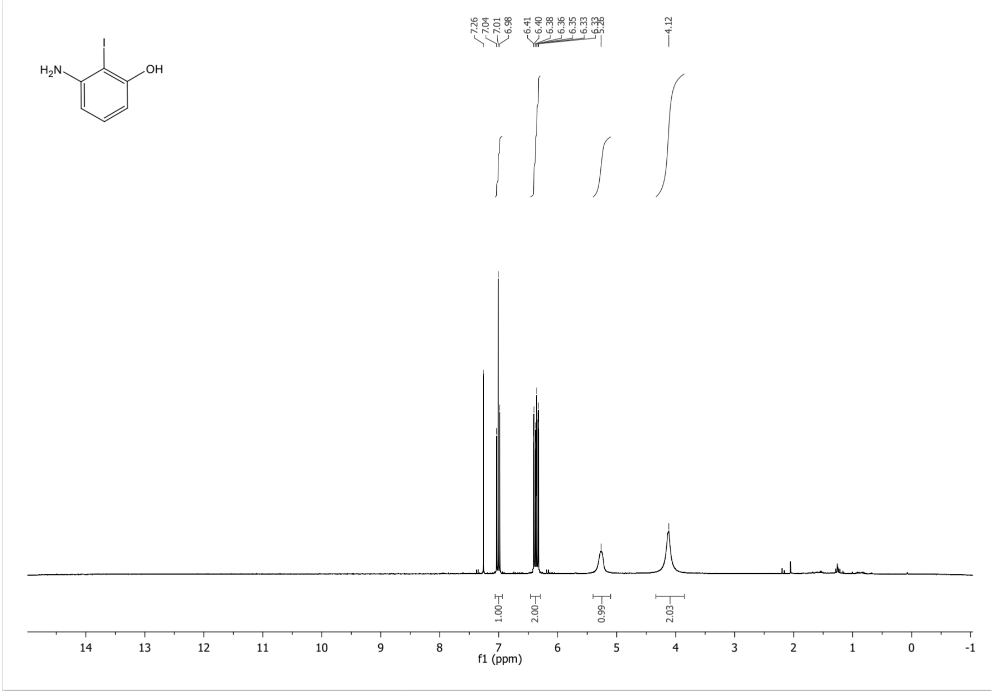

$^{13}C\{^{1}H\}$ NMR (100 MHz, $CDCl_3$)

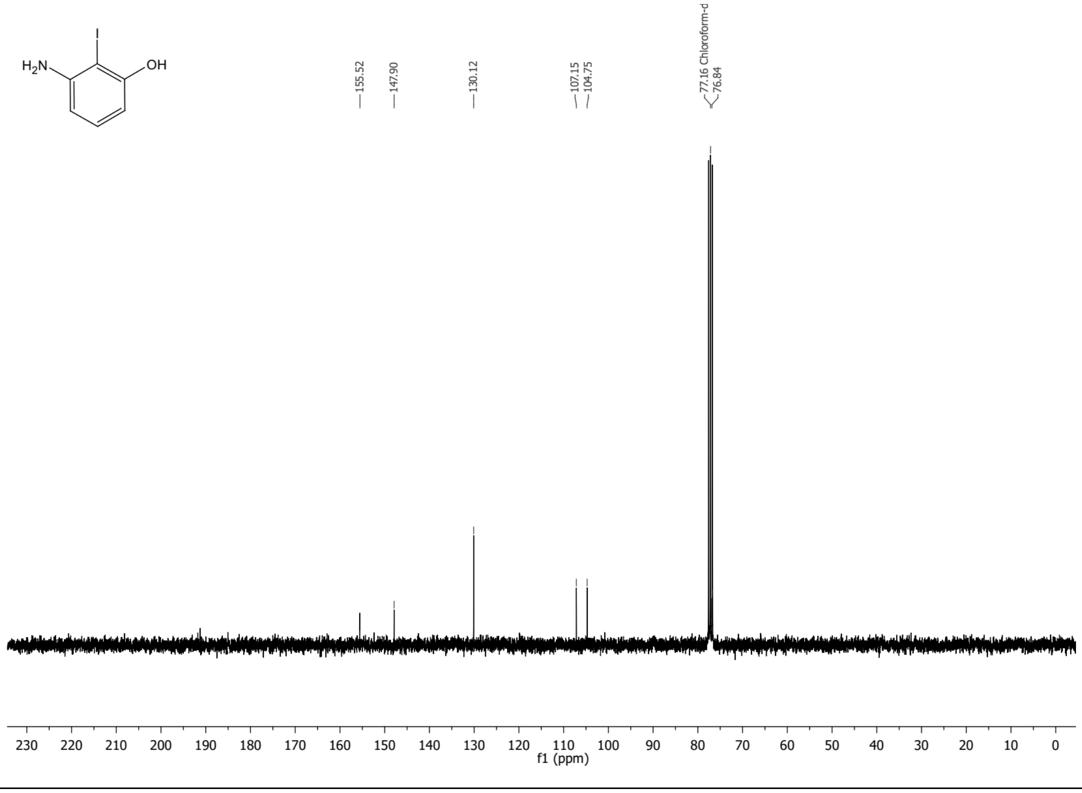

N-(3-Amino-2-iodophenyl)-4-methylbenzenesulfonamide (16)
^{1}H NMR (300 MHz, $CDCl_3$)
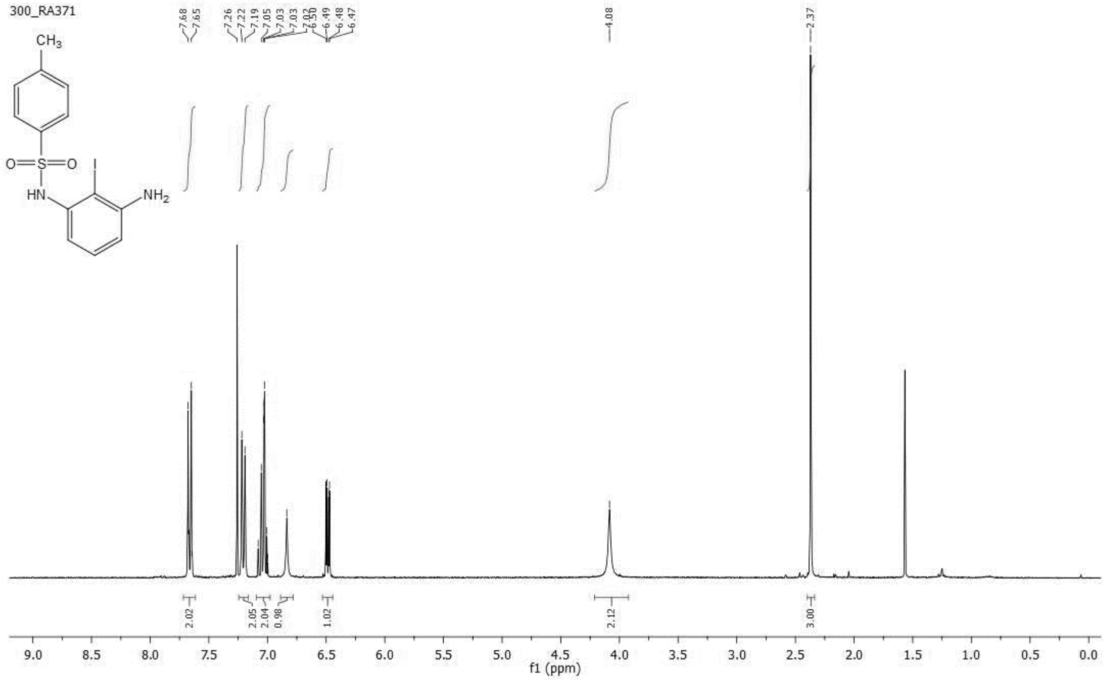
$^{13}C\{^{1}H\}$ NMR (75 MHz, $CDCl_3$)
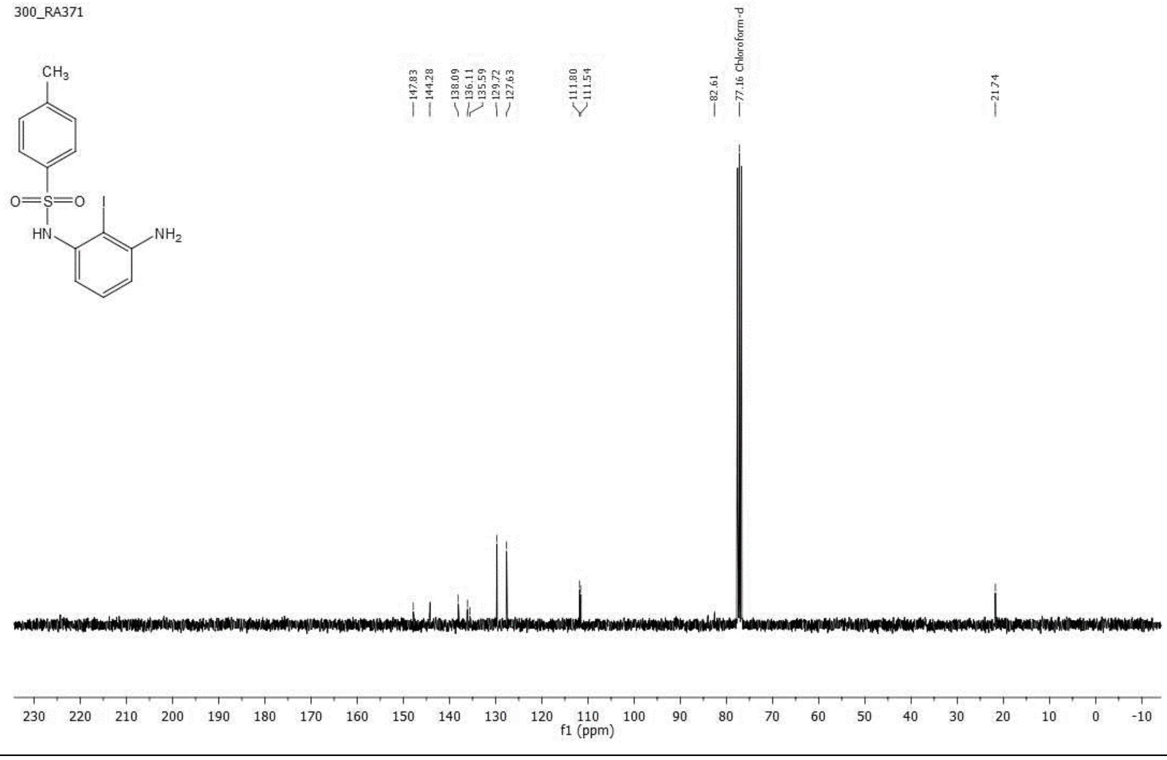

N-(3-hydroxy-2-iodophenyl)-4-methylbenzenesulfonamide (17a)

^{1}H NMR (500 MHz, CDCl$_3$)

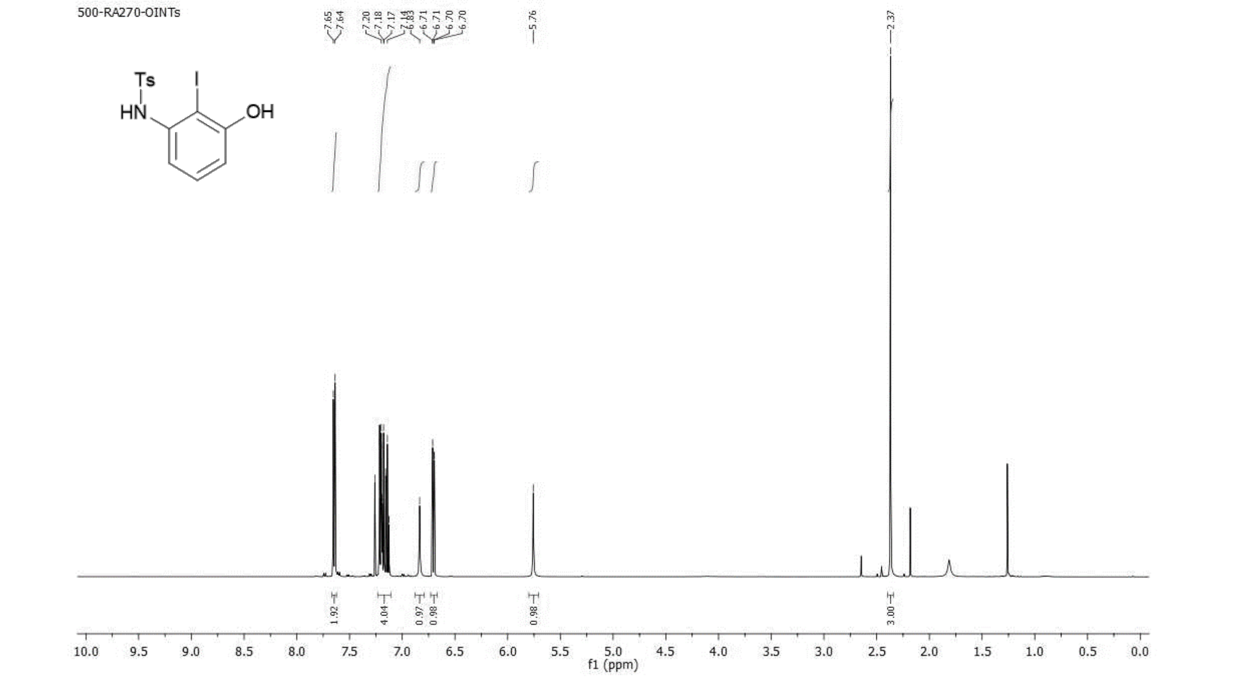

^{13}C{^{1}H} NMR (126 MHz, CDCl$_3$)

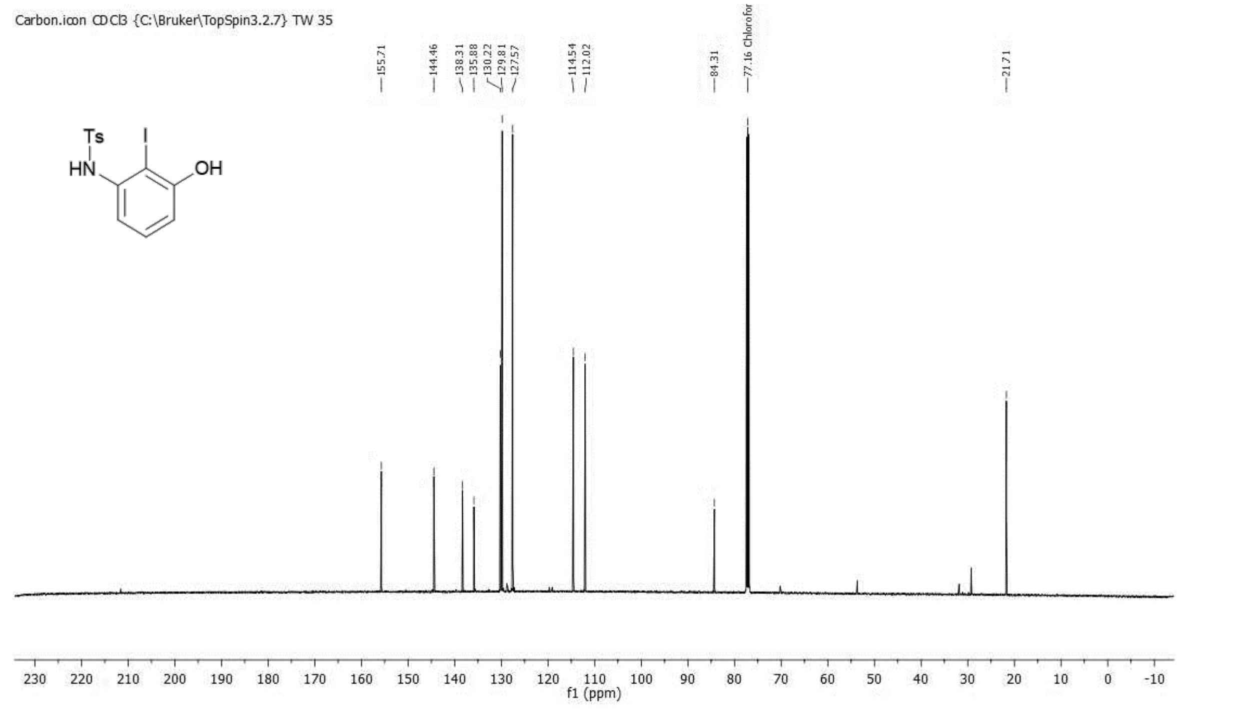

_N,N′_-(2-Iodo-1,3-phenylene)bis(4-methylbenzenesulfonamide) (17b)

^{1}H NMR (500 MHz, $CDCl_3$)

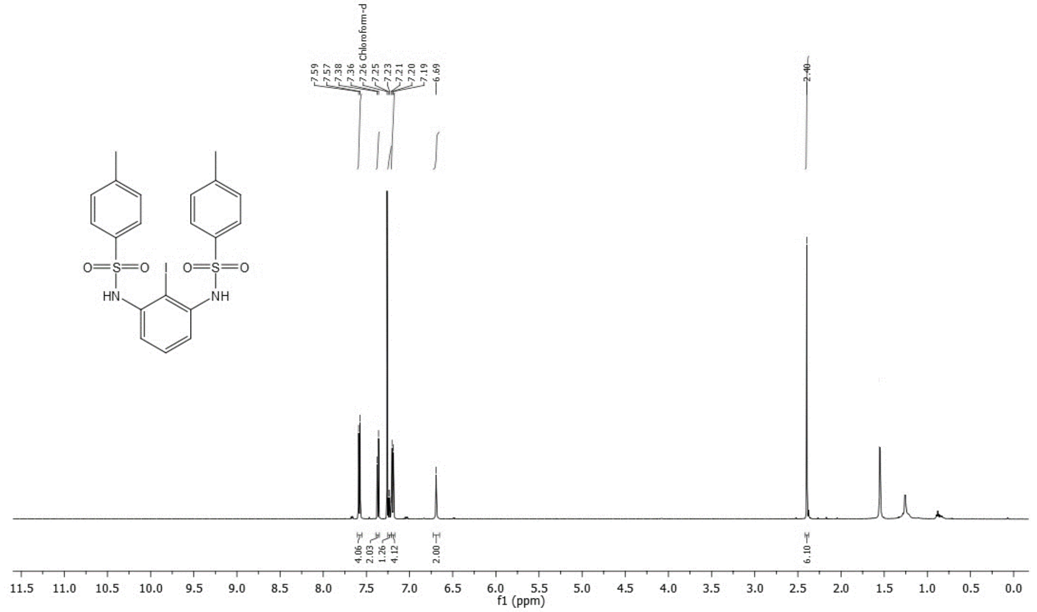

^{13}C{^{1}H} NMR (126 MHz, $CDCl_3$)

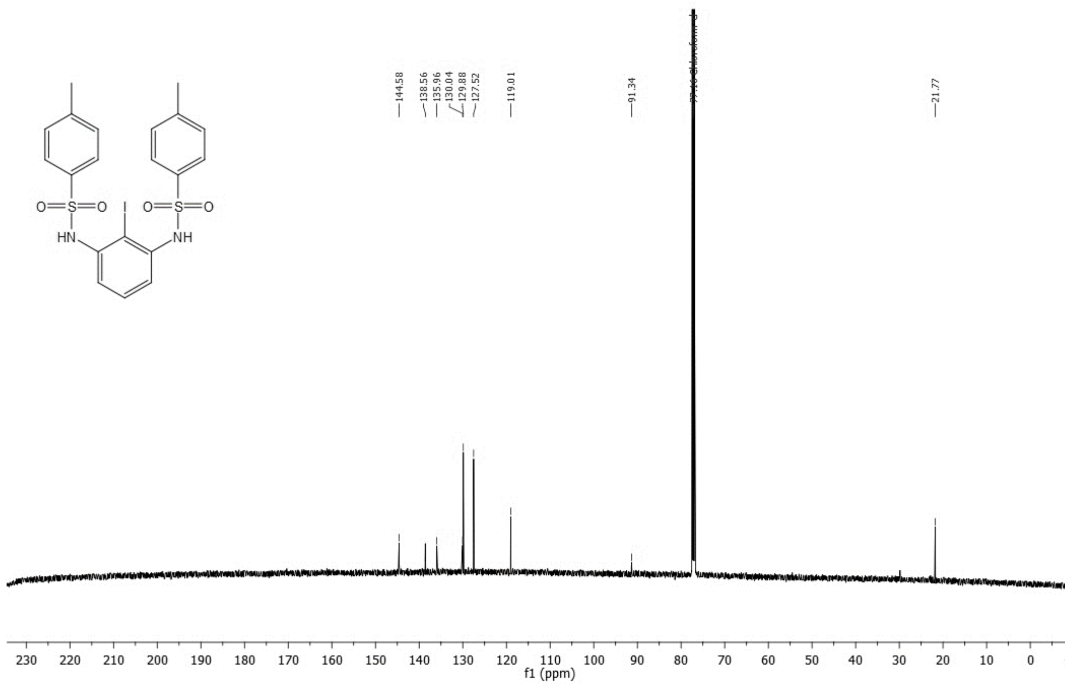

Methyl *N*-(2-iodo-3-(((*R*)-1-methoxy-1-oxopropan-2-yl)oxy)phenyl)-*N*-tosyl-*D*-alaninate (18a)

^{1}H NMR (300 MHz, $CDCl_3$)

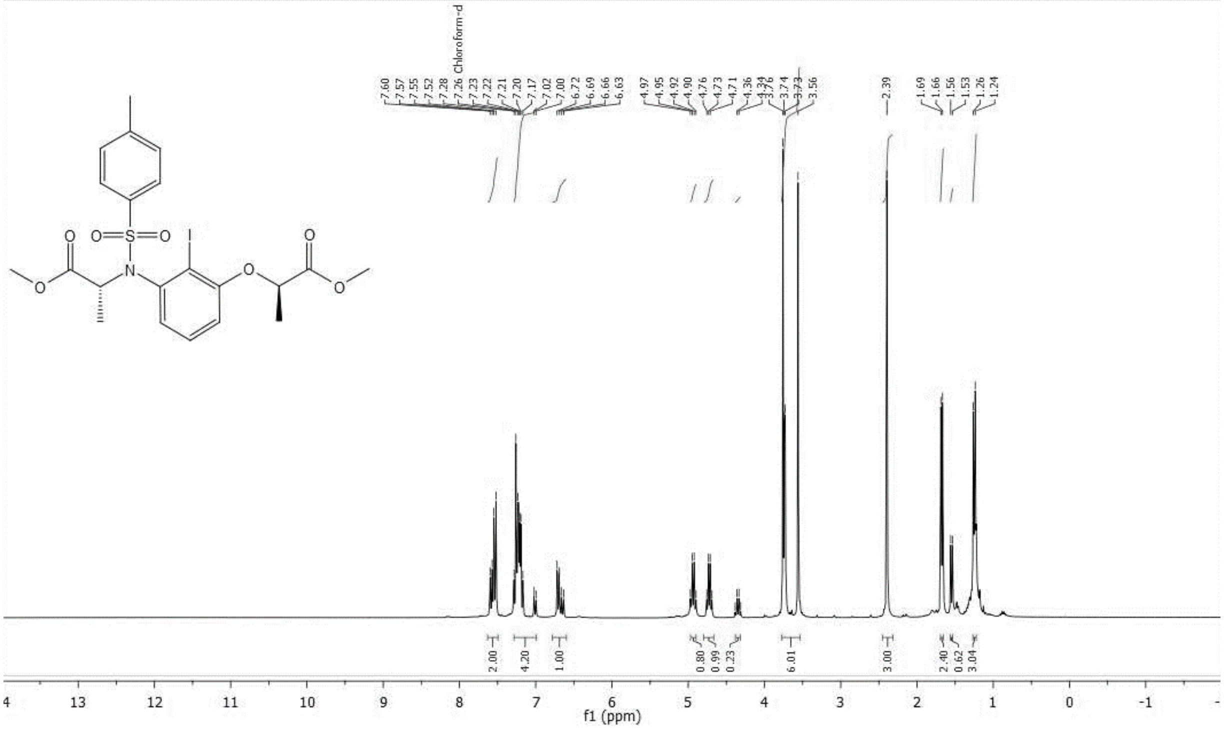

$^{13}C\{^{1}H\}$ NMR (126 MHz, $CDCl_3$)

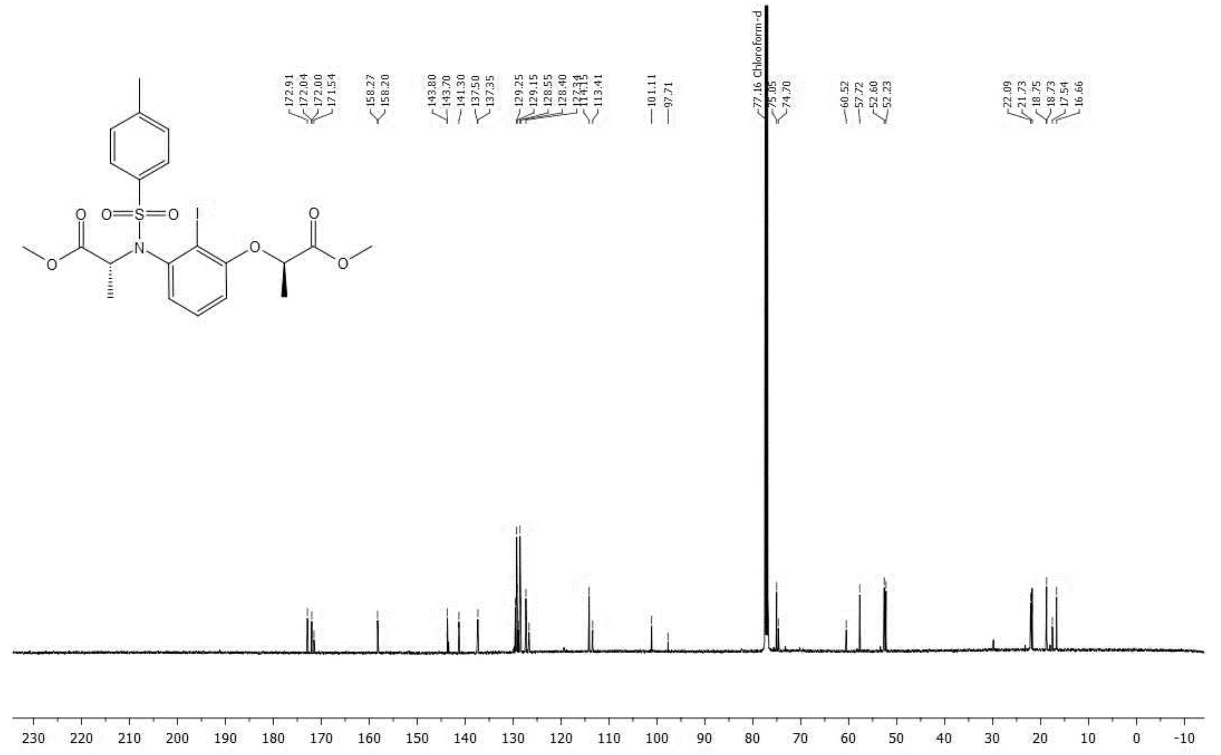

Ethyl (*R*)-2-(3-((*N*-((*R*)-1-ethoxy-1-oxopropan-2-yl)-4-methylphenyl)sulfonamido)-2-iodophenoxy)propanoate (18b)

^{1}H NMR (500 MHz, $CDCl_3$)

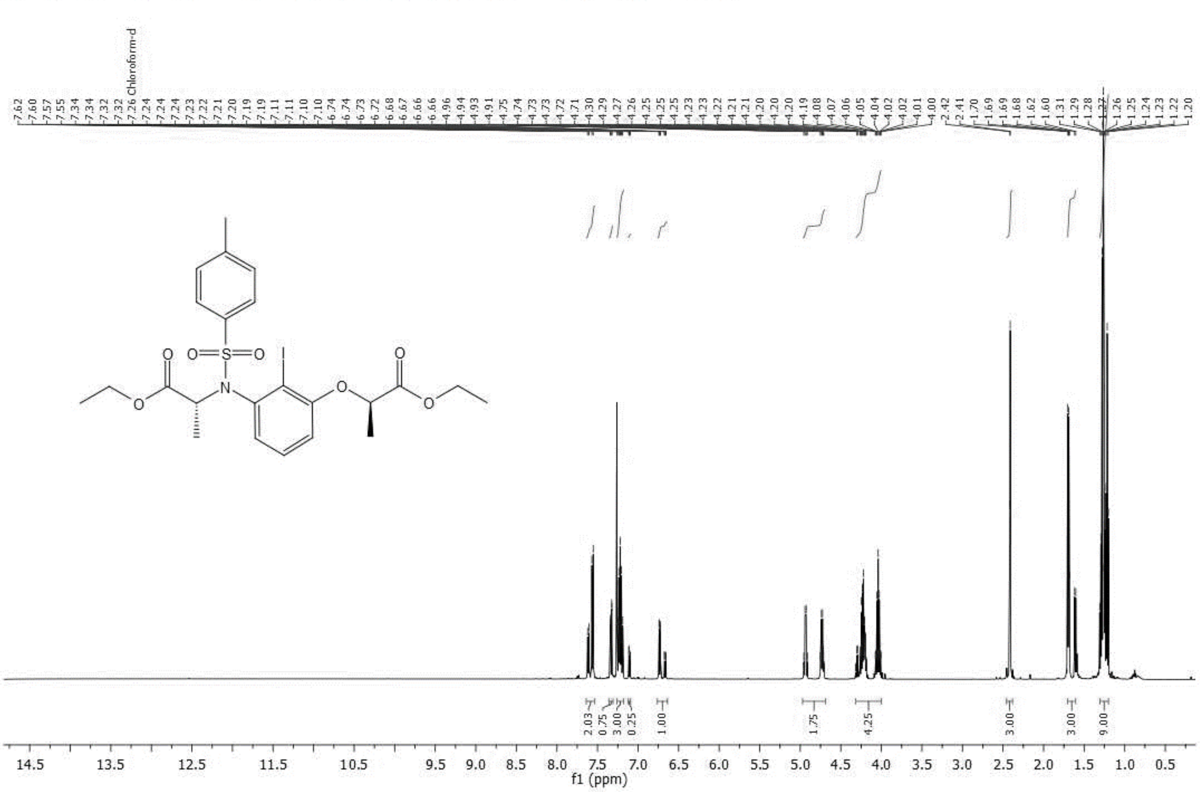

$^{13}C\{^{1}H\}$ NMR (126 MHz, $CDCl_3$)

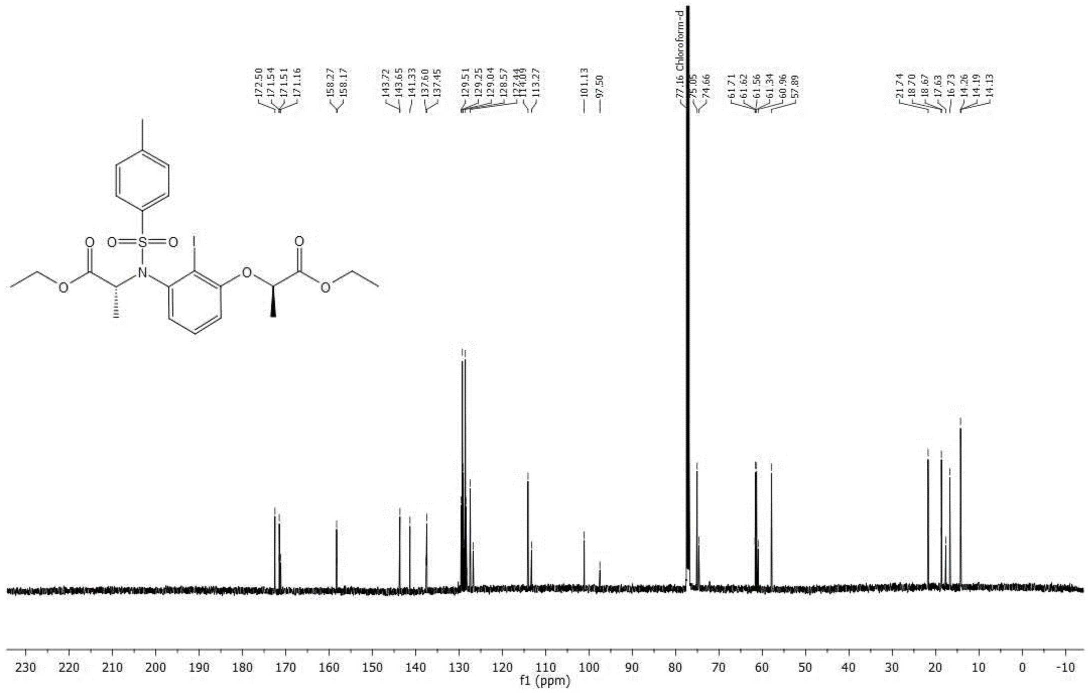

Dimethyl 2,2'-((2-iodo-1,3-phenylene)bis(tosylazanediyl))(2*R*,2'*R*)-dipropionate (18c)

^{1}H NMR (500 MHz, $CDCl_3$)

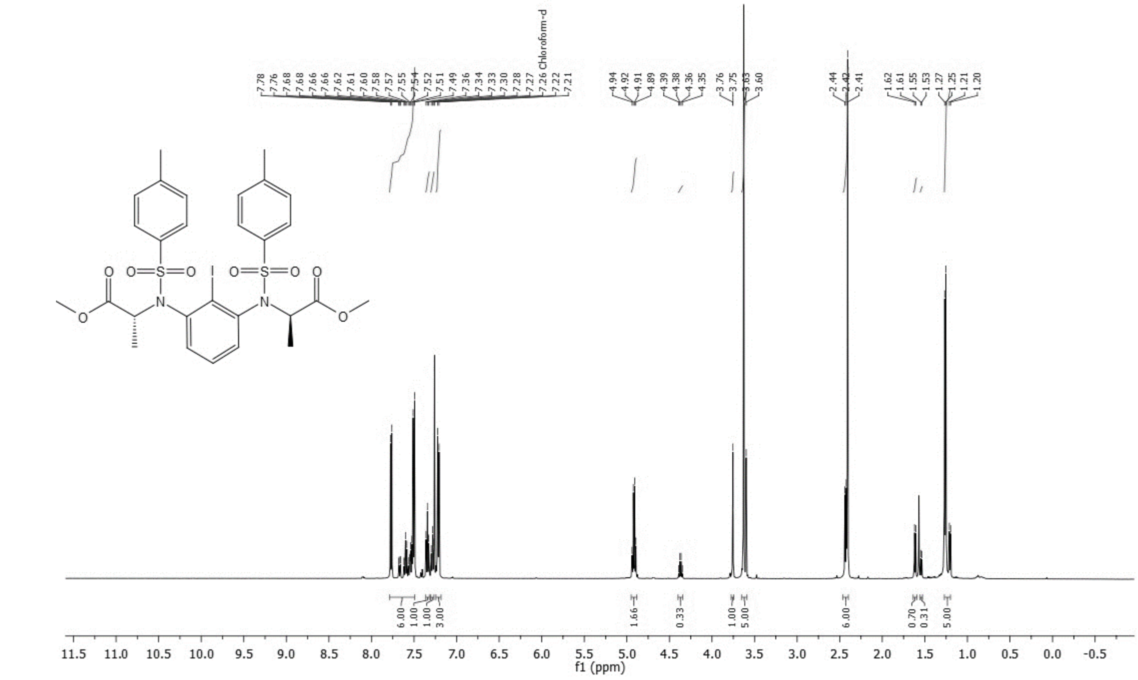

^{13}C{^{1}H} NMR (126 MHz, $CDCl_3$)

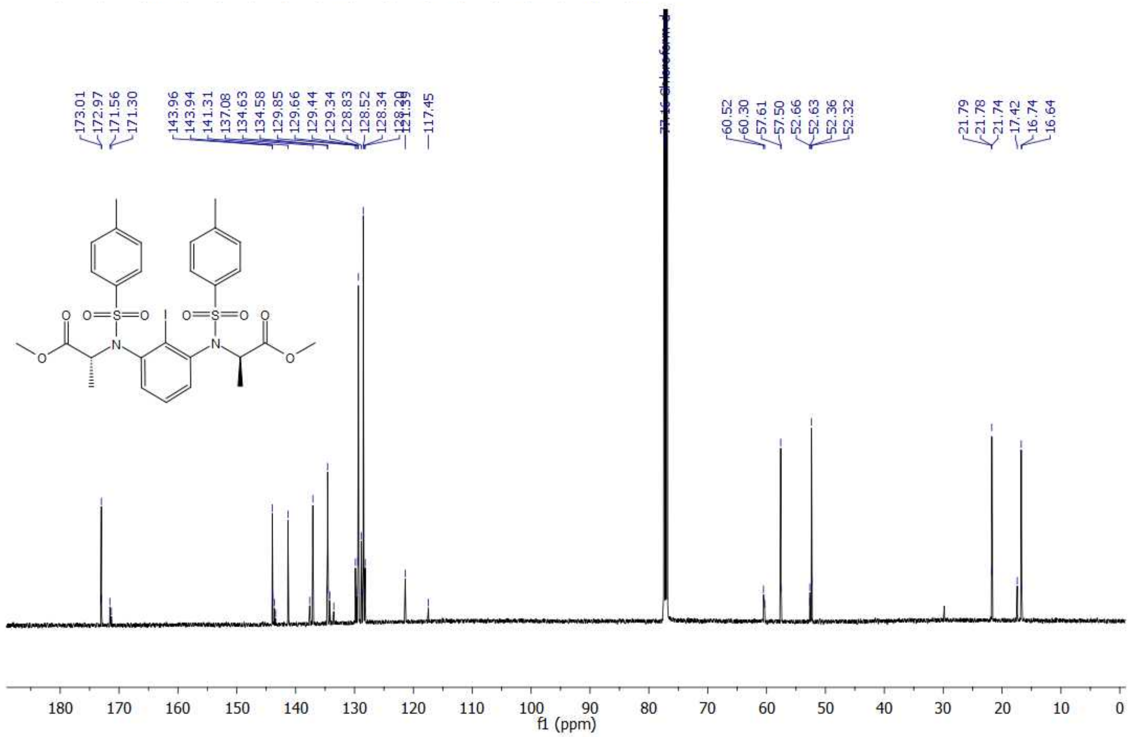

Diethyl 2,2'-((2-iodo-1,3-phenylene)bis(tosylazanediyl))(2*R*,2'*R*)-dipropionate (18d)

^{1}H NMR (300 MHz, $CDCl_3$)

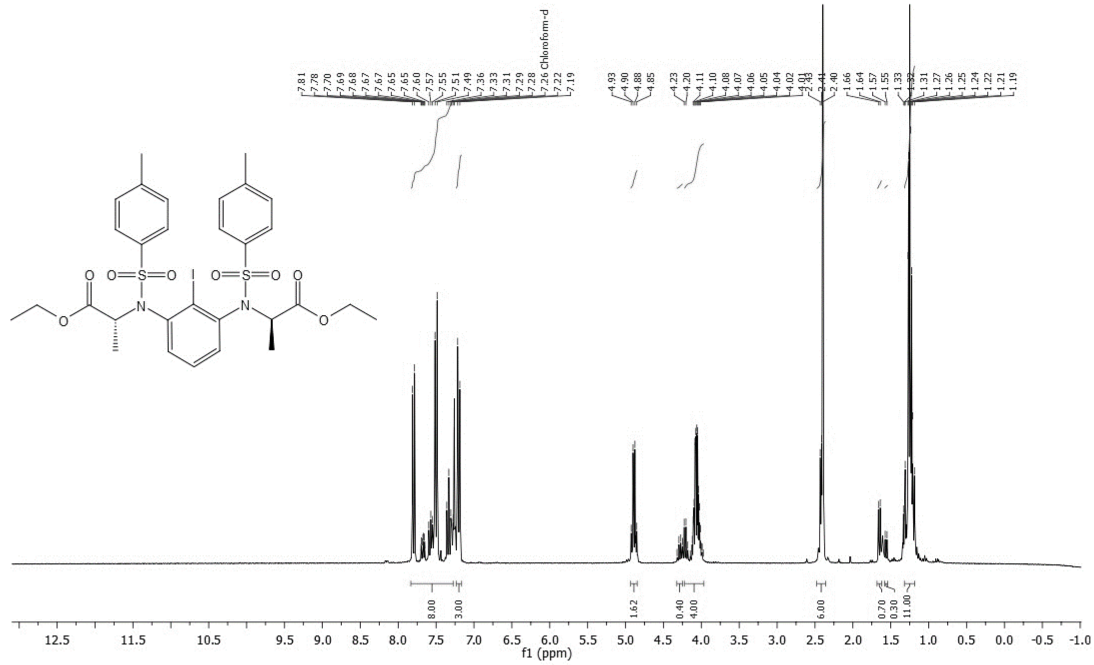

$^{13}C\{^{1}H\}$ NMR (126 MHz, $CDCl_3$)

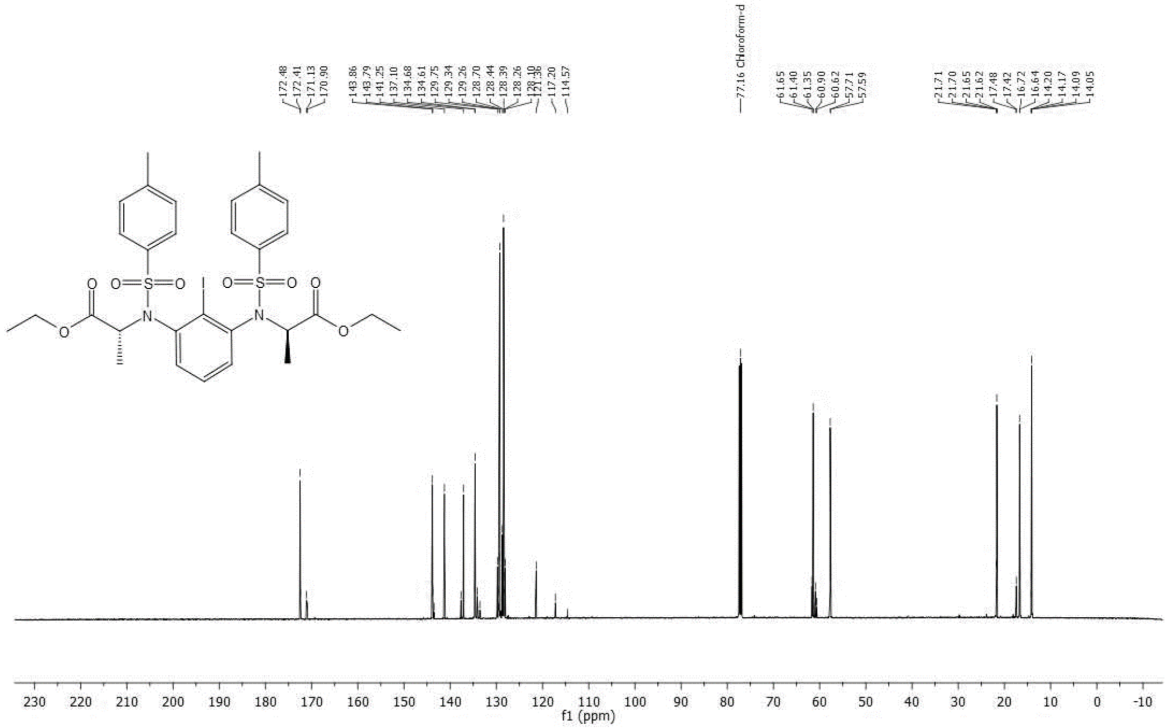

Methyl (*R*)-2-(2-iodophenoxy)propanoate (19a)

^{1}H NMR (500 MHz, $CDCl_3$)

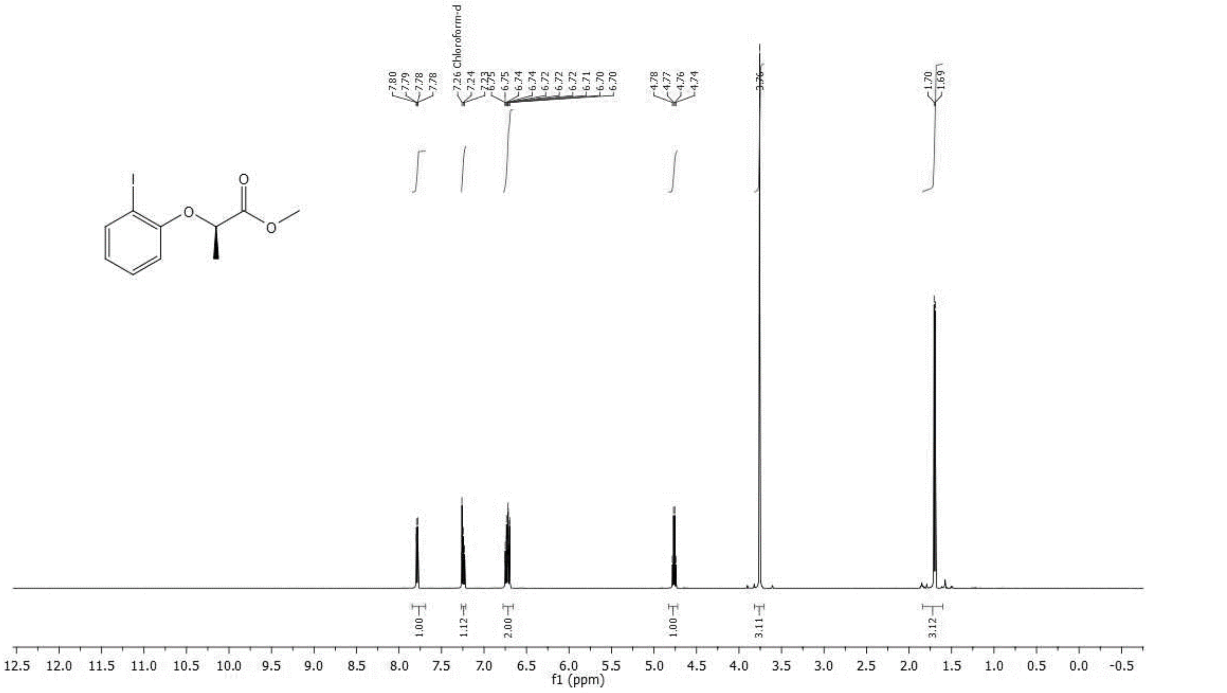

$^{13}C\{^{1}H\}$ NMR (126 MHz, $CDCl_3$)

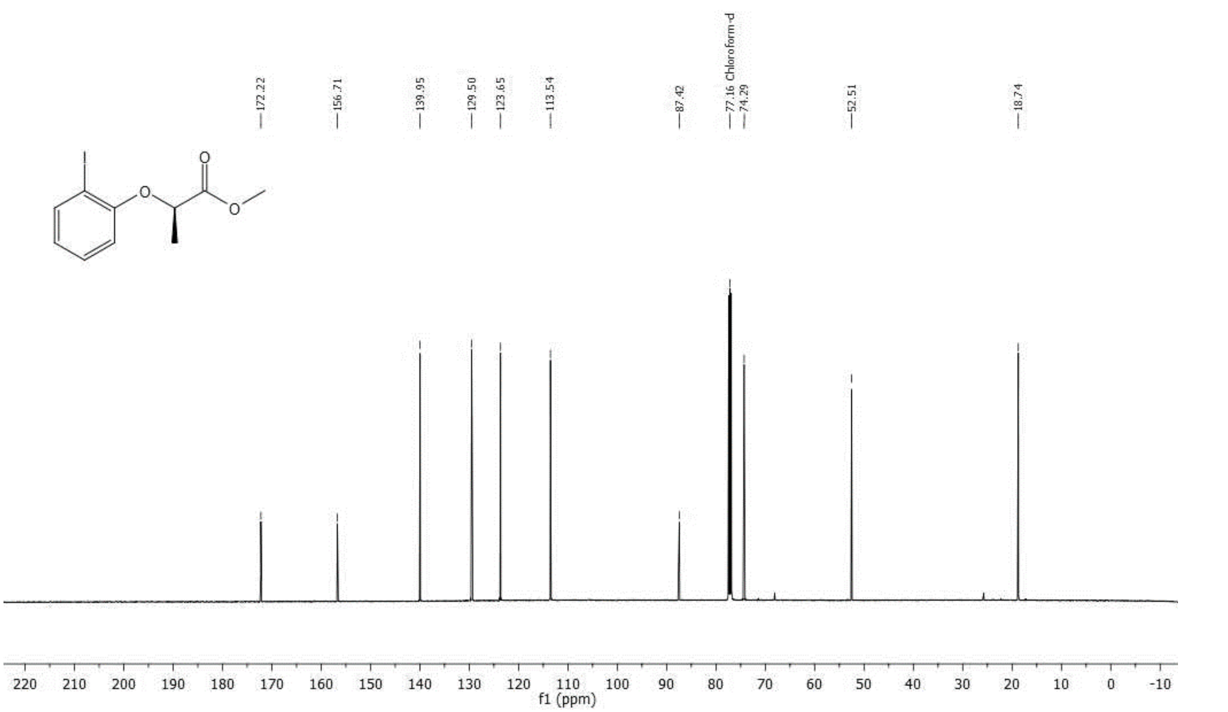

Methyl (*R*)-2-(2-iodo-3-methoxyphenoxy)propanoate (19b)

^{1}H NMR (500 MHz, $CDCl_3$)

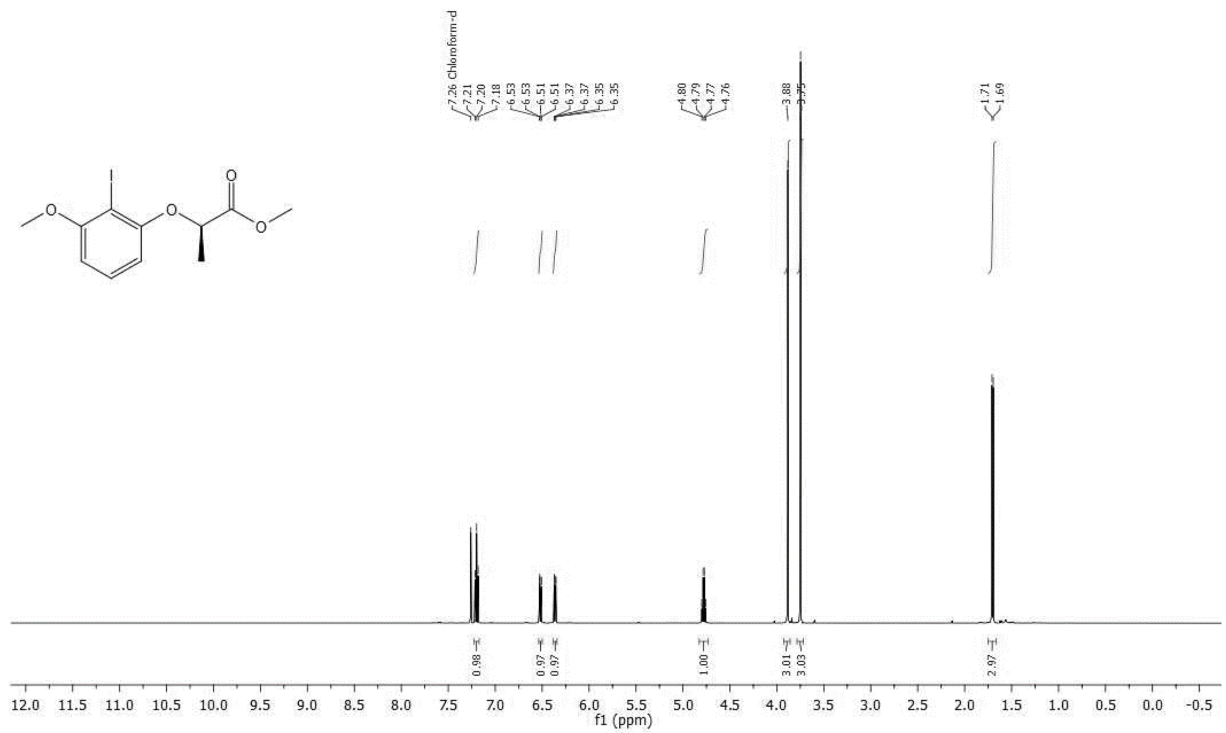

$^{13}C\{^{1}H\}$ NMR (126 MHz, $CDCl_3$)

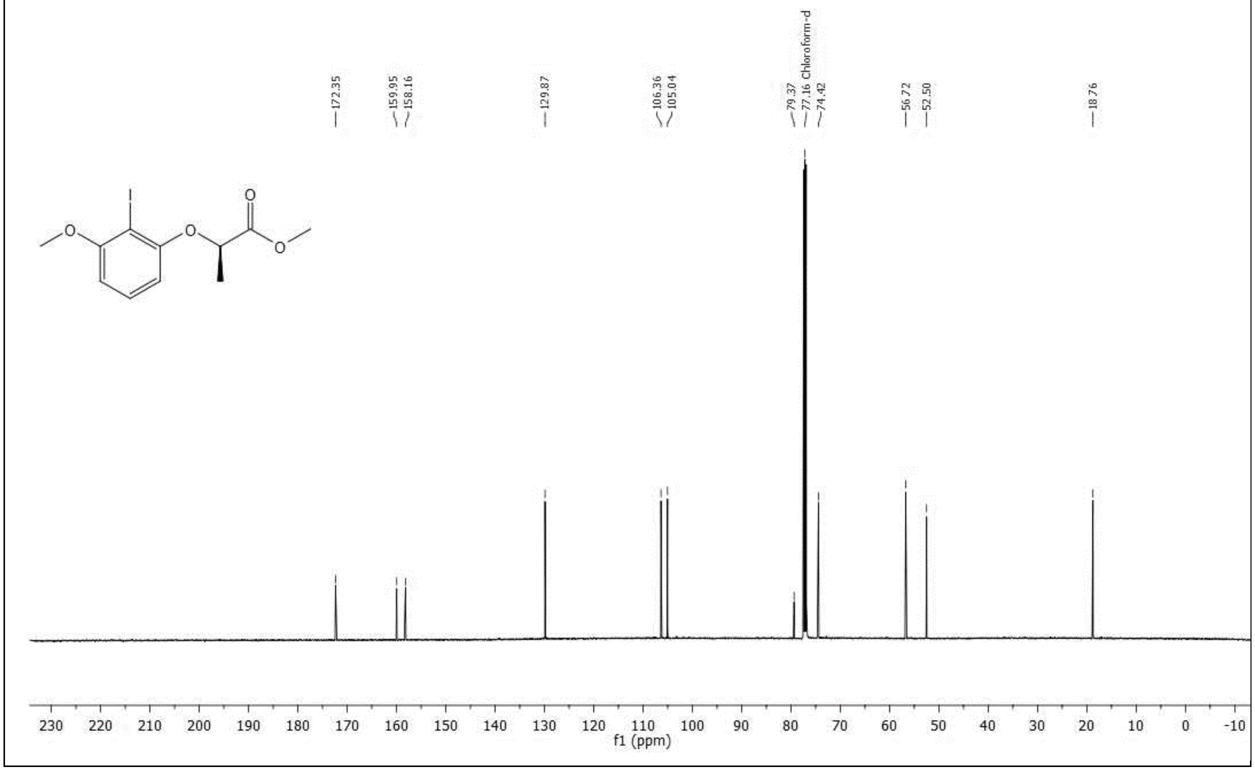

Methyl (*R*)-2-(2-iodo-3-nitrophenoxy)propanoate (19c)

^{1}H NMR (500 MHz, $CDCl_3$)

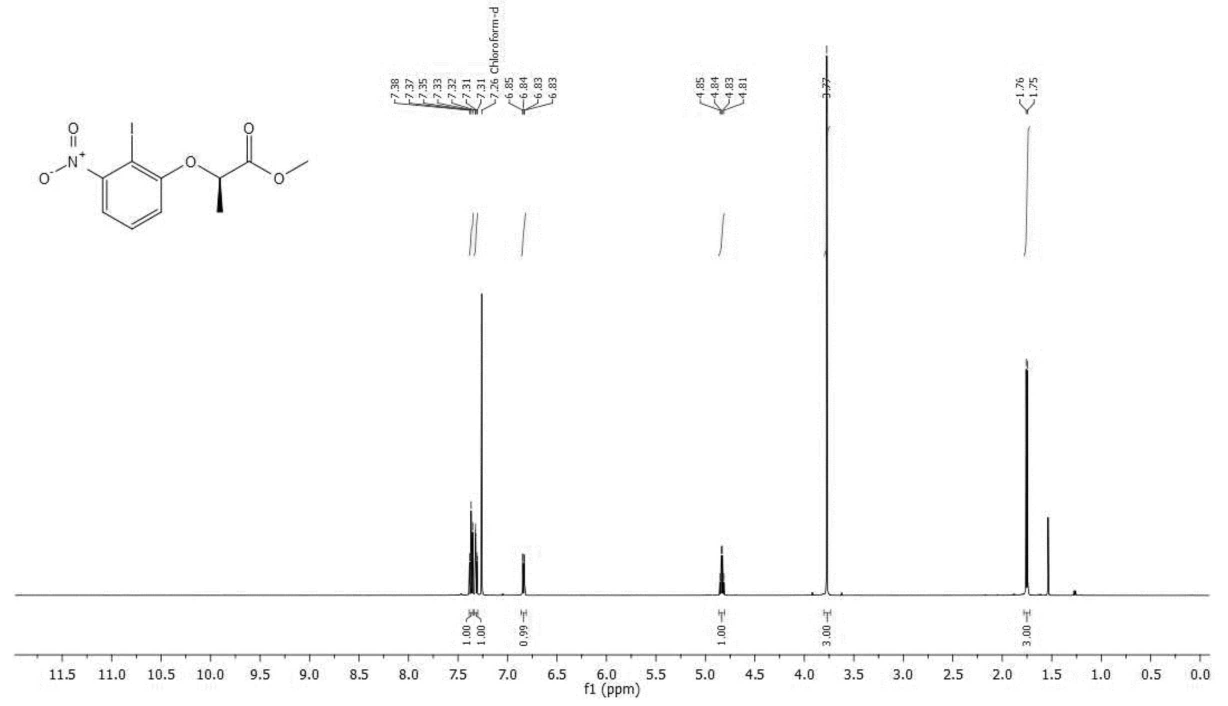

$^{13}C\{^{1}H\}$ NMR (126 MHz, $CDCl_3$)

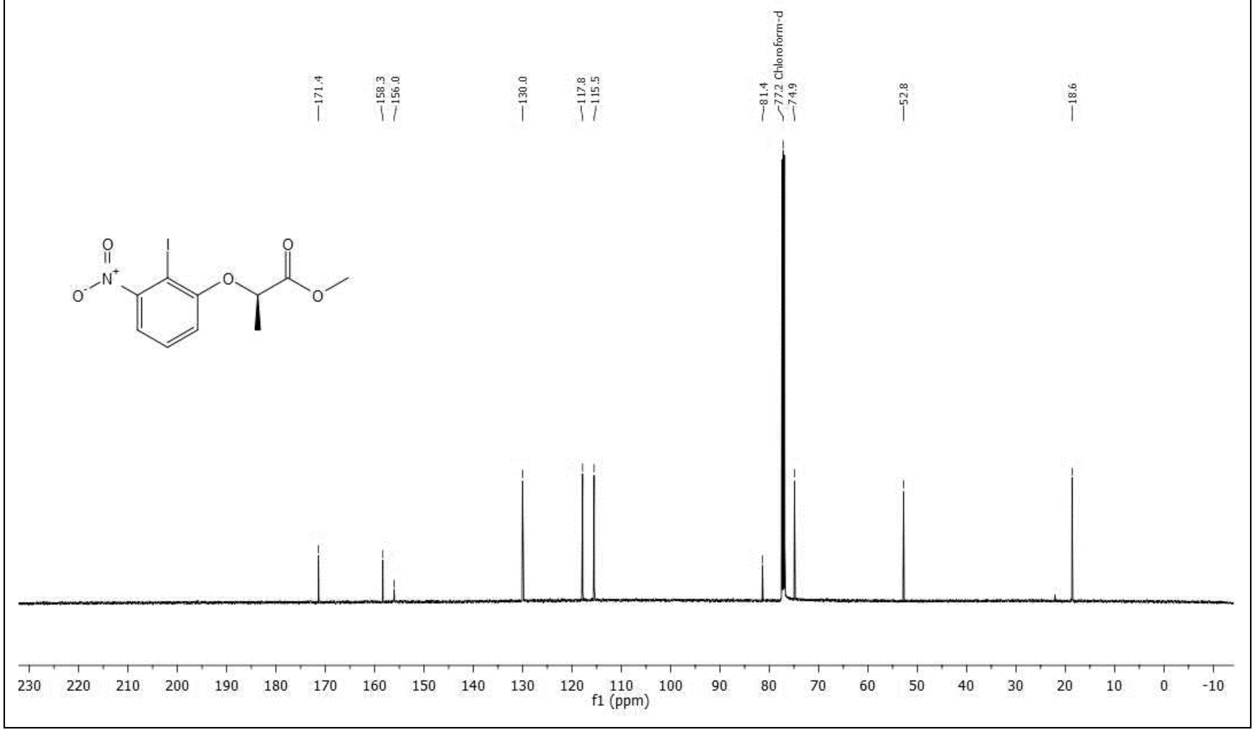

(*S*)-1-Oxo-1-phenylpropan-2-yl 4-methylbenzenesulfonate (20a)

^{1}H NMR (300 MHz, $CDCl_3$)

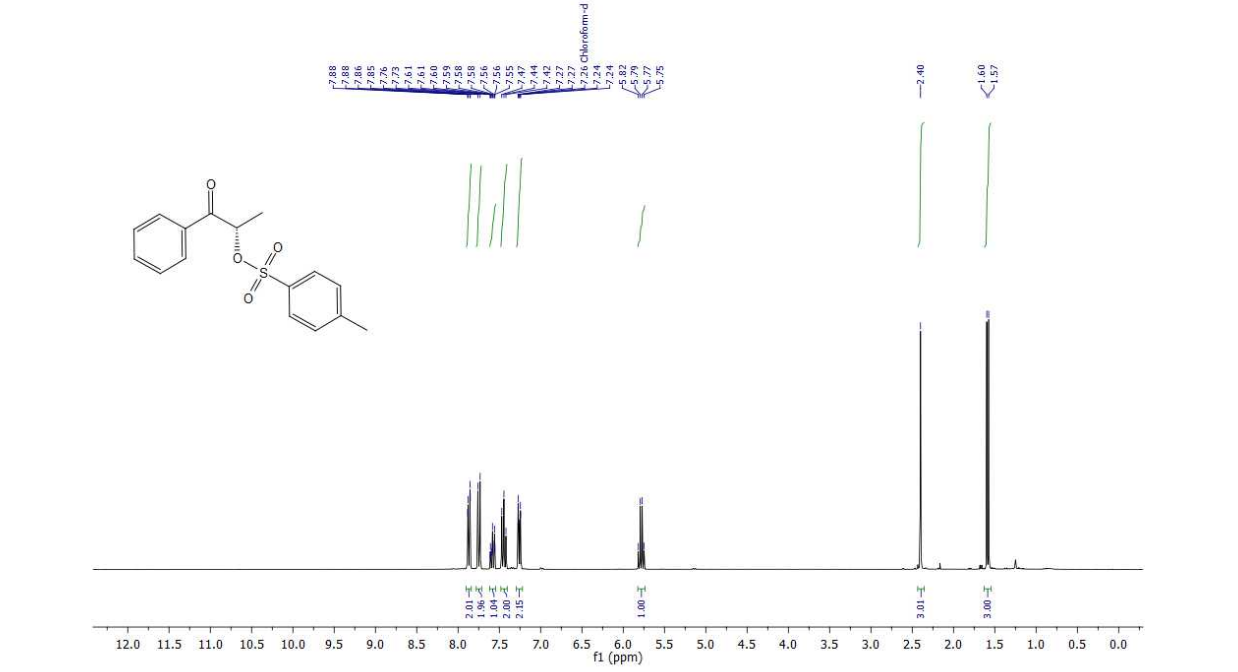

^{13}C{^{1}H} NMR (75 MHz, $CDCl_3$)

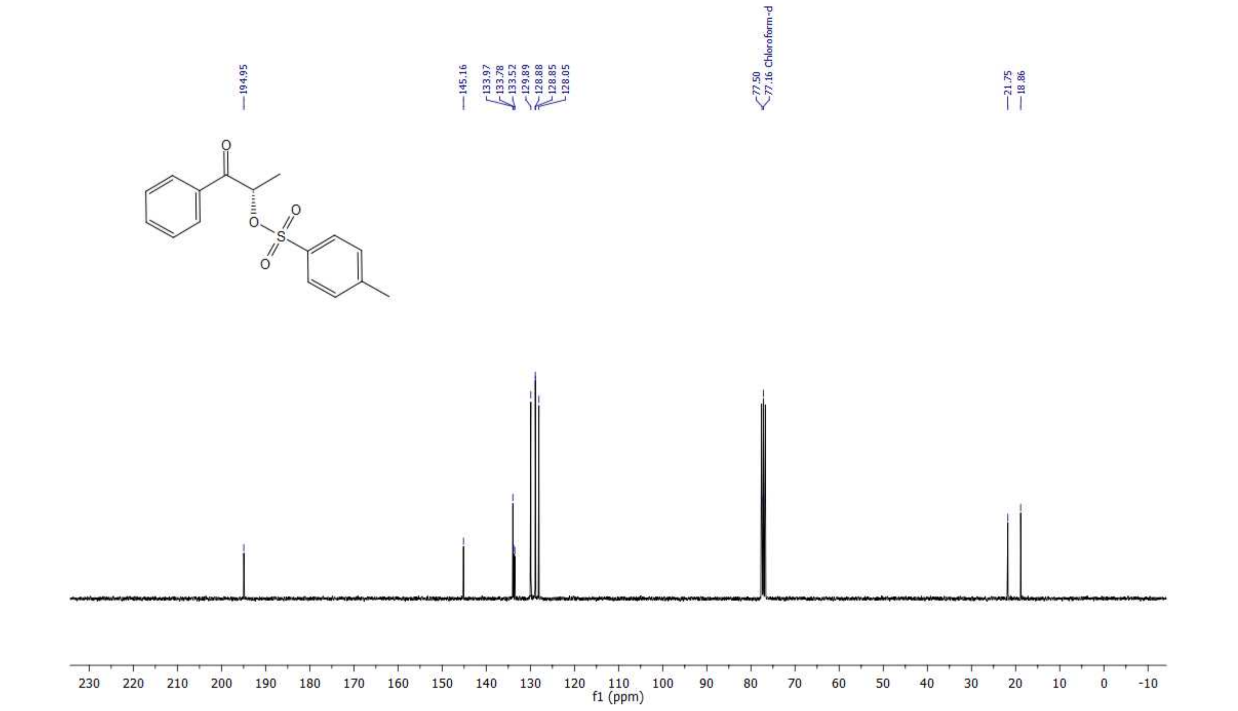

(*S*)-1-(3-Chlorophenyl)-1-oxopropan-2-yl 4-methylbenzenesulfonate (20b)

^{1}H NMR (500 MHz, $CDCl_3$)

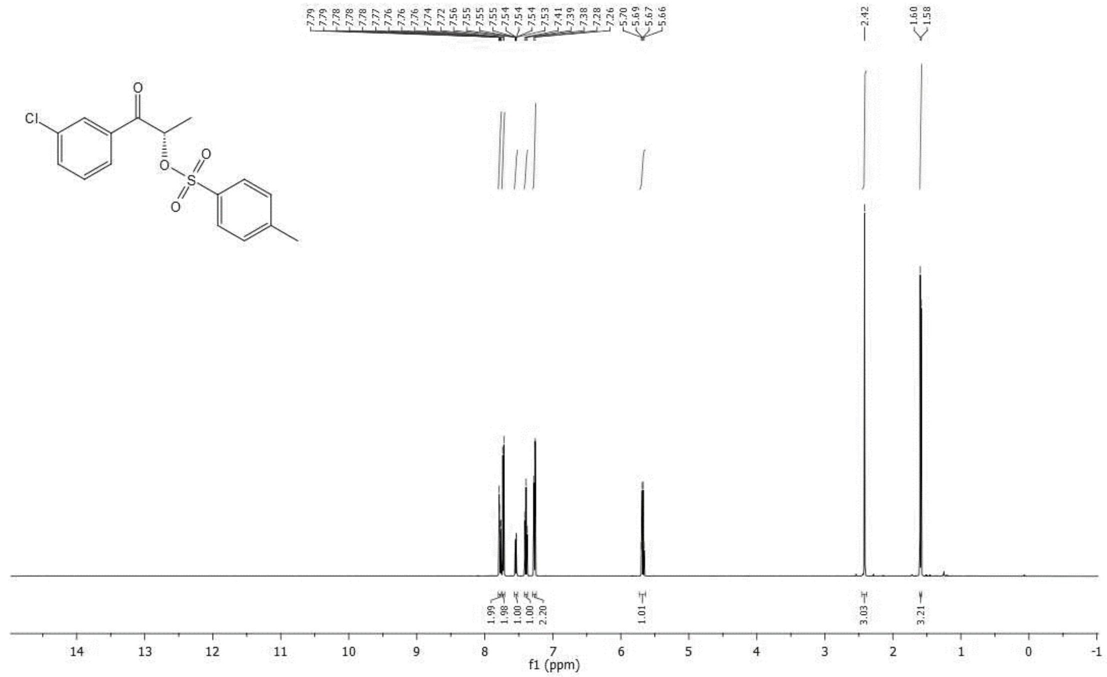

$^{13}C\{^{1}H\}$ NMR (126 MHz, $CDCl_3$)

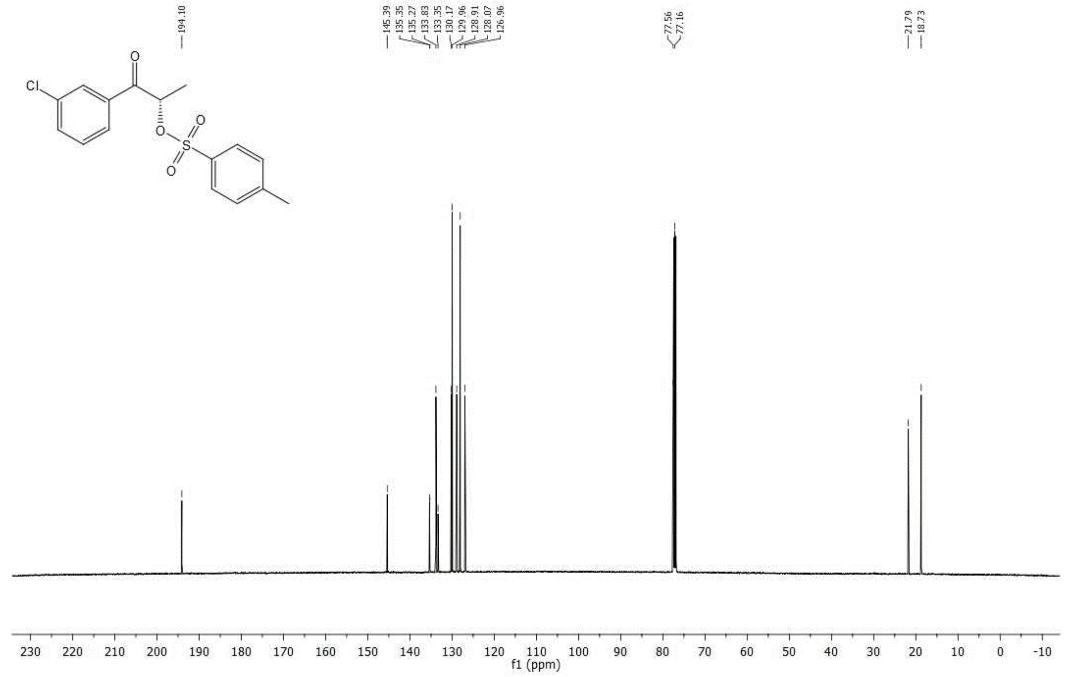

(*S*)-1-(3-Bromophenyl)-1-oxopropan-2-yl 4-methylbenzenesulfonate (20c)

^{1}H NMR (500 MHz, CDCl$_3$)

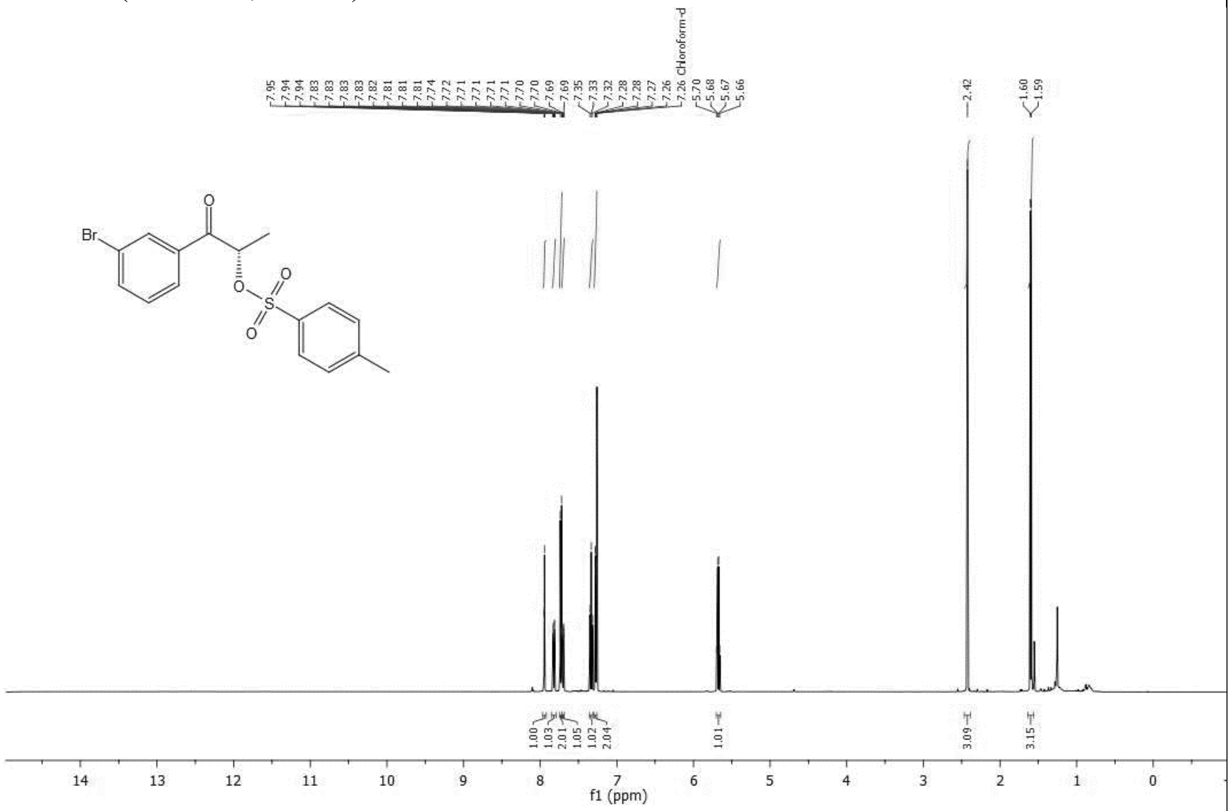

^{13}C{^{1}H} NMR (126 MHz, CDCl$_3$)

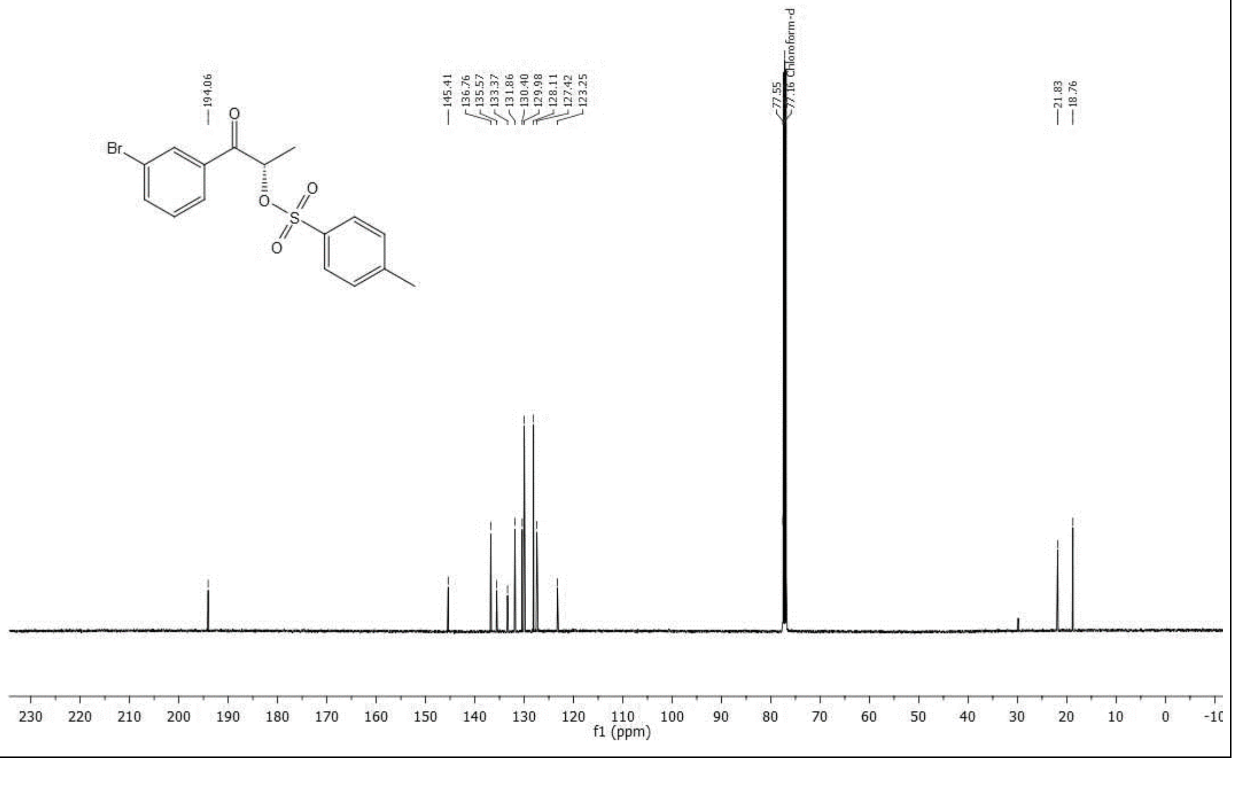

(*S*)-1-(3-Nitrophenyl)-1-oxopropan-2-yl 4-methylbenzenesulfonate (20d)

^{1}H NMR (400 MHz, $CDCl_3$)

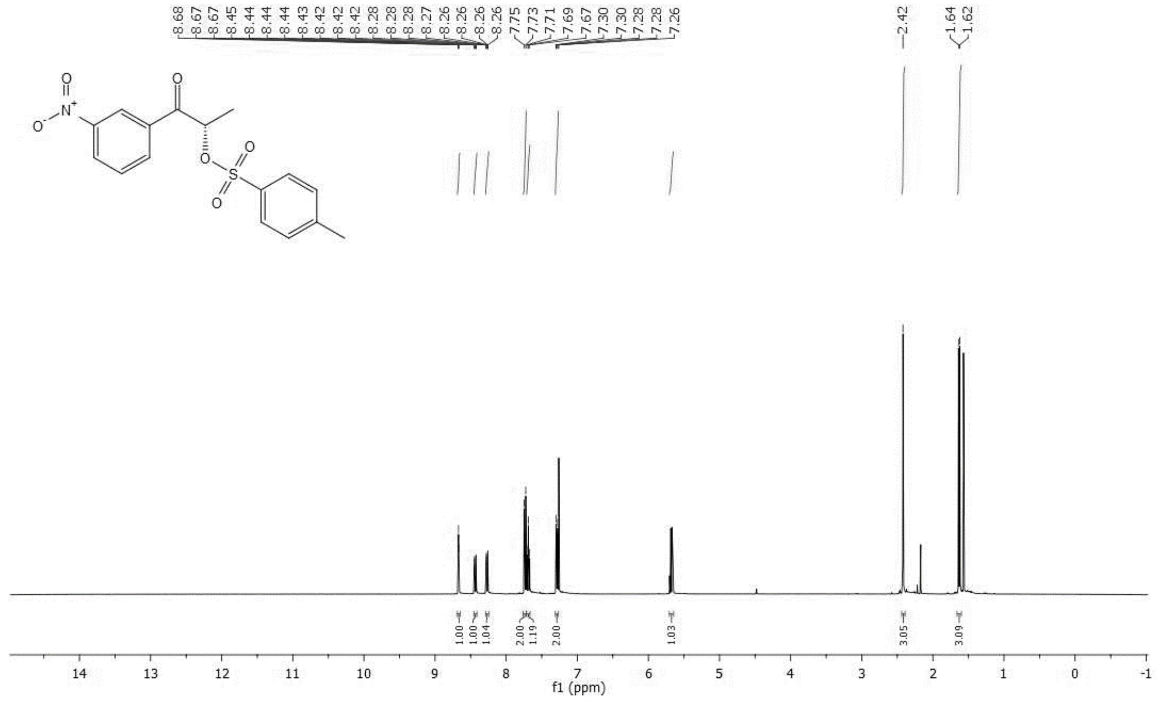

$^{13}C\{^{1}H\}$ NMR (101 MHz, $CDCl_3$)

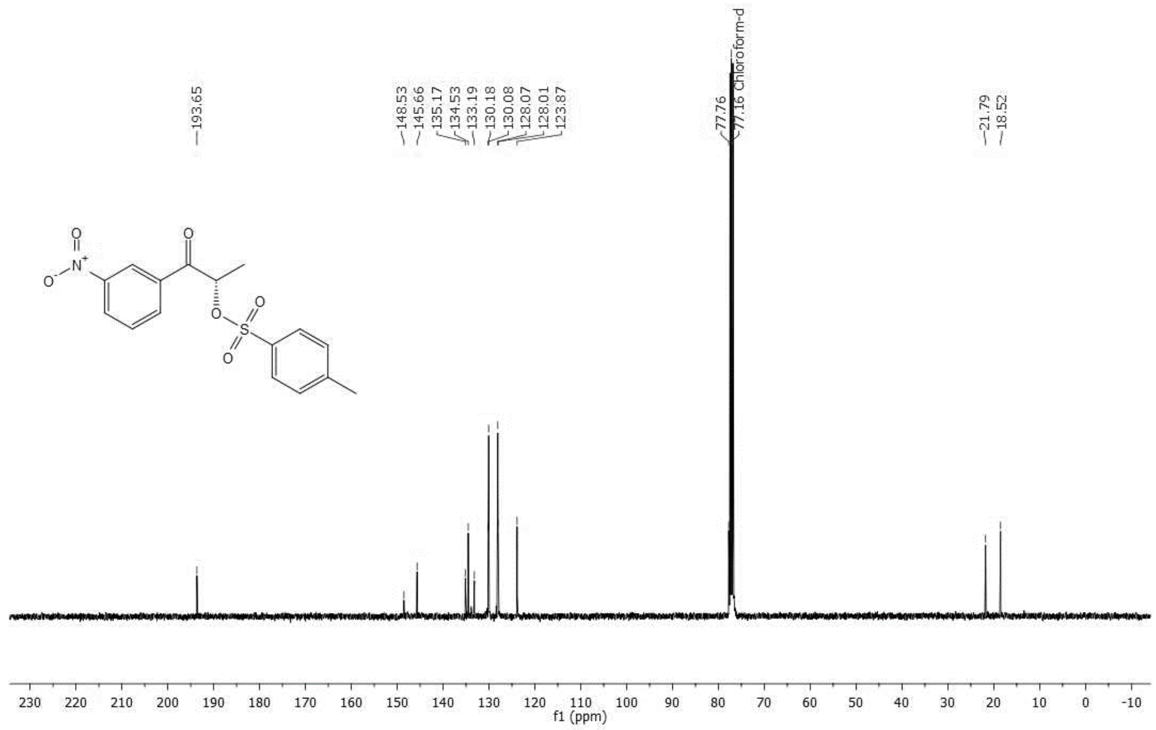

(*S*)-1-Oxo-1-(3-(rifluoromethyl)phenyl)propan-2-yl 4-methylbenzenesulfonate (20e)
^{1}H NMR (500 MHz, $CDCl_3$)

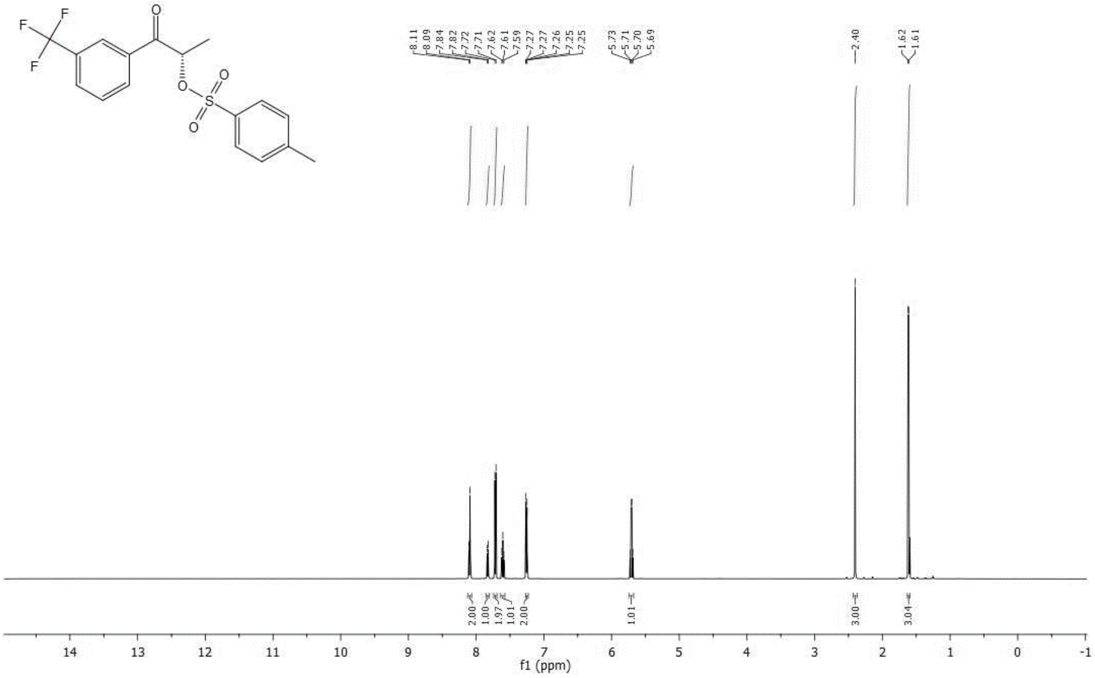

$^{13}C\{^{1}H\}$ NMR (126 MHz, $CDCl_3$)

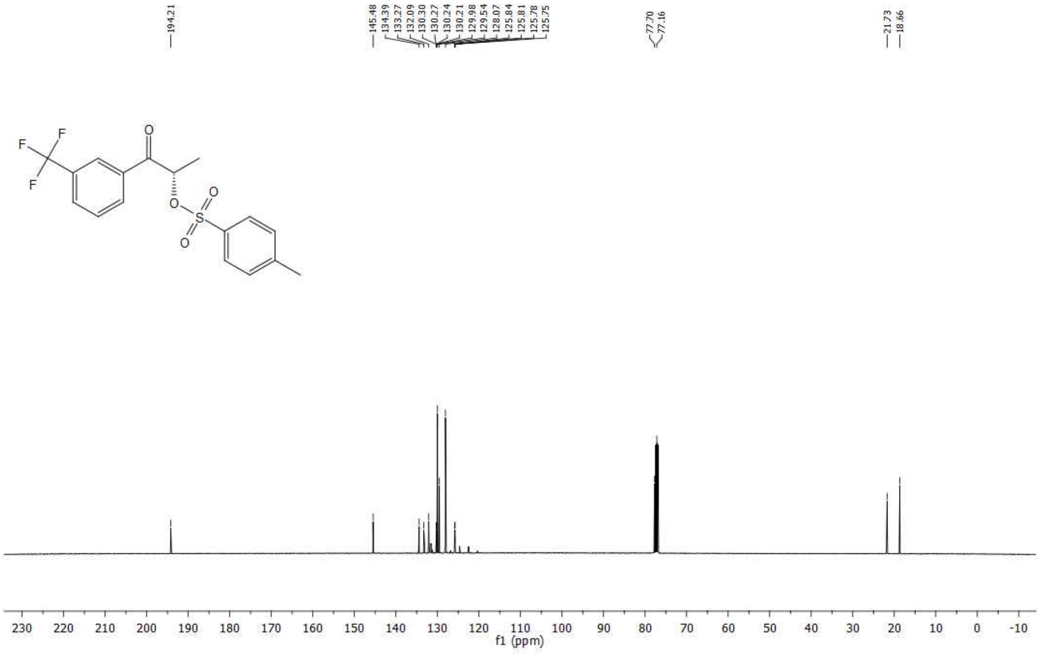

(*S*)-1-(4-Fluorophenyl)-1-oxopropan-2-yl 4-methylbenzenesulfonate (20f)

^{1}H NMR (500 MHz, $CDCl_3$)

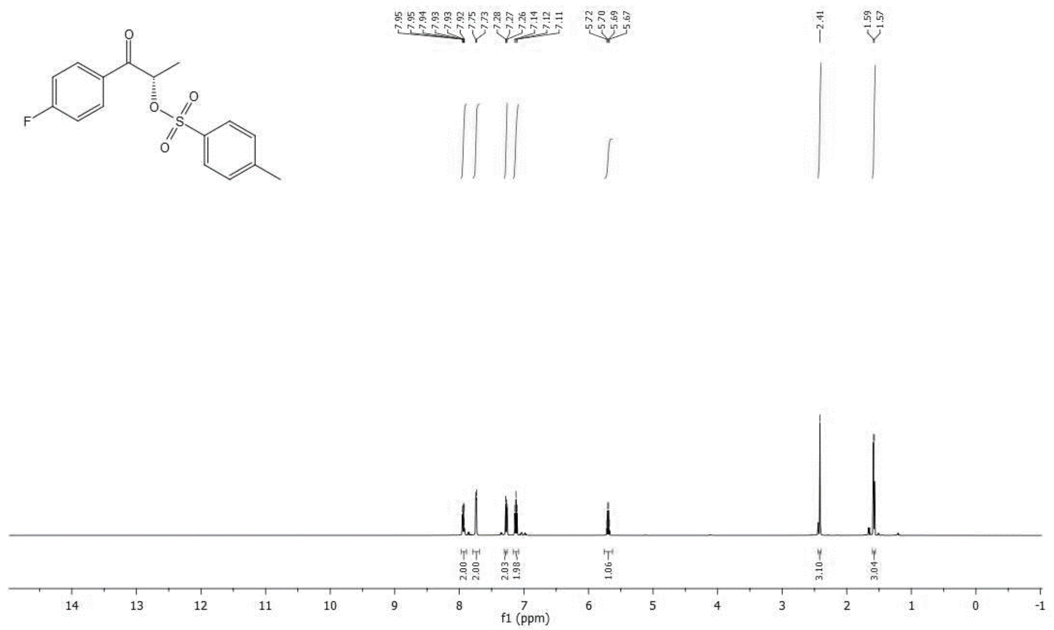

$^{13}C\{^{1}H\}$ NMR (126 MHz, $CDCl_3$)

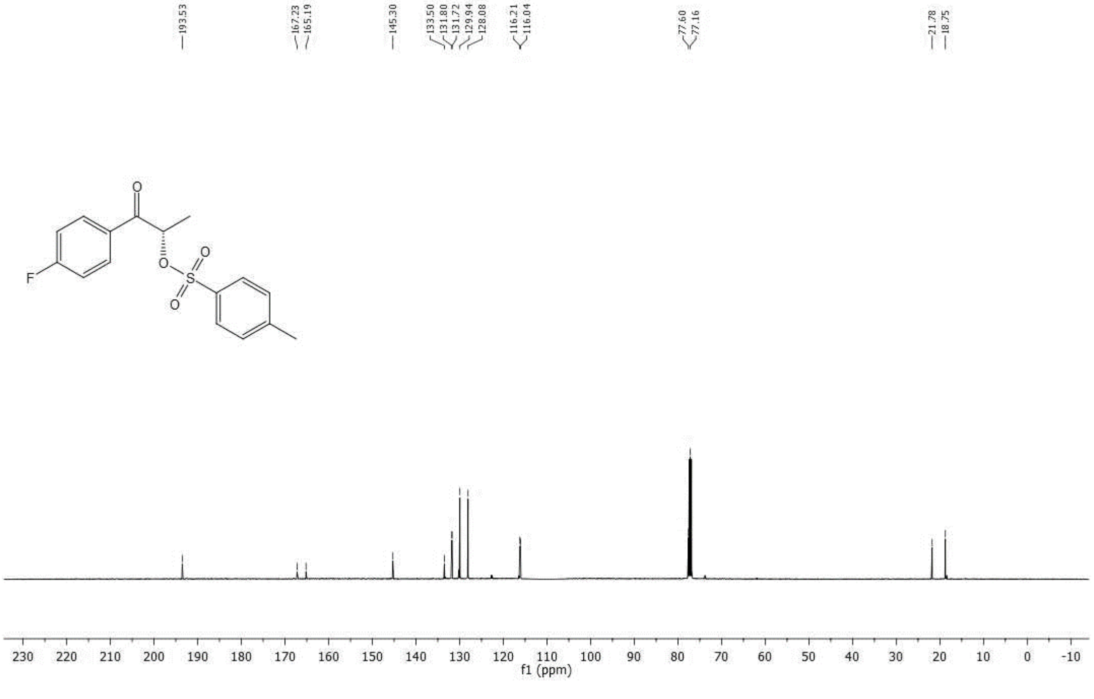

(*S*)-1-(4-Bromophenyl)-1-oxopropan-2-yl 4-methylbenzenesulfonate (20g)

^{1}H NMR (500 MHz, $CDCl_3$)

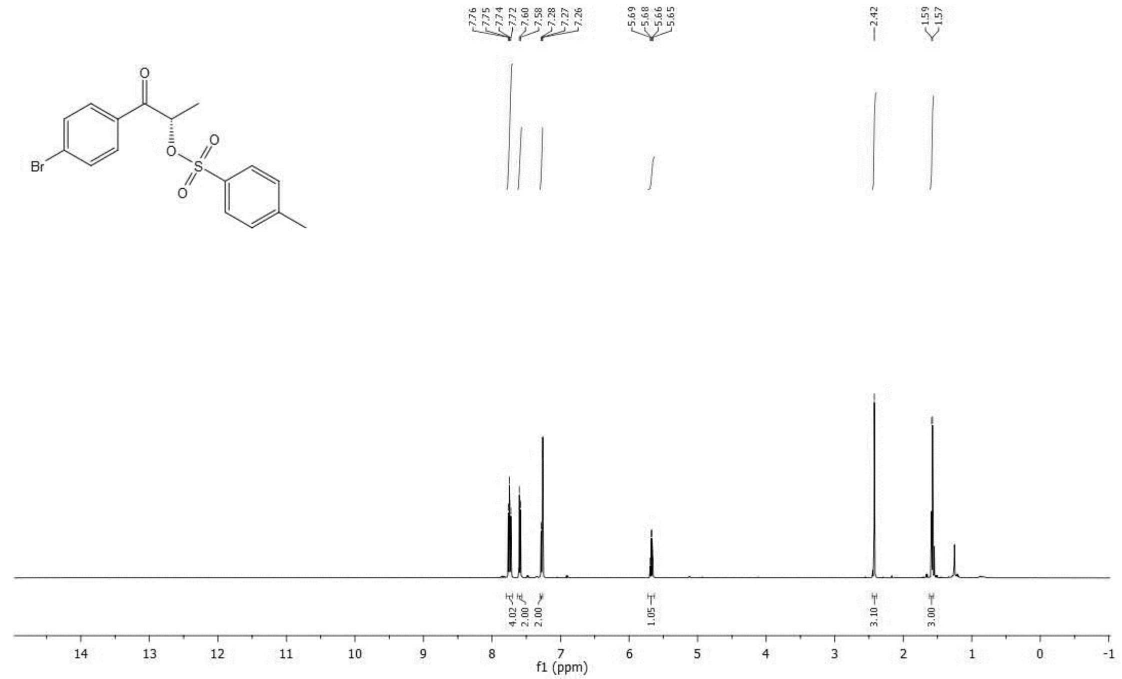

^{13}C{^{1}H} NMR (126 MHz, $CDCl_3$)

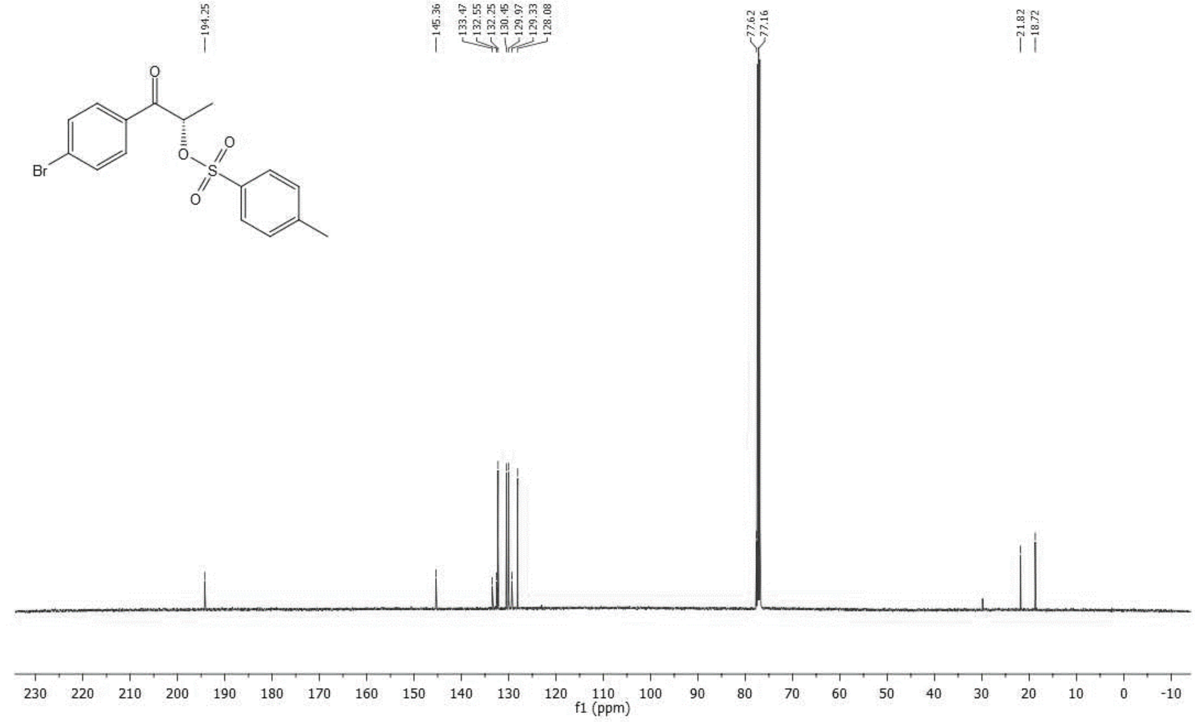

(*S*)-1-Oxo-1-(4-(trifluoromethyl)phenyl)propan-2-yl 4-methylbenzenesulfonate (20h)

^{1}H NMR (500 MHz, $CDCl_3$)

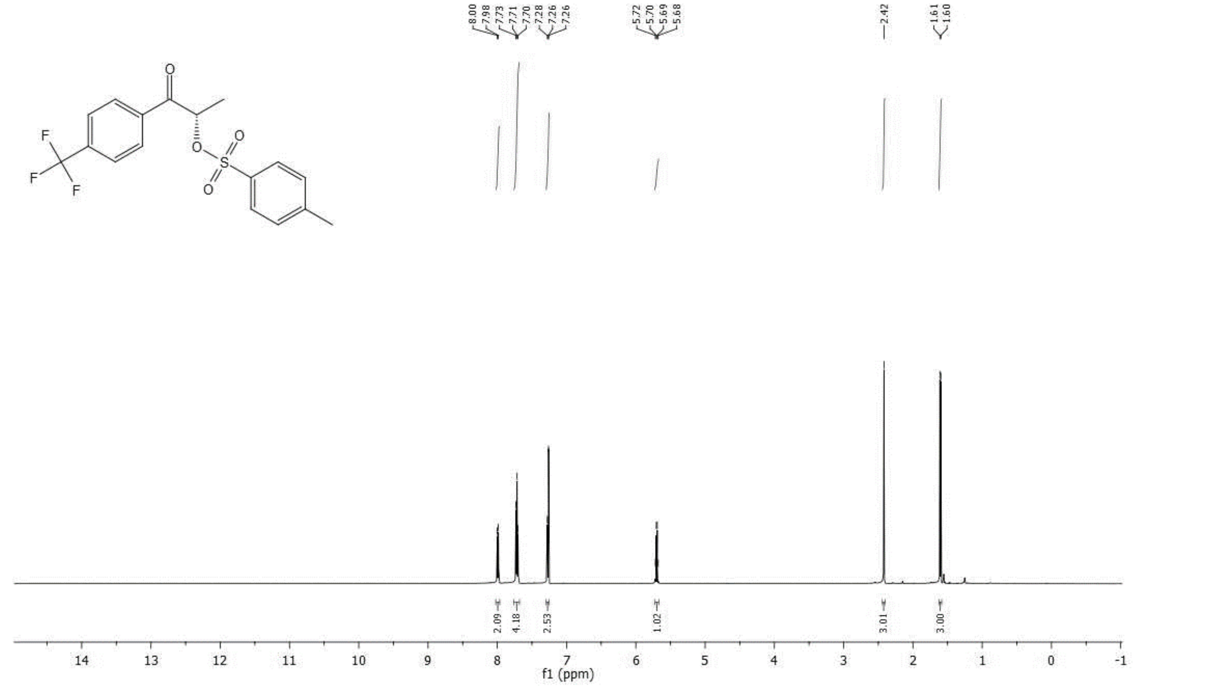

^{13}C{^{1}H} NMR (126 MHz, $CDCl_3$)

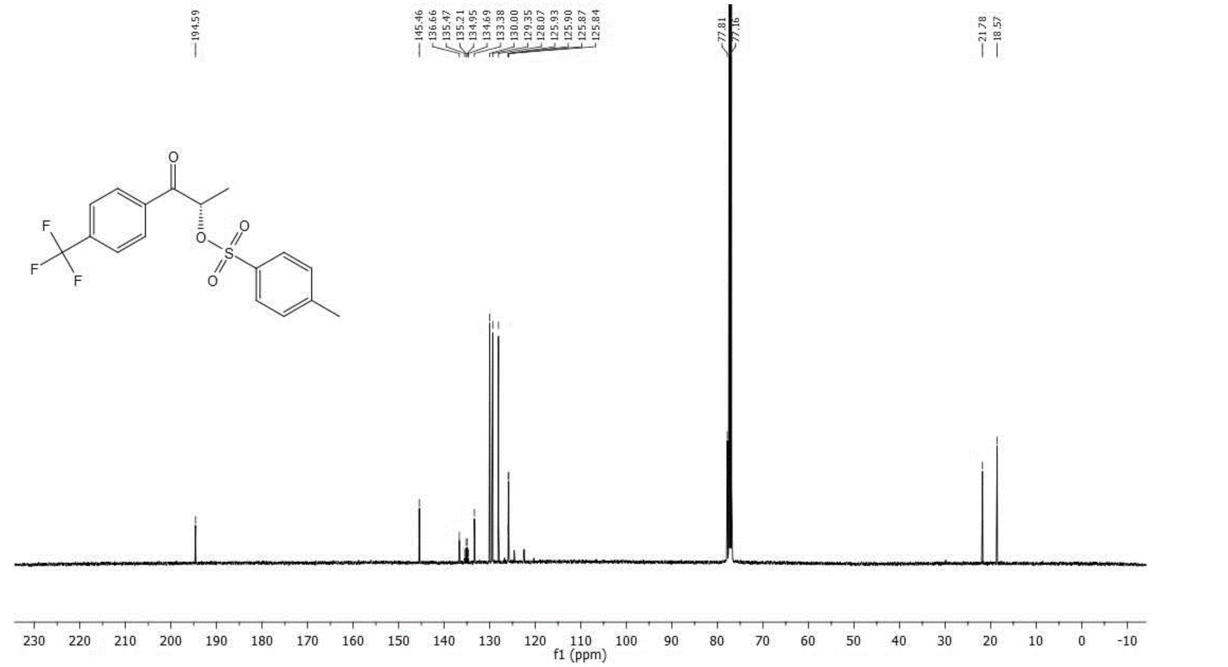

(*S*)-1-Oxo-1-(p-tolyl)propan-2-yl 4-methylbenzenesulfonate (20i)

^{1}H NMR (500 MHz, $CDCl_3$)

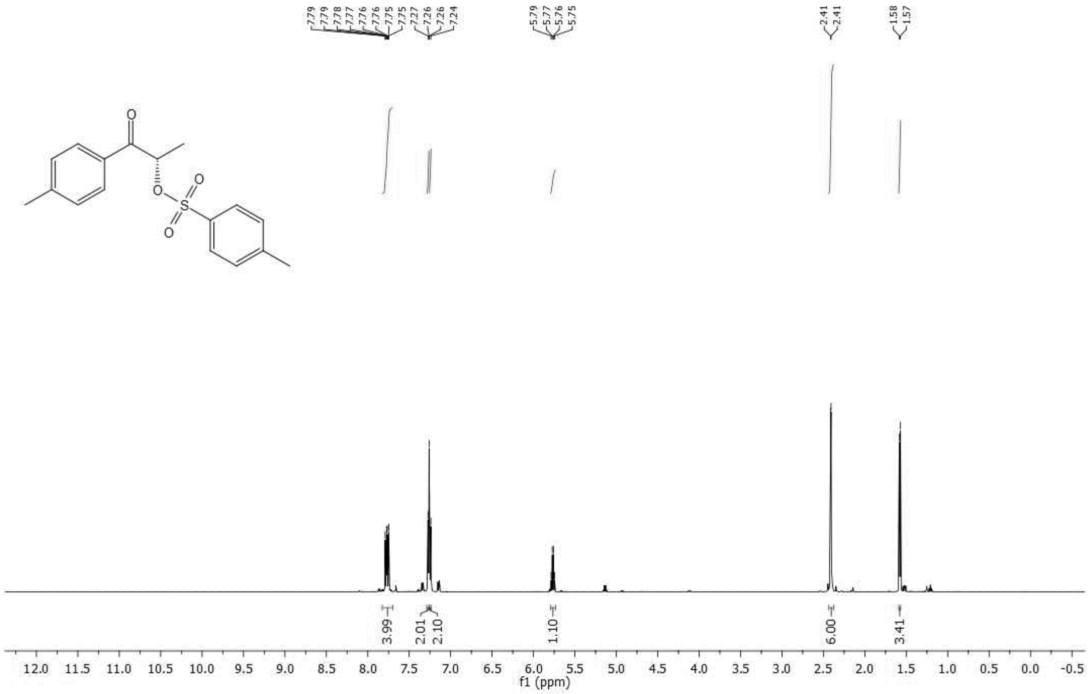

$^{13}C\{^{1}H\}$ NMR (126 MHz, $CDCl_3$)

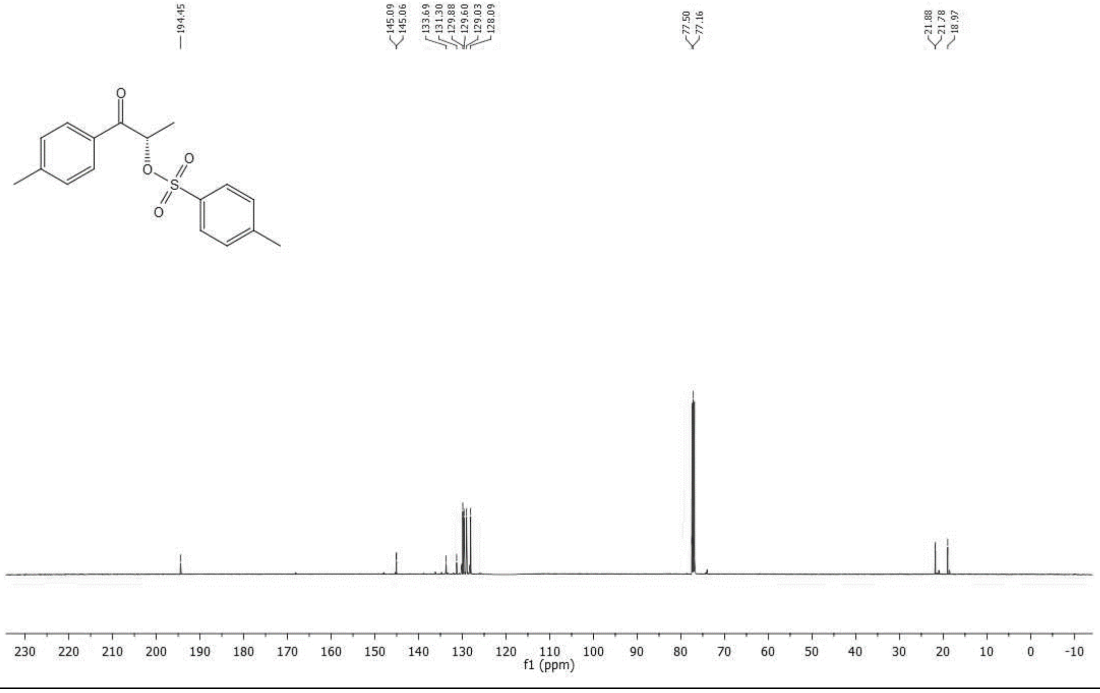

(*S*)-1-(4-Methoxyphenyl)-1-oxopropan-2-yl 4-methylbenzenesulfonate (20j)

^{1}H NMR (500 MHz, $CDCl_3$)

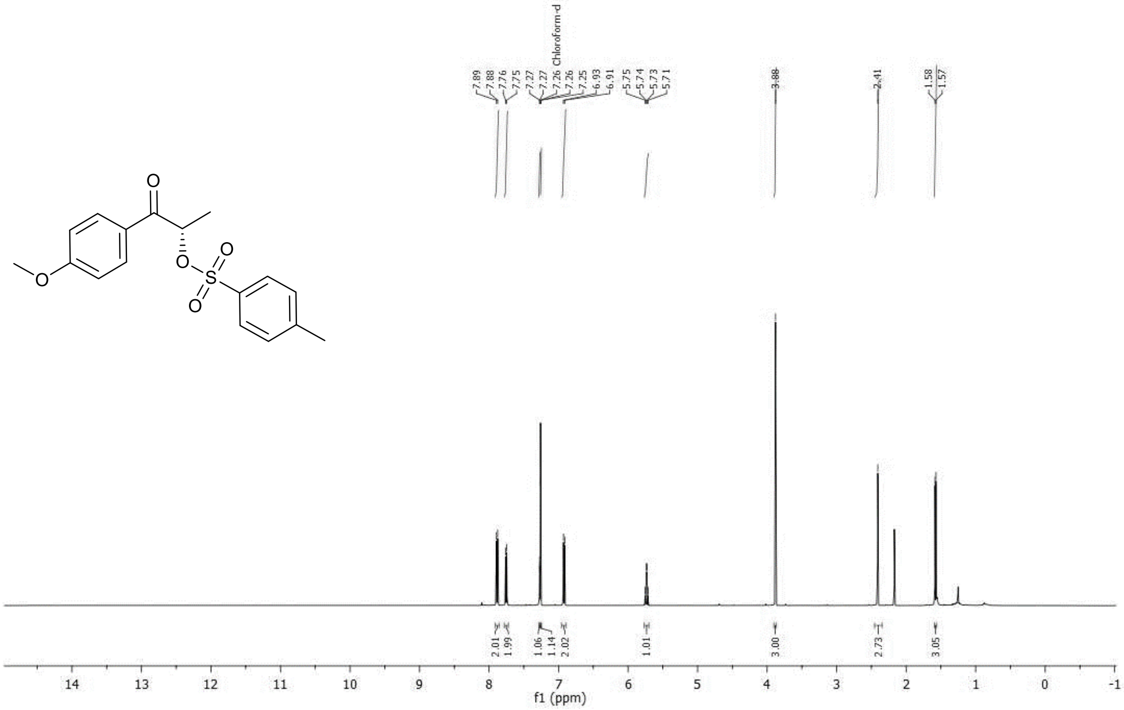

^{13}C{^{1}H} NMR (126 MHz, $CDCl_3$)

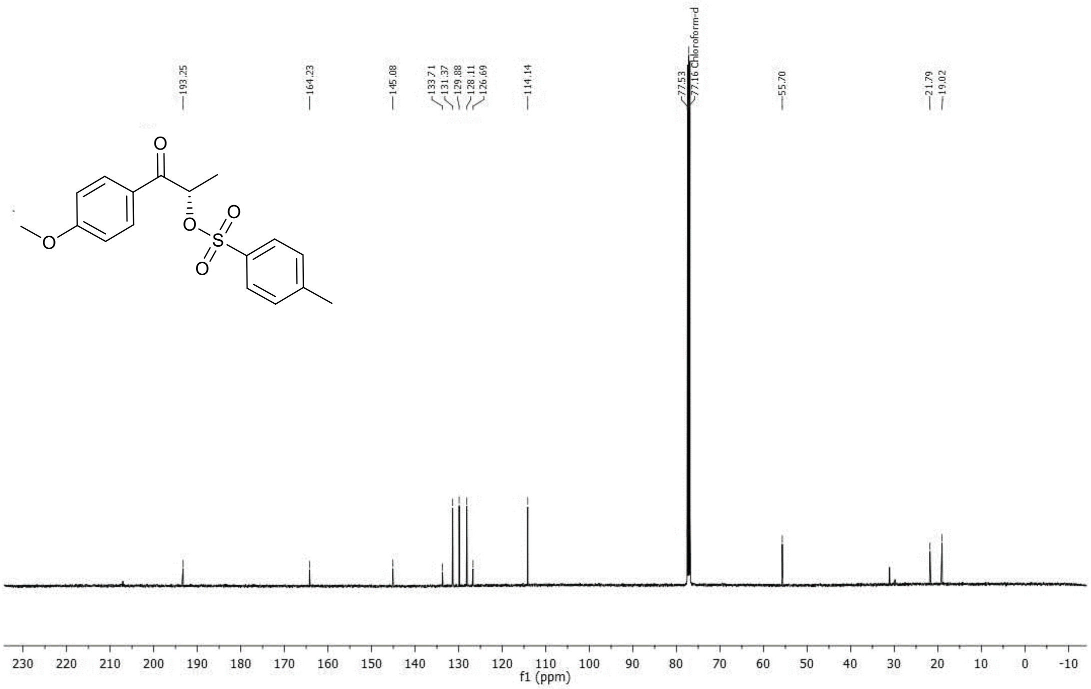

(*S*)-1-(4-(*tert*-Butyl)phenyl)-1-oxopropan-2-yl 4-methylbenzenesulfonate (20k)

^{1}H NMR (500 MHz, $CDCl_3$)

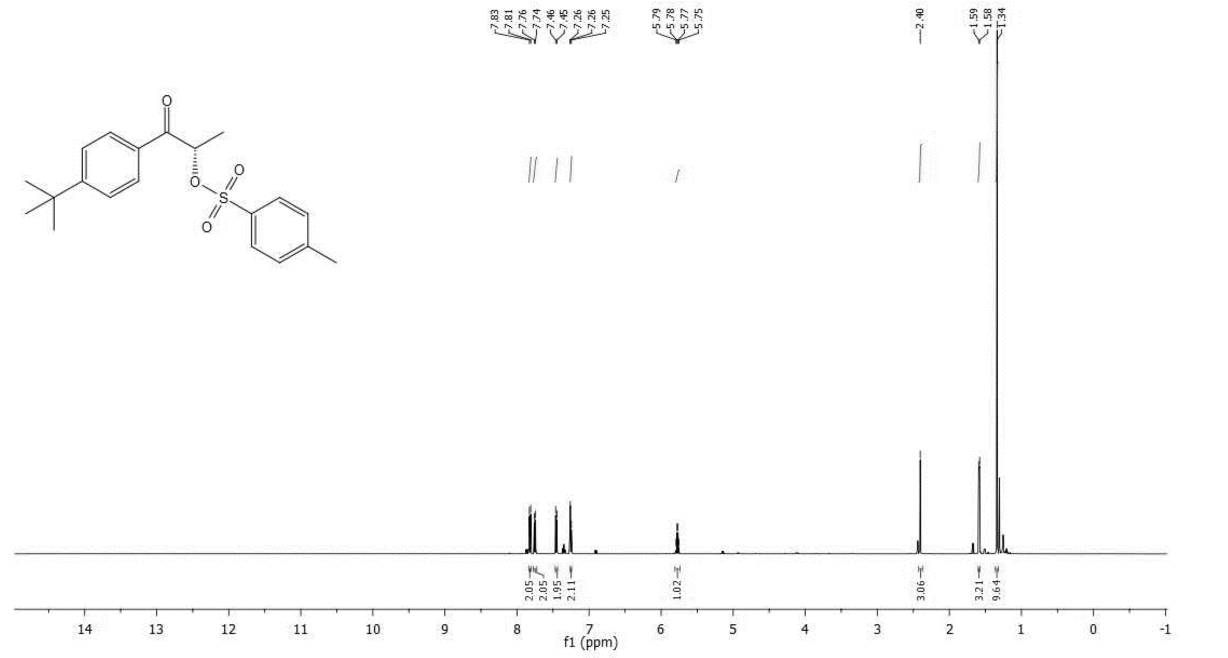

^{13}C{^{1}H} NMR (101 MHz, $CDCl_3$)

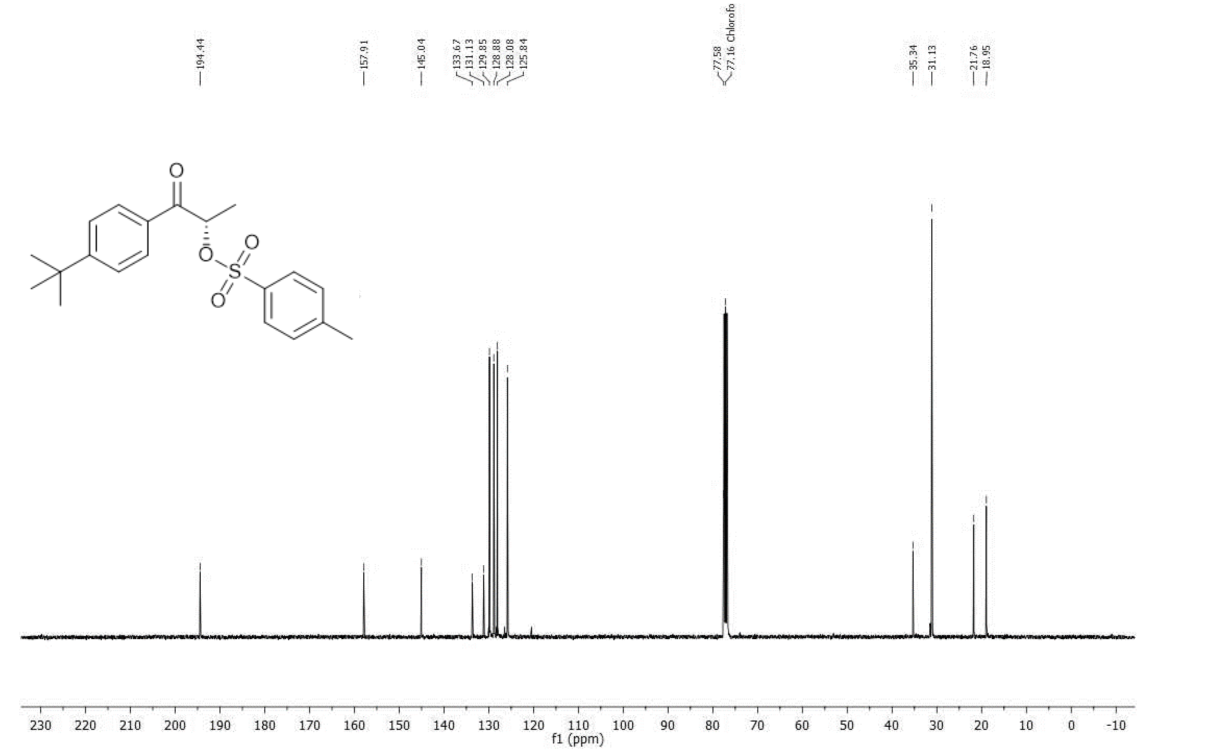

(*S*)-2-Oxo-1,2-diphenylethyl 4-methylbenzenesulfonate (20l)

^{1}H NMR (300 MHz, $CDCl_3$)

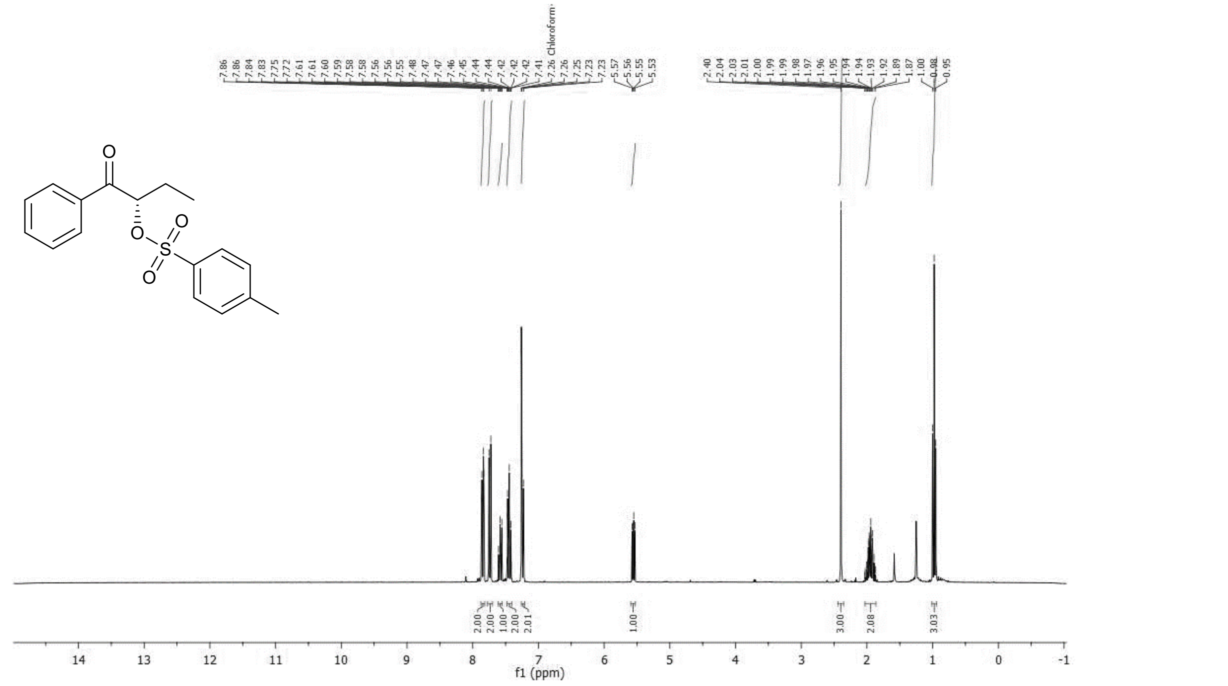

$^{13}C\{^{1}H\}$ NMR (126 MHz, $CDCl_3$)

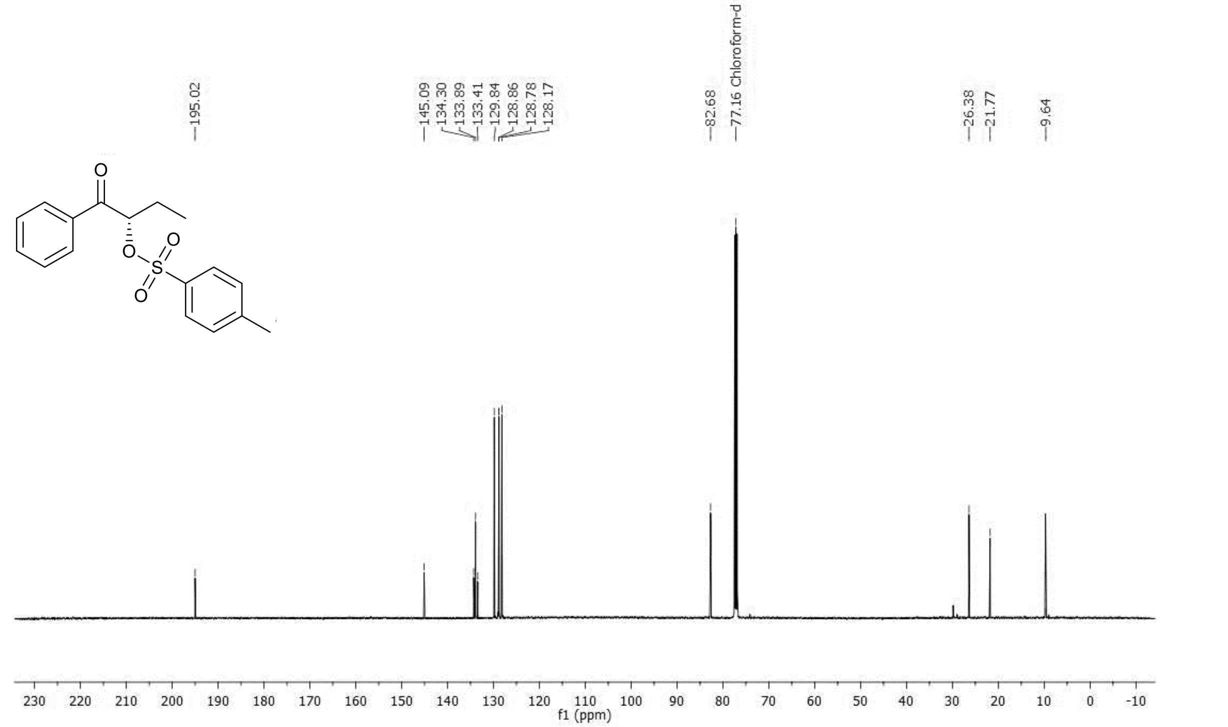

(*S*)-1-Oxo-1-phenyloctan-2-yl 4-methylbenzenesulfonate (20m)

^{1}H NMR (300 MHz, $CDCl_3$)

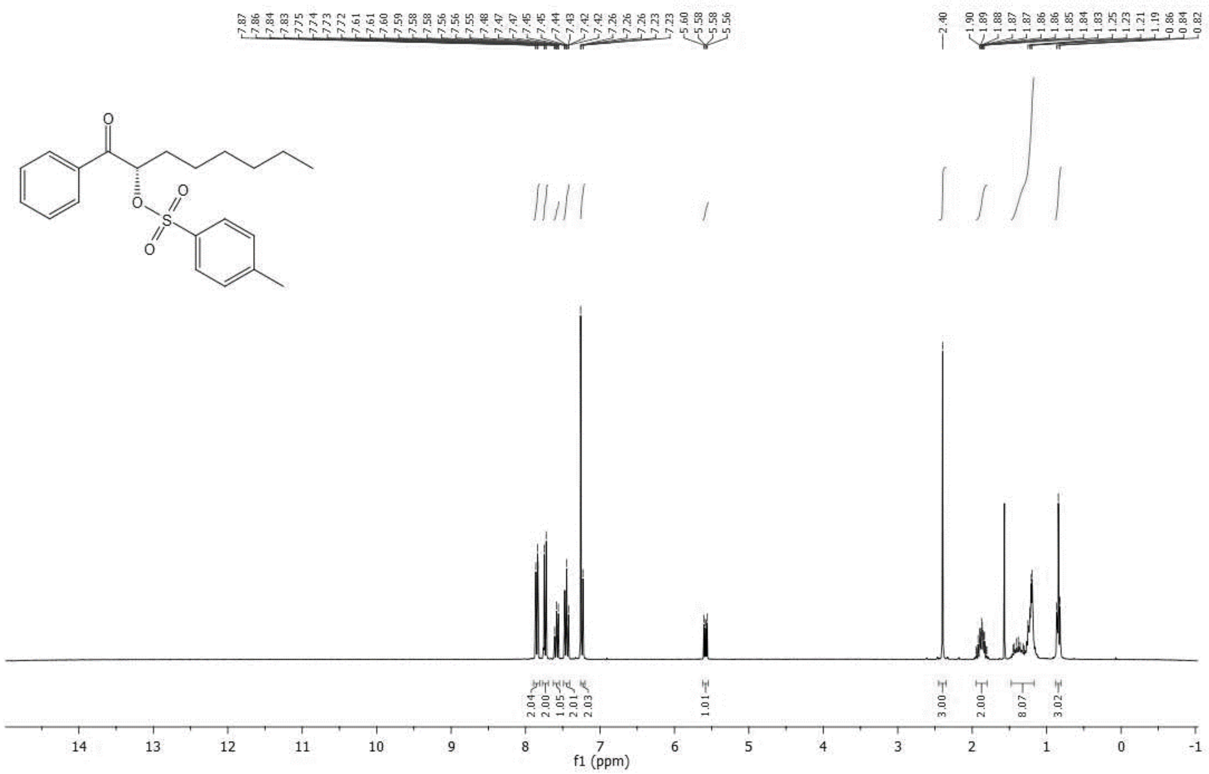

$^{13}C\{^{1}H\}$ NMR (75 MHz, $CDCl_3$)

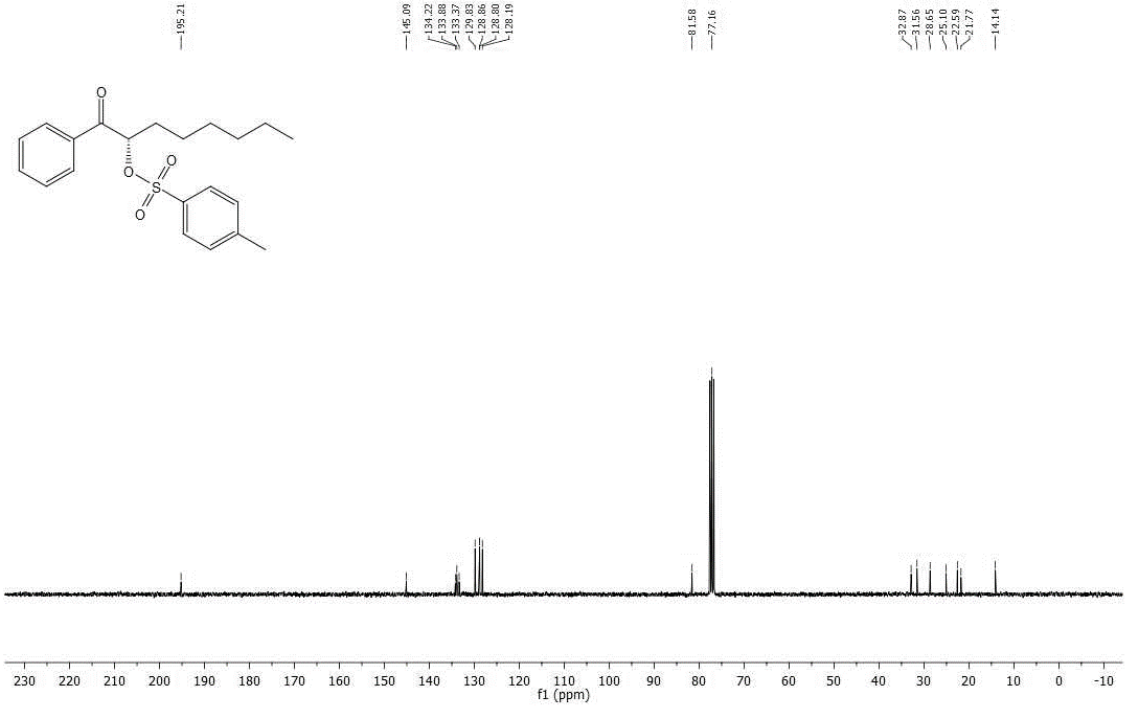

(*S*)-2-Oxo-1,2-diphenylethyl 4-methylbenzenesulfonate (20n)

^{1}H NMR (500 MHz, $CDCl_3$)

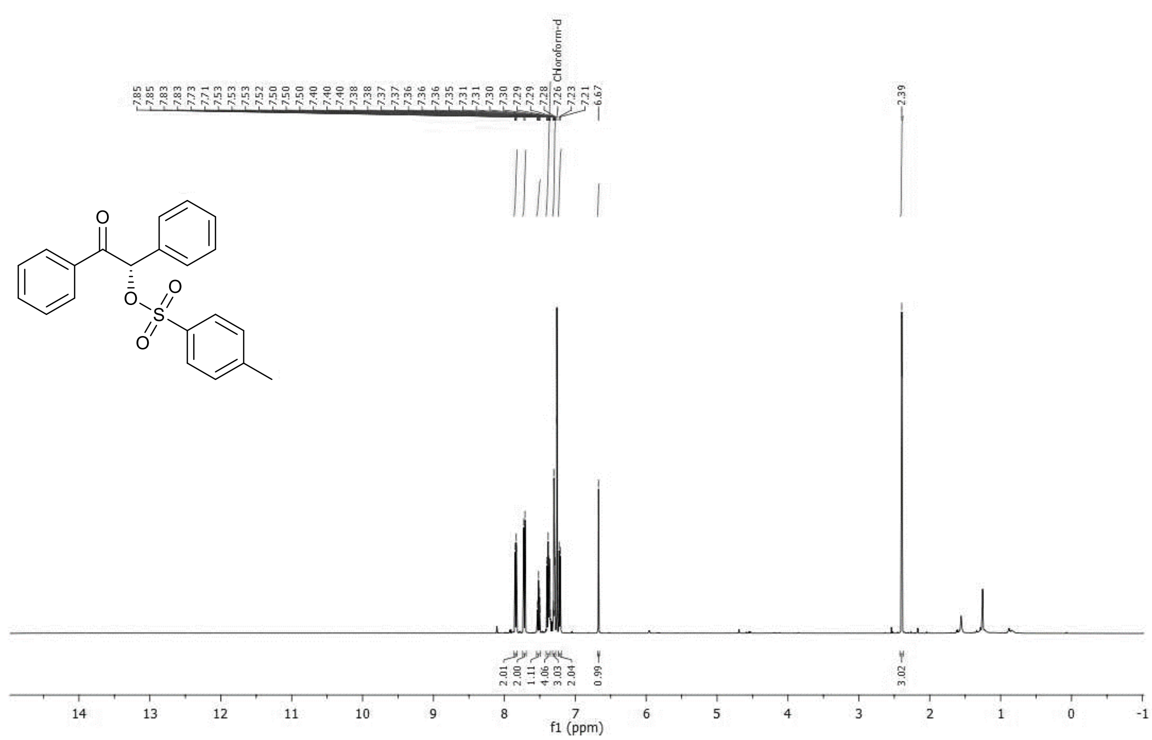

$^{13}C\{^{1}H\}$ NMR (126 MHz, $CDCl_3$)

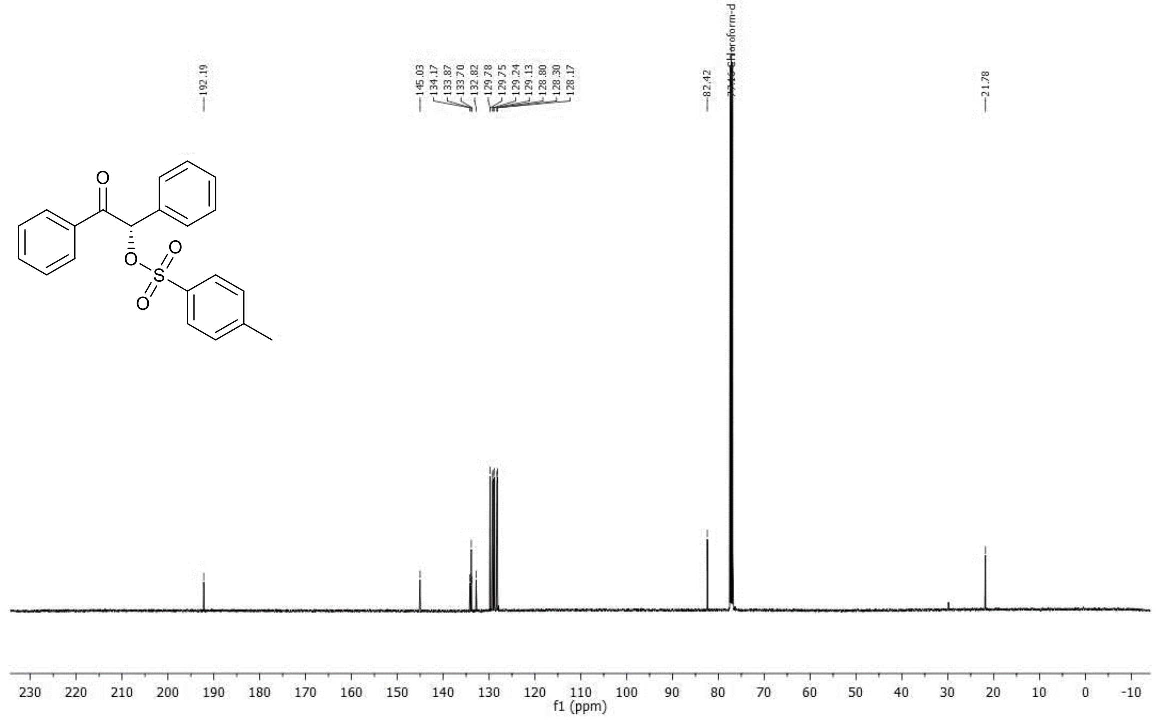

(*S*)-1-Oxo-2,3-dihydro-1H-inden-2-yl 4-methylbenzenesulfonate (20p)

^{1}H NMR (500 MHz, $CDCl_3$)

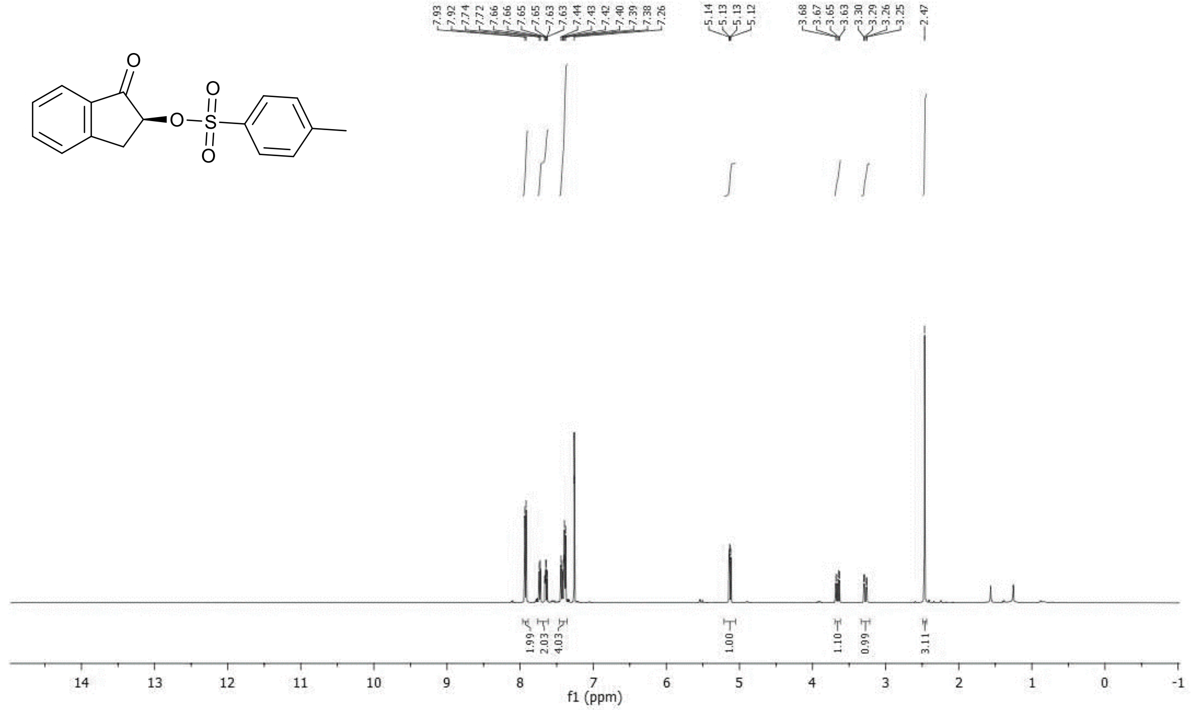

^{13}C{^{1}H} NMR (126 MHz, $CDCl_3$)

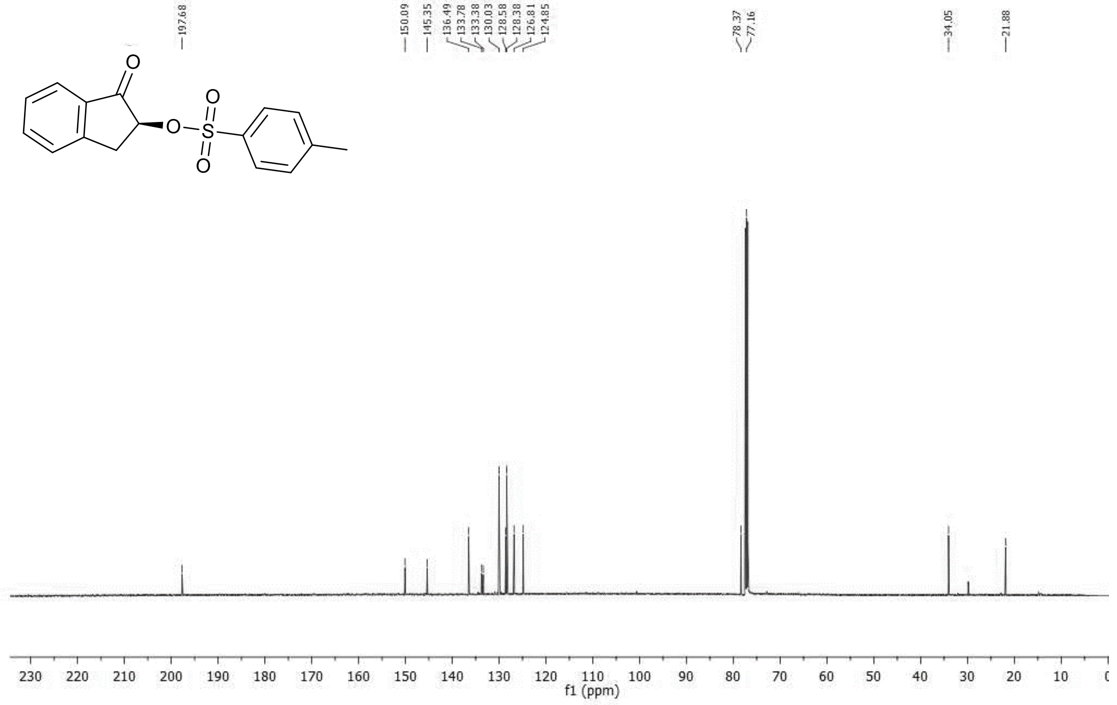

(*S*)-1-Oxo-1,2,3,4-tetrahydronaphthalen-2-yl 4-methylbenzenesulfonate (20q)

^{1}H NMR (400 MHz, $CDCl_3$)

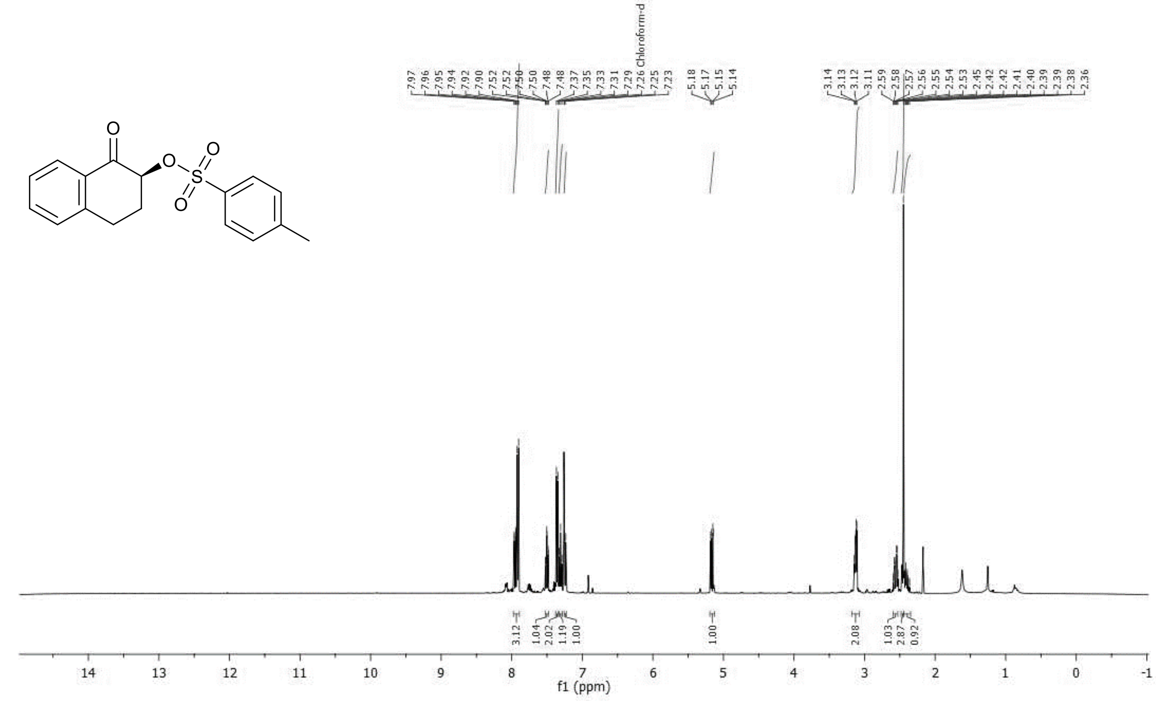

^{13}C{^{1}H} NMR (101 MHz, $CDCl_3$)

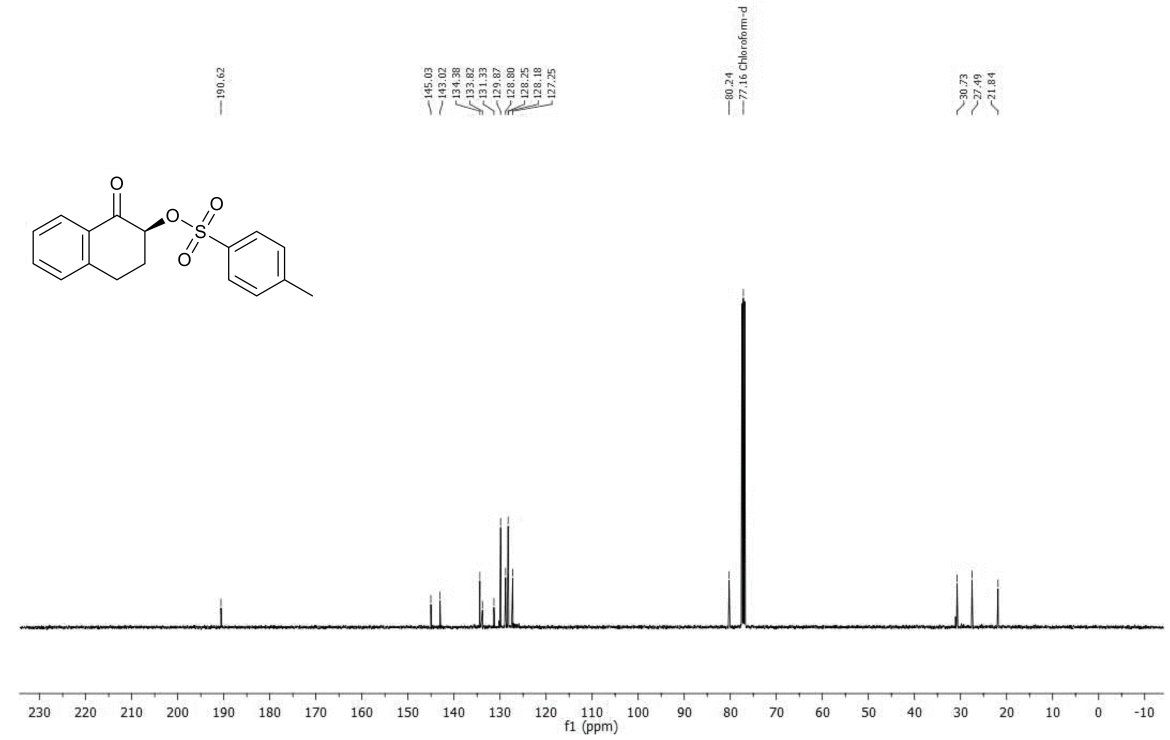

5-Oxo-6,7,8,9-tetrahydro-5H-benzo[7]annulen-6-yl 4-methylbenzenesulfonate (20r)

^{1}H NMR (500 MHz, $CDCl_3$)

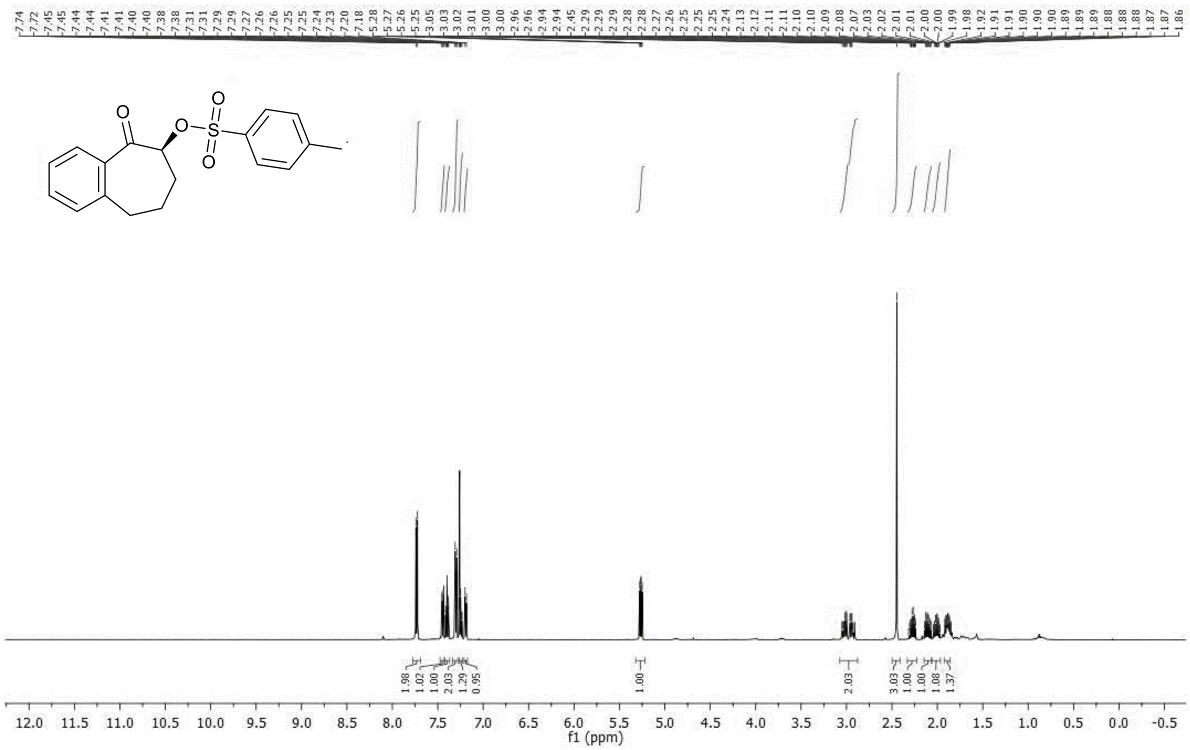

^{13}C{^{1}H} NMR (126 MHz, $CDCl_3$)

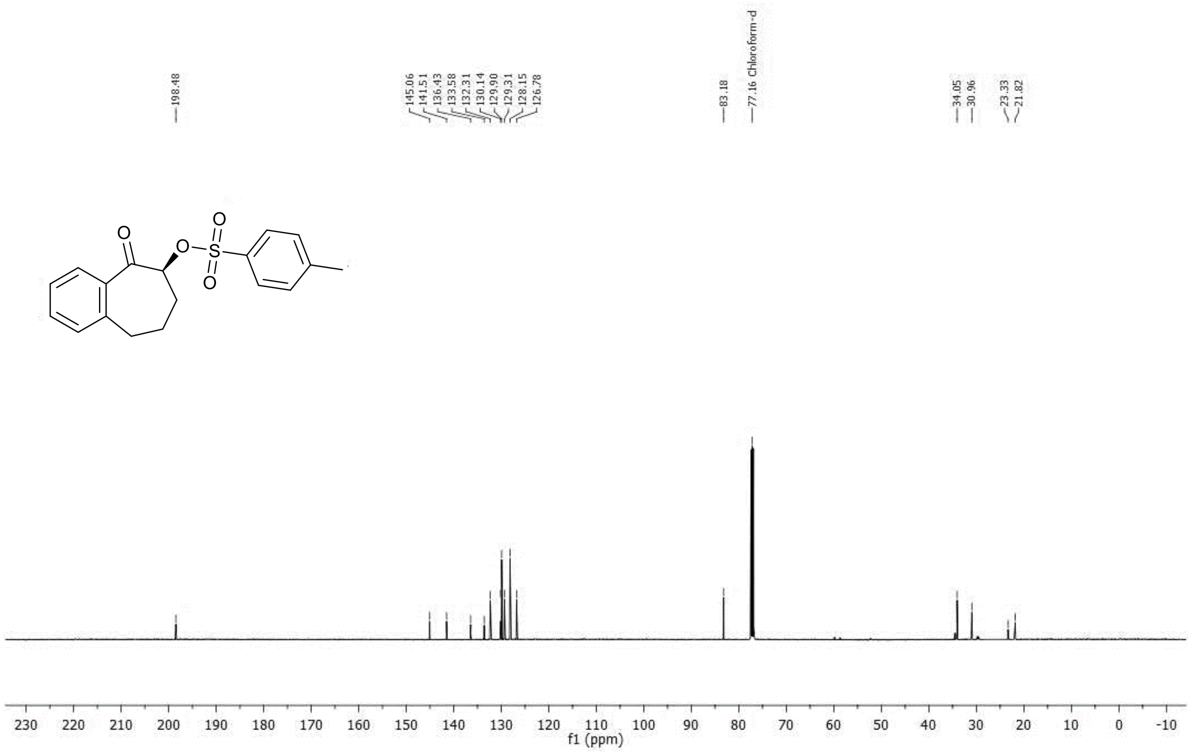

(*S*)-1-(Naphthalen-2-yl)-1-oxopropan-2-yl 4-methylbenzenesulfonate (20s)

^{1}H NMR (400 MHz, $CDCl_3$)

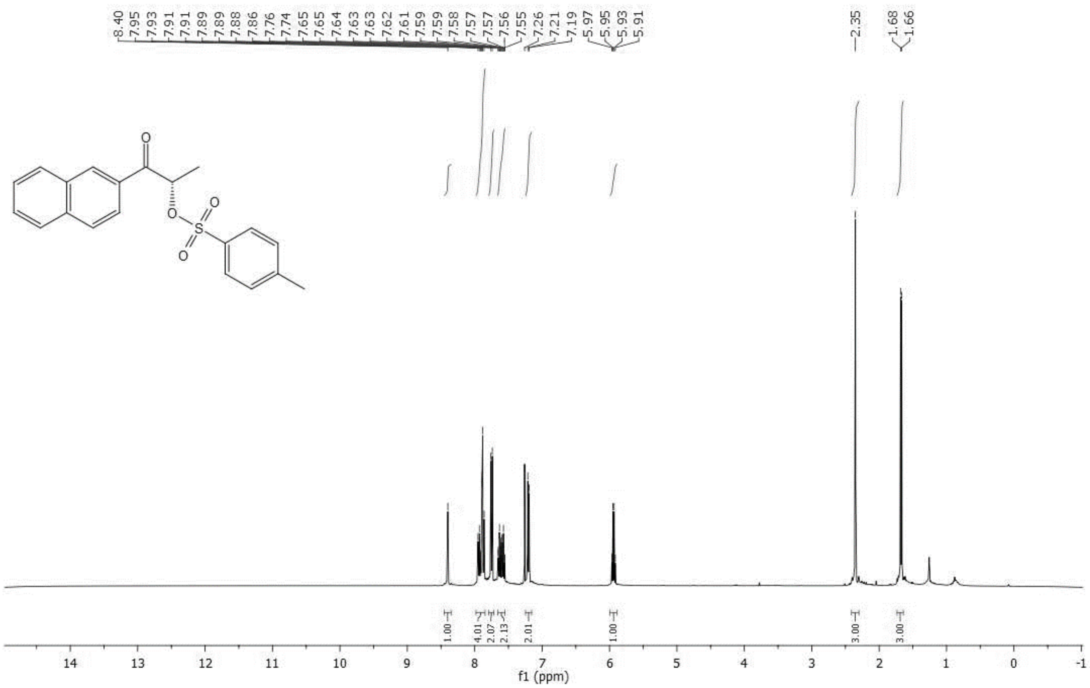

$^{13}C\{^1H\}$ NMR (101 MHz, $CDCl_3$)

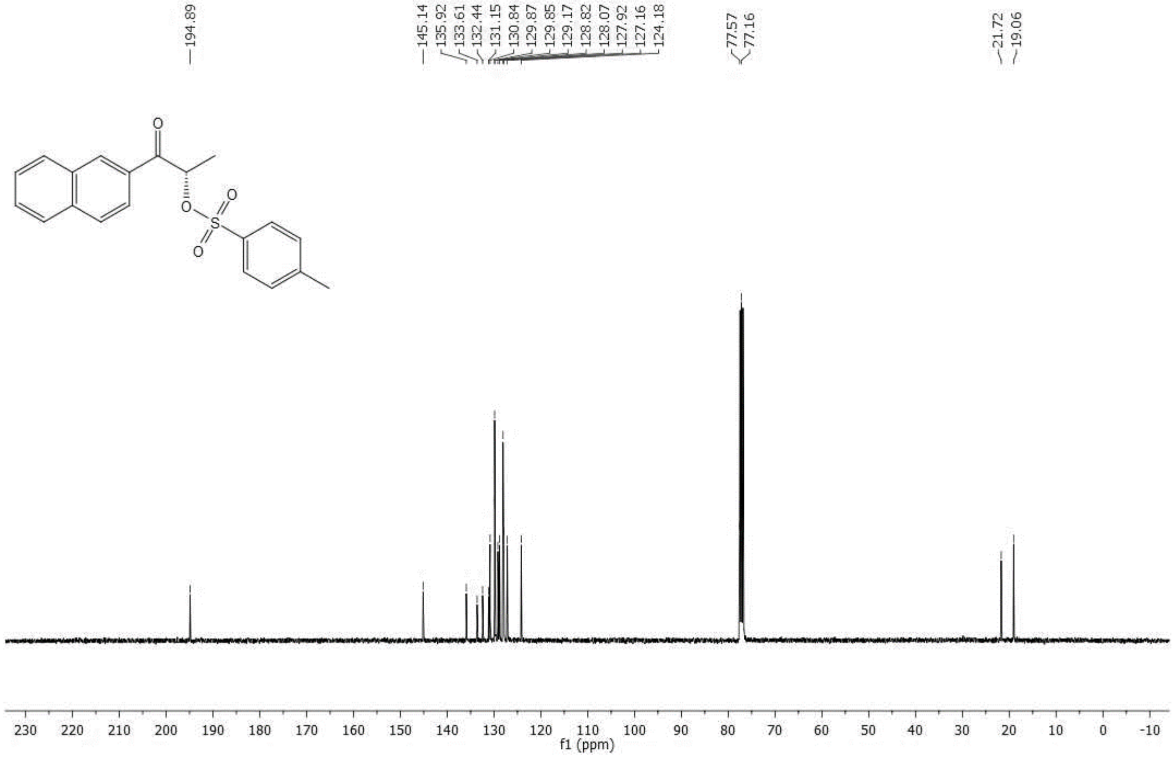

**(S)-1-(Furan-2-yl)-1-oxopropan-2-yl 4-methylbenzenesulfonate (20t)**
^{1}H NMR (500 MHz, $CDCl_3$)

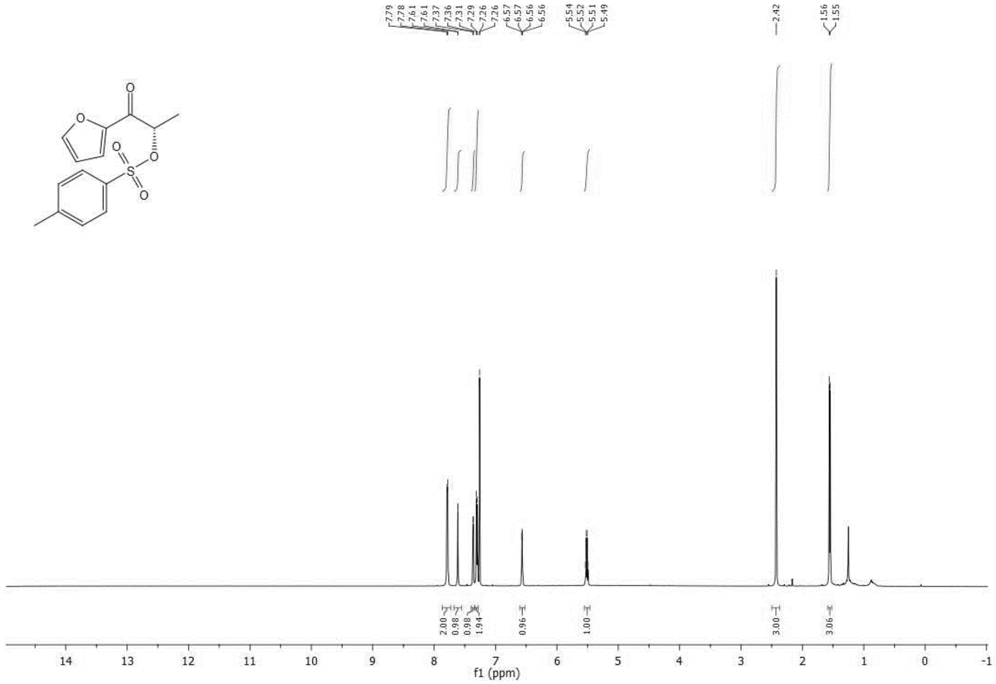

^{13}C{^{1}H} NMR (126 MHz, $CDCl_3$)

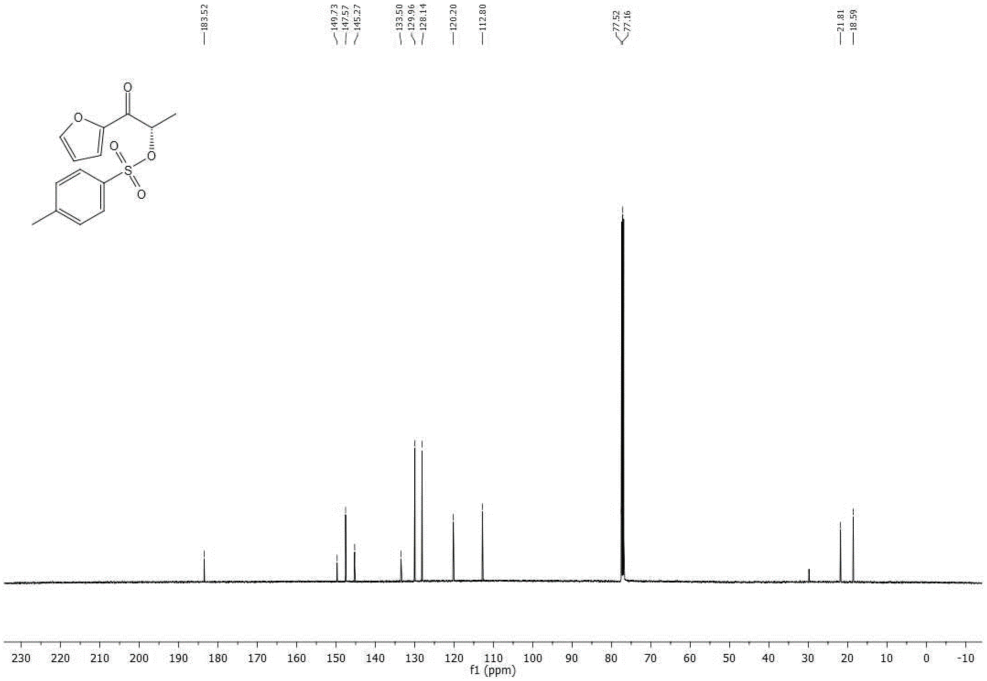

**(*S*)-1-Oxo-1-(thiophen-2-yl)propan-2-yl 4-methylbenzenesulfonate (20u)**

^{1}H NMR (500 MHz, $CDCl_3$)

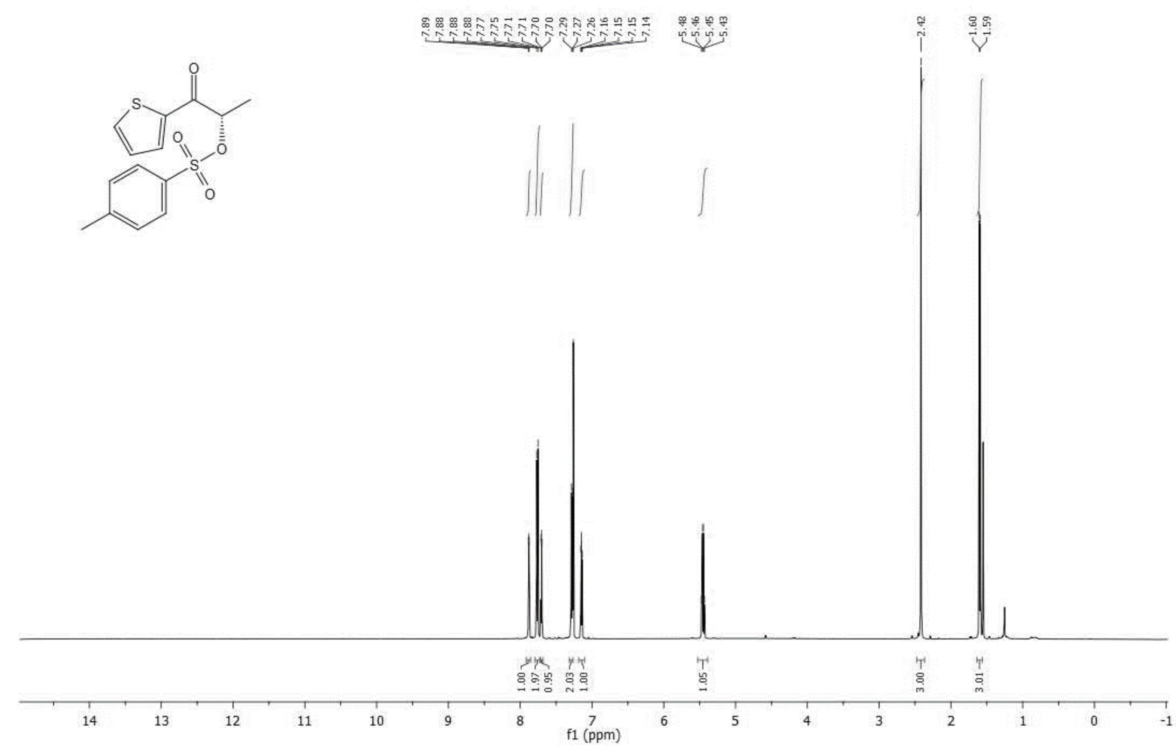

$^{13}C\{^{1}H\}$ NMR (126 MHz, $CDCl_3$)

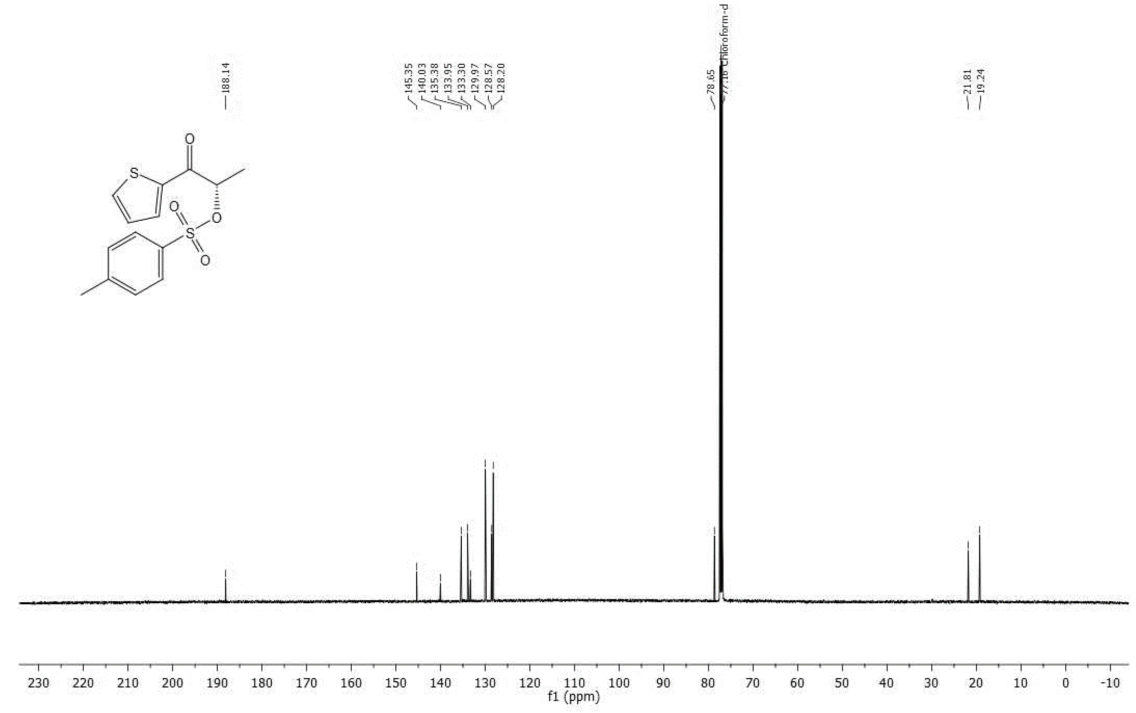

(*S*)-1-Oxo-1-phenylpropan-2-yl benzenesulfonate (20v)

^{1}H NMR (500 MHz, $CDCl_3$)

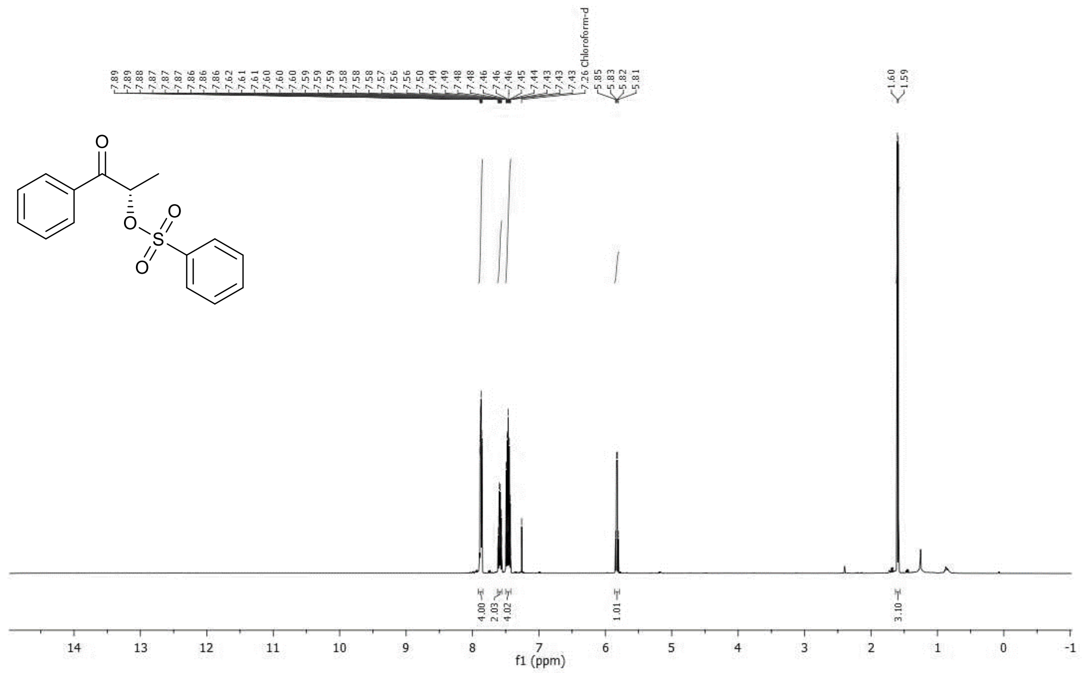

$^{13}C\{^{1}H\}$ NMR (126 MHz, $CDCl_3$)

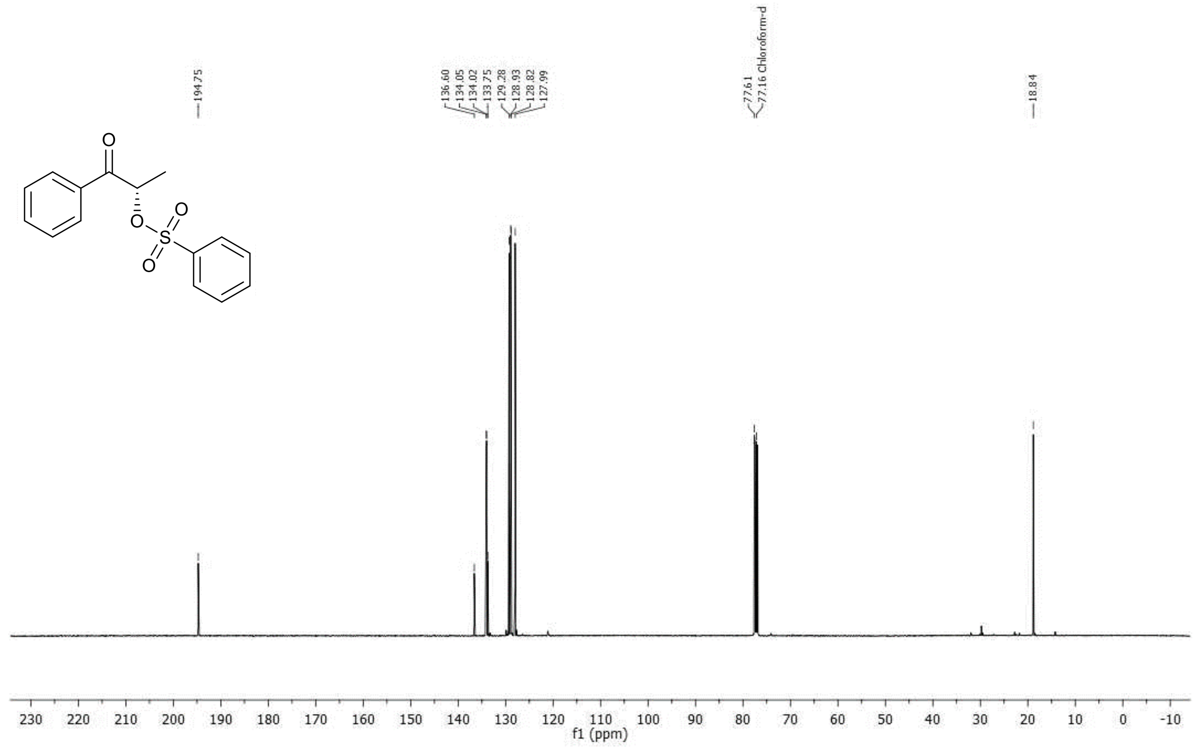

(*S*)-1-Oxo-1-phenylpropan-2-yl 2,4,6-trimethylbenzenesulfonate (20w)

^{1}H NMR (500 MHz, $CDCl_3$)

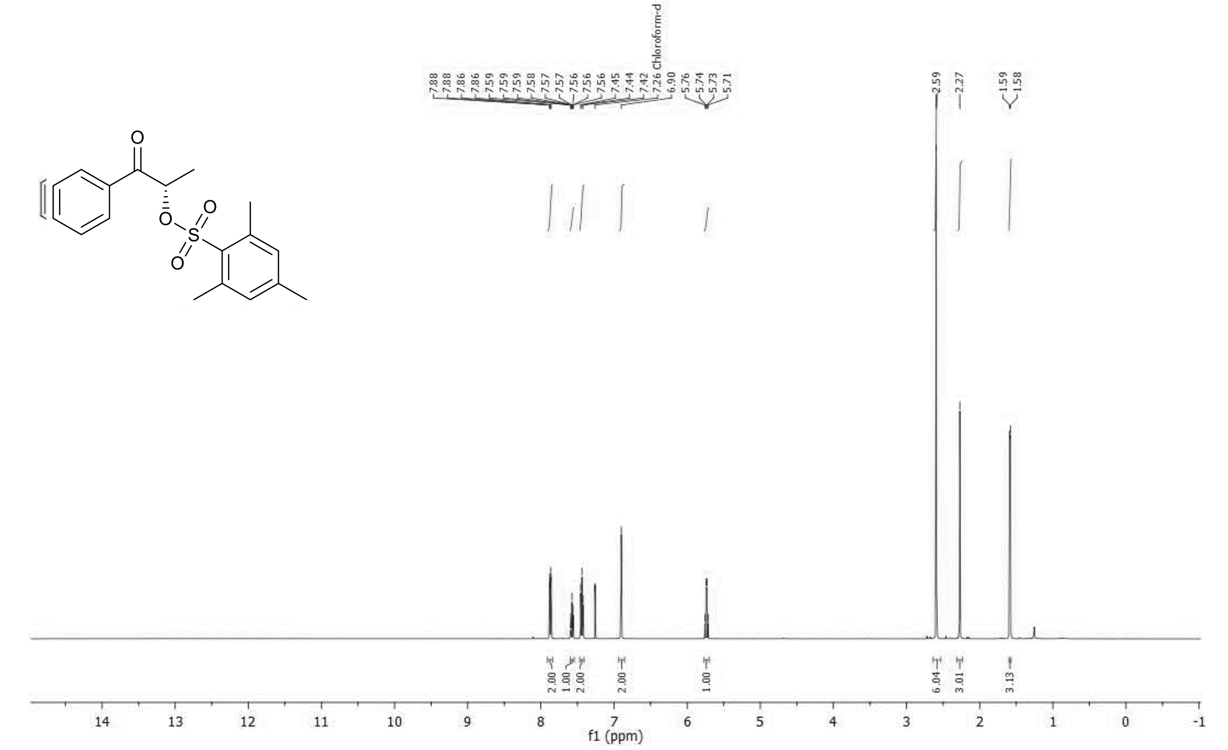

^{13}C{^{1}H} NMR (126 MHz, $CDCl_3$)

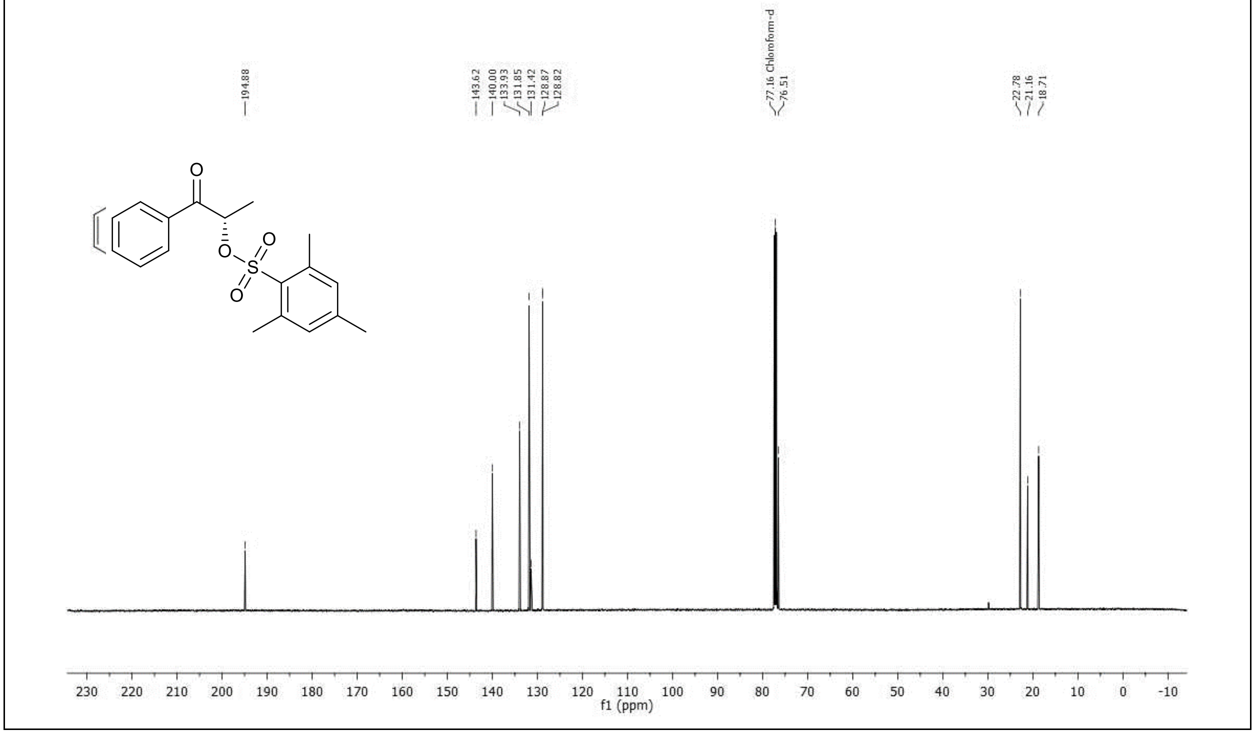

(*S*)-1-Oxo-1-phenylpropan-2-yl methanesulfonate (20x)

^{1}H NMR (500 MHz, $CDCl_3$)

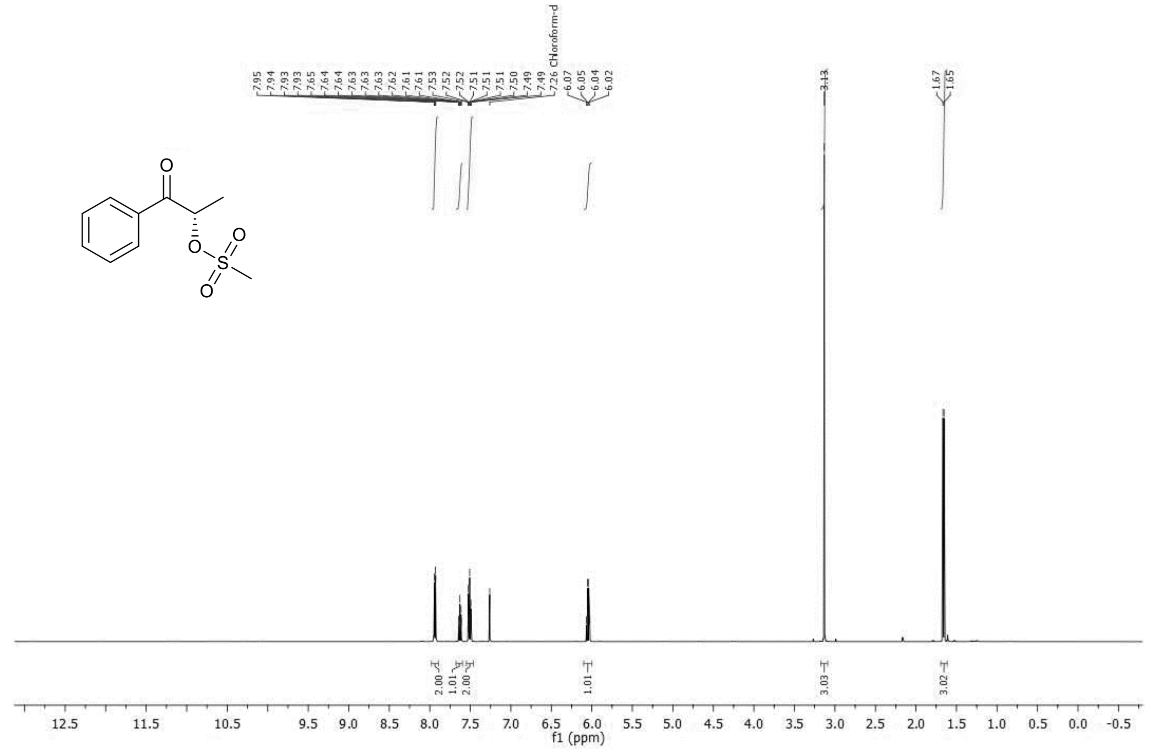

$^{13}C\{^{1}H\}$ NMR (126 MHz, $CDCl_3$)

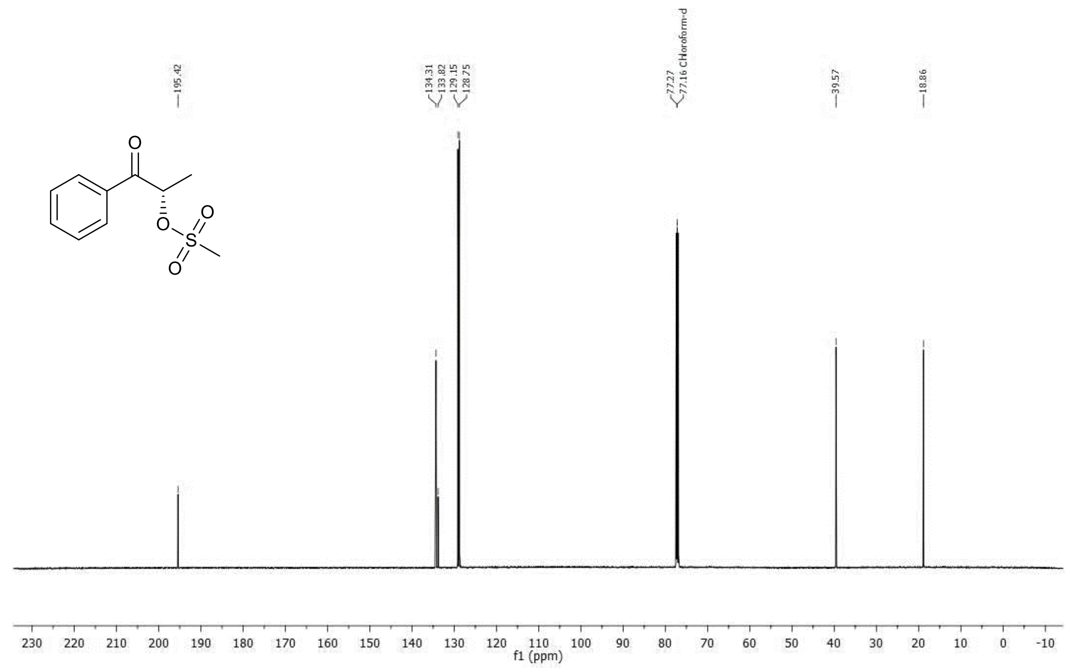

(*S*)-1-Oxo-1-phenylpropan-2-yl 4-chlorobenzenesulfonate (20y)

^{1}H NMR (500 MHz, $CDCl_3$)

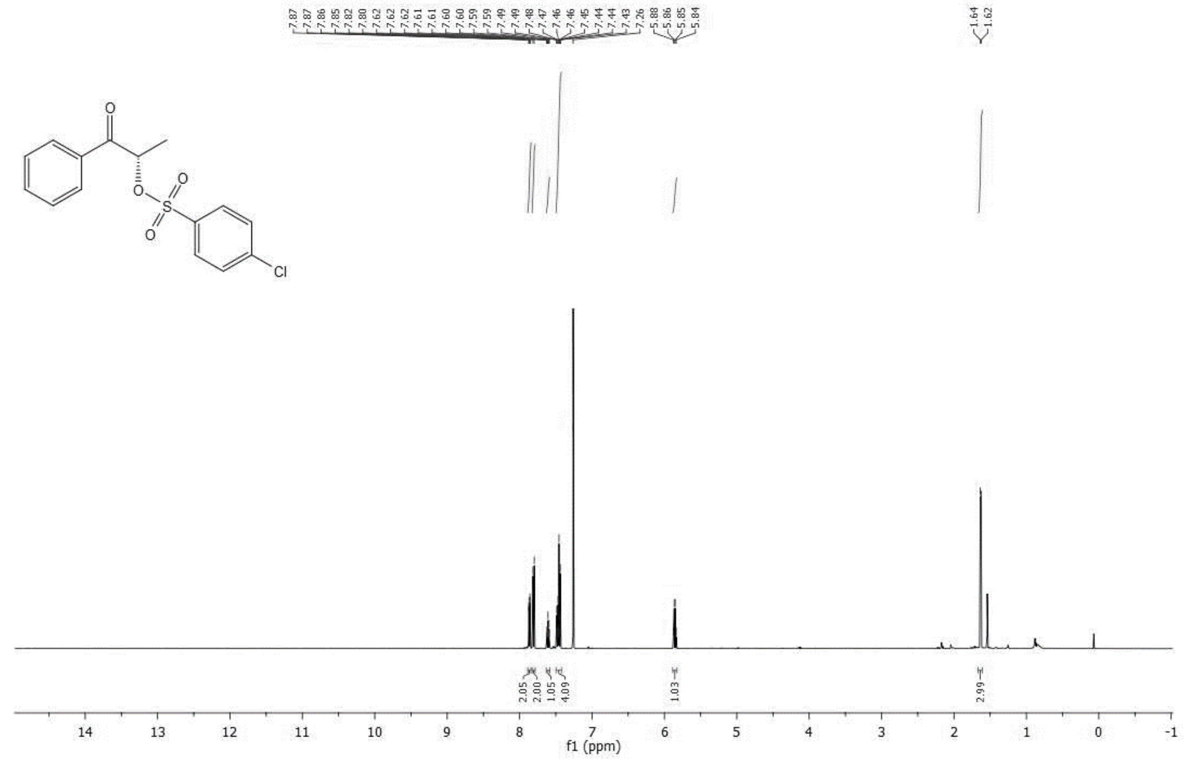

$^{13}C\{^{1}H\}$ NMR (126 MHz, $CDCl_3$)

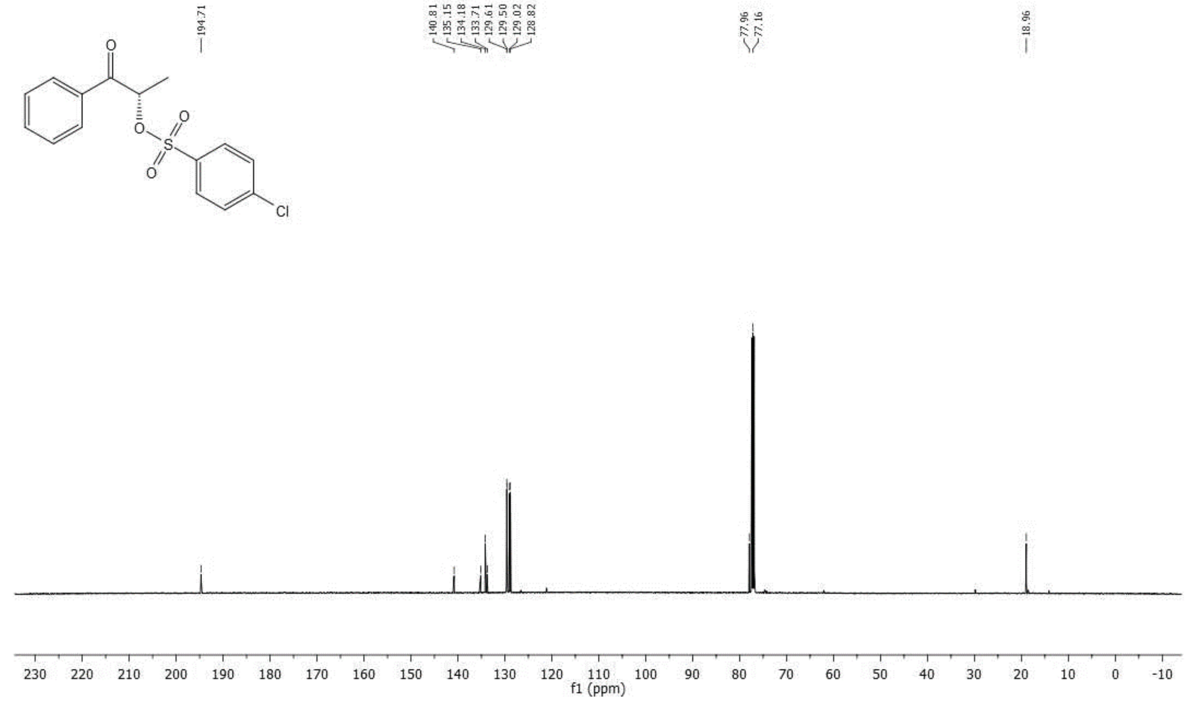

Supplement: Supplementary file 1 — gg3c00012_si_001.pdf [file gg3c00012_si_001.pdf]
